# The impact of inpatient bloodstream infections caused by antibiotic-resistant bacteria in low- and middle-income countries: A systematic review and meta-analysis

**DOI:** 10.1371/journal.pmed.1004199

**Published:** 2023-06-22

**Authors:** Kasim Allel, Jennifer Stone, Eduardo A. Undurraga, Lucy Day, Catrin E. Moore, Leesa Lin, Luis Furuya-Kanamori, Laith Yakob

**Affiliations:** 1 Department of Disease Control, Faculty of Infectious & Tropical Diseases, London School of Hygiene & Tropical Medicine, London, United Kingdom; 2 Antimicrobial Resistance Centre, London School of Hygiene & Tropical Medicine, London, United Kingdom; 3 Institute for Global Health, University College London, London, United Kingdom; 4 Multidisciplinary Initiative for Collaborative Research in Bacterial Resistance (MICROB-R), Santiago, Chile; 5 JBI, Faculty of Health and Medical Sciences, The University of Adelaide, Adelaide, South Australia, Australia; 6 Escuela de Gobierno, Pontificia Universidad Católica de Chile, Santiago, Chile; 7 CIFAR Azrieli Global Scholars Program, CIFAR, Toronto, Canada; 8 Research Center for Integrated Disaster Risk Management (CIGIDEN), Santiago, Chile; 9 The Centre for Neonatal and Paediatric Infection, Infection and Immunity Institute, St George’s, University of London, London, United Kingdom; 10 Department of Infectious Disease Epidemiology, Faculty of Epidemiology and Population Health, London School of Hygiene & Tropical Medicine, London, United Kingdom; 11 Laboratory of Data Discovery for Health (D24H), Hong Kong Science Park, Hong Kong Special Administrative Region, China; 12 The University of Hong Kong, Hong Kong Special Administrative Region, China; 13 UQ Centre for Clinical Research, Faculty of Medicine, The University of Queensland, Herston, Australia

## Abstract

**Background:**

Bloodstream infections (BSIs) produced by antibiotic-resistant bacteria (ARB) cause a substantial disease burden worldwide. However, most estimates come from high-income settings and thus are not globally representative. This study quantifies the excess mortality, length of hospital stay (LOS), intensive care unit (ICU) admission, and economic costs associated with ARB BSIs, compared to antibiotic-sensitive bacteria (ASB), among adult inpatients in low- and middle-income countries (LMICs).

**Methods and findings:**

We conducted a systematic review by searching 4 medical databases (PubMed, SCIELO, Scopus, and WHO’s Global Index Medicus; initial search *n* = 13,012 from their inception to August 1, 2022). We only included quantitative studies. Our final sample consisted of *n* = 109 articles, excluding studies from high-income countries, without our outcomes of interest, or without a clear source of bloodstream infection. Crude mortality, ICU admission, and LOS were meta-analysed using the inverse variance heterogeneity model for the general and subgroup analyses including bacterial Gram type, family, and resistance type. For economic costs, direct medical costs per bed-day were sourced from WHO-CHOICE. Mortality costs were estimated based on productivity loss from years of potential life lost due to premature mortality. All costs were in 2020 USD. We assessed studies’ quality and risk of publication bias using the MASTER framework. Multivariable meta-regressions were employed for the mortality and ICU admission outcomes only. Most included studies showed a significant increase in crude mortality (odds ratio (OR) 1.58, 95% CI [1.35 to 1.80], *p* < 0.001), total LOS (standardised mean difference “SMD” 0.49, 95% CI [0.20 to 0.78], *p* < 0.001), and ICU admission (OR 1.96, 95% CI [1.56 to 2.47], *p* < 0.001) for ARB versus ASB BSIs. Studies analysing Enterobacteriaceae, *Acinetobacter baumanii*, and *Staphylococcus aureus* in upper-middle-income countries from the African and Western Pacific regions showed the highest excess mortality, LOS, and ICU admission for ARB versus ASB BSIs per patient. Multivariable meta-regressions indicated that patients with resistant *Acinetobacter baumanii* BSIs had higher mortality odds when comparing ARB versus ASB BSI patients (OR 1.67, 95% CI [1.18 to 2.36], *p* 0.004). Excess direct medical costs were estimated at $12,442 (95% CI [$6,693 to $18,191]) for ARB versus ASB BSI per patient, with an average cost of $41,103 (95% CI [$30,931 to $51,274]) due to premature mortality. Limitations included the poor quality of some of the reviewed studies regarding the high risk of selective sampling or failure to adequately account for relevant confounders.

**Conclusions:**

We provide an overview of the impact ARB BSIs in limited resource settings derived from the existing literature. Drug resistance was associated with a substantial disease and economic burden in LMICs. Although, our results show wide heterogeneity between WHO regions, income groups, and pathogen–drug combinations. Overall, there is a paucity of BSI data from LMICs, which hinders implementation of country-specific policies and tracking of health progress.

## Abstract 2

Kasim Allel and colleagues systematically review published literature from low- and middle-income countries and meta-analyse data extracted from 109 articles to explore the impact of bloodstream infections caused by antibiotic-resistant bacteria.

## Author summary

**Why was this study done?:**

- Bloodstream infections (BSIs) caused by antibiotic-resistant bacteria (ARB) have multifaceted impacts, including higher admission to intensive care units (ICUs), prolonged hospitalisations, and high economic and societal costs worldwide.
- Despite the global burden, most evidence on the excess burden of ARB BSIs has been derived from high-income countries; comparatively, there are limited data from low- and middle-income countries (LMICs).

**What did the researchers do and find?:**

- We employed a systematic literature review and subsequent meta-analysis of 109 individual studies to quantify the impact of ARB BSIs in hospitalised patients from LMICs.
- Based mostly on crude data comparisons ignoring the possible influence of confounding factors, we found that ARB BSIs, compared to BSIs caused by antibiotic-sensitive bacteria (ASB), were associated with substantially longer stays in hospitals and ICUs, higher mortality, and increased direct medical and productivity costs.

**What do these findings mean?:**

- Our findings highlight the excess morbidity, mortality, and costs associated with ARB BSIs and the sparsity of data from LMICs.
- Targeted strategies to improve the prevention, detection, and treatment of resistant BSIs in LMICs are required to reduce the economic and disease burden.

## Introduction

Antibiotic-resistant bacteria (ARB) constitute a global health priority, particularly where resistance proportion is highest in low- and middle-income countries (LMICs) [1]. Resource-limited hospital infrastructure, poor health system capacity, and inadequate sanitation and hygiene infrastructure partly explain the spread and impact of ARB in LMICs [1,2]. Ameliorating health inequities is hampered by the feedback caused by ARB infections resulting in increased morbidity and mortality, more complicated treatments due to the use of reserved antibiotics, and prolonged hospitalisations, all of which exacerbate costs to countries’ health systems and society [1,3]. Recent figures from the World Health Organization (WHO) Global Antimicrobial Resistance and Surveillance System (GLASS) report show that the proportion of *Escherichia coli* bloodstream infections (BSIs) caused by third-generation cephalosporins resistant *E*. *coli* was more than triple in LMICs compared to high-income countries, (58.3% and 17.53%, respectively) [4]. A similar trend was observed for other WHO critical- and high-priority BSI pathogens, including *Klebsiella pneumoniae* and *Staphylococcus aureus* [4,5].

BSIs are one of the most lethal infections, having an estimated overall crude mortality of 15% to 30% [4,6]. BSIs are intrinsically more deadly as pathogens can spread quickly via blood, producing multiple infections and leading to organ damage and dysfunction. Extensive literature has examined the excess burden of ARB BSIs in specific locations [7–13]. For example, compared to their sensitive counterparts, carbapenem-resistant *Klebsiella* spp. [12] and methicillin-resistant *Staphylococcus aureus* (MRSA) [11] BSIs are associated with 9.08 (95% CI [1.17 to 70.51]) and 2.23 (95% CI [1.14 to 4.37]) times greater mortality, respectively. Higher admission to the intensive care units (ICUs), (OR 8.57; 95% CI [3.99 to 18.38]), greater length of hospital stay (LOS), (4.89 additional days; 95% CI [0.56 to 11.52]) and sizeable hospital costs ($23,318, 95% CI [$858 to $57,090]) have been linked to vancomycin-resistant versus -sensitive *Enterococci* BSIs [13]. Studies conducted in high-income countries contribute disproportionately to these estimates [14–16]; data from LMICs are scant. This comprises a critical gap in our understanding of the impact of drug-resistant BSI in countries with higher underlying health risks (e.g., cancer, neutropenia and haematological malignancies, pneumonia, and diabetes) [17].

Here, we present a systematic review and meta-analysis of the literature on the impact (i.e., LOS, mortality, and ICU admission) and excess economic costs per patient associated with ARB BSI compared with antibiotic-sensitive (ASB) BSI among hospitalised patients in LMICs.

## Methods

This study is reported as per the Preferred Reporting Items for Systematic Reviews and Meta-Analyses (PRISMA) guideline (S1 Checklist) [18] and was prospectively registered with PROSPERO (id number: CRD42021264056).

### Search strategy

We searched the literature for studies examining the burden of ARB BSIs compared with ASB BSIs among inpatients from LMICs. PubMed, SCIELO, Scopus, and WHO’s Global Index Medicus (Latin American and Caribbean Health Sciences Literature “LILACs” and African Index Medicus “AIM”) were searched without restrictions to language or year of publication using a family of keywords related to antibiotic/drug-resistance, bloodstream infections/bacteraemia, and burden measures among inpatients. We searched articles published through August 1, 2022. The complete list of terms, abbreviations, and Boolean connectors used by search engine can be found in the Supporting information (S1 Text, section 1).

### Study selection

We selected articles according to a step-guided protocol. First, articles were excluded if carried out in high-income countries; these were defined according to the 2021 World Bank classification list (i.e., gross national income “GNI” per capita > $12,696) [19]. Second, studies were only included if BSIs were presented based on laboratory-confirmed positive blood cultures. Either primary or secondary BSIs were included. Articles that analysed patients with different culture types (e.g., blood, urine, wound, nasal) were removed unless BSI episodes were clearly detailed. Third, articles were included if the ASB and ARB groups were identified among adult patients presenting BSIs in the hospital. Fourth, participants with chronic or severe diseases (e.g., HIV, cancer) were removed unless they were present in the ARB and ASB groups (e.g., studies were withdrawn if HIV–positive patients having ARB BSIs were compared with HIV–negative patients having ASB BSIs). Finally, studies were removed if they did not present our selected outcomes (i.e., mortality, ICU admission, LOS, or costs). Experimental and observational articles were included. We removed correspondence letters or opinions, short reports without data analysis, literature reviews, and single-case studies.

Studies were analysed only when the number of patients was reported. We only included the adult population (average ≥18 years of age) because (i) the number of studies focusing on children was limited (*n* = 4) after looking at the provisional results; and (ii) children’s inherent behaviour and exposure level differ from adults [3]. Only data on WHO-priority pathogens were retained [20]. The Results section (PRISMA chart) and Table A in S1 Text present the complete list of search criteria used.

To avoid our study hinging only on published articles’ results, we systematically reviewed the grey literature and other current literature reviews analysing similar topics. Four referees resolved any disagreement presented at any stage of study selection through scholarly discussion. Two native Spanish speakers fluent in Portuguese and English, a native English speaker, and a native Chinese speaker fluent in English conducted the screening and consecutive data extraction. Papers written in any other language were translated to English using Google Translate PDF (<1% of the included articles). We used the Rayyan free online tool (https://rayyan.ai/) to screen, select, and decide which articles were included. Double article screening for eligibility was employed, and discrepancies were resolved via scholarly dialogue.

### Data extraction

We extracted data including authors, publication year, country, study setting, population characteristics, bacterium type, resistance type, and sample sizes (for cases and control groups). We classified pathogen resistance based on the specific pathogen-resistance profiles evaluated in each study (e.g., cephalosporin-resistant *Acinetobacter baumanii*). For completeness, we also collated data on ESBL+ and non-ESBL (ESBL-) groups for gram-negative pathogens. For the analysis, the case group comprised infections with resistant strains (ARB), whereas the control group comprised sensitive-strain infections (ASB). Selected studies were organised using unique identifiers (e.g., 1, 2, 3), and sub-studies within the primary articles were classified using consecutive numbers separated by a dot (e.g., 1.1, 1.2, 1.3) if they presented bacterium- or resistance type-specific information (S1 Data).

We extracted the following outcomes by case/control group: mortality (crude 30-day mortality, whenever available, or overall crude mortality if timing was not reported), LOS (average total days and standard deviation), and ICU admission (patients admitted). We also collected data on demographics and underlying conditions: average age, previous surgery and hospitalisation, community- or hospital-acquired BSI, any underlying condition (diabetes, hypertension, cardiovascular or heart diseases, solid tumour or malignancy, liver or kidney disease, pulmonary/respiratory diseases, and any hematologic disease), and BSI source (urinary tract, intravenous or catheter, pulmonary, and intrabdominal or gastrointestinal). Pitt bacteraemia score, APACHE II, and CHARLSON scores were collected if presented. We compared ARB and ASB groups by comparing variables’ proportion or mean using McNemar’s χ^2^ or T-tests for binary and continuous data, respectively. Additionally, we classified the studies by World Bank income level, WHO region, WHO Global Priority Pathogens List, bacterium family and antibiotic class, pathogen strain, and bacterium Gram type. We used Microsoft Excel 2022 to compile and extract included articles’ data. We used double data extraction reviewing, and inconsistencies (14% disagreement) were resolved through scholarly discussion.

### Study quality and risk assessment

We used a unified framework to evaluate the methodological quality of analytic study designs (MASTER scale) [21]. This framework comprises 36 questions classified into 7 domains concerning equal recruitment, retention, implementation, prognosis, ascertainment, sufficient analysis, and temporal precedence. Each question was scored independently by 2 reviewers as 1 if the study complied with the domain or 0 if it did not. Therefore, a higher score indicates higher study quality. Two independent reviewers performed a risk of bias assessment. Conflicts were addressed through scholarly discussion.

### Statistical analysis

Firstly, we employed population-weighted descriptive statistics of the health and demographic characteristics collated by studies’ patients having ARB and ASB BSIs to contrast both groups and check whether mean differences across patient features existed. Secondly, the overall estimates for excess mortality, ICU admission, and LOS associated with resistant strains compared to their sensitive counterparts were meta-analysed using the inverse variance heterogeneity model [22]. The heterogeneity was calculated using the I^2^ statistics; I^2^ values were classified as high (>75%), moderate (50% to 75%), and low (<50%) heterogeneity. All results were computed using odds ratios (ORs) for mortality and ICU admission rates, and the standardised mean difference (SMD) for LOS. We estimated ORs based on studies’ crude numbers or unadjusted ORs provided. Forest plots and meta-analyses were computed by outcome and subgroups of variables, including bacterial family, Gram type, reported resistance type, most common antibiotic-resistant microbial strains, World Bank income group, and WHO region. *P*-values (p) were reported using a two-tailed *t* test (*p* < 0.05) for the ORs for mortality and ICU admissions and LOS’s standardised mean difference. We also analysed and compared, whenever reported, the unadjusted and confounder-adjusted ORs, for studies reporting univariate and multivariable regression analyses.

As a secondary analysis, we used univariate and multivariable meta-regressions to explore the main determinants of mortality and ICU admission (LOS was not included because of a small sample size). We included the bacterial family and resistance profile, demographics, and underlying health condition variables in the univariate regression. Variables were transformed to odds between ARB and ASB groups. We evaluated the associations with the original and fully imputed observations. Multiple imputations were performed using fully completed data as factors and with 1,000 repetitions following a multivariable normal regression design. Variables associated with our outcomes in the univariate analysis with *p* < 0.05 using non-imputed data were included in the fully imputed multivariable model.

Excess economic costs per patient (i.e., costs associated with ARB BSI minus costs associated with ASB BSI) were computed only for excess length of stay, separated by ICU and non-ICU wards. Hospital-day costs included all the inpatient hospitality costs per patient stay for primary and secondary level and teaching hospitals and were calculated based on WHO-CHOICE costs [23]. ICU costs were calculated per patient stay for tertiary/teaching hospitals and were retrieved from the literature for countries with available information [24–36], or by using an approximation ratio between hospital and ICU costs [37–39]. Direct medical costs comprised hospital-day and ICU admission costs per patient, adjusted to their respective patients’ LOS in the hospitalised or ICU services. We also calculated excess productivity losses per patient associated with premature mortality from ARB BSIs (compared to ASB BSIs) using the life expectancy at death and human capital approaches [40]. Excess productivity losses associated with premature mortality costs were computed by multiplying the years of life lost, based on the reference standard life expectancy at the average age of death [41] from ARB BSI (i.e., costs associated with ARB BSI minus costs associated with ASB BSI), using the study-weighted average age for all patients over all studies, without age-weights and a 5% time discount [42]. All costs were expressed in 2020 USDs, adjusting for inflation using US GDP implicit price deflators. Due to a lack of data, we excluded direct and indirect nonmedical costs (e.g., travel). Cost computations and methods are detailed in S1 Text, section 4.

### Small-study effects

The Doi [43] plots and the LFK index were used to evaluate small-study effects when there were at least 5 studies in the meta-analysis. Leave-one-out cross-validation [44] was used to estimate the generalisation performance of our main meta-analyses to cross-validate the results’ sensitivity.

### Sensitivity analyses

We evaluated whether our main meta-analysis results varied by location. Due to the large proportion of studies from China (*N* = 41), we assessed our meta-analyses by separating our sampled studies into those performed in China and other LMICs.

All statistical analyses included studies and sub-studies according to their specific population features and were performed in Stata 17, College Station, TX: StataCorp LLC.

## Results

### Yield of the search strategy

**Fig 1:**
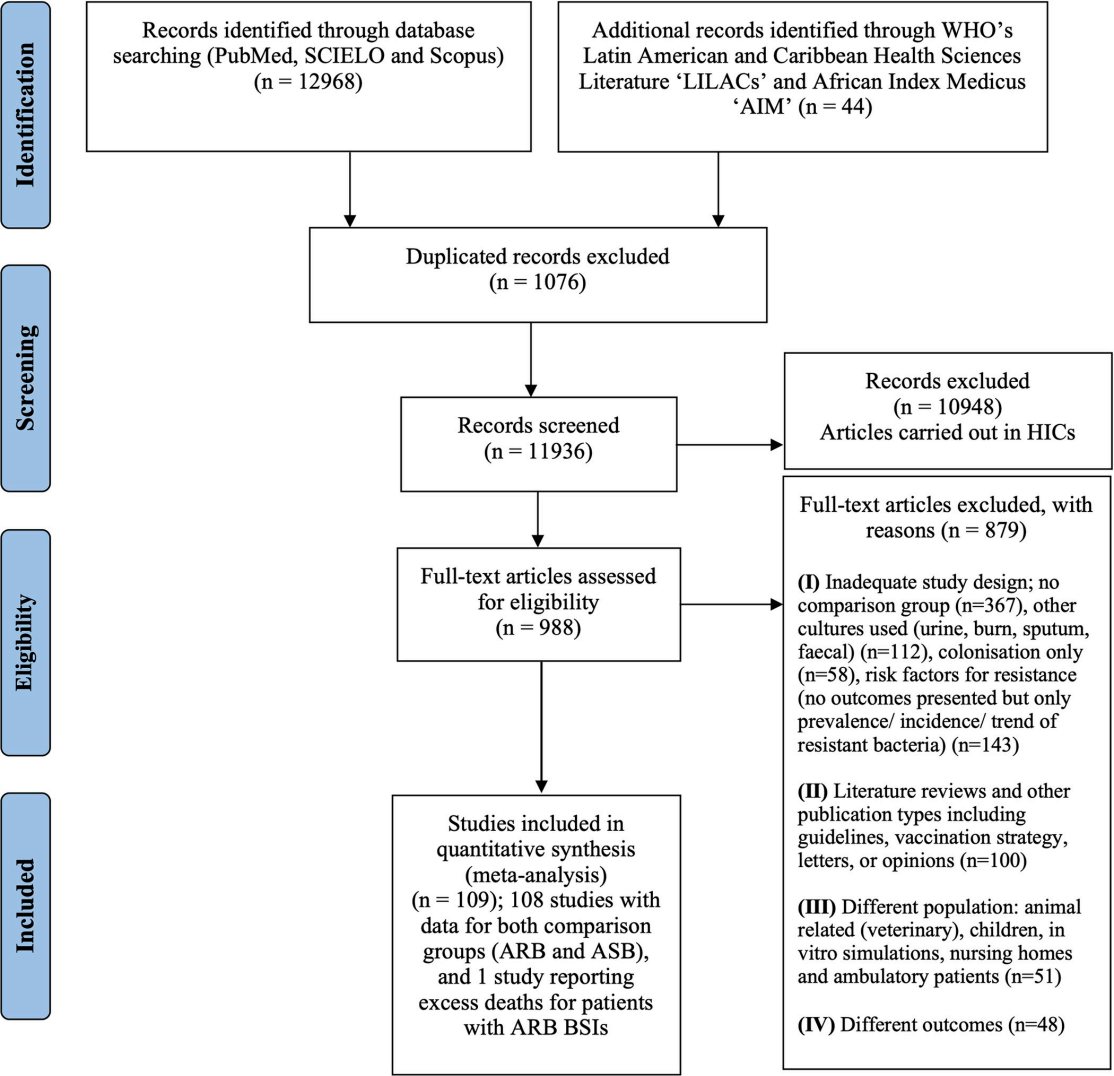
Flowchart detailing systematic review according to PRISMA guidelines. PRISMA: Preferred Reporting Items for Systematic Reviews and Meta-Analyses guidelines [18]. HICs: High-income countries. PRISMA checklist is provided in S1 Text. ARB, antibiotic-resistant bacteria; ASB, antibiotic-sensitive bacteria; BSI, bloodstream infections; WHO, World Health Organization.

Our search strategy identified 13,012 articles: 4,720 through PubMed, 8,193 in Scopus, 55 in SCIELO, and 44 in AIM and LILACs (Fig 1). Of these, 1,076 were duplicated (8.3%; 1,076/13,012), and 10,948 were performed in high-income countries (84.1%; 10,948/13,012) and hence removed. In total, 988 articles were full-text screened, resulting in the inclusion of 109 studies (*N* = 22,756 patients).

### Characteristics of included studies

**Fig 2:**
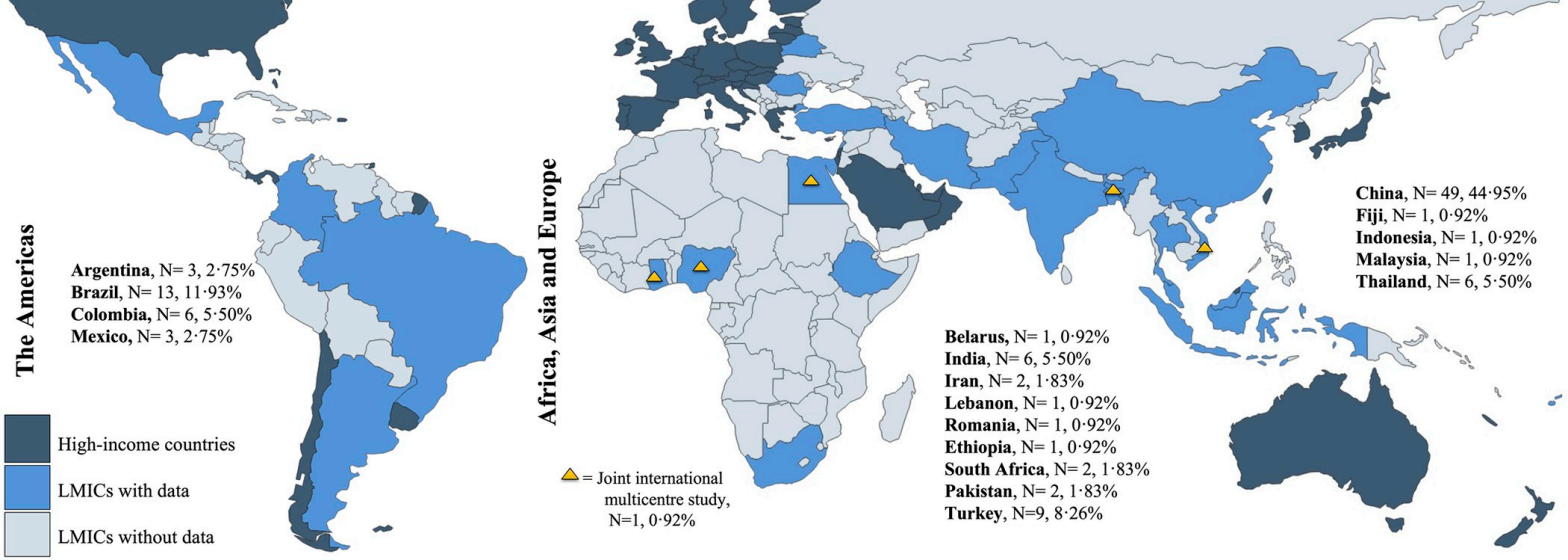
Distribution of the included studies according to country (*N* = 109 articles). Maps indicate the country where studies came from with their respective number (*N*) of studies included and the percentage of studies per country of the total studies analysed. Joint studies used cross-country designs (i.e., analysed ARB BSIs in more than 1 country). White areas represent high-income countries or missing LMICs. Maps were computed in QGIS Development Team (2020), Geographic Information System, version 3.16: Open-Source Geospatial Foundation Project. http://qgis.osgeo.org. ARB, antibiotic-resistant bacteria; BSI, bloodstream infection; LMIC, low- and middle-income country; QGIS, Quantum Geographic Information System.

**Fig 3:**
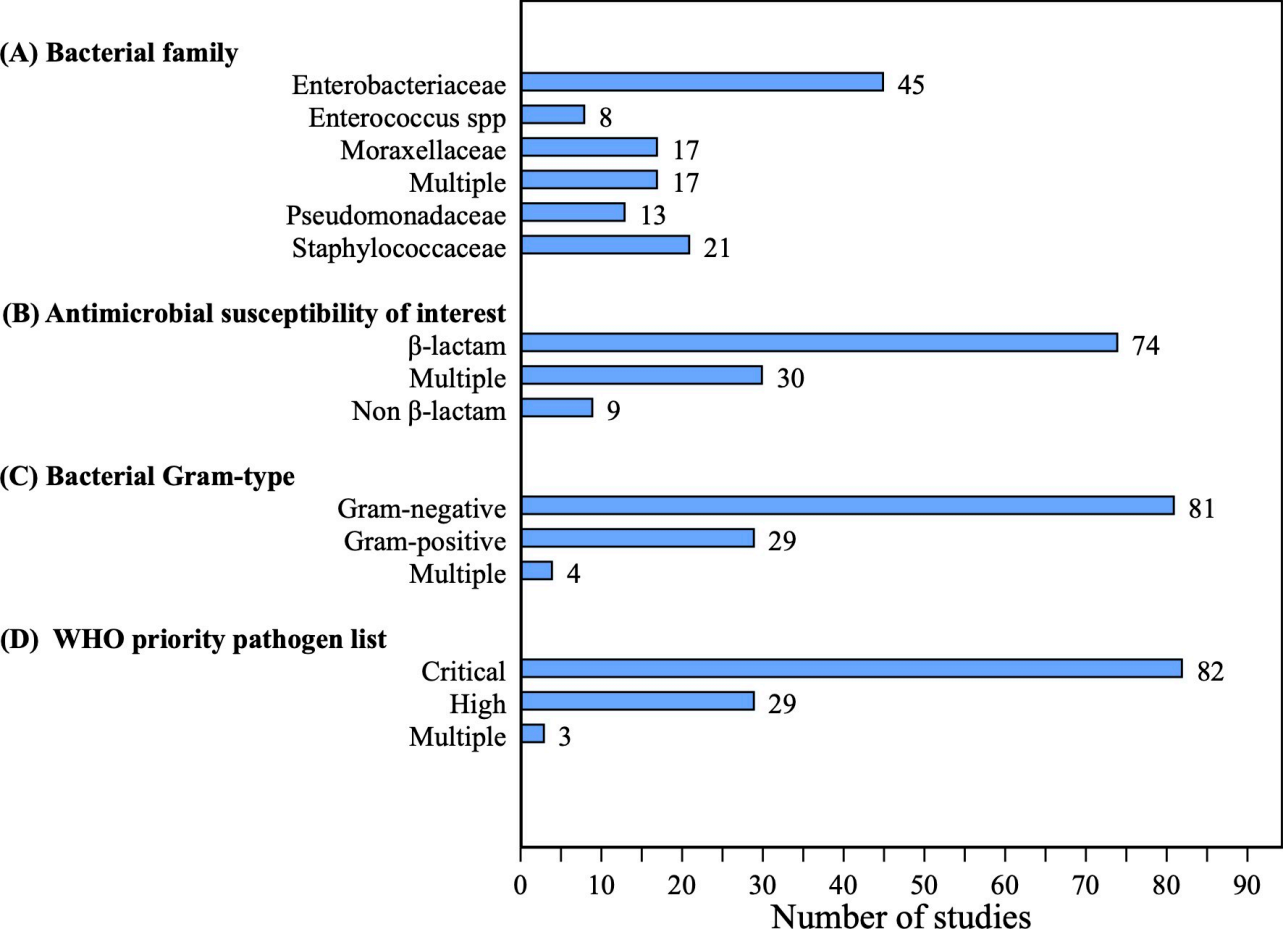
Number of included studies categorised by microbiological features †. (A) Number of included studies by bacterial family (B) Number of included studies by antimicrobial susceptibility of interest (C) Number of included studies by bacterial Gram-type (D) Number of included studies by WHO priority pathogen list. Enterobacteriaceae included *Escherichia coli* and *Klebsiella pneumoniae*. Enterococcus spp. stands for Enterococcus species pluralis (multiple species), which included *Enterococcus faecalis* and *faecium*. The multiple categories stand for either multiple bacteria or antibiotics analysed throughout our selected studies, which were not reported disaggregated by bacterial family, biological strain, gram type, or WHO priority pathogen list. † Studies could include more than 1 subcategory per biological feature (i.e., a study might report Enterobacteriaceae and Pseudomonadaceae species separately in their analyses, or altogether, in which case it was classified as “Multiple,” meaning no clear distinction between subcategories). Categories might not be exclusive per study. WHO, World Health Organization.

Of the 109 articles, 100 (91.7%; 100/109) studies reported the impacts of ARB BSIs on mortality, 42 on hospital LOS, but only 18 displayed the average LOS with its standard deviation (16.5%; 18/109) and 52 (47.7%; 52/109) reported on ICU admission (Table 1). Studies were primarily conducted in China (44.9%; 49/109, *N* = 12,092 patients), Brazil (11.9%; 13/109, *N* = 1,559 patients), and Turkey (8.3%; 9/109, *N* = 2,190 patients) (Fig 2). Most studies collected data from the Western Pacific region according to the WHO classification (46.8%; 51/109) and 88% (96/109) were from upper-middle-income countries (S1 Text, section 2). The majority of the studies reported on gram-negative bacteria, mainly Enterobacteriaceae (41.3%; 45/109), Moraxellaceae or *Acinetobacter baumanii* (15.6%; 17/109), and *Pseudomonas aeruginosa* (11.9%, 13/109) (Fig 3). The main gram-positive pathogens reported were *Staphylococcus aureus* (19.3%; 21/109) and *Enterococcus* spp. (7.3%; 8/109); 75.2% (82/109) of the pathogens reported were classified as a critical priority following the WHO criteria (Fig 3). β-lactam antibiotics were among the most tested antibiotic class within the studies (67.9%; 74/109), 71.6% (53/74) of which were carbapenems or cephalosporins (Fig 3). The total number of patients and most prevalent features per country’s studies are reported in Table E in S1 Text. Table F in S1 Text presents the weighted unadjusted differences for sociodemographic and health variables among ARB and ASB groups. We found no statistically significant difference between ARB and ASB groups for most of these variables (χ^2^ test *p* > 0.05). S1 Text section 2 describes the distribution of our studies by WHO region, World Bank income group, year, and outcomes densities per ARB/ASB group.

**Table 1:** Details of all studies included in the systematic literature review (*N* = 109).

ID^⁂^	Author/year	Country setting	Bacterium family	Group comparison		Group *N* of obs.		Mortality, *n* (%)		LOS (mean)		ICU admission, *n* (%)	
				Case	Control	Case	Control	Case	Control	Case	Control	Case	Control
**1**	Abhilash, 2010 [46]	India	Enterobacteriaceae	ESBL+	ESBL-	96	35	24(25)	9(26)				
**2**	Abolghasemi, 2018 [47]	Iran	Moraxellaceae	XDR	non-XDR	16	14	13(81)	1(7)			8(50)	0(0)
**3**	Akhtar, 2016 [48]	Pakistan	Enterococcus spp.	VRE	VSE	46	65	29(63)	28(43)	28.5	13.2	23(50)	9(14)
**4**	Anggraini, 2022 [49]	Indonesia	Moraxellaceae	CRAB	CSAB	72	72	41(57)	35(49)	17	13	60(83)	49(68)
**5**	Anunnatsiri, 2011 [50]	Thailand	Moraxellaceae	MDR	non-MDR	24	25	22(92)	12(48)	21.5	14	9(38)	3(12)
**6**	Arias-Ortiz, 2016 [51]	Colombia	Staphylococcaceae	MRSA	MSSA	186	186					105(56)	89(48)
**7**	Atmaca, 2014 [52]	Turkey	Staphylococcaceae	MRSA	MSSA	99	99			70.84	14	25(25)	6(6)
**8**	Barrero, 2014 [53]	Colombia	Staphylococcaceae	MRSA	MSSA	102	102	62(61)	46(45)	30	21	64(63)	54(53)
**9.1**	Braga, 2013 [54]	Brazil	Staphylococcacea	MRSA	MSSA	12	44	7(58)	25(57)				
**9.2**	Braga, 2013 [54]	Brazil	Pseudomonadaceae	CRPA	CSPA	14	42	13(93)	19(45)				
**9.3**	Braga, 2013 [54]	Brazil	Enterobacteriaceae	CREN	CSEN	3	53	2(67)	30(57)				
**9.4**	Braga, 2013 [54]	Brazil	Enterobacteriaceae	CERKP	CESKP	5	51	4(80)	28(55)				
**10**	Castillo 2012 [55]	Colombia	Staphylococcaceae	MRSA	MSSA	186	186	62(33)	48(26)			105(56)	90(48)
**11**	Carena, 2020 [56]	Argentina	Multiple	MDR	non-MDR	168	226	58(35)	36(16)			54(32)	43(19)
**12**	Cetin, 2021 [57]	Turkey	Multiple gram-negative	CRGN	CSGN	54	157	29(54)	31(20)	45	20		
**13**	Chang, 2020 [58]	China	Enterobacteriaceae	CRKP	CSKP	46	239	27(59)	37(15)			26(57)	33(14)
**14**	Chen, 2022 [59]	China	Enterobacteriaceae	CRKP	CSKP	29	223	14(48)	13(6)			21(72)	38(17)
**15**	Chen, 2012 [60]	China	Staphylococcaceae	MRSA	MSSA	75	43	25(33)	8(19)	55	38.7		
**16**	Chusri 2019 [61]	Thailand	Moraxellaceae	CRAB	CSAB	31	11	20(65)	2(18)	89	57	20(65)	6(55)
**17**	Conterno 1998 [62]	Brazil	Staphylococcaceae	MRSA	MSSA	90	46	44(49)	9(20)			54(60)	13(28)
**18**	Dantas 2017 [63]	Brazil	Pseudomonadaceae	MDR	non-MDR	67	90					39(58)	35(39)
**19**	Deodhar 2015 [64]	India	Staphylococcaceae	MRSA	MSSA	40	61	8(20)	13(21)				
**20**	De-Oliveira 2002 [65]	Brazil	Staphylococcaceae	MRSA	MSSA	159	92	73(46)	19(21)				
**21**	Deris, 2011 [66]	Malaysia	Moraxellaceae	IRAB	ISAB	15	41	6(40)	9(22)	32.3	32.8	11(73)	20(49)
**22**	Dramowski, 2022 [67]	South Africa	Enterobacteriaceae	CEREN	CESEN	62	115	27(44)	33(29)	10.5	9		
**23**	Durdu, 2016 [68]	Turkey	Enterobacteriaceae	CRKP	CRSKP	46	63	23(50)	23(37)				
**24**	Ergönül, 2016 [69]	Turkey	Multiple	CRGN	CSGN	379	452	236(62)	135(30)				
**25**	Ferreira, 2018 [70]	Brazil	Multiple	MDR	non-MDR	25	37	10(40)	3(8)				
**26**	Fu, 2015 [71]	China	Moraxellaceae	XDR	non-XDR	39	86	31(79)	38(44)	36.7	36.1	31(79)	45(52)
**27**	Furtado, 2006 [72]	Brazil	Enterococcus spp.	VRE	VSE	34	55			57.7	29	13(38)	18(33)
**28**	Garnica, 2009 [73]	Brazil	Multiple	MDR	non-MDR	10	44	4(40)	4(9)				
**29**	Gaytán, 2006 [74]	Mexico	Enterobacteriaceae	CiREC	CiSEC	26	24	4(15)	3(13)				
**30**	Ghafur, 2014 [75]	India	Multiple	MDR	non-MDR	44	97	28(64)	37(38)				
**31.1**	Goda, 2022 [76]	India	Multiple	MDR	non-MDR	8	22	1(13)	8(36)				
**31.2**	Goda, 2022 [76]	India	Multiple	XDR	non-XDR	20	10	8(40)	1(10)				
**32**	González, 2014 [77]	Colombia	Pseudomonadaceae	MDR	non-MDR	92	141						
**33**	Guo, 2016 [78]	China	Moraxellaceae	MDR	non-MDR	64	23	38(59)	1(4)			51(80)	5(22)
**34**	Hincapié, 2020 [45]	Colombia	Staphylococcaceae	MRSA	MSSA	292	909	219(75)	71(8)			239(82)	84(9)
**35.1**	Islas-Muñoz, 2018 [79]	Mexico	Enterobacteriaceae	ESBL+	ESBL-	123	148	37(30)	35(24)				
**35.2**	Islas-Muñoz, 2018 [79]	Mexico	Multiple gram-negative	MDR	non-MDR	9	34	6(67)	5(15)				
**35.3**	Islas-Muñoz, 2018 [79]	Mexico	Multiple gram-positive	MDR	non-MDR	6	43	2(33)	4(9)				
**36**	Jafari, 2020 [80]	Iran	Enterococcus spp.	VRE	VSE	52	21	30(57)	6(29)	36.6	22.32	30(58)	5(24)
**37**	Jamulitrat, 2009 [81]	Thailand	Moraxellaceae	IRAB	ISAB	67	131	35(52)	26(20)	37	27		
**38**	Kalam, 2014 [82]	Pakistan	Multiple	MDR	non-MDR	117	126	54(46)	34(27)			32(27)	36(29)
**39**	Li, 2019 [83]	China	Enterobacteriaceae	CRKP	CSKP	19	21	8(42)	2(10)	21	18	11(58)	5(24)
**40**	Li, 2017 [84]	China	Enterobacteriaceae	MDR	non-MDR	76	28	23(30)	3(11)				
**41**	Li, 2018 [85]	China	Pseudomonadaceae	CRPA	CSPA	63	63	17(27)	8(13)	30	21		
**42**	Li, 2017 [86]	China	Enterobacteriaceae	CREN	CSEN	26	122	17(65)	21(17)	25.4	21	20(77)	10(8)
**43**	Li, 2020 [87]	China	Enterobacteriaceae	CRKP	CSKP	164	328	72(44)	49(15)	31	19	116(71)	58(18)
**44**	Liang, 2021	China	Enterobacteriaceae	CRKP	CSKP	56	47	22(39)	9(19)	28.5	28	20(36)	13(28)
**45.1**	Lim, 2016 [88]	Thailand	Staphylococcaceae	MDR	non-MDR	2017		299*					
**45.2**	Lim, 2016 [88]	Thailand	Enterobacteriaceae	MDR	non-MDR	144		20*					
**45.3**	Lim, 2016 [88]	Thailand	Enterobacteriaceae	MDR	non-MDR	288		7*					
**45.4**	Lim, 2016 [88]	Thailand	Pseudomonadaceae	MDR	non-MDR	94		4*					
**45.5**	Lim, 2016 [88]	Thailand	Moraxellaceae	MDR	non-MDR	864		351*					
**46**	Lima, 2020 [89]	Brazil	Multiple	CR	CS	60	30	30(50)	12(40)	26.5	15		
**47**	Lipari, 2020 [90]	Argentina	Enterobacteriaceae	CREN	CSEN	42	42	22(52)	7(17)			32(76)	12(29)
**48**	Liu, 2019 [91]	China	Enterobacteriaceae	CRKP	CSKP	20	69	11(55)	11(16)				
**49**	Liu, 2015 [92]	China	Moraxellaceae	MDR	non-MDR	182	59	50(27)	3(5)			109(60)	7(12)
**50**	Liu, 2019 [93]	China	Enterobacteriaceae	CRKP	CSKP	70	28	30(43)	12(43)				
**51**	Liu, 2020 [94]	China	Moraxellaceae	CRAB	CSAB	229	88	60(26)	4(5)			129(56)	26(30)
**52**	Loftus, 2022 [95]	Fiji	Enterobacteriaceae	CREN	CSEN	66	96	20(30)	16(17)	13	8		
**53.1**	Lopez-Luis, 2020 [96]	Mexico	Enterococcus spp	VRE	VSE	107	85	34(32)	11(13)			41(38)	11(13)
**53.2**	Lopez-Luis, 2020 [96]	Mexico	Enterococcus spp	ARE	ASE	18	129	5(28)	23(18)			4(22)	22(17)
**54**	Ma, 2017 [97]	China	Enterobacteriaceae	ESBL+	ESBL-	70	43	15(21)	6(14)				
**55**	Marra, 2006 [98]	Brazil	Enterobacteriaceae	ESBL+	ESBL-	56	52	18(32)	8(15)			31(55)	18(35)
**56**	Meneküe 2019 [99]	Turkey	Enterobacteriaceae	CRKP	CSKP	111	99	77(69)	44(44)				
**57**	Metan, 2009 [100]	Turkey	Moraxellaceae	CRAB	CSAB	54	46	41(76)	22(48)				
**58**	Moghnieh, 2015 [101]	Lebanon	Multiple	MDR	non-MDR	7	68	4(57)	3(4)				
**59**	Moreira, 1998 [102]	Brazil	Staphylococcaceae	ORSA	OSSA	71	71	40(56)	8(11)	32.7	29.7		
**60**	Najmi, 2019 [103]	India	Enterobacteriaceae	ESBL+	ESBL-	101	81	29(29)	19(24)				
**61**	Niu, 2018 [104]	China	Moraxellaceae	CRAB	CSAB	242	51	84(35)	2(4)				
**62.1**	Palavutitotai, 2018 [105]	Thailand	Pseudomonadaceae	MDR	non-MDR	32	167	12(38)	38(23)				
**62.2**	Palavutitotai, 2018 [105]	Thailand	Pseudomonadaceae	XDR	non-XDR	56	199	23(41)	50(25)	53.5	45.5	8(14)	42(21)
**63**	Porto, 2013 [106]	Brazil	Staphylococcaceae	MRSA	MSSA	61	169	44(71)	36(21)	43.2	20.5		
**64**	Rao 2020 [107]	India	Enterococcus spp.	VRE	VSE	73	100	27(37)	33(33)	34.47	26.25	21(29)	41(41)
**65**	Seboxa, 2015 [108]	Ethiopia	Enterobacteriaceae	CEREC	CESEC	10	6	10(100)	0(0)				
**66**	Serefhanoglu 2009 [109]	Turkey	Enterobacteriaceae	MDR	non-MDR	30	64	7(23)	12(19)				
**67**	Shi, 2009 [110]	China	Multiple	MDR	non-MDR	70	82	27(39)	12(15)				
**68.1**	Shi, 2022 [111]	China	Multiple	CRGN	CSGN	65	953	29(45)	79(8)				
**68.2**	Shi, 2022 [111]	China	Multiple	ESBL+	ESBL-	347	671	33(10)	75(11)				
**68.3**	Shi, 2022 [111]	China	Multiple	MDR	non-MDR	412	606	56(14)	52(9)				
**69.1**	Sirijatuphat, 2018 [112]	Thailand	Enterobacteriaceae	CREC	CSEC	106	100	23(22)	18(18)				
**69.2**	Sirijatuphat, 2018 [112]	Thailand	Enterobacteriaceae	CRKP	CSKP	45	65	23(51)	22(34)				
**69.3**	Sirijatuphat, 2018 [112]	Thailand	Pseudomonadaceae	CRPA	CSPA	21	47	10(48)	19(40)				
**69.4**	Sirijatuphat, 2018 [112]	Thailand	Moraxellaceae	CRAB	CSAB	57	24	38(67)	3(13)				
**69.5**	Sirijatuphat, 2018 [112]	Thailand	Enterobacteriaceae	FRS	FSS	2	2	0(0)	1(50)				
**69.6**	Sirijatuphat, 2018 [112]	Thailand	Staphylococcaceae	MRSA	MSSA	16	47	9(56)	13(28)				
**69.7**	Sirijatuphat, 2018 [112]	Thailand	Enterococcus spp.	VRE	VSE	9	20	6(67)	12(60)				
**70**	Soares, 2022 [113] ^⍴^	Brazil	Enterobacteriaceae	CRKP	CSKP	28	79						
**71**	Steinhaus, 2018 [114] ^a^	South Africa	Staphylococcaceae	MRSA	MSSA	23	75						
**72**	Stewardson, 2019 [115]	Multiple LMICs ☨	Enterobacteriaceae	CREN	CSEN	123	174	43(35)	35(20)	3.7*		54(44)	51(29)
**73.1**	Stoma, 2016 [116]	Belarus	Multiple	CR	CS	23	112	17(74)	25(22)				
**73.2**	Stoma, 2016 [116]	Belarus	Enterobacteriaceae	ESBL+	ESBL-	24	111	6(25)	36(32)				
**73.3**	Stoma, 2016 [116]	Belarus	Staphylococcaceae	MRSA	MSSA	15	120	4(27)	38(32)				
**74**	Tang, 2021 [117]	China	Multiple	CRGN	CSGN	78	757	27(35)	79(10)				
**75**	Tian, 2016 [118]	China	Enterobacteriaceae	CRKP	CSKP	33	81	14(42)	16(20)	50	24		
**76**	Topeli, 2000 [119]	Turkey	Staphylococcaceae	MRSA	MSSA	46	55	27(59)	17(31)	50.3	32.7	20(43)	13(24)
**77**	Traverso, 2010 [120]	Argentina	Staphylococcaceae	MRSA	MSSA	17	22	12(71)	8(36)				
**78**	Tu, 2018 [121]	China	Enterobacteriaceae	MDR	non-MDR	55	145	9(16)	19(13)			16(29)	18(12)
**79**	Tuon, 2012 [122]	Brazil	Pseudomonadaceae	CRPA	CSPA	29	48	13(45)	26(54)	43	43.1	24(83)	25(52)
**80**	Valderrama, 2016 [123]	Colombia	Pseudomonadaceae	CRPA	CSPA	42	126	24(57)	45(36)	26	16	26(62)	73(58)
**81**	Wang, 2016 [124]	China	Enterobacteriaceae	CREN	CSEN	94	93	33(35)	11(12)	40	26	49(52)	33(35)
**82**	Wang, 2018 [125]	China	Enterobacteriaceae	CRKP	CSKP	48	48	23(48)	2(4)	84	33	25(52)	3(6)
**83**	Wei, 2020 [126]	China	Pseudomonadaceae	CRPA	CSPA	23	58	14(61)	10(17)				
**84.1**	Wu, 2021 [127]	China	Enterobacteriaceae	CRKP	CSKP	24	55	10(42)	12(22)				
**84.2**	Wu, 2021 [127]	China	Enterobacteriaceae	ESBL+	ESBL-	24	55	9(38)	15(27)				
**84.3**	Wu, 2021 [127]	China	Enterobacteriaceae	MDR	non-MDR	36	43	12(33)	12(28)				
**85**	Xiao, 2018 [128]	China	Enterobacteriaceae	CRKP	CSKP	135	293	52(39)	26(9)				
**86**	Xiao, 2020 [129]	China	Enterobacteriaceae	CRKP	CSKP	104	267	58(56)	37(14)	35	23		
**87**	Xie, 2018 [130]	China	Multiple	MDR	non-MDR	186	322	59(32)	72(22)			42(23)	40(12)
**88**	Xu, 2015 [131]	China	Enterococcus spp.	VRE	VSE	31	54					21(68)	24(44)
**89**	Yang, 2018 [132]	China	Moraxellaceae	CRAB	CSAB	84	34	23(27)	2(6)			55(65)	6(18)
**90**	Yang, 2021 [133]	China	Pseudomonadaceae	CRPA	CSPA	65	155	17(26)	29(19)	38	24	34(52)	46(30)
**91**	Ye, 2014 [134]	China	Multiple	rESKAPE	sESKAPE	39	32	22(56)	12(38)				
**92**	Yilmaz, 2016 [135]	Turkey	Staphylococcaceae	MRSA	MSSA	100	145	22(22)	7(5)				
**93**	Yuan, 2020 [136]	China	Enterobacteriaceae	CRKP	CSKP	98	141	7(7)	2(1)	55	51	82(84)	44(31)
**94**	Zhang, 2020 [137]	China	Enterobacteriaceae	CRKP	CSKP	108	388	41(38)	34(9)	24.5	26	85(79)	155(40)
**95**	Zhang, 2019 [138]	China	Enterobacteriaceae	ESBL+	ESBL-	160	164	39(24)	32(20)				
**96**	Zhang, 2017 [139]	China	Enterobacteriaceae	CEREC	CESEC	51	197	13(25)	24(12)	29.88	30.98	4(8)	23(12)
**97**	Zhang, 2017 [140]	China	Enterococcus spp.	VRE	VSE	7	217	2(29)	52(24)				
**98**	Zhang, 2020 [141]	China	Pseudomonadaceae	CRPA	CSPA	40	29	30(75)	12(41)				
**99**	Zhao, 2022 [142]	China	Enterobacteriaceae	ESBL+	ESBL-	159	205	29(18)	24(12)				
**100.1**	Zhao, 2020 [143]	China	Pseudomonadaceae	CRPA	CSPA	55	238	11(20)	14(6)	29	26		
**100.2**	Zhao, 2020 [143]	China	Pseudomonadaceae	MDR	non-MDR	38	255	11(29)	14(5)	27	26		
**101**	Zheng, 2018 [144]	China	Enterobacteriaceae	CRKP	CSKP	59	230	32(54)	45(20)			28(47)	47(20)
**102**	Zheng, 2017 [145]	China	Enterobacteriaceae	CRKP	CSKP	31	17	19(61)	8(47)	31.74	21.47		
**103**	Zhou, 2019 [146]	China	Moraxellaceae	MDR	non-MDR	274	64	161(59)	8(13)	29	22.5	184(67)	12(19)
**104**	Zhu, 2016 [147]	China	Staphylococcaceae	MRSA	MSSA	22	42	6(27)	6(14)	25.7	15.3		
**105**	Zhu, 2021 [148]	China	Enterobacteriaceae	CREN	CSEN	152	727	87(57)	133(18)	35	20	98(64)	135(19)
**106**	Zlatian, 2018 [149]	Romania	Staphylococcaceae	MRSA	MSSA	23	40					14(61)	19(48)
**107**	Zou, 2020 [150]	China	Enterobacteriaceae	CREC	CSEC	31	367	17(55)	39(11)			20(65)	61(17)
**108**	Zhang, 2018 [151]	China	Enterobacteriaceae	MDR	non-MDR	77	33	10(13)	10(30)				
**109**	Zhang, 2017 [152]	China	Moraxellaceae	CRAB	CSAB	49	29	40(82)	6(21)			10(20)	12(41)

Full information can be found in S1 Data.

*Reported as excess mortality or length of stay. Empty cells did not reported values for the outcomes.

^a^This study reported unadjusted and adjusted ORs rather than raw values for outcome variables.

^**⁂**^Studies ID comprised the main articles and articles’ sub-studies if information on the outcomes by comparison group was reported separately for more than 1 bacterium or resistance-type according to their specific populations.

☨LMICs included in the study were India, Egypt, Nigeria, Colombia, Ghana, Pakistan, Lebanon, Vietnam, and Bangladesh.

^⍴^Odds ratios were reported only.

MRSA, methicillin-resistant *Staphylococcus aureus*; MSSA, methicillin-sensitive *Staphylococcus aureus*; MDR, multi-drug resistance; CRKP, carbapenem-resistant *Klebsiella pneumoniae*; CSKP, carbapenem-sensitive *Klebsiella pneumoniae*; CRPA, carbapenem-resistant *Pseudomonas aeruginosa*; CSPA, carbapenem-sensitive *Pseudomonas aeruginosa*; CRAB, carbapenem-resistant *Acinetobacter baumannii*; CSAB, carbapenem-sensitive *Acinetobacter baumannii*; CREC, carbapenem-resistant *Escherichia coli*; CSEC, carbapenem-sensitive *Escherichia coli*; IRAB, imipenem-resistant *Acinetobacter baumannii*; ISAB, imipenem-sensitive *Acinetobacter baumannii*; ESBL, extended-spectrum β-lactamases; VRE, Vancomycin-resistant *Enterococcus spp*; VRE, Vancomycin-sensitive *Enterococcus* spp.; CERKP, Cephalosporins-resistant *Klebsiella pneumoniae;* CESKP, Cephalosporins-sensitive *Klebsiella pneumoniae; CiREC*, Ciprofloxacin-resistant *Escherichia coli; CiSEC*, Ciprofloxacin-sensitive *Escherichia coli;* CRGN, Carbapenem-resistant gram-negative bacteria; CSGN, Carbapenem sensitive gram-negative bacteria; CR, Carbapenem resistance; CS, Carbapenem sensitive; CREN, Carbapenem-resistant *Enterobacteriaceae;* CSEN, Carbapenem-sensitive *Enterobacteriaceae;* ARE, Ampicillin-resistant *Enterococcus* spp.; ASE, Ampicillin-sensitive *Enterococcus* spp.; ORSA, Oxacillin-resistant *Staphylococcus aureus;* OSSA, Oxacillin-sensitive *Staphylococcus aureus;* CEREC, Cephalosporins-resistant *Escherichia coli;* CESEC, Cephalosporins-sensitive *Escherichia coli; FRS*, Fluoroquinolone-resistant *Salmonella* spp.*; FSS*, Fluoroquinolone-sensitive *Salmonella* spp.; XDR, Extensive drug-resistance. rESKAPE: Vancomycin-resistant *E*. *faecium*, methicillin-resistant *S*. *aureus* (MRSA), extended-spectrum β-lactamase (ESBL)-producing *K*. *pneumoniae*, carbapenem-resistant *A*. *baumannii*, carbapenem- and quinolone-resistant P. aeruginosa, and de-repressed chromosomal β-lactam and ESBL-producing Enterobacter species. sESKAPE: sensitive ESKAPE; ICU: intensive care unit; LOS: length of stay.

### Quantitative results

#### The odds of health outcomes

The crude OR for mortality of ARB versus ASB BSIs was 1.58 (95% CI [1.35 to 1.80], *p* < 0.001); we obtained similar values for gram-negative or WHO critical priority pathogens (OR 1.59, 95% CI [1.34 to 1.83], *p* < 0.001) (Table 2, section I). The highest OR of crude mortality for resistant pathogens was for carbapenem-resistant Enterobacteriaceae (OR 1.97, 95% CI [1.37 to 2.56], *p* < 0.001) (Table 3). The impact seemed to be lower among gram-positive bacteria, with an OR of 1.51 (95% CI [0.76 to 2.26], *p* 0.13) for MRSA and an OR of 1.31 (95% CI [1.01 to 1.60], *p* 0.02) for vancomycin-resistant Enterococcus species. Compared to ASB BSIs, ARB BSIs in upper-middle-income countries (OR 1.64, 95% CI [1.36 to 1.92], *p* < 0.001) from Europe and Western Pacific WHO regions (OR 1.79, 95% CI [1.49 to 2.11], *p* < 0.001, and OR 1.66, 95% CI [1.18 to 2.14], *p* < 0.001, respectively) had the highest excess mortality (Table G in S1 Text). Among priority pathogens defined by the WHO, crude excess mortality from carbapenem-resistant *K*. *pneumoniae* was substantially higher than for other pathogens (OR 1.79, 95% CI [1.15 to 2.43], *p* 0.002; Table 3), compared to sensitive counterparts. Among studies reporting both adjusted and unadjusted ORs for mortality (*N* = 12), we found 1.35 and 1.57 times higher unadjusted and adjusted mortality figures, respectively, for patients having BSIs caused by ARB versus ASB (Fig AJ in S1 Text). We found lower mortality estimates among studies reporting adjusted ORs for gram-negative ARB BSIs (OR = 1.88), specifically for Enterobacteriaceae and Moraxellaceae species (OR 1.91 and OR 1.73, respectively), compared to the same unadjusted estimates (OR 2.95 and OR 3.28, respectively) (Figs AK and AL in S1 Text). However, and surprisingly for the most part, adjusted ORs for mortality among ARB versus ASB BSI patients reflected greater odds compared to unadjusted ORs. This is explained by a single, highly influential study [45] among unadjusted estimates displaying a smaller OR (although confidence intervals overlap between unadjusted and adjusted ORs, and study’s weight is lower among adjusted estimates).

Overall, the crude odds of ICU admission were 1.96 times higher for ARB compared to ASB BSIs (95% CI [1.56 to 2.47], *p* < 0.001) (Table 2, section II). Patients with WHO critical priority pathogens resistant to antibiotics were twice as likely to be admitted to ICU (OR 2.02, 95% CI [1.62 to 2.52], *p* < 0.001), with the highest observed ratio for gram-negative BSIs caused by antibiotic-resistant Enterobacteriaceae (OR 2.59, 95% CI [1.95 to 3.45], *p* < 0.001). Carbapenem-resistant Enterobacteriaceae in general (OR 2.66, 95% CI [1.98 to 3.57], *p* < 0.001), and specifically *Escherichia coli* (OR 3.88, 95% CI [2.74 to 5.49], *p* < 0.001), accounted for the highest figures (Table 3). Among gram-positive bacteria, Methicillin-resistant *Staphylococcus aureus* had an OR of 1.91 for ICU admission rate (95% CI [0.86 to 4.25], *p* 0.11), and vancomycin-resistant *Enterococcus faecium/faecalis* had an OR of 1.48 (95% CI [0.87 to 2.54], *p* 0.15) (Table 3). The Western Pacific region had the highest increase in ICU odds (OR 2.42, 95% CI [1.88 to 3.12], *p* < 0.001), followed by the Americas (OR 1.77, 95% CI [1.08 to 2.89], *p* 0.02), whereas the Southeast Asia region had the lowest odds of ICU admission of ARB BSIs compared to ASB BSIs (Table G in S1 Text).

The crude SMD for LOS was 0.49 (95% CI [0.20 to 0.78], *p* < 0.001; Table 2, section III). In other words, the curve representing the distribution of LOS times was shifted to the right by 0.49 standard deviations for the ARB BSIs group (i.e., LOS is approximately 7 days longer for the ARB group; derived from multiplying SMD by LOS’s standard deviation among all patients [0.49*13.91]). The SMD was higher for resistant pathogens classified as WHO high-priority pathogens (or gram-positive, SMD 0.71, 95% CI [0.03 to 1.39], *p* 0.04) compared with WHO critical priority pathogens (or gram-negative, SMD 0.37, 95% CI [0.17 to 0.57], *p* 0.13). Studies reporting MRSA accounted for the greatest excess LOS estimated (SMD 0.82; Table 3), compared to methicillin-sensitive *S*. *aureus*. The highest excess LOS was observed in studies from Turkey (SMD 1.29). Studies from Europe (SMD 1.29) and Brazil (SMD 0.43) contributed substantially to the greater LOS in ARB BSI patients (Table G in S1 Text).

Full details on the meta-analysis main and subgroup results, including their respective forest plots, can be found in S1 Text, section 3.

Tables W and X in S1 Text show the results of the univariate and multivariable meta-regressions for mortality and ICU admission, respectively. Among the variables selected from the univariate analyses, our multivariable meta-regression showed that patients with resistant Moraxellaceae BSIs and hypertension had higher mortality odds when ARB versus ASB BSI patients were compared (OR 1.67, 95% CI [1.18 to 2.36], *p* 0.004; OR 1.13, 95% CI [1.00 to 1.28], *p* 0.035, respectively). Yet, countries from the Southeast Asia WHO region displayed lower mortality odds (OR 0.62, 95% CI [0.46 to 0.85], *p* 0.004). For the ICU admission multivariable meta-regression, we found a weak negative association between BSIs originating as a secondary infection from the urinary tract and the odds of mortality between patients having ARB and ASB BSIs (OR 0.72, 95% CI [0.51 to 1.02], *p* 0.06).

**Table 2:** Main results of the meta-analysis comparing outcomes between patients with drug-resistant and drug-sensitive infections, overall and per bacterial family and WHO priority list classification (*N* = 109 studies^‡^).

Outcome variables	OR/SMD	95% CI	*P*-value	tau^2^	*N* of patients	*N* of studies
**I. Mortality** ^**a**^	**OR**					
Overall	1.58	1.35, 1.80	<0.001	0.39	19,597	93
WHO classification						
Critical priority pathogens (gram-negative)	1.59	1.34, 1.83	<0.001	0.36	15,206	72
High-priority pathogens (gram-positive)	1.47	0.94, 2.00	0.045	0.48	4,472	22
Bacterial family						
Enterobacteriaceae	1.49	1.09, 1.90	0.005	0.61	8,646	40
Enterococcus spp.	1.32	1.02, 1.61	0.017	0.00	949	6
Moraxellaceae	1.59	1.16, 2.02	<0.001	0.12	2,297	16
Pseudomonadaceae	1.37	1.04, 1.69	0.011	0.10	1,353	10
Staphylococcaceae	1.52	0.76, 2.28	0.135	0.80	3,566	17
**II. ICU admission** ^**b**^	**OR**					
Overall	1.96	1.56, 2.47	<0.001	0.33	12,005	52
WHO classification						
Critical priority pathogens (gram-negative)	2.02	1.62, 2.52	<0.001	0.21	8,488	38
High-priority pathogens (gram-positive)	1.82	0.99, 3.37	0.055	0.68	3,517	14
Bacterial family						
Enterobacteriaceae	2.59	1.95, 3.45	<0.001	0.16	4,841	18
Enterococcus spp.	1.48	0.90, 2.41	0.119	0.27	870	6
Moraxellaceae	1.57	1.02, 2.41	0.039	0.20	1,625	12
Pseudomonadaceae	1.37	1.05, 1.77	0.018	0.05	877	5
Staphylococcaceae	1.91	0.86, 4.25	0.112	0.82	2,647	8
**III. LOS** ^**c**^	**SMD**					
Overall	0.49	0.20, 0.78	<0.001	0.27	3,185	18
WHO classification						
Critical priority pathogens (gram-negative)	0.37	0.17, 0.57	<0.001	0.06	2,097	11
High-priority pathogens (gram-positive)	0.71	0.03, 1.39	0.040	0.66	1,088	7
Bacterial family						
Enterobacteriaceae	0.43	0.14, 0.73	0.004	0.06	1,175	5
Enterococcus spp.	0.25	−0.05, 0.55	0.102	-	173	1
Moraxellaceae	0.16	−0.06, 0.38	0.155	0.00	379	3
Pseudomonadaceae	0.14	−0.11, 0.39	0.276	0.00	332	2
Staphylococcaceae	0.82	0.01, 1.63	0.047	0.78	915	6

WHO, World Health Organization. Where the numbers of studies seem inconsistent, this is attributable to several studies reporting on multiple categories (WHO) or combined pathogens simultaneously. ICU stands for intensive care unit. Fully disaggregated results, including their respective forest plots, are shown in S1 Text, section 3. OR, odds ratio; SMD, standardised mean difference; CI, Confidence interval; N, number.

^**a**^From the total 109 studies included in the systematic review, 9 were excluded as they had missing data; one study was excluded as it only reported excess deaths for ARB BSIs at the country level [88]; and, 6 studies evaluated mortality by comparison group but reported different bacteria for the sample of individuals and therefore were excluded from the overall analysis but had sufficient information to be retained for the subgroup analyses.

^**b**^One study [96] reported data on demographics and ARB BSI for 2 different pathogens and with non-duplicate episodes, which were included as separate sub-studies.

^**c**^The number of studies/sub-studies differs from Table F in S1 Text because some studies did not report the standard deviation of LOS, so the SMD could not be computed.

^‡^One study was excluded from the *N* = 109 initial sample because it only reported excess mortality. *P*-values (p) were reported using a two-sided z-test (α = 5%) for the log-transformed mortality and ICU admission ratios and LOS’s SMD.

ARB, antibiotic-resistant bacteria; BSI, bloodstream infection; LOS, length of hospital stay.

**Table 3:** Meta-analysis subgroup results by the most common antibiotic-resistant microbial strains according to the WHO global priority list of antibiotic-resistant bacteria.

Outcome	Most common antibiotic-resistant microbial strains*	OR/SMD	95% CI	*P*-value	*N* of studies
**I. Mortality**		**OR**			
	CRAB	1.46	0.80, 2.11	0.120	10
	CREN	1.97	1.37, 2.56	<0.001	26
	CREC	1.54	0.00, 6.37	0.857	2
	CRKP	1.79	1.15, 2.43	0.002	19
	CRPA	1.36	0.89, 1.82	0.088	9
	MRSA	1.51	0.76, 2.26	0.132	16
	VRE	1.31	1.01, 1.60	0.021	6
**II. ICU admission**		**OR**			
	CRAB	1.36	0.85, 2.16	0.198	6
	CREN	2.66	1.98, 3.57	<0.001	15
	CREC‡	3.88	2.74, 5.49	<0.001	1
	CRKP	2.60	1.81, 3.75	<0.001	9
	CRPA	1.39	1.02, 1.90	<0.001	3
	MRSA	1.91	0.86, 4.25	0.112	8
	VRE	1.48	0.87, 2.54	0.152	6
**III. LOS**		**SMD**			
	CRAB	0.22	−0.04, 0.49	0.104	2
	CREN	0.53	0.39, 0.67	<0.001	4
	CREC^‡^	-	-	-	-
	CRKP	0.56	0.41, 0.71	<0.001	3
	CRPA^‡^	0.00	−0.46, 0.46	1.000	1
	MRSA	0.82	0.00, 1.63	0.048	6
	VRE^‡^	0.25	−0.05, 0.55	0.102	1

*All comparisons and ORs/SMD computations were made concerning their sensitive-specific counterpart. CRAB, Carbapenem-resistant *Acinetobacter baumanii*; CREN, Carbapenem-resistant *Enterobacteriaceae*; CREC, Carbapenem-resistant *Escherichia coli*; CRKP, Carbapenem-resistant *Klebsiella pneumoniae*; CRPA, Carbapenem-resistant *Pseudomonas aeruginosa*; MRSA, Methicillin-resistant *Staphylococcus aureus*; VRE, Vancomycin-resistant *Enterococcus faecium/faecalis*.

‡Either non or only study-reported estimates for the specific antibiotic-bacterium pair. Full charts, including the studies, can be found in S1 Text, section 7. *P*-values (p) were reported using a two-sided z-test (α = 5%) for the log-transformed mortality and ICU admission ratios and LOS’s SMD.

ARB, antibiotic-resistant bacteria; CI, confidence interval; ICU, intensive care unit; LOS, length of hospital stay; OR, odds ratio; SMD, standardised mean difference; WHO, World Health Organization.

### Estimated excess costs

**Fig 4:**
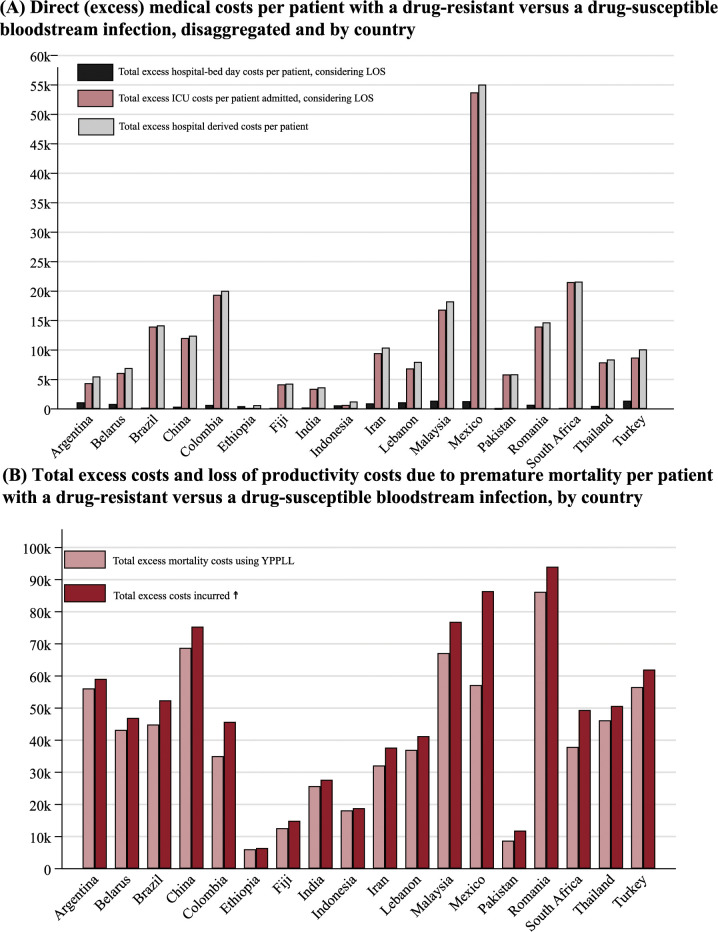
Excess costs (in 2020 USD) associated with productivity loss or excess length of stay per patient with a drug-resistant versus a drug-sensitive bloodstream infection. (A) Direct excess medical costs dissagreggated by ICU and hospital-bed days and by country (B) Total excess costs and productivity lossess due to premature mortality by country. ARB, antibiotic-resistant bacteria; BSI, bloodstream infection; YPLL, years of potential life lost from premature mortality; LOS, length of stay; USD, United States dollars. Full information and data are provided in S1 Text, section 4. ☨ Total excess costs incurred including YPLL and hospital-derived costs per patient with ARB BSI. “k” = thousands. Costs of productivity loss are found in Table L in S1 Text.

The average excess hospital bed-days cost per ARB BSI patient in tertiary/teaching hospitals, adjusted by the calculated excess LOS from Table 2 and excluding drugs and tests costs, was $812.5 (95% CI [$331.6 to $1,293.3]) (Table J in S1 Text). The excess costs per patient varied considerably between countries, ranging from $30.9, $95.9, and $131.7 (Ethiopia, Pakistan, and India, respectively) to $1,681.7 and $1,683.2 (Mexico and Turkey) (Fig 4, panel A).

We estimated an average excess of productivity loss (indirect costs associated with ARB BSI for an average patient) from years of potential life lost due to premature mortality of $41,102 (95% CI = $30,931 to $51,274) for all bacteria combined (Table L in S1 Text). Romania presented the highest excess producitivity lossess attributed to years of potential life-lost costs per patient, while Ethiopia had the lowest ($86,217 and $6,070, respectively). Mortality costs due to premature mortality using the life expectancy approach had an observed average of $132,560 per patient (95% CI [$99,753 to $165,363]) among all sampled countries (Table L in S1 Text).

The average excess ICU admission costs per patient, multiplied by the calculated ICU LOS, was $11,629 (95% CI [$6,016 to $17,243]) (Table O in S1 Text) for all bacteria combined. The estimates varied, with a middle data dispersion of $5,669 (i.e., third quartile–second quartile). Mexico had the highest costs per patient ($53,747), and Ethiopia had the lowest ($188) (Table O in S1 Text).

Fig 4 displays the direct medical and productivity loss due to premature mortality costs per patient by country (panel B). Direct medical costs (i.e., hospital bed-day costs and bed-day ICU costs per day multiplied by the average hospital and ICU respective LOS) were estimated at $12,442 (95% CI [$6,693 to $18,191]). The average total excess costs for a patient with ARB compared to ASB BSI, comprising direct medical and years of potential life lost, were $53,545 (95% CI [$39,838 to $67,251]). Excess costs for ICU adjusted to ICU’s length of stay were 14 times higher compared with hospital-bed LOS-adjusted among patients with ARB BSIs. Lower middle-income countries had the lowest economic burdens per patient; however, we found substantial between-country differences.

Full details on cost calculation can be found in S1 Text, section 4.

### Quality and risk assessment

Using the MASTER scale for methodological assessment, we calculated, on average, 25.1, 23.7, and 23.6 points (out of 36) for the mortality, ICU admission, and length of hospital stay outcomes, respectively (Table 4). Our scores reflect that few studies addressed key confounders (e.g., using statistical methods to control for other correlated risk factors) to account for different prognoses and equal ascertainment (especially for participants, analysts, and caregivers’ blindness towards evaluation; <2% of included studies). Only 37%, 11%, and 13% of the studies incorporated statistical techniques (e.g., regression analyses, stratification, matching, among others) for an equal prognosis for the mortality, ICU admission, and LOS outcomes, respectively (Table 4, equal prognosis scores). Most studies achieved equal retention (e.g., low missing data and null attrition) and sufficient analyses safeguards (e.g., absence of numerical contradictions and data dredging), regardless of the outcome analysed. Full results are found in S1 Text sections 8 and 9 and S1 Data, Master Scale spreadsheet.

**Table 4:** Assessment of study quality and risk of bias using the MASTER scale.

Safeguard items and sub-items	Outcomes		
	Mortality	ICU admission	LOS
*Equal recruitment*	60.4%	58.9%	60.6%
**1.** Data collected after the start of the study was not used to exclude participants or to select them for the analysis	38.8%	39.6%	40.0%
**2.** Participants in all comparison groups met the same eligibility requirements and were from the same population and timeframe	100.0%	100.0%	100.0%
**3.** Determination of eligibility and assignment to treatment group/exposure strategy were synchronised	17.5%	11.3%	12.5%
**4.** None of the eligibility criteria were common effects of exposure and outcome	85.4%	84.9%	90.0%
*Equal retention*	96.9%	97.4%	96.5%
**5.** Any attrition (or exclusions after entry) was less than 20% of total participant numbers	92.2%	94.3%	87.5%
**6.** Missing data was less than 20%	97.1%	96.2%	97.5%
**7.** Analysis accounted for missing data	96.1%	96.2%	97.5%
**8.** Exposure variations/treatment deviations were less than 20%	100.0%	100.0%	100.0%
**9.** The analysis addressed variations in exposure or withdrawals after start of the study	99.0%	100.0%	100.0%
*Equal ascertainment*	57.1%	57.4%	57.1%
**10.** Procedures for data collection of covariates were reliable and the same for all participants	100.0%	100.0%	100.0%
**11.** The outcome was objective and/or reliably measured	100.0%	100.0%	100.0%
**12.** Exposures/interventions were objectively and/or reliably measured	100.0%	100.0%	100.0%
**13.** Outcome assessor(s) were blinded	100.0%	100.0%	100.0%
**14.** Participants were blinded	0.0%	0.0%	0.0%
**15**. Caregivers were blinded	0.0%	0.0%	0.0%
**16.** Analyst(s) were blinded	0.0%	1.9%	0.0%
*Equal implementation*	64.6%	66.4%	66.3%
**17.** Care was delivered equally to all participants	0.0%	0.0%	0.0%
**18.** Cointerventions that could impact the outcome were comparable between groups or avoided	0.9%	0.0%	0.0%
**19.** Control and active interventions/exposures were sufficiently distinct	100.0%	100.0%	100.0%
**20.** Exposure/intervention definition was consistently applied to all participants	87.4%	98.1%	97.5%
**21.** Outcome definition was consistently applied to all participants	100.0%	100.0%	100.0%
**22.** The period between exposure and outcome was similar across patients and between groups or the analyses adjusted for different lengths of follow-up of patients	99.0%	100.0%	100.0%
*Equal prognosis*	37.6%	11.0%	12.5%
**23.** Design and/or analysis strategies were in place that addressed potential confounding	84.5%	0.0%	0.0%
**24.** Key confounders addressed through design or analysis were not common effects of exposure and outcome	69.9%	0.0%	0.0%
**25**. Key baseline characteristics/prognostic indicators for the study were comparable across groups	3.9%	0.0%	2.6%
**26.** Participants were randomly allocated to groups with an adequate randomisation process	4.9%	9.4%	10.0%
**27.** Allocation procedure was adequately concealed	0.0%	0.0%	0.0%
**28.** Conflict of interests were declared and absent	62.1%	56.6%	62.5%
*Sufficient analysis*	89.9%	92.3%	92.5%
**29.** Analytic method was justified by study design or data requirements	84.2%	88.5%	90.0%
**30.** Computation errors or contradictions were absent	93.2%	94.3%	90.0%
**31.** There was no discernible data dredging or selective reporting of the outcomes	92.2%	94.2%	97.4%
*Temporal precedence*	100.0%	100.0%	100.0%
**32.** All subjects were selected prior to intervention/exposure and evaluated prospectively	100.0%	100.0%	100.0%
**33.** Carry-over or refractory effects were avoided or considered in the design of the study or were not relevant	100.0%	100.0%	100.0%
**34.** The intervention/exposure period was long enough to have influenced the study outcome	100.0%	100.0%	100.0%
**35.** Dose of intervention/exposure was sufficient to influence the outcome	100.0%	100.0%	100.0%
**36.** Length of follow-up was not too long or too short in relation to the outcome assessment	100.0%	100.0%	100.0%
Average count of safeguard items (raw score out of 36 items)	25.1	23.6	23.7
Average percentage of sufficiency considering all 36 items (i.e., average raw score/36)	69.6%	65.6%	65.9%

Percentage of fulfilment among all included studies, and per outcome, is presented by MASTER’s scale safeguard and items [21].

ICU, intensive care unit; LOS, length of hospital stay. Full results are reported in S1 Data, Master Scale spreadsheet. See S1 Text, section 9, for a subgroup meta-analysis according to quality scores.

### Small-study effects

We found a medium level of heterogeneity between studies for the mortality outcome (I^2^ 69%, 95% CI [52% to 78%]), and high variation for ICU admission (I^2^ 91%, 95% CI [83% to 94%]) and LOS (I^2^ 90%, 95% CI [75%, 95%]) for the meta-analysis run by specific groups (S1 Text, section 5). Studies reporting ICU admission and LOS were either symmetrical (LFK index ≤1) or slightly asymmetrical (LFK index <3) (Figs AM and AN in S1 Text).

### Sensitivity analyses

General mortality estimates from studies in China were not different from studies conducted elsewhere. However, we found larger disaggregated estimates for subgroup meta-analyses, such as Enterobacteriaceae, Moraxellaceae, Pseudomonaceae, and Staphylococcaceae species (8%, 25%, 26%, and 20%, respectively) compared to the average mortality estimates reported in Table 2 for the same subgroups. General LOS SMD was 16% higher among countries other than China, compared to the estimates reported in Table 2, specifically driven by Moraxellaceae and Staphylococcaceae species. Finally, the odds for excess ICU admission were 25% greater in China, with respect to average ICU admission found in all included studies, driven by 27% elevated odds among patients having BSIs caused by gram-negative bacteria. Full results in Tables U and V in S1 Text.

When applying the leave-one-out method to our meta-analyses, we observed that after assessing the effect of every single study on the overall estimates, the numbers presented a relative variation with respect to overall estimates ranging between −2% and 4% for mortality (OR 95% CI [1.57 to 1.58]), −8% and 4% for ICU admission (OR 95% CI [1.95 to 1.97]), and −10% and 4% for LOS (SMD 95% CI [0.48 to 0.50]) (S1 Text, section 6). These results suggest a moderate influence of our studies in the overall estimates if relative variations are compared, especially for ICU admission and LOS.

## Discussion

Antibiotic resistance imposes substantial morbidity, mortality, and societal costs in LMICs [153]. Bloodstream infections with ARB are among the most lethal, imposing a large disease burden. Examining all available data for hospitalised patients in LMICs, we found that ARB BSIs with WHO critical- and high-priority pathogens were associated with increased mortality (OR 1.58, 95% CI [1.35 to 1.80]), overall length of stay (SMD 0.49, 95% CI [0.20 to 0.78]), and ICU admission (OR 1.96, 95% CI [1.56 to 2.47]).

Our findings on mortality are consistent with the recent estimates by the Global Burden of Disease study [154]. The largest mortality impact was associated with resistant *A*. *baumannii* and Enterobacteriaceae. Both bacteria featured in the global top 5 contributors to resistance-associated and -attributable deaths in 2019 [154]. Between a quarter and half of the patients with ARB BSIs caused by Enterobacteriaceae, *A*. *baumannii* or *P*. *aureginosa* die, corroborating findings from different country settings for Enterobacteriaceae [8,67], *P*. *aeruginosa* [155], and large university hospitals in Israel and the US for *A*. *baumanii* [156,157].

Our results suggest that patients who acquired ARB BSIs during their hospital stay had an overall hospital stay that is about a week longer than patients that acquired ASB BSIs. However, in our study, we could not distinguish between excess length of stay before or after BSI, and as such this is likely an overestimation. Depending on the pathogen, resistant infections have previously been shown to increase LOS typically by 2.0 to 12.7 days [158]. Longer hospital stay, especially before BSI onset, is a primary risk factor for acquiring a resistant infection due to the cumulative risk of hospital transmission of ARBs [158,159]. We found that MRSA had the greatest impact on LOS (extending stay by 14 days relative to sensitive *S*. *aureus*). Others have also shown considerably increased LOS as a result of MRSA compared with sensitive *S*. *aureus*: Tsuzuki and colleagues [160] showed an excess overall LOS and LOS after BSI onset of 20 and 7 days, respectively; similarly, Graffunder and colleagues [161] showed MRSA patients presented an overall LOS of 3 weeks longer. Resistant infections are more difficult to treat and increase the rate of ICU admissions. Our analysis showed that resistant Enterobacteriaceae infections more than doubled the odds of ICU admission. This finding is comparable with the 2.69 higher odds of ICU admission previously shown among patients with carbapenem-resistant *K*. *pneumoniae* BSIs [162]. Our exploratory analysis for studies performed in China and LMICs other than China exhibited divergent results. We found that China’s patients with antibiotic-resistant gram-negative BSIs (*A*. *baumanii*, Enterobacteriaceae, and *P*. *aeruginosa*) displayed higher excess mortality, ICU admission, and LOS, compared to the other LMICs with reported data. Large increases in antibiotic consumption and resistance levels over the last 20 years and the rapid development or acquisition of drug resistance among gram-negative pathogens might explain the greater excess mortality and morbidity for ARB BSIs in China [1,163,164]. Correspondingly, inappropriate administration of empirical treatments and low testing rates could increase the burden outcomes for patients with ARB BSIs in these settings [165].

Despite being fundamental to resource allocation for healthcare provision, we found very little data on excess costs associated with ARB BSIs among the reviewed studies. One study conducted in Thailand, reported excess costs associated with hospital-acquired carbapenem-resistant *A*. *baumannii* of $5,682 [61]. A study conducted in Colombia, reported excess hospitalisation costs associated with MRSA BSI of $10,212, compared to sensitive *S*. *aureus* [53]. We estimated costs associated with mortality, LOS, and ICU admissions from the provider and societal perspective following the WHO-CHOICE standards and human capital approach. We found that the average hospital-related 2020 USD excess costs were $12,442 (95% CI [$6,693 to $18,190]) per ARB BSI patient, compared to ASB, ranging between Ethiopia, with the lowest figures, to Mexico, with the highest. These differences are partly explained by the countries’ disparate economies (Pearson correlation = 0.27 between GDP and hospital costs). Several LMIC-setting studies detailing excess costs of resistant infections were excluded from our review because they did not meet specific inclusion criteria. Cost estimates from these studies include 1 from Turkey in which excess hospital stay and treatment costs were $10,002 [166]. Our estimate for Turkey of $10,403 is similar; however, our estimates did not include therapy/treatment costs. Our estimate for China ($12,516) was higher than a previous study including BSI treatment costs for carbapenem-resistant *K*. *pneumoniae* ($10,763) [167]. The average excess total costs comprising direct medical costs and years of potential life lost associated with premature mortality were $53,545 (95% CI [$39,838 to $67,251]) per patient with ARB BSI. WHO [168] recently reported that 58.3% of 22,371 isolates were identified as ARB *E*. *coli*, while 33.3% of 23,031 isolates were ARB *S*. *aureus* in LMICs, indicating the high relevance of these costs.

This study has limitations. First, the most important limitation is consistent with conclusions from the Global Burden of Diseases study [154]: there is a sparsity of data on ARB from LMICs. Only 18 of the 137 (13%) LMICs published any AMR outcome study. Consistent antibiotic resistance surveillance puts demands on clinical bacteriology, quality control, and data linkage between culture test results and clinical outcomes, which is beyond the capabilities of many LMICs. Applying the leave-one-out method to our meta-analyses (S1 Text, section 6) showed a minor-to-moderate influence of individual studies likely due to the heterogeneity in clinical settings, indicating that our model’s results are robust (assuming countries’ missing information and selection biases are heterogeneously distributed). Future efforts to improve coverage should prioritise WHO’s Africa region, where data were remarkably absent, with no estimates for resistance-associated LOS or ICU admissions. Our results indicate that the studies from the Western Pacific and European areas show the highest excess mortality from ARB BSIs. Studies from Africa show among the lowest but this region has limited data and substantial uncertainty; it is essential to improve epidemiological surveillance of ARB BSIs in this region in particular [169]. Second, some articles were of low quality or reported limited data. Studies often failed to account for confounding factors; hence our analyses relied upon crude estimates. ARB surveillance networks vary in blood culture sampling, potentially overestimating the number of severe cases if selective sampling among patients fulfilling the case definition is present. Third, we did not estimate the total relative harm of ARB BSIs relative to where such infections were prevented (compared to non-infected patients) [170], primarily because of the limited number of studies [171]. While we accounted for some key risk factors when comparing antibiotic-sensitive and antibiotic-resistant groups in the metaregression, others were unavailable. We could not match comparison groups by factors known to impact patients’ underlying health conditions, such as illness severity, prolonged previous hospital stays, or the use of invasive devices. The reported LOS does not distinguish between total LOS and LOS following BSI infection, thus risking reverse causality [172]. This ecological study was designed to identify associations; consequently, our results should be interpreted cautiously. Also, we adjusted WHO-CHOICE country estimates using US GPD implicit price deflators, which may not necessarily reflect price changes in some LMICs, particularly for non-tradable cost components of healthcare. Finally, we may have overestimated the true effect size of the association between ARB BSIs and mortality as indicated by the exploratory analysis of studies’ adjusted—compared to unadjusted—ORs reporting both estimates, specifically among gram-negative species.

Here, we described an updated evaluation of the health impact and excess economic costs of resistant BSIs in low-resourced settings. Our results highlight regions where improved surveillance, expanding microbiology laboratory capacity, and data collection systems are most needed and where the current evidence indicates WHO critical and high-priority drug-resistant pathogens exert the greatest toll on morbidity and mortality.

## Supporting information

S1 Text Supporting text, tables, and figures. **Text A.** Search criteria used by search engine. **Table A.** Studies inclusion and exclusion criteria. **Table B.** Years of the studies included. **Table C.** Number of studies included by WHO region and WB income group. **Table D.** Correlation between main outcomes and demographic variables. **Table E.** Most prevalent bacterium family, Gram type, resistance type, and antibiotic-bacterium pair by country among the included studies. **Table F.** Descriptive statistics of the studies included in the meta-analysis. **Table G.** Summary of the subgroup meta-analysis results for income level and WHO region by outcome variable. **Table H.** Costs of hospital bed-day per patient and by country and hospital level (in 2008 USDs). **Table I.** Costs of total excess hospital bed-days per patient by country and hospital level using estimated SMD and their respective 95% CIs (in 2008 USDs). **Table J.** Costs of total excess hospital bed-days per patient and by country and hospital level using estimated SMD and their respective 95% CIs (inflated to 2020 USDs). **Table K.** Calculation of YPLL, YPPLL, and CPL, by country. **Table L.** Total productivity losses due to premature mortality costs by country using the LE at the age of death and productivity cost approach (age of retirement), discounted. **Table M.** Intensive care unit costs per patient (daily). **Table N.** Intensive care unit costs (per patient and daily) adjusted to 2020 USDs (inflated accordingly). **Table O.** Intensive care unit costs (per day/patient) adjusted to ICU LOS and reported in 2020 USDs (inflated accordingly). **Table P.** Total excess costs incurred for bloodstream infections caused by antibiotic-resistant bacteria, per patient. **Table Q.** Statistics calculated for meta-analysis using mortality as an outcome, by model. **Table R.** Statistics calculated for meta-analysis using ICU admission as an outcome, by model. **Table S.** Statistics calculated for meta-analysis using the length of stay at hospital as an outcome, by model. **Table T.** Summary of the subgroup meta-analysis results for specific antibiotic-bacterium combinations declared important by the WHO, by outcome variable. **Table U.** Meta-analysis subgroup results for bacterium family, and Gram type for those studies carried out in China and other than China, by outcome. **Table V.** Summary results of meta-analysis results for critical antibiotic-bacterium pathogens for those studies in China and other than China, by outcome. **Table W.** Meta-regression results for the mortality outcome (univariate and multivariable). **Table X.** Meta-regression results for the ICU admission outcome (univariate and multivariate). **Table Y.** Summary results of the meta-analysis for the main outcome variables by separating the studies for low- [LS] and high-scores [HS] obtained from the MASTER scale. **Table Z.** Checklist of information that should be included in new reports of global health estimates. **Table AA.** PRISMA Checklist. **Fig A.** Density of the studies over time. **Fig B.** Violin and kernel density estimate plots for the main outcomes and by ARB susceptibility. **Fig C.** Relationship between the main outcomes. **Fig D.** Meta-analysis using all the studies reporting mortality rates. **Fig E**. Subgroup meta-analysis using all the studies reporting mortality rates/odds for critical (*N* = 72) and high-priority (*N* = 22) pathogens according to the WHO criteria. **Fig F.** Subgroup meta-analysis using all the studies reporting mortality rates by bacterium’s family name. **Fig G.** Subgroup meta-analysis using all the studies reporting mortality rates by WHO Region. **Fig H.** Subgroup meta-analysis using all the studies reporting mortality rates by income level. **Fig I.** Meta-analysis results using all the studies reporting the mean and SD for the length of stay at the hospital. **Fig J**. Subgroup meta-analysis using all the studies reporting the mean and SD for the length of stay at the hospital for critical and high-priority pathogens according to the WHO. **Fig K.** Subgroup meta-analysis using all the studies reporting the mean and SD for the length of stay at the hospital for Enterococcus spp., Enterobacteriaceae, Moraxellaceae, Pseudomonadaceae, and Staphyloccocaceae. **Fig L.** Subgroup meta-analysis using all the studies reporting the mean and SD for the length of stay at the hospital by income level. **Fig M.** Subgroup meta-analysis using all the studies reporting the mean and SD for the length of stay at the hospital by WHO region. **Fig N.** Meta-analysis results using all the studies reporting ICU admission rates. **Fig O.** Subgroup meta-analysis using all the studies reporting ICU admission rates for critical pathogens according to the WHO criteria. **Fig P.** Subgroup meta-analysis using all the studies reporting ICU admission rates for high-priority pathogens according to the WHO criteria. **Fig Q.** Subgroup meta-analysis using all the studies reporting ICU admission rates for Enterobacteriaceae. **Fig R.** Subgroup meta-analysis using all the studies reporting ICU admission rates for Enterobacteriaceae. **Fig S.** Subgroup meta-analysis using all the studies reporting ICU admission rates for Moraxellaceae. **Fig T.** Subgroup meta-analysis using all the studies reporting ICU admission rates for Pseudomonadaceae. **Fig U.** Subgroup meta-analysis using all the studies reporting ICU admission rates for Staphylococcaceae. **Fig V.** Subgroup meta-analysis using all the studies reporting ICU admission rates by resistance type (ESBL+). **Fig W.** Subgroup meta-analysis using all the studies reporting ICU admission rates by WHO region: Americas. **Fig X.** Subgroup meta-analysis using all the studies reporting ICU admission rates by WHO region: Eastern Mediterranean. **Fig Y.** Subgroup meta-analysis using all the studies reporting ICU admission rates by WHO region: Europe. **Fig Z.** Subgroup meta-analysis using all the studies reporting ICU admission rates by WHO region: Southeast Asia. **Fig AA**. Subgroup meta-analysis using all the studies reporting ICU admission rates by WHO region: Western Pacific region. **Fig AB.** Subgroup meta-analysis using all the studies reporting ICU admission rates by income level: Low and lower-middle income countries. **Fig AC.** Subgroup meta-analysis using all the studies reporting ICU admission rates by income level: Upper-middle income countries. **Fig AD.** Subgroup analysis for studies reporting unadjusted ORs. **Fig AE.** Subgroup analysis for studies reporting unadjusted ORs, by bacteria’s gram type or WHO criticality category (critical = gram-negative, high-priority = gram-positive in this study). **Fig AF.** Subgroup analysis for studies reporting unadjusted ORs, by specific bacterium. **Fig AG.** Subgroup analysis for studies reporting adjusted ORs. **Fig AH.** Subgroup analysis for studies reporting adjusted ORs, by bacteria’s gram type (critical = gram-negative, high-priority = gram-positive in this study). **Fig AI.** Subgroup analysis for studies reporting adjusted ORs, by specific bacterium. **Fig AJ.** Subgroup analysis for studies reporting adjusted and unadjusted ORs simultaneously, general mortality estimates. **Fig AK.** Subgroup analysis for studies reporting adjusted and unadjusted ORs simultaneously, mortality rates by Gram type or WHO criticality list classification (high = gram-positive, critical = gram-negative). **Fig AL.** Subgroup analysis for studies reporting adjusted and unadjusted ORs simultaneously, mortality rates by bacterium family. **Fig AM.** Doi plots for Model 1 (general) and by outcome based on Tables Q, R, and S. **Fig AN.** Funnel plots for Model 1 (general) and by outcome based on Tables Q, R, and S. **Fig AO.** Influence analysis for Model 1 using the mortality outcome compared to the general estimates and without subgroup analyses. **Fig AP.** Influence analysis for Model 1 using the ICU admission outcome compared to the general estimates and without subgroup analyses. **Fig AQ.** Influence analysis for Model 1 using the length of hospital stay outcome compared to the general estimates and without subgroup analyses. **Fig AR.** Meta-analysis results disaggregated by specific and prioritised antibiotic-bacterium pairs for mortality. **Fig AS**. Meta-analysis results disaggregated by carbapenem-resistant Enterobacteriaceae for mortality. **Fig AT.** Meta-analysis results disaggregated by specific and prioritised antibiotic-bacterium pairs for LOS. **Fig AU.** Meta-analysis results disaggregated by carbapenem-resistant Enterobacteriaceae for LOS. **Fig AV.** Meta-analysis results disaggregated by specific and prioritised antibiotic-bacterium pairs for ICU admission. **Fig AW.** Meta-analysis results disaggregated by carbapenem-resistant Enterobacteriaceae for ICU admission. **Fig AX.** Graphical results of Table V. **Fig AY.** Distribution of the Master scale scores by outcome. **Fig AZ.** Kernel density estimate of the Master scale scores by outcome. **Fig BA.** Percentage of full completion by MASTER scale main safeguard and outcome.
(PDF)

S1 Data Supporting dataset of the included studies and results of the application of the MASTER scale. **MasterData spreadsheet.** Description and data extracted from each included study. **MasterScale spreadsheet.** Application of the MASTER scale by outcome and study. **Summary MasterScale spreadsheet**. Summary statistics per safeguard/item of the application of the MASTER scale.
(XLSX)

## Abbreviations

AIM: African Index Medicus
ARB: antibiotic-resistant bacteria
ASB: antibiotic-sensitive bacteria
BSI: bloodstream infection
GLASS: Global Antimicrobial Resistance and Surveillance System
GNI: gross national income
ICU: intensive care unit
LMIC: low- and middle-income country
LOS: length of hospital stay
MRSA: methicillin-resistant Staphylococcus aureus
OR: odds ratio
SMD: standardised mean difference
WHO: World Health Organization

## References

[1] Organisation for Economic Cooperation and Development. Stemming the Superbug Tide: Just a Few Dollars More: OECD; 2019.

[2] Okeke IN, Laxminarayan R, Bhutta ZA, Duse AG, Jenkins P, O’Brien TF, et al. Antimicrobial resistance in developing countries. Part I: recent trends and current status. Lancet Infect Dis. 2005;5(8):481–493. doi: 10.1016/S1473-3099(05)70189-4 16048717

[3] Cassini A, Högberg LD, Plachouras D, Quattrocchi A, Hoxha A, Simonsen GS, et al. Attributable deaths and disability-adjusted life-years caused by infections with antibiotic-resistant bacteria in the EU and the European Economic Area in 2015: a population-level modelling analysis. Lancet Infect Dis. 2019;19(1):56–66. doi: 10.1016/S1473-3099(18)30605-4 30409683 PMC6300481

[4] World Health Organization. Global antimicrobial resistance and use surveillance system (GLASS) report: 2021. 2021.

[5] Tacconelli E, Carrara E, Savoldi A, Harbarth S, Mendelson M, Monnet DL, et al. Discovery, research, and development of new antibiotics: the WHO priority list of antibiotic-resistant bacteria and tuberculosis. Lancet Infect Dis. 2018;18(3):318–327. doi: 10.1016/S1473-3099(17)30753-3 29276051

[6] Hattori H, Maeda M, Nagatomo Y, Takuma T, Niki Y, Naito Y, et al. Epidemiology and risk factors for mortality in bloodstream infections: A single-center retrospective study in Japan. Am J Infect Control. 2018;46(12):e75–e79. doi: 10.1016/j.ajic.2018.06.019 30172607

[7] de Kraker ME, Wolkewitz M, Davey PG, Grundmann H. Clinical impact of antimicrobial resistance in European hospitals: excess mortality and length of hospital stay related to methicillin-resistant Staphylococcus aureus bloodstream infections. Antimicrob Agents Chemother. 2011;55(4):1598–1605. doi: 10.1128/AAC.01157-10 21220533 PMC3067153

[8] De Kraker M, Wolkewitz M, Davey P, Koller W, Berger J, Nagler J, et al. Burden of antimicrobial resistance in European hospitals: excess mortality and length of hospital stay associated with bloodstream infections due to Escherichia coli resistant to third-generation cephalosporins. J Antimicrob Chemother. 2011;66(2):398–407. doi: 10.1093/jac/dkq412 21106563

[9] Thaden JT, Li Y, Ruffin F, Maskarinec SA, Hill-Rorie JM, Wanda LC, et al. Increased costs associated with bloodstream infections caused by multidrug-resistant gram-negative bacteria are due primarily to patients with hospital-acquired infections. Antimicrob Agents Chemother. 2017;61(3):e01709–e01716. doi: 10.1128/AAC.01709-16 27993852 PMC5328522

[10] Wozniak TM, Barnsbee L, Lee XJ, Pacella RE. Using the best available data to estimate the cost of antimicrobial resistance: a systematic review. Antimicrob Resist Infect Control. 2019;8(1):1–12. 30733860 10.1186/s13756-019-0472-z PMC6359818

[11] Lee H-Y, Chen C-L, Liu S-Y, Yan Y-S, Chang C-J, Chiu C-H. Impact of molecular epidemiology and reduced susceptibility to glycopeptides and daptomycin on outcomes of patients with methicillin-resistant Staphylococcus aureus bacteremia. PLoS ONE. 2015;10(8):e0136171. doi: 10.1371/journal.pone.0136171 26295150 PMC4546585

[12] Biehle LR, Cottreau JM, Thompson DJ, Filipek RL, O’Donnell JN, Lasco TM, et al. Outcomes and risk factors for mortality among patients treated with carbapenems for Klebsiella spp. bacteremia. PLoS ONE. 2015;10(11):e0143845. 26618357 10.1371/journal.pone.0143845 PMC4664260

[13] Cheah A, Spelman T, Liew D, Peel T, Howden B, Spelman D, et al. Enterococcal bacteraemia: factors influencing mortality, length of stay and costs of hospitalization. Clin Microbiol Infect. 2013;19(4):E181–E189. doi: 10.1111/1469-0691.12132 23398607

[14] Naylor NR, Atun R, Zhu N, Kulasabanathan K, Silva S, Chatterjee A, et al. Estimating the burden of antimicrobial resistance: a systematic literature review. Antimicrob Resist Infect Control. 2018;7(1):1–17. 29713465 10.1186/s13756-018-0336-y PMC5918775

[15] Ang H, Sun X. Risk factors for multidrug-resistant Gram-negative bacteria infection in intensive care units: A meta-analysis. Int J Nurs Pract. 2018;24(4):e12644. doi: 10.1111/ijn.12644 29575345

[16] Saharman YR, Karuniawati A, Severin JA, Verbrugh HA. Infections and antimicrobial resistance in intensive care units in lower-middle income countries: a scoping review. Antimicrob Resist Infect Control. 2021;10(1):1–19. 33514432 10.1186/s13756-020-00871-x PMC7844809

[17] Akova M. Epidemiology of antimicrobial resistance in bloodstream infections. Virulence. 2016;7(3):252–266. 10.1080/21505594.2016.1159366 PMC4871634 26984779

[18] Moher D, Liberati A, Tetzlaff J, Altman DG, Group P. Preferred reporting items for systematic reviews and meta-analyses: the PRISMA statement. PLoS Med. 2009;6(7):e1000097. doi: 10.1371/journal.pmed.1000097 19621072 PMC2707599

[19] World Bank. World Bank Country and Lending Groups 2021 [cited 2021 Aug 31]. Available from: https://datahelpdesk.worldbank.org/knowledgebase/articles/906519-world-bank-country-and-lending-groups.

[20] World Health Organization. Antimicrobial resistance: global report on surveillance: World Health Organization. 2014.

[21] Stone JC, Glass K, Clark J, Ritskes-Hoitinga M, Munn Z, Tugwell P, et al. The MethodologicAl STandards for Epidemiological Research (MASTER) scale demonstrated a unified framework for bias assessment. J Clin Epidemiol. 2021. doi: 10.1016/j.jclinepi.2021.01.012 33485928

[22] Doi SA, Barendregt JJ, Khan S, Thalib L, Williams GM. Advances in the meta-analysis of heterogeneous clinical trials I: the inverse variance heterogeneity model. Contemp Clin Trials. 2015;45:130–138. doi: 10.1016/j.cct.2015.05.009 26003435

[23] World Health Organization. Choosing interventions that are cost effective (WHO—CHOICE). Cost effectiveness and strategic planning. Available at: http://www.who.int/choice/costs/en/. Accessed March, 2020. 2021.

[24] Khwannimit B, Bhurayanontachai R. The direct costs of intensive care management and risk factors for financial burden of patients with severe sepsis and septic shock. J Crit Care. 2015;30(5):929–934. doi: 10.1016/j.jcrc.2015.05.011 26051981

[25] Kockaya PD, Kavuncubasi S, Kockaya G. Cost of Intensive Care Stay in Turkey: In the View of Payer and Health Care Provider. Value Health. 2013;16(7):A466.

[26] Mahomed S, Mahomed O. Cost of intensive care services at a central hospital in South Africa. S Afr Med J. 2019;109(1):35–39. 10.7196/SAMJ.2018.v109i1.13268 30606302

[27] Lorenzovici L, Székely A, Csanádi M, Gaál P. Cost assessment of inpatient care episodes of stroke in Romania. Front Public Health. 2020;8. 33344405 10.3389/fpubh.2020.605919 PMC7746609

[28] Haque A, Naveed-ur-Rehman Siddiqui RK, Hoda M, Lakahni G, Hooda K. Cost of care in a paediatric intensive care unit of a tertiary-care university hospital of Pakistan. Trauma. 2015;21:14.1. 26060165

[29] Velázquez LDS. Análisis de costos en las Unidades de Terapia Intensiva mexicanas. Estudio multicéntrico. Medicina Crítica. 2010;24(4):159–166.

[30] Aung YN, Nur AM, Ismail A, Aljunid SM. Determining the cost and length of stay at intensive care units and the factors influencing them in a teaching hospital in Malaysia. Value Health Reg Issues. 2020;21:149–156. doi: 10.1016/j.vhri.2019.09.006 31958748

[31] Soleymani F. Costs of hospital-acquired infection for patients hospitalized in intensive care unit of an Iranian referral hospital. Med J Islam Repub Iran. 2018;32:67. doi: 10.14196/mjiri.32.67 30643742 PMC6325278

[32] Peter JV, Thomas K, Jeyaseelan L, Yadav B, Sudarsan TI, Christina J, et al. Cost of intensive care in India. Int J Technol Assess Health Care. 2016;32(4):241–245. doi: 10.1017/S0266462316000398 27608529

[33] Olivera COE, Urrego KAG, Duque MG, Góngora EM. Costos de atención en UCI de un Hospital universitario de Bogotá DC. Revista Repertorio de Medicina y Cirugía. 2006;15(3):133–142.

[34] Cong Y. Ethical challenges in critical care medicine: a Chinese perspective. J Med Philos. 1998;23(6):581–600. doi: 10.1076/jmep.23.6.581.2558 10190842

[35] Sogayar AM, Machado FR, Rea-Neto A, Dornas A, Grion CM, Lobo SM, et al. A multicentre, prospective study to evaluate costs of septic patients in Brazilian intensive care units. Pharmacoeconomics. 2008;26(5):425–434. doi: 10.2165/00019053-200826050-00006 18429658

[36] Rosenthal VD, Guzman S, Migone O, Crnich CJ. The attributable cost, length of hospital stay, and mortality of central line-associated bloodstream infection in intensive care departments in Argentina: a prospective, matched analysis. Am J Infect Control. 2003;31(8):475–480. doi: 10.1016/j.ajic.2003.03.002 14647110

[37] Tan SS, Bakker J, Hoogendoorn ME, Kapila A, Martin J, Pezzi A, et al. Direct cost analysis of intensive care unit stay in four European countries: applying a standardized costing methodology. Value Health. 2012;15(1):81–86. doi: 10.1016/j.jval.2011.09.007 22264975

[38] Evans J, Kobewka D, Thavorn K, D’Egidio G, Rosenberg E, Kyeremanteng K. The impact of reducing intensive care unit length of stay on hospital costs: evidence from a tertiary care hospital in Canada. Can J Anesth. 2018;65(6):627–635. 29476403 10.1007/s12630-018-1087-1

[39] Oostenbrink JB. Buijs-Van der Woude T, van Agthoven M, Koopmanschap MA, Rutten FF. Unit costs of inpatient hospital days. Pharmacoeconomics. 2003;21(4):263–271. doi: 10.2165/00019053-200321040-00004 12600221

[40] Springer. Human Capital Approach. In: Kirch W, editor. Encyclopedia of Public Health. Dordrecht: Springer Netherlands; 2008. p. 697–8.

[41] Murray CJ. Comprehensive systematic analysis of global epidemiology: definitions, methods, simplification of DALYs, and comparative results from the Global Burden of Disease Study 2010. Supplement to: Murray CJL, Ezzati M, Flaxman AD, Lim S, Lozano R, Michaud C, et al. GBD 2010: design, definitions, and metrics. Lancet. 2012;380:2063–2066. 23245602 10.1016/S0140-6736(12)61899-6

[42] Haacker M, Hallett TB, Atun R. On discount rates for economic evaluations in global health. Health Policy Plan. 2020;35(1):107–114. doi: 10.1093/heapol/czz127 31625564

[43] Furuya-Kanamori L, Barendregt JJ, Doi SA. A new improved graphical and quantitative method for detecting bias in meta-analysis. Int J Evid Based Halthc. 2018;16(4):195–203. doi: 10.1097/XEB.0000000000000141 29621038

[44] Hastie T, Tibshirani R, Friedman JH, Friedman JH. The elements of statistical learning: data mining, inference, and prediction: Springer; 2009.

[45] Hincapié C, Galeano JA, Tibaduiza MF, Restrepo CA, Garcés D, Caraballo C, et al. Staphylococcemia mortality: Influence of methicillin resistance and site of infection acquisition in a patient’s cohort from Medellin. Colombia. Enferm Infecc Microbiol. 2020;40(1):8–15.

[46] Abhilash K, Veeraraghavan B, Abraham O. Epidemiology and outcome of bacteremia caused by extended spectrum beta-lactamase (ESBL)-producing Escherichia coli and Klebsiella spp. in a tertiary care teaching hospital in south India. J Assoc Physicians India. 2010;58(Suppl):13–17. 21563608

[47] Abolghasemi S, Madadi Z, Mardani M. Risk Factors for Resistance and Mortality in Patients with Extensively Resistant Acinetobacter Bacteremia in Taleghani Hospital in Tehran, Iran. Arch Pediatr Infect Dis. 2018;6(3).

[48] Akhtar N, Sultan F, Nizamuddin S, Zafar W. Risk factors and clinical outcomes for vancomycin-resistant enterococcus bacteraemia in hospitalised cancer patients in Pakistan: A case-control study. J Pak Med Assoc. 2016;66(7):829–36. Epub 2016/07/19. . 27427131

[49] Anggraini D, Santosaningsih D, Endraswari PD, Jasmin N, Siregar FM, Hadi U, et al. Multicenter Study of the Risk Factors and Outcomes of Bloodstream Infections Caused by Carbapenem-Non-Susceptible Acinetobacter baumannii in Indonesia. Trop Med Infect Dis. 2022;7(8):161. doi: 10.3390/tropicalmed7080161 36006253 PMC9412432

[50] Anunnatsiri S, Tonsawan P. Risk factors and clinical outcomes of multidrug-resistant Acinetobacter baumannii bacteremia at a university hospital in Thailand. Southeast Asian J Trop Med Public Health. 2011;42(3):693–703. Epub 2011/06/29. . 21706949

[51] Arias-Ortiz PM, Calderón LP, Castillo JS, Moreno J, Leal AL, Cortés JA, et al. Risk factors for methicillin-resistant Staphylococcus aureus bacteremia: A multicenter matched case-control study. Biomedica. 2016;36(4):612–9. doi: 10.7705/biomedica.v36i4.3193 PubMed Central PMCID: PMC27992988. 27992988

[52] Atmaca Ö, Köşker PZ, Karahan C, Çakir B, Ünal S. Risk factors and antibiotic use in methicillin-resistant Staphylococcus aureus Bacteremia in hospitalized patients at Hacettepe University Adult and Oncology Hospitals (2004–2011) and antimicrobial susceptibilities of the isolates: A nested case-control study. Mikrobiyol Bulteni. 2014;48(4):523–37. doi: 10.5578/mb.8280 PubMed Central PMCID: PMC25492648. 25492648

[53] Barrero LI, Castillo JS, Leal AL, Sánchez R, Cortés JA, Álvarez CA, et al. Economic burden of methicillin-resistant Staphylococcus aureus bacteremia in critical care patients in hospitals in Bogotá. Biomedica. 2014;34(3):345–53. doi: 10.7705/biomedica.v34i3.1692 PubMed Central PMCID: PMC25504122. 25504122

[54] Braga IA, Pirett CC, Ribas RM, Gontijo Filho PP, Diogo Filho A. Bacterial colonization of pressure ulcers: assessment of risk for bloodstream infection and impact on patient outcomes. J Hosp Infect. 2013;83(4):314–20. Epub 2013/01/15. doi: 10.1016/j.jhin.2012.11.008 . 23313027

[55] Castillo Londoño JS, Leal AL, Cortes JA, Alvarez CA, Sanchez R, Buitrago G, et al. Mortality among critically ill patients with methicillin-resistant Staphylococcus aureus bacteremia: A multicenter cohort study in Colombia. Rev Panam Salud Publica Pan Am J Public Health. 2012;32(5):343–50. doi: 10.1590/S1020-49892012001100004 PubMed Central PMCID: PMC23338691. 23338691

[56] Carena AA, Laborde A, Roccia-Rossi I, Palacios CJ, Jordán R, Valledor A, et al. Proposal of a clinical score to stratify the risk of multidrug-resistant gram-negative rods bacteremia in cancer patients. Braz J Infect Dis. 2020;24(1):34–43. doi: 10.1016/j.bjid.2019.11.001 PubMed Central PMCID: PMC31851901. 31851901 PMC9392047

[57] Cetin S, Dokmetas I, Hamidi AA, Bayraktar B, Gunduz A, Sevgi DY. Comparison of risk factors and outcomes in carbapenem-resistant and carbapenem-susceptible Gram-negative bacteremia. Med Bull Sisli Etfal Hospital. 2021;55(3):398. doi: 10.14744/SEMB.2020.49002 34712083 PMC8526226

[58] Chang H, Wei J, Zhou W, Yan X, Cao X, Zuo L, et al. Risk factors and mortality for patients with Bloodstream infections of Klebsiella pneumoniae during 2014–2018: Clinical impact of carbapenem resistance in a large tertiary hospital of China. J Infect Public Health. 2020;13(5):784–90. Epub 2019/12/18. doi: 10.1016/j.jiph.2019.11.014 . 31843651

[59] Chen Y, Chen Y, Liu P, Guo P, Wu Z, Peng Y, et al. Risk factors and mortality for elderly patients with Bloodstream infection of Carbapenem resistance Klebsiella pneumoniae: a 10-year longitudinal study in China. 2022. 10.1186/s12877-022-03275-1 PMC9281020 35831805

[60] Chen R, Yan ZQ, Feng D, Luo YP, Wang LL, Shen DX. Nosocomial bloodstream infection in patients caused by Staphylococcus aureus: drug susceptibility, outcome, and risk factors for hospital mortality. Chin Med J (Engl). 2012;125(2):226–9. Epub 2012/02/22. . 22340550

[61] Chusri S, Chongsuvivatwong V, Silpapojakul K, Singkhamanan K, Hortiwakul T, Charernmak B, et al. Clinical characteristics and outcomes of community and hospital-acquired Acinetobacter baumannii bacteremia. J Microbiol Immunol Infect. 2019;52(5):796–806. Epub 2019/04/30. doi: 10.1016/j.jmii.2019.03.004 . 31031096

[62] Conterno LO, Wey SB, Castelo A. Risk factors for mortality in Staphylococcus aureus bacteremia. Infect Control Hosp Epidemiol. 1998;19(1):32–7. Epub 1998/02/25. doi: 10.1086/647704 . 9475347

[63] Dantas RCC, Silva RTE, Ferreira ML, Gonçalves IR, Araújo BF, De Campos PA, et al. Molecular epidemiological survey of bacteremia by multidrug resistant Pseudomonas aeruginosa: The relevance of intrinsic resistance mechanisms. PLoS ONE. 2017;12(5). doi: 10.1371/journal.pone.0176774 PubMed Central PMCID: PMC28481953. 28481953 PMC5421754

[64] Deodhar D, Varghese G, Balaji V, John J, Rebekah G, Janardhanan J, et al. Prevalence of Toxin Genes among the Clinical Isolates of Staphylococcus aureus and its Clinical Impact. J Glob Infect Dis. 2015;7(3):97–102. Epub 2015/09/24. doi: 10.4103/0974-777X.162234 ; PubMed Central PMCID: PMC4557147. 26392716 PMC4557147

[65] de Oliveira Conterno L, Wey SB, Castelo A. Staphylococcus aureus bacteremia: comparison of two periods and a predictive model of mortality. Braz J Infect Dis. 2002;6(6):288–97. Epub 2003/02/15. doi: 10.1590/s1413-86702002000600004 . 12585972

[66] Deris ZZ, Shafei MN, Harun A. Risk factors and outcomes of imipenem-resistant Acinetobacter bloodstream infection in North-Eastern Malaysia. Asian Pac J Trop Biomed. 2011;1(4):313–5. Epub 2011/08/01. doi: 10.1016/S2221-1691(11)60050-6 ; PubMed Central PMCID: PMC3614228. 23569782 PMC3614228

[67] Dramowski A, Aiken AM, Rehman AM, Snyman Y, Reuter S, Grundmann H, et al. Mortality associated with third-generation cephalosporin resistance in Enterobacteriaceae bloodstream infections at one South African hospital. J Glob Antimicrob Resist. 2022;29:176–184. doi: 10.1016/j.jgar.2022.03.001 35283332 PMC9200643

[68] Durdu B, Hakyemez IN, Bolukcu S, Okay G, Gultepe B, Aslan T. Mortality markers in nosocomial Klebsiella pneumoniae bloodstream infection. Springerplus. 2016;5(1):1892. Epub 2016/11/16. doi: 10.1186/s40064-016-3580-8 ; PubMed Central PMCID: PMC5084144. 27843749 PMC5084144

[69] Ergönül Ö, Aydin M, Azap A, Başaran S, Tekin S, Kaya Ş, et al. Healthcare-associated Gram-negative bloodstream infections: antibiotic resistance and predictors of mortality. J Hosp Infect. 2016;94(4):381–5. Epub 2016/11/03. doi: 10.1016/j.jhin.2016.08.012 . 27717604

[70] Ferreira AM, Moreira F, Guimaraes T, Spadão F, Ramos JF, Batista MV, et al. Epidemiology, risk factors and outcomes of multi-drug-resistant bloodstream infections in haematopoietic stem cell transplant recipients: importance of previous gut colonization. J Hosp Infect. 2018;100(1):83–91. Epub 2018/03/14. doi: 10.1016/j.jhin.2018.03.004 . 29530743

[71] Fu Q, Ye H, Liu S. Risk factors for extensive drug-resistance and mortality in geriatric inpatients with bacteremia caused by Acinetobacter baumannii. Am J Infect Control. 2015;43(8):857–60. Epub 2015/05/12. doi: 10.1016/j.ajic.2015.03.033 . 25960385

[72] Furtado GHC, Mendes RE, Campos Pignatari AC, Wey SB, Medeiros EAS. Risk factors for vancomycin-resistant Enterococcus faecalis bacteremia in hospitalized patients: An analysis of two case-control studies. Am J Infect Control. 2006;34(7):447–51. doi: 10.1016/j.ajic.2005.08.015 PubMed Central PMCID: PMC16945692. 16945692

[73] Garnica M, Maiolino A, Nucci M. Factors associated with bacteremia due to multidrug-resistant Gram-negative bacilli in hematopoietic stem cell transplant recipients. Braz J Med Biol Res. 2009;42(3):289–93. doi: 10.1590/s0100-879x2009000300010 PubMed Central PMCID: PMC19287908. 19287908

[74] Gaytán JJA, Mancilla GC, Meza HAR, Padilla PAV, González CYA, Lara CEG. Tendency of resistance to ciprofloxacin in bacteriemias due to Escherichia coli. Med Interna Mex. 2006;22(5):386–390.

[75] Ghafur AK, Vidyalakshmi PR, Kannaian P, Balasubramaniam R. Clinical study of carbapenem sensitive and resistant Gram-negative bacteremia in neutropenic and nonneutropenic patients: The first series from India. Indian J Cancer. 2014;51(4):453–5. doi: 10.4103/0019-509X.175362 PubMed Central PMCID: PMC26842159. 26842159

[76] Goda R, Sharma R, Borkar SA, Katiyar V, Narwal P, Ganeshkumar A, et al. Frailty and Neutrophil Lymphocyte Ratio as Predictors of Mortality in Patients with Catheter-Associated Urinary Tract Infections or Central Line–Associated Bloodstream Infections in the Neurosurgical Intensive Care Unit: Insights from a Retrospective Study in a Developing Country. World Neurosurgery. 2022;162:e187–e197. doi: 10.1016/j.wneu.2022.02.115 35248769

[77] González AL, Leal AL, Cortés JA, Sánchez R, Barrero LI, Castillo JS, et al. [Effect of adequate initial antimicrobial therapy on mortality in critical patients with Pseudomonas aeruginosa bacteremia]. Biomedica. 2014;34(Suppl 1):58–66. Epub 2014/06/27. doi: 10.1590/s0120-41572014000500008 . 24968037

[78] Guo N, Xue W, Tang D, Ding J, Zhao B. Risk factors and outcomes of hospitalized patients with blood infections caused by multidrug-resistant Acinetobacter baumannii complex in a hospital of Northern China. Am J Infect Control. 2016;44(4):e37–9. Epub 2016/01/26. doi: 10.1016/j.ajic.2015.11.019 . 26804303

[79] Islas-Muñoz B, Volkow-Fernández P, Ibanes-Gutiérrez C, Villamar-Ramírez A, Vilar-Compte D, Cornejo-Juárez P. Bloodstream infections in cancer patients. Risk factors associated with mortality. Int J Infect Dis. 2018;71:59–64. Epub 2018/04/13. doi: 10.1016/j.ijid.2018.03.022 . 29649549

[80] Jafari S, Abdollahi A, Sabahi M, Salehi M, Asadollahi-Amin A, Hasannezhad M, et al. An Update to Enterococcal Bacteremia: Epidemiology, Resistance, and Outcome. Infect Disord Drug Targets. 2020. 10.2174/1871526520999201103191829 33155917

[81] Jamulitrat S, Pranee Arunpan RN, Parichart Phainuphong RN. Attributable mortality of imipenem-resistant nosocomial Acinetobacter baumannii bloodstream infection. J Med Assoc Thailand. 2009;92(3):413–9. PubMed Central PMCID: PMC19301737. 19301737

[82] Kalam K, Qamar F, Kumar S, Ali S, Baqi S. Risk factors for carbapenem resistant bacteraemia and mortality due to gram negative bacteraemia in a developing country. J Pak Med Assoc. 2014;64(5):530–6. Epub 2014/10/03. . 25272538

[83] Li H, Zheng Y, Yang X, Zhang P, Xiao W, Yang M. Clinical characteristics and prognosis of carbapenem-resistant klebsiella pneumoniae infection of critical patients. Chin J Evid Based Med. 2019;19(2):129–134. doi: 10.7507/1672-2531.201809113

[84] Li L, Huang H. Risk factors of mortality in bloodstream infections caused by Klebsiella pneumonia: A single-center retrospective study in China. Medicine (Baltimore). 2017;96(35):e7924. Epub 2017/09/01. doi: 10.1097/MD.0000000000007924 ; PubMed Central PMCID: PMC5585510. 28858116 PMC5585510

[85] Li S, Jia X, Li C, Zou H, Liu H, Guo Y, et al. Carbapenem-resistant and cephalosporin-susceptible pseudomonas aeruginosa: A notable phenotype in patients with bacteremia. Infect Drug Resist. 2018;11:1225–1235. doi: 10.2147/IDR.S174876 30154669 PMC6108401

[86] Li X, Ye H. Clinical and Mortality Risk Factors in Bloodstream Infections with Carbapenem-Resistant Enterobacteriaceae. Can J Infect Dis Med Microbiol. 2017;2017:6212910. Epub 2018/01/31. doi: 10.1155/2017/6212910 ; PubMed Central PMCID: PMC5742906. 29379527 PMC5742906

[87] Li Y, Li J, Hu T, Hu J, Song N, Zhang Y, et al. Five-year change of prevalence and risk factors for infection and mortality of carbapenem-resistant Klebsiella pneumoniae bloodstream infection in a tertiary hospital in North China. Antimicrob Resist Infect Control. 2020;9(1):79. Epub 2020/06/04. doi: 10.1186/s13756-020-00728-3 ; PubMed Central PMCID: PMC7268443. 32487221 PMC7268443

[88] Lim C, Takahashi E, Hongsuwan M, Wuthiekanun V, Thamlikitkul V, Hinjoy S, et al. Epidemiology and burden of multidrug-resistant bacterial infection in a developing country. eLife. 2016;5(September). doi: 10.7554/eLife.18082 PubMed Central PMCID: PMC27599374. 27599374 PMC5030096

[89] Lima EM, Cid PA, Beck DS, Pinheiro LHZ, Tonhá JPS, Alves MZO, et al. Predictive factors for sepsis by carbapenem resistant Gram-negative bacilli in adult critical patients in Rio de Janeiro: A case-case-control design in a prospective cohort study. Antimicrob Resist Infect Control. 2020;9(1). doi: 10.1186/s13756-020-00791-w PubMed Central PMCID: PMC32795380. 32795380 PMC7426895

[90] Lipari FG, Hernández D, Vilaró M, Caeiro JP, Saka HA. Clinical, epidemiological and microbiological characterization of bacteremia produced by carbapenem-resistant enterobacteria in a university hospital in Córdoba, Argentina. Rev Chil Infectol. 2020;37(4):362–70. doi: 10.4067/S0716-10182020000400362 PubMed Central PMCID: PMC33399656. 33399656

[91] Liu J, Wang H, Huang Z, Tao X, Li J, Hu Y, et al. Risk factors and outcomes for carbapenem-resistant Klebsiella pneumoniae bacteremia in onco-hematological patients. J Infect Dev Ctries. 2019;13(5):357–64. Epub 2020/02/14. doi: 10.3855/jidc.11189 . 32053504

[92] Liu Q, Li W, Du X, Li W, Zhong T, Tang Y, et al. Risk and prognostic factors for multidrug-resistant Acinetobacter baumannii complex bacteremia: A retrospective study in a tertiary hospital of West China. PLoS ONE. 2015;10(6). doi: 10.1371/journal.pone.0130701 PubMed Central PMCID: PMC26083415. 26083415 PMC4471170

[93] Liu Q, Wu J, Wang Z, Wu X, Wang G, Ren J. Polymicrobial Bacteremia Involving Klebsiella pneumoniae in Patients with Complicated Intra-Abdominal Infections: Frequency, Co-Pathogens, Risk Factors, and Clinical Outcomes. Surg Infect (Larchmt). 2019;20(4):317–25. Epub 2019/02/09. doi: 10.1089/sur.2018.207 . 30735082

[94] Liu Y, Wang Q, Zhao C, Chen H, Li H, Wang H, et al. Prospective multi-center evaluation on risk factors, clinical characteristics and outcomes due to carbapenem resistance in Acinetobacter baumannii complex bacteraemia: experience from the Chinese Antimicrobial Resistance Surveillance of Nosocomial Infections (CARES) Network. J Med Microbiol. 2020;69(7):949–59. Epub 2020/06/26. doi: 10.1099/jmm.0.001222 . 32584215

[95] Loftus MJ, Young-Sharma TE, Lee SJ, Wati S, Badoordeen GZ, Blakeway LV, et al. Attributable mortality and excess length of stay associated with third-generation cephalosporin-resistant Enterobacterales bloodstream infections: a prospective cohort study in Suva, Fiji. J Glob Antimicrob Resist. 2022;30:286–293. doi: 10.1016/j.jgar.2022.06.016 35738385 PMC9452645

[96] López-Luis BA, Sifuentes-Osornio J, Lambraño-Castillo D, Ortiz-Brizuela E, Ramírez-Fontes A, Tovar-Calderón YE, et al. Risk factors and outcomes associated with vancomycin-resistant Enterococcus faecium and ampicillin-resistant Enterococcus faecalis bacteraemia: A 10-year study in a tertiary-care centre in Mexico City. J Glob Antimicrob Resist. 2020;24:198–204. Epub 2020/12/29. doi: 10.1016/j.jgar.2020.12.005 . 33359937

[97] Ma J, Li N, Liu Y, Wang C, Liu X, Chen S, et al. Antimicrobial resistance patterns, clinical features, and risk factors for septic shock and death of nosocomial e coli bacteremia in adult patients with hematological disease. Medicine. 2017;96(21). doi: 10.1097/MD.0000000000006959 PubMed Central PMCID: PMC28538389. 28538389 PMC5457869

[98] Marra AR, Wey SB, Castelo A, Gales AC, Cal RG, Filho JR, et al. Nosocomial bloodstream infections caused by Klebsiella pneumoniae: impact of extended-spectrum beta-lactamase (ESBL) production on clinical outcome in a hospital with high ESBL prevalence. BMC Infect Dis. 2006;6:24. Epub 2006/02/16. doi: 10.1186/1471-2334-6-24 ; PubMed Central PMCID: PMC1382232. 16478537 PMC1382232

[99] Menekşe Ş, Çağ Y, Işık ME, Şahin S, Hacıseyitoğlu D, Can F, et al. The effect of colistin resistance and other predictors on fatality among patients with bloodstream infections due to Klebsiella pneumoniae in an OXA-48 dominant region. Int J Infect Dis. 2019;86:208–11. doi: 10.1016/j.ijid.2019.06.008 PubMed Central PMCID: PMC31402295. 31402295

[100] Metan G, Sariguzel F, Sumerkan B. Factors influencing survival in patients with multi-drug-resistant Acinetobacter bacteraemia. Eur J Intern Med. 2009;20(5):540–4. doi: 10.1016/j.ejim.2009.05.005 PubMed Central PMCID: PMC19712862. 19712862

[101] Moghnieh R, Estaitieh N, Mugharbil A, Jisr T, Abdallah DI, Ziade F, et al. Third generation cephalosporin resistant Enterobacteriaceae and multidrug resistant gram-negative bacteria causing bacteremia in febrile neutropenia adult cancer patients in Lebanon, broad spectrum antibiotics use as a major risk factor, and correlation with poor prognosis. Front Cell Infect Microbiol. 2015;5(FEB). doi: 10.3389/fcimb.2015.00011 25729741 PMC4325930

[102] Moreira M, Medeiros EA, Pignatari AC, Wey SB, Cardo DM. [Effect of nosocomial bacteremia caused by oxacillin-resistant Staphylococcus aureus on mortality and length of hospitalization]. Rev Assoc Med Bras (1992). 1998;44(4):263–8. Epub 1998/12/16. doi: 10.1590/s0104-42301998000400002 . 9852643

[103] Najmi A, Karimi F, Kunhikatta V, Varma M, Nair S. Resistance Trend, Antibiotic Utilization and Mortality in Patients with E. coli Bacteraemia. Open Access Maced J Med Sci. 2019;7(7):1119–23. Epub 2019/05/03. doi: 10.3889/oamjms.2019.223 ; PubMed Central PMCID: PMC6490482. 31049092 PMC6490482

[104] Niu T, Xiao T, Guo L, Yu W, Chen Y, Zheng B, et al. Retrospective comparative analysis of risk factors and outcomes in patients with carbapenem-resistant Acinetobacter baumannii bloodstream infections: Cefoperazone–sulbactam associated with resistance and tigecycline increased the mortality. Infect Drug Resist. 2018;11:2021–2030. doi: 10.2147/IDR.S169432 30464544 PMC6208797

[105] Palavutitotai N, Jitmuang A, Tongsai S, Kiratisin P, Angkasekwinai N. Epidemiology and risk factors of extensively drug-resistant Pseudomonas aeruginosa infections. PLoS ONE. 2018;13(2). doi: 10.1371/journal.pone.0193431 PubMed Central PMCID: PMC29470531. 29470531 PMC5823452

[106] Porto JP, Santos RO, Gontijo Filho PP, Ribas RM. Active surveillance to determine the impact of methicillin resistance on mortality in patients with bacteremia and influences of the use of antibiotics on the development of MRSA infection. Rev Soc Bras Med Trop. 2013;46(6):713–8. Epub 2014/01/30. doi: 10.1590/0037-8682-0199-2013 . 24474012

[107] Rao C, Dhawan B, Vishnubhatla S, Kapil A, Das B, Sood S. Clinical and molecular epidemiology of vancomycin-resistant Enterococcus faecium bacteremia from an Indian tertiary hospital. Eur J Clin Microbiol Infect Dis. 2021;40(2):303–14. doi: 10.1007/s10096-020-04030-3 PubMed Central PMCID: PMC32909085. 32909085

[108] Seboxa T, Amogne W, Abebe W, Tsegaye T, Azazh A, Hailu W, et al. High Mortality from Blood Stream Infection in Addis Ababa, Ethiopia, Is Due to Antimicrobial Resistance. PLoS ONE. 2015;10(12):e0144944. Epub 2015/12/17. doi: 10.1371/journal.pone.0144944 ; PubMed Central PMCID: PMC4682922. 26670718 PMC4682922

[109] Serefhanoglu K, Turan H, Timurkaynak FE, Arslan H. Bloodstream infections caused by ESBL-producing E. coli and K. pneumoniae: Risk factors for multidrug-resistance. Braz J Infect Dis. 2009;13(6):403–7. doi: 10.1590/s1413-86702009000600003 PubMed Central PMCID: PMC20464329. 20464329

[110] Shi SH, Kong HS, Xu J, Zhang WJ, Jia CK, Wang WL, et al. Multidrug resistant gram-negative bacilli as predominant bacteremic pathogens in liver transplant recipients. Transplant Infect Dis. 2009;11(5):405–12. doi: 10.1111/j.1399-3062.2009.00421.x PubMed Central PMCID: PMC19638006. 19638006

[111] Shi N, Kang J, Wang S, Song Y, Yin D, Li X, et al. Bacteriological Profile and Antimicrobial Susceptibility Patterns of Gram-Negative Bloodstream Infection and Risk Factors Associated with Mortality and Drug Resistance: A Retrospective Study from Shanxi, China. Infect Drug Resist. 2022;15:3561. doi: 10.2147/IDR.S370326 35833010 PMC9271686

[112] Sirijatuphat R, Sripanidkulchai K, Boonyasiri A, Rattanaumpawan P, Supapueng O, Kiratisin P, et al. Implementation of global antimicrobial resistance surveillance system (GLASS) in patients with bacteremia. PLoS ONE. 2018;13(1). doi: 10.1371/journal.pone.0190132 PubMed Central PMCID: PMC29298323. 29298323 PMC5752004

[113] de Moraes LS, Magalhaes GLG, Soncini JGM, Pelisson M, Perugini MRE, Vespero EC. High mortality from carbapenem-resistant Klebsiella pneumoniae bloodstream infection. Microb Pathog. 2022;167:105519. doi: 10.1016/j.micpath.2022.105519 35483557

[114] Steinhaus N, Al-Talib M, Ive P, Boyles T, Bamford C, Davies MA, et al. The management and outcomes of Staphylococcus aureus bacteraemia at a South African referral hospital: A prospective observational study. Int J Infect Dis. 2018;73:78–84. Epub 2018/06/17. doi: 10.1016/j.ijid.2018.06.004 . 29908251

[115] Stewardson AJ, Marimuthu K, Sengupta S, Allignol A, El-Bouseary M, Carvalho MJ, et al. Effect of carbapenem resistance on outcomes of bloodstream infection caused by Enterobacteriaceae in low-income and middle-income countries (PANORAMA): a multinational prospective cohort study. Lancet Infect Dis. 2019;19(6):601–10. Epub 2019/05/03. doi: 10.1016/S1473-3099(18)30792-8 . 31047852

[116] Stoma I, Karpov I, Milanovich N, Uss A, Iskrov I. Risk factors for mortality in patients with bloodstream infections during the pre-engraftment period after hematopoietic stem cell transplantation. Blood Res. 2016;51(2):102–6. Epub 2016/07/07. doi: 10.5045/br.2016.51.2.102 ; PubMed Central PMCID: PMC4931927. 27382554 PMC4931927

[117] Tang Y, Xu C, Xiao H, Wang L, Cheng Q, Li X. Gram-negative bacteria bloodstream infections in patients with hematological malignancies–the impact of pathogen type and patterns of antibiotic resistance: a Retrospective Cohort Study. Infect Drug Resist. 2021;14:3115. doi: 10.2147/IDR.S322812 34413656 PMC8370111

[118] Tian L, Tan R, Chen Y, Sun J, Liu J, Qu H, et al. Epidemiology of Klebsiella pneumoniae bloodstream infections in a teaching hospital: factors related to the carbapenem resistance and patient mortality. Antimicrob Resist Infect Control. 2016;5:48. Epub 2016/11/29. doi: 10.1186/s13756-016-0145-0 ; PubMed Central PMCID: PMC5114729. 27891222 PMC5114729

[119] Topeli A, Unal S, Akalin HE. Risk factors influencing clinical outcome in Staphylococcus aureus bacteraemia in a Turkish University Hospital. Int J Antimicrob Agents. 2000;14(1):57–63. Epub 2000/03/16. doi: 10.1016/s0924-8579(99)00147-8 . 10717502

[120] Traverso F, Peluffo M, Louge M, Funaro F, Suasnabar R, Cepeda R. [Impact of methicillin resistance on mortality and surveillance of vancomycin susceptibility in bacteremias caused by Staphylococcus aureus]. Rev Argent Microbiol. 2010;42(4):274–8. Epub 2011/01/14. doi: 10.1590/s0325-75412010000400007 . 21229197

[121] Tu B, Bi J, Wu D, Zhao P, Shi L, Xie Y, et al. Bloodstream infection due to Escherichia coli in liver cirrhosis patients: clinical features and outcomes. Oncotarget. 2018;9(87):35780–9. Epub 2018/12/06. doi: 10.18632/oncotarget.23200 ; PubMed Central PMCID: PMC6254670. 30515269 PMC6254670

[122] Tuon FF, Gortz LW, Rocha JL. Risk factors for pan-resistant Pseudomonas aeruginosa bacteremia and the adequacy of antibiotic therapy. Braz J Infect Dis. 2012;16(4):351–6. doi: 10.1016/j.bjid.2012.06.009 PubMed Central PMCID: PMC22846123. 22846123

[123] Valderrama SL, González PF, Caro MA, Ardila N, Ariza B, Gil F, et al. Risk factors for hospital-acquired bacteremia due to carbapenem-resistant Pseudomonas aeruginosa in a Colombian hospital. Biomedica. 2016;36:69–77. doi: 10.7705/biomedica.v36i2.2784 PubMed Central PMCID: PMC27622627. 27622627

[124] Wang Q, Zhang Y, Yao X, Xian H, Liu Y, Li H, et al. Risk factors and clinical outcomes for carbapenem-resistant Enterobacteriaceae nosocomial infections. Eur J Clin Microbiol Infect Dis. 2016;35(10):1679–89. Epub 2016/07/13. doi: 10.1007/s10096-016-2710-0 . 27401905

[125] Wang Z, Qin RR, Huang L, Sun LY. Risk Factors for Carbapenem-resistant Klebsiella pneumoniae Infection and Mortality of Klebsiella pneumoniae Infection. Chin Med J (Engl). 2018;131(1):56–62. Epub 2017/12/23. doi: 10.4103/0366-6999.221267 ; PubMed Central PMCID: PMC5754959. 29271381 PMC5754959

[126] Wei J, Zhu QL, Sun Z, Wang C. [The impact of carbapenem-resistance Pseudomonas aeruginosa infections on mortality of patients with hematological disorders]. Zhonghua Nei Ke Za Zhi. 2020;59(5):353–9. Epub 2020/05/07. doi: 10.3760/cma.j.cn112138-20191104-00728 . 32370463

[127] Wu X, Shi Q, Shen S, Huang C, Wu H. Clinical and bacterial characteristics of Klebsiella pneumoniae affecting 30-day mortality in patients with bloodstream infection. Front Cell Infect Microbiol. 2021;11. doi: 10.3389/fcimb.2021.688989 34604103 PMC8482843

[128] Xiao T, Yu W, Niu T, Huang C, Xiao Y. A retrospective, comparative analysis of risk factors and outcomes in carbapenem-susceptible and carbapenem-nonsusceptible Klebsiella pneumoniae bloodstream infections: tigecycline significantly increases the mortality. Infect Drug Resist. 2018;11:595–606. Epub 2018/05/08. doi: 10.2147/IDR.S153246 ; PubMed Central PMCID: PMC5926074. 29731648 PMC5926074

[129] Xiao T, Zhu Y, Zhang S, Wang Y, Shen P, Zhou Y, et al. A Retrospective Analysis of Risk Factors and Outcomes of Carbapenem-Resistant Klebsiella pneumoniae Bacteremia in Nontransplant Patients. J Infect Dis. 2020;221(Suppl 2):S174–s83. Epub 2020/03/17. doi: 10.1093/infdis/jiz559 . 32176799

[130] Xie Y, Tu B, Zhang X, Bi J, Shi L, Zhao P, et al. Investigation on outcomes and bacterial distributions of liver cirrhosis patients with gram-negative bacterial bloodstream infection. Oncotarget. 2018;9(3):3980–95. Epub 2018/02/10. doi: 10.18632/oncotarget.23582 ; PubMed Central PMCID: PMC5790516. 29423099 PMC5790516

[131] Xu X, Wu S, Xie Y, Chen Z, Ma Y, He C, et al. Risk factors of bloodstream infections caused by vancomycin-resistant Enterococcus. Chin J Infect Chemother. 2015;15(5):447–451.

[132] Yang S, Sun J, Wu X, Zhang L. Determinants of Mortality in Patients with Nosocomial Acinetobacter baumannii Bacteremia in Southwest China: A Five-Year Case-Control Study. Can J Infect Dis Med Microbiol. 2018;2018:3150965. Epub 2018/07/06. doi: 10.1155/2018/3150965 ; PubMed Central PMCID: PMC6008754. 29973964 PMC6008754

[133] Yang K, Xiao T, Shi Q, Zhu Y, Ye J, Zhou Y, et al. Socioeconomic burden of bloodstream infections caused by carbapenem-resistant and carbapenem-susceptible Pseudomonas aeruginosa in China. J Glob Antimicrob Resist. 2021;26:101–107. doi: 10.1016/j.jgar.2021.03.032 34023532

[134] Ye QF, Zhao J, Wan QQ, Qiao BB, Zhou JD. Frequency and clinical outcomes of ESKAPE bacteremia in solid organ transplantation and the risk factors for mortality. Transpl Infect Dis. 2014;16(5):767–74. Epub 2014/08/16. doi: 10.1111/tid.12278 . 25124187

[135] Yilmaz M, Elaldi N, Balkan İ, Arslan F, Batırel AA, Bakıcı MZ, et al. Mortality predictors of Staphylococcus aureus bacteremia: a prospective multicenter study. Ann Clin Microbiol Antimicrob. 2016;15:7. Epub 2016/02/11. doi: 10.1186/s12941-016-0122-8 ; PubMed Central PMCID: PMC4748515. 26860463 PMC4748515

[136] Yuan Y, Wang J, Yao Z, Ma B, Li Y, Yan W, et al. Risk Factors for Carbapenem-Resistant Klebsiella pneumoniae Bloodstream Infections and Outcomes. Infect Drug Resist. 2020;13:207–15. Epub 2020/03/12. doi: 10.2147/IDR.S223243 ; PubMed Central PMCID: PMC6985980. 32158236 PMC6985980

[137] Zhang G, Zhang M, Sun F, Zhou J, Wang Y, Zhu D, et al. Epidemiology, mortality and risk factors for patients with K. pneumoniae bloodstream infections: Clinical impact of carbapenem resistance in a tertiary university teaching hospital of Beijing. J Infect Public Health. 2020;13(11):1710–4. Epub 2020/10/22. doi: 10.1016/j.jiph.2020.09.012 . 33082112

[138] Zhang Q, Gao HY, Li D, Li Z, Qi SS, Zheng S, et al. Clinical outcome of Escherichia coli bloodstream infection in cancer patients with/without biofilm formation: a single-center retrospective study. Infect Drug Resist. 2019;12:359–71. Epub 2019/02/28. doi: 10.2147/IDR.S192072 ; PubMed Central PMCID: PMC6377049. 30809097 PMC6377049

[139] Zhang Q, Zhang W, Li Z, Bai C, Li D, Zheng S, et al. Bacteraemia due to AmpC β-lactamase-producing Escherichia coli in hospitalized cancer patients: risk factors, antibiotic therapy, and outcomes. Diagn Microbiol Infect Dis. 2017;88(3):247–51. Epub 2017/04/25. doi: 10.1016/j.diagmicrobio.2017.04.006 . 28434898

[140] Zhang Y, Du M, Chang Y, Chen LA, Zhang Q. Incidence, clinical characteristics, and outcomes of nosocomial Enterococcus spp. bloodstream infections in a tertiary-care hospital in Beijing, China: a four-year retrospective study. Antimicrob Resist Infect Control. 2017;6:73. Epub 2017/07/07. doi: 10.1186/s13756-017-0231-y ; PubMed Central PMCID: PMC5496248. 28680588 PMC5496248

[141] Zhang Y, Li Y, Zeng J, Chang Y, Han S, Zhao J, et al. Risk Factors for Mortality of Inpatients with Pseudomonas aeruginosa Bacteremia in China: Impact of Resistance Profile in the Mortality. Infect Drug Resist. 2020;13:4115–23. Epub 2020/11/20. doi: 10.2147/IDR.S268744 ; PubMed Central PMCID: PMC7669529. 33209041 PMC7669529

[142] Zhao S, Wu Y, Dai Z, Chen Y, Zhou X, Zhao J. Risk factors for antibiotic resistance and mortality in patients with bloodstream infection of Escherichia coli. Eur J Clin Microbiol Infect Dis. 2022;41(5):713–721. doi: 10.1007/s10096-022-04423-6 35190911

[143] Zhao Y, Lin Q, Liu L, Ma R, Chen J, Shen Y, et al. Risk Factors and Outcomes of Antibiotic-resistant Pseudomonas aeruginosa Bloodstream Infection in Adult Patients With Acute Leukemia. Clin Infect Dis. 2020;71(Supplement_4):S386–s93. Epub 2020/12/29. doi: 10.1093/cid/ciaa1522 . 33367574

[144] Zheng SH, Cao SJ, Xu H, Feng D, Wan LP, Wang GJ, et al. Risk factors, outcomes and genotypes of carbapenem-nonsusceptible Klebsiella pneumoniae bloodstream infection: a three-year retrospective study in a large tertiary hospital in Northern China. Infect Dis (Lond). 2018;50(6):443–51. Epub 2018/01/06. doi: 10.1080/23744235.2017.1421772 . 29303020

[145] Zheng X, Wang JF, Xu WL, Xu J, Hu J. Clinical and molecular characteristics, risk factors and outcomes of Carbapenem-resistant Klebsiella pneumoniae bloodstream infections in the intensive care unit. Antimicrob Resist Infect Control. 2017;6:102. Epub 2017/10/14. doi: 10.1186/s13756-017-0256-2 ; PubMed Central PMCID: PMC5625719 Institutional Review Board of the First Affiliated Hospital, College of Medicine, Zhejiang University. This research was conducted in compliance with the tenets of the Helsinki Declaration. CONSENT FOR PUBLICATION: Not applicable. COMPETING INTERESTS: The authors declare that they have no competing interests. PUBLISHER’S NOTE: Springer Nature remains neutral with regard to jurisdictional claims in published maps and institutional affiliations. 29026535 PMC5625719

[146] Zhou H, Yao Y, Zhu B, Ren D, Yang Q, Fu Y, et al. Risk factors for acquisition and mortality of multidrug-resistant Acinetobacter baumannii bacteremia: A retrospective study from a Chinese hospital. Medicine (Baltimore). 2019;98(13):e14937. Epub 2019/03/29. doi: 10.1097/MD.0000000000014937 ; PubMed Central PMCID: PMC6456023. 30921191 PMC6456023

[147] Zhu C, Liu C, Wu B, Wu Q, Huang D. Analysis of antibiotic resistance in the staphylococcus aureus strains isolated from bloodstream infections and associated patient outcome. Chin J Infect Chemother. 2016;16 (1):1–4. doi: 10.16718/j.1009-7708.2016.01.001

[148] Zhu Y, Xiao T, Wang Y, Yang K, Zhou Y, Luo Q, et al. Socioeconomic Burden of Bloodstream Infections Caused by Carbapenem-Resistant Enterobacteriaceae. Infect Drug Resist. 2021;14:5385. doi: 10.2147/IDR.S341664 34938086 PMC8685763

[149] Zlatian O, Balasoiu AT, Balasoiu M, Cristea O, Docea AO, Mitrut R, et al. Antimicrobial resistance in bacterial pathogens among hospitalised patients with severe invasive infections. Exp Ther Med. 2018;16(6):4499–4510. doi: 10.3892/etm.2018.6737 30542398 PMC6257814

[150] Zou XL, Feng DY, Wu WB, Yang HL, Zhang TT. Blood urea nitrogen to serum albumin ratio independently predicts 30-day mortality and severity in patients with Escherichia coli bacteraemia. Med Clin (Barc). 2020. Epub 2020/10/17. doi: 10.1016/j.medcli.2020.06.060 . 33059940

[151] Zhang WL, Huang J, Wu SY, Liu Y, Long F, Xiao YL, et al. [Antibiotic Resistance and Risk Factors for Mortality of Blood Stream Infections (BSIs) with Escherichia coli in Patients with Hematological Malignancies]. Sichuan Da Xue Xue Bao Yi Xue Ban. 2018;49(1):133–5. Epub 2018/05/08. . 29737104

[152] Zhang Y, Zhu W, Zhang J, Chen B. The risk factors associated with bloodstream infections caused by multi-drug resistant acinetobacter baumannii. Chin J Infect Chemother. 2017;17(2):134–139. doi: 10.16718/j.1009-7708.2017.02.003

[153] Jit M, Ng DHL, Luangasanatip N, Sandmann F, Atkins KE, Robotham JV, et al. Quantifying the economic cost of antibiotic resistance and the impact of related interventions: rapid methodological review, conceptual framework and recommendations for future studies. BMC Med. 2020;18(1):1–14. 32138748 10.1186/s12916-020-1507-2 PMC7059710

[154] Murray CJ, Ikuta KS, Sharara F, Swetschinski L, Aguilar GR, Gray A, et al. Global burden of bacterial antimicrobial resistance in 2019: a systematic analysis. Lancet. 2022. 10.1016/S0140-6736(21)02724-0 PMC8841637 35065702

[155] Zhang Y, Chen X-L, Huang A-W, Liu S-L, Liu W-J, Zhang N, et al. Mortality attributable to carbapenem-resistant Pseudomonas aeruginosa bacteremia: a meta-analysis of cohort studies. Emerg Microbes Infect. 2016;5(1):1–6. doi: 10.1038/emi.2016.22 27004762 PMC4820673

[156] Paul M, Weinberger M, Siegman-Igra Y, Lazarovitch T, Ostfeld I, Boldur I, et al. Acinetobacter baumannii: emergence and spread in Israeli hospitals 1997–2002. J Hosp Infect. 2005;60(3):256–260. doi: 10.1016/j.jhin.2005.01.007 15893851 PMC7114673

[157] Chopra T, Marchaim D, Awali RA, Krishna A, Johnson P, Tansek R, et al. Epidemiology of bloodstream infections caused by Acinetobacter baumannii and impact of drug resistance to both carbapenems and ampicillin-sulbactam on clinical outcomes. Antimicrob Agents Chemother. 2013;57(12):6270–6275. doi: 10.1128/AAC.01520-13 24100492 PMC3837851

[158] Barrasa-Villar JI, Aibar-Remón C, Prieto-Andrés P, Mareca-Doñate R, Moliner-Lahoz J. Impact on morbidity, mortality, and length of stay of hospital-acquired infections by resistant microorganisms. Clin Infect Dis. 2017. doi: 10.1093/cid/cix411 28472416

[159] Cosgrove SE. The relationship between antimicrobial resistance and patient outcomes: mortality, length of hospital stay, and health care costs. Clin Infect Dis. 2006;42(Supplement_2):S82–S89. doi: 10.1086/499406 16355321

[160] Tsuzuki S, Yu J, Matsunaga N, Ohmagari N. Length of stay, hospitalisation costs and in-hospital mortality of methicillin-susceptible and methicillin-resistant Staphylococcus aureus bacteremia in Japan. Public Health. 2021;198:292–296. doi: 10.1016/j.puhe.2021.07.046 34507134

[161] Graffunder EM, Venezia RA. Risk factors associated with nosocomial methicillin-resistant Staphylococcus aureus (MRSA) infection including previous use of antimicrobials. J Antimicrob Chemother. 2002;49(6):999–1005. doi: 10.1093/jac/dkf009 12039892

[162] Ben-David D, Kordevani R, Keller N, Tal I, Marzel A, Gal-Mor O, et al. Outcome of carbapenem resistant Klebsiella pneumoniae bloodstream infections. Clin Microbiol Infect. 2012;18(1):54–60. doi: 10.1111/j.1469-0691.2011.03478.x 21722257

[163] Van Boeckel TP, Gandra S, Ashok A, Caudron Q, Grenfell BT, Levin SA, et al. Global antibiotic consumption 2000 to 2010: an analysis of national pharmaceutical sales data. Lancet Infect Dis. 2014;14(8):742–750. doi: 10.1016/S1473-3099(14)70780-7 25022435

[164] Klein EY, Van Boeckel TP, Martinez EM, Pant S, Gandra S, Levin SA, et al. Global increase and geographic convergence in antibiotic consumption between 2000 and 2015. Proc Natl Acad Sci U S A. 2018;115(15):E3463–E3470. doi: 10.1073/pnas.1717295115 29581252 PMC5899442

[165] Qu J, Huang Y, Lv X. Crisis of antimicrobial resistance in China: now and the future. Front Microbiol. 2019;10:2240. doi: 10.3389/fmicb.2019.02240 31611863 PMC6777638

[166] Gulen TA, Guner R, Celikbilek N, Keske S, Tasyaran M. Clinical importance and cost of bacteremia caused by nosocomial multi drug resistant Acinetobacter baumannii. Int J Infect Dis. 2015;38:32–35. doi: 10.1016/j.ijid.2015.06.014 26129972

[167] Huang W, Qiao F, Zhang Y, Huang J, Deng Y, Li J, et al. In-hospital medical costs of infections caused by carbapenem-resistant Klebsiella pneumoniae. Clin Infect Dis. 2018;67(suppl_2):S225–S230. doi: 10.1093/cid/ciy642 30423052

[168] World Health Organization. Sustainable Development Goals (SDGs) AMR indicator 2022 [cited 2022 Mar 29]. Available from: https://www.who.int/data/gho/data/themes/topics/global-antimicrobial-resistance-surveillance-system-glass/sustainable-development-goals-amr-indicator.

[169] MAAP: Mapping AMR and AMU partnership. Incomplete antimicrobial resistance (AMR) data in Africa: The crisis within the crisis. 2022.

[170] de Kraker ME, Lipsitch M. Burden of antimicrobial resistance: compared to what? Epidemiol Rev. 2021;43(1):53–64. 10.1093/epirev/mxab001 PMC8763122 33710259

[171] Pezzani MD, Tornimbene B, Pessoa-Silva C, de Kraker M, Rizzardo S, Salerno ND, et al. Methodological quality of studies evaluating the burden of drug-resistant infections in humans due to the WHO Global Antimicrobial Resistance Surveillance System target bacteria. Clin Microbiol Infect. 2021;27(5):687–696. doi: 10.1016/j.cmi.2021.01.004 33450389 PMC8113024

[172] De Angelis G, Murthy A, Beyersmann J, Harbarth S. Estimating the impact of healthcare-associated infections on length of stay and costs. Clin Microbiol Infect. 2010;16(12):1729–1735. doi: 10.1111/j.1469-0691.2010.03332.x 20673257

