# Supplementary material for: The impact of inpatient bloodstream infections caused by antibiotic-resistant bacteria in low- and middle-income countries: A systematic review and meta-analysis
Source: PLoS Med. 2023 Jun 22;20(6):e1004199. doi: 10.1371/journal.pmed.1004199 (PMC10287017; doi:10.1371/journal.pmed.1004199)
Supplement: S1 Text — Text A. Search criteria used by search engine. Table A. Studies inclusion and exclusion criteria. Table B. Years of the studies included. Table C. Number of studies included by WHO region and WB income group. Table D. Correlation between main outcomes and demographic variables. Table E. Most prevalent bacterium family, Gram type, resistance type, and antibiotic-bacterium pair by country among the included studies. Table F. Descriptive statistics of the studies included in the meta-analysis. Table G. Summary of the subgroup meta-analysis results for income level and WHO region by outcome variable. Table H. Costs of hospital bed-day per patient and by country and hospital level (in 2008 USDs). Table I. Costs of total excess hospital bed-days per patient by country and hospital level using estimated SMD and their respective 95% CIs (in 2008 USDs). Table J. Costs of total excess hospital bed-days per patient and by country and hospital level using estimated SMD and their respective 95% CIs (inflated to 2020 USDs). Table K. Calculation of YPLL, YPPLL, and CPL, by country. Table L. Total productivity losses due to premature mortality costs by country using the LE at the age of death and productivity cost approach (age of retirement), discounted. Table M. Intensive care unit costs per patient (daily). Table N. Intensive care unit costs (per patient and daily) adjusted to 2020 USDs (inflated accordingly). Table O. Intensive care unit costs (per day/patient) adjusted to ICU LOS and reported in 2020 USDs (inflated accordingly). Table P. Total excess costs incurred for bloodstream infections caused by antibiotic-resistant bacteria, per patient. Table Q. Statistics calculated for meta-analysis using mortality as an outcome, by model. Table R. Statistics calculated for meta-analysis using ICU admission as an outcome, by model. Table S. Statistics calculated for meta-analysis using the length of stay at hospital as an outcome, by model. Table T. Summary of the subgroup meta-analy [file pmed.1004199.s001.pdf]

Supplementary Material

The impact of inpatient bloodstream infections caused by antibiotic-resistant bacteria in low- and middle-income countries: A systematic review and meta-analysis

Kasim Allel, Jennifer Stone, Eduardo A. Undurraga, Lucy Day, Catrin E. Moore, Leesa Lin, Luis Furuya-Kanamori, Laith Yakob

Table of Contents

(1) SEARCH CRITERIA USED BY SEARCH ENGINE ... 2

(2) ADDITIONAL DESCRIPTIVE STATISTICS FOR SAMPLED COUNTRIES ... 5

(3) META-ANALYSIS OF FULL RESULTS ... 12

3.A) MORTALITY RESULTS: GENERAL AND SUBGROUP META-ANALYSES ... 13
3.B) LENGTH OF HOSPITAL STAY (LOS) RESULTS: GENERAL AND SUBGROUP META-ANALYSES ... 18
3.C) ICU ADMISSION RESULTS: GENERAL AND SUBGROUP META-ANALYSES ... 23
3.D) ODDS RATIOS UNADJUSTED AND ADJUSTED FOR MORTALITY RATES ... 35

(4) COSTS PER HOSPITAL BED-DAY, MORTALITY, ICU ADMISSION, AND TOTAL COSTS PER PATIENT INCURRED ... 45

(4.1) COSTS PER HOSPITAL BED-DAY STAY PER PATIENT ACCORDING TO THE WHO-CHOICE ... 45
(4.2) MORTALITY COSTS ... 48
(4.3) INTENSIVE CARE UNIT (ICU) COSTS PER DAY AND PATIENT ... 51
(4.4) TOTAL EXCESS COSTS INCURRED PER PATIENT ... 54

(5) META-ANALYSIS STATISTICS, BY OUTCOME AND MODEL TYPE (SUBGROUP) ... 55

(6) INFLUENCE ANALYSES: META-ANALYSES ... 60

(7) ADDITIONAL ANALYSES ... 63

(7.A) META-ANALYSIS RESULTS DISSAGREGATED BY PRIORITISED ANTIBIOTIC-BACTERIUM SPECIFIC PATHOGENS ACCORDING TO THE WHO ... 63
(7.B) META-ANALYSIS RESULTS DISSAGREGATED BY STUDIES' LOCATION INCLUDING CHINA AND OTHER THAN CHINA ... 70
(7.C) META-REGRESSION USING INDEPENDENT VARIABLES, BY OUTCOME ... 74

(8) RISK OF BIAS ASSESSMENT ... 78

(9) META-ANALYSIS GENERAL RESULTS ADJUSTED BY ARTICLES'S RISK OF BIAS ASSESSMENT ... 81

(10) GUIDELINES FOR ACCURATE AND TRANSPARENT HEALTH ESTIMATES REPORTING (GATHER) ... 83

(11) PRISMA CHECKLIST ... 85

(12) ADDITIONAL REFERENCES ... 89

Notes: S1 Data presents the complete information on the studies, data used and the risk of bias assessment by the study is separately attached.

(1) Search criteria used by search engine

Text A. Search criteria used by search engine

A) Search criteria → PubMED (N=4,720):

("multidrug-resistan*"[Title] OR "drug-resistan*"[Title] OR "antimicrobial-resistan*"[Title] OR "antibiotic-resistan*"[Title] OR "resistan*"[Title/Abstract] OR “nonsusceptib*”[Title/Abstract]) AND ("bacteremia*"[Title] OR "bacteraemia*"[Title] OR “sepsis”[Title] OR "infection*"[Title/Abstract] OR "bloodstream*"[Title] OR "bacteremic*"[Title] OR "bacteraemic*"[Title] OR “blood stream”[Title]) AND ("cost*"[Title] OR "burden*"[Title] OR "economic*"[Title] OR "mortalit*"[Title] OR "morbidit*"[Title] OR “length of stay”[Title] OR “duration of stay”[Title] OR “productivity”[Title] OR “outcome*”[Title] OR “impact*”[Title] OR “excess*”[Title]) AND ("hospital"[Title/Abstract] OR "inpatient*"[Title/Abstract] OR "intensive care unit*"[Title/Abstract] OR "icu"[Title/Abstract] OR "critical care*"[Title/Abstract] OR "in patient*"[Title/Abstract] OR “patient*”[Title/Abstract] OR “nosocomial*”[Title/Abstract] OR “ICU*”[Title/Abstract] OR “intensive care”[Title/Abstract] OR “clinic*”[Title/Abstract]) NOT ("animals"[MeSH Terms] OR "plants"[MeSH Terms] NOT "humans"[MeSH Terms]) NOT ("infant, newborn"[MeSH Terms] OR "infant"[Title/Abstract] OR "newborn"[Title/Abstract] OR "newborn infant"[Title/Abstract] OR "neonatal"[Title/Abstract] OR "neonate"[Title/Abstract] OR "neonates"[Title/Abstract] OR "neonatality"[Title/Abstract] OR "neonatals"[Title/Abstract] OR "neonate s"[Title/Abstract] OR "neonate*"[Title/Abstract]) NOT (“HIV”[Title] OR “Fungus”[Title/Abstract] OR “Virus*”[Title/Abstract] OR “Malaria”[Title/Abstract] OR “SARS-CoV-2”[Title/Abstract] OR “COVID-19”[Title/Abstract] OR “Candida”[Title/Abstract])

B) Search criteria → SCIELO (N=55):

("multidrug-resistance" OR “multidrug-resistant” OR "drug-resistant" OR "drug-resistance" OR "antimicrobial-resistant" OR "antimicrobial-resistance" OR "antibiotic-resistant" OR "antibiotic-resistance" OR “nonsusceptible” OR “resistant” OR “resistance”) AND ("cost" OR “costs” OR "burden" OR "economic" OR “economic” OR "mortality" OR "morbidity" OR “morbidities” OR “length of stay” OR “duration of stay” OR “productivity” OR “outcome” OR “outcomes” OR “impact” OR “impacts” OR “excess”)

C) Search criteria → SCOPUS (N=8,193):

TITLE ("multidrug-resistance" OR "multidrug-resistant" OR "drug-resistant" OR "drug-resistance" OR "antimicrobial-resistant" OR "antimicrobial-resistance" OR "antibiotic-resistant" OR "antibiotic-resistance" OR "nonsusceptible" OR "resistant" OR "resistance") AND TITLE ("cost" OR "costs" OR "burden" OR "economic" OR "economic" OR "mortalitity" OR "morbidity" OR "morbidities" OR "length of stay" OR "duration of stay" OR "productivity" OR "outcome" OR "outcomes" OR "impact" OR "impacts" OR "excess") OR ("resistant" OR "resistance") AND TITLE ("bacteremia" OR "bacteremias" OR "bacteraemia" OR "sepsis" OR "infection" OR "infections" OR "bloodstream" OR "bacteremic" OR "bacteremics" OR "bacteraemics" OR "bacteraemic" OR "blood stream") AND TITLE-ABS-KEY (("hospital" OR "inpatient*" OR "intensive care unit*" OR "icu" OR "critical care*" OR "in patient*" OR "patient*" OR "nosocomial*" OR "ICU*" OR "intensive care" OR "clinic*")) AND NOT TITLE-ABS-KEY ("animals" OR "animal" OR "plant" OR "plants" OR "infant" OR "infants" OR "newborn" OR "newborn infant" OR "neonatal" OR "neonate" OR "neonates" OR "neonatality" OR "neonatal" OR "neonates" OR "neonate*" OR "HIV" OR "Fungus" OR "Virus*" OR "Malaria" OR "SARS-CoV-2" OR "COVID-19" OR "Candida") AND (LIMIT-TO (DOCTYPE , "ar") OR LIMIT-TO (DOCTYPE , "re"))

D) Search criteria → WHO’s Global Index Medicus (Latin American and Caribbean Health Sciences Literature ‘LILACs’ and African Index Medicus ‘AIM’) (N=44):

("multidrug-resistance" OR “multidrug-resistant” OR "drug-resistant" OR "drug-resistance" OR "antimicrobial-resistant" OR "antimicrobial-resistance" OR "antibiotic-resistant" OR "antibiotic-resistance" OR “nonsusceptible” OR “resistant” OR “resistance”) AND ("cost" OR “costs” OR "burden" OR "economic" OR “economic” OR "mortalitity" OR "morbidity" OR “morbidities” OR “length of stay” OR “duration of stay” OR “productivity” OR “outcome” OR “outcomes” OR “impact” OR “impacts” OR “excess”) OR (“resistant” OR “resistance”) AND ("bacteremia" OR “bacteremias” OR "bacteraemia" OR “sepsis” OR "infection" OR

“infections” OR "bloodstream" OR "bacteremic" OR “bacteremics” OR “bacteraemics” OR "bacteraemic" OR “blood stream”)

Table A. Studies inclusion and exclusion criteria.

Criteria	Inclusion Criteria	Exclusion Criteria
**Population**	Hospitalised patients (adults, age >18 years)	Patients outside hospital, children and neonates, animals
**Geography**	All low- and middle-income countries, as defined by the 2021 World Bank classification	High-income countries
**Time**	All	None
**Setting**	Primary, secondary, or tertiary public or private hospitals	Care homes, community sites
**Interventions or comparison groups analysed**	Studies comparing patients having antibiotic-resistant bloodstream infections versus patients with antibiotic susceptible bloodstream infections. Also, participants having BSIs with chronic or severe underlying diseases were included if they were prevalent among both ARB and ASB groups.	All studies only reporting one group or comparing antibiotic-resistant bloodstream infections against all the rest patients (including the non-infection counterfactual). Studies reporting individuals having BSIs in combination with underlying conditions not clearly distributed throughout the ARB and ASB groups were withdrawn. For instance, the study was removed if HIV-positive patients with ARB BSIs were compared to HIV-negative patients with ASB BSIs.
	Bloodstream infections must be reported through the use of blood specimens	All studies without clear source of blood specimens. Also, studies which combined different cultures all together were removed (for instance, some studies analysed patients having blood, urine, swab, skin or faecal positive cultures on the whole).
	We only included those bacteria from the WHO priority list (*Acinetobacter baumannii*,	All other pathogens were removed.

	Pseudomonas aeruginosa, *Enterobacteriaceae*, *Enterococcus faecium*, *Staphylococcus aureus*, *Helicobacter pylori*, *Campylobacter spp*, *Salmonellae*, *Neisseria gonorrhoeae*, *Streptococcus pneumoniae*, *Haemophilus influenzae*, and *Shigella spp*.)	
**Outcomes**	Studies must have at least one outcome measured from the following list: mortality, Intensive care unit admission, length of hospital stay, or economic costs	All other health outcomes or studies without reporting any
**Publication language**	Any	None
**Publication Type**	Peer-reviewed published articles	Conference proceedings, correspondence letters or opinion, short reports without data-analyses, literature reviews, single case reports, magazine entries
**Study design**	Experimental and observational designs including cross-sectional studies, matched case-control studies, prospective and retrospective cohort studies, and longitudinal studies.	All other study designs (e.g.literature reviews; systematic reviews; meta-analyses, case studies)
**Time**	Any	None

ARB: Antibiotic-resistant bacteria. ASB: antibiotic-sensitive bacteria. BSI: bloodstream infections.

(2) Additional descriptive statistics for sampled countries

Table B. Years of the studies included (N=109 studies).

Year	Freq.	Percent (%)	Cum. (%)
1998	2	1.83	1.83
2000	1	0.92	2.75
2002	1	0.92	3.67
2006	3	2.75	6.42
2009	5	4.59	11.01
2010	2	1.83	12.84
2011	2	1.83	14.68
2012	3	2.75	17.43
2013	2	1.83	19.27
2014	6	5.50	24.77
2015	6	5.50	30.28
2016	12	11.01	41.28
2017	8	7.34	48.62
2018	16	14.68	63.30
2019	9	8.26	71.56
2020	17	15.60	87.16
2021	6	5.50	92.66
2022	8	7.34	100.00
Total	109	100	100

Percent stands for percentage. Cum. = cumulative.

Fig A. Density of the studies over time (N=109 studies).

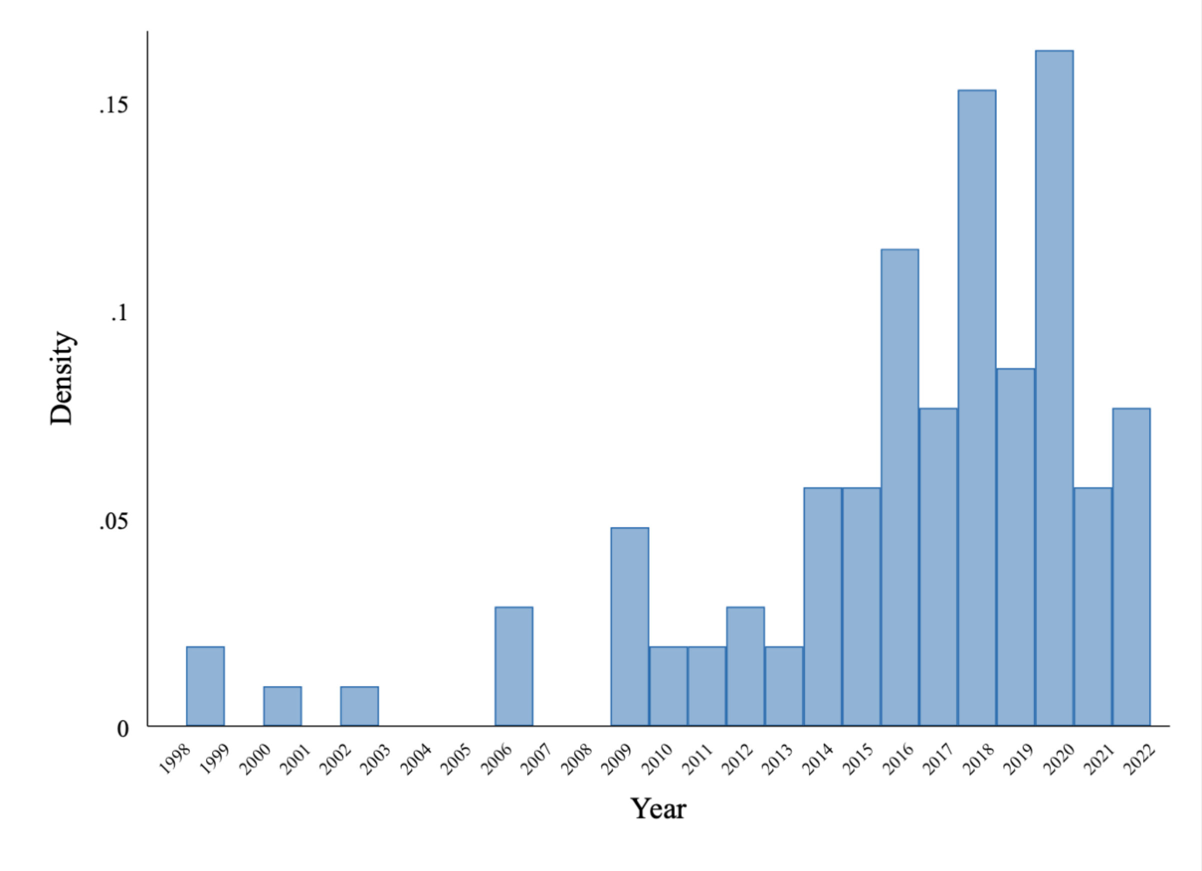

Table C. Number of studies included by WHO region and World Bank income group (N=109 studies).

WHO region	N	(%)
Multiple	1	0.92
African Region	3	2.75
Americas	25	25.94
Eastern Mediterranean Region	5	4.59
European Region	11	10.0
South-East Asian Region	13	11.93
Western Pacific Region	51	46.79

WB income group	N	(%)
Multiple	1	0.92
Low Income	1	0.92
Lower Middle Income	11	10.09
Upper Middle Income	96	88.07

The ‘Multiple’ categories stand for the joint study comprised of various countries. WB: World Bank

Table D. Correlation between primary outcomes and demographic variables.

	AMR susceptibility	Mortality	Length of stay	ICU admission	Age in years	Gender (female)
AMR susceptibility	1.000	0.575	0.274	0.520	0.021	-0.119
Mortality	0.575	1.000	0.058	0.348	-0.102	0.023
Length of stay	0.274	0.058	1.000	0.251	0.130	-0.191
ICU admission	0.520	0.348	0.251	1.000	-0.071	-0.260
Age in years	0.021	-0.102	0.130	-0.071	1.000	0.162
Gender (female)	-0.119	0.023	-0.191	-0.260	0.162	1.000

AMR denotes antimicrobial resistance. We use it as a synonym for ARB (antibiotic-resistant bacteria).

Table E. Most prevalent bacterium family, gram type, resistance type, and antibiotic-bacterium pair by country among the included studies.

Country	Gram-type	Most prevalent bacterium family	Most prevalent antibiotic-bacterium pair analysed	Total number of patients
Argentina	67% Gram-negative	Enterobacteriaceae & Staphylococcus	MRSA, CREN	517
Belarus	67% Gram-negative	Enterobacteriaceae & Staphylococcus	MRSA	135
Brazil	56% Gram-negative	Staphylococcus, Enterobacteriaceae & Pseudomonas	MRSA, CRPA	1559
China	91% Gram-negative	Enterobacteriaceae, Moraxellaceae, Pseudomonas	CRKP, CRPA & CRAB	12092
Colombia	66% Gram-positive	Staphylococcus, Pseudomonas	MRSA	2550
Ethiopia	100% Gram-negative	Enterobacteriaceae	CEREC	16
Fiji	100% Gram-negative	Enterobacteriaceae	CREN	162
India	60% Gram-negative	Enterobacteriaceae, Staphylococcus & Enterococcus	MRSA, VRE	758
Indonesia	100% Gram-negative	Moraxellaceae	CRAB	144
Iran	50% Gram-negative	Enterobacteriaceae & Enterococcus	VRE	103
Lebanon	100% Gram-positive	Multiple	Multiple	75
Malaysia	100% Gram-negative	Moraxellaceae	CRAB	56
Mexico	50% Gram-negative	Enterococcus & Enterobacteriaceae	VRE	660
Pakistan	50% Gram-negative	Enterococcus	VRE	354
Romania	100% Gram-positive	Staphylococcus	MRSA	63
South Africa	50% Gram-positive and 50% gram-positive	Staphylococcus & Enterobacteriaceae	MRSA, CEREC	275
Thailand	81% Gram-negative	Enterobacteriaceae & Moraxellaceae	CRAB	750
Turkey	67% Gram-negative	Enterobacteriaceae & Staphylococcus, Moraxellaceae	MRSA, CREC	2190

MRSA= Methicilin-resistance *Staphylococcus aureus*, CREC= Carbapenem-resistant *Escherichia coli*, CRAB= Carbapenem-resistant *Acinetobacter baumanii*, VRE= Vancomycin-resistant *Enterocuccus spp*., CEREC= Cephalosporins-resistant Enterobactericieae, CRPA= Carbapenem-resistant Pseudomonas aureginosa, CRKP= Carbapenem-resistant *Klebsciella pneumoniae*, CREN= Carbpanem-resistant Enetrobactericiacea.

Table F. Descriptive statistics of the studies included in the meta-analysis (N=109 studies‡).

Characteristics/ARB sensitivity*	Patients with ASB BSIs				Patients with ARB BSIs					
	Mean	Median	IQR •	SD	Mean	Median	IQR •	SD	N[a]	χ^2 or T-tests[b]
Mortality rate (%)	22.37	19.15	19.20	14.24	44.03	42.11	29.76	20.41	100	9.42 (p<0.0001)
ICU admission (%)	25.24	23.50	10.70	10.61	36.37	32.30	16.70	17.22	42	3.57 (p<0.0001)
Length of hospital stay (LOS) ❋	27.93	25.00	23.33	10.61	54.03	56.52	28.84	17.22	52	7.55 (p<0.0001)
Demographic and background characteristics										
Age in years	53.45	56.95	12.30	11.28	53.94	56.10	12.16	10.79	66	0.26 (p=0.796)
Sex (Female) (%)	40.93	40.55	16.88	12.11	37.58	37.08	12.84	9.94	73	1.86 (p=0.064)
Community-acquired infection (%)	31.72	23.48	29.21	27.01	19.82	9.68	27.08	24.57	35	1.98 (p=0.051)
Prior surgery (%)	30.32	29.44	21.85	15.38	38.26	38.90	26.25	17.52	44	2.26 (p=0.027)
Previous hospitalisation (%)	44.87	46.92	20.22	16.76	56.05	58.89	20.93	18.19	29	2.64 (p=0.011)
Comorbidities and underlying conditions										
Diabetes (%)	21.18	18.18	15.50	10.43	22.41	20.00	15.01	10.66	48	0.58 (p=0.564)
Hypertension (%)	30.57	27.75	10.03	13.45	33.84	29.59	22.03	17.62	18	0.63 (p=0.536)
Cardiovascular diseases (%)	18.72	16.00	19.91	15.85	21.91	18.75	16.73	18.14	34	0.78 (p=0.436)
Solid tumour or malignancy (%)	29.97	25.81	24.28	21.81	23.97	16.02	15.10	21.78	39	1.26 (p=0.211)
Liver disease (%)	10.50	8.30	10.58	5.95	12.74	9.09	12.37	11.41	19	0.79 (p=0.432)
Kidney or renal disease (%)	15.20	12.20	20.57	10.43	21.16	19.35	18.13	13.22	36	2.15 (p=0.035)
Pulmonary disease (%)	12.15	9.09	15.13	9.09	20.96	14.29	18.74	18.63	31	2.37 (p=0.021)
Hematologic disease (%)	21.94	14.69	18.82	20.62	25.41	16.67	24.51	24.13	25	0.59 (p=0.559)
Any comorbidity (%)	56.40	59.38	34.06	26.49	82.82	67.82	42.97	85.87	10	0.93 (p=0.365)
Source of reported bacteraemia [c]										
Urinary tract infection (%)	16.16	10.54	12.72	16.15	17.58	9.52	18.19	20.04	29	0.31 (p=0.758)
Pneumonia or respiratory (%)	27.91	23.57	23.82	18.68	34.93	31.39	30.97	22.44	37	1.45 (p=0.152)
Intravenous catheter or vascular (%)	30.55	25.94	25.94	21.93	30.24	30.30	32.95	19.11	34	0.07 (p=0.949)
Intrabdominal (gastrointestinal) or skin/soft tissues (%)	26.46	24.80	30.42	18.02	25.65	24.11	23.33	18.12	33	0.19 (p=0.853)
Scores										
Pitt bacteraemia at BSI onset	1.41	1.00	1.00	0.73	2.58	2.50	1.00	1.47	11	2.35 (p=0.029)
CHARLSON score at BSI onset	2.50	2.00	1.50	1.24	2.73	2.02	1.95	1.16	15	0.55 (p=0.588)

[a]N for the number of studies included.. [b] χ^2 or T-test were employed according to each variable's distribution (α=0.05). [c] Other and undetermined sources are not shown. SD stands for standard deviation. ARB: Antibiotic-resistant bacteria, ASB: Antibiotic-susceptible bacteria. ICU: Intensive care unit, APACHE II: Acute Physiology and Chronic Health Evaluation score II. SD: Standard deviation. [a]N considers the number of studies included (see Supplementary material 2); there is only one missing study that only reported ARB BSIs excess deaths. * We reported weighted means of proportions extracted from the original papers. •IQR= Interquartile range (75th percentile – 25th percentile). ‡ One study was excluded from the N=109 initial sample because it only reported excess mortality. ❈ 42 studies reported the average LOS, but only 18 reported the mean and SD, which were consecutively used for meta-analyses. χ2 tests were applied to binary variables to test difference in proportions, by group.

Fig B. Violin and kernel density estimates plots for the primary outcomes and by ARB susceptibility. R stands for antibiotic-resistant strains whereas S for antibiotic susceptible. Data were weighted. AMR= antimicrobial-resistant or (ARB).

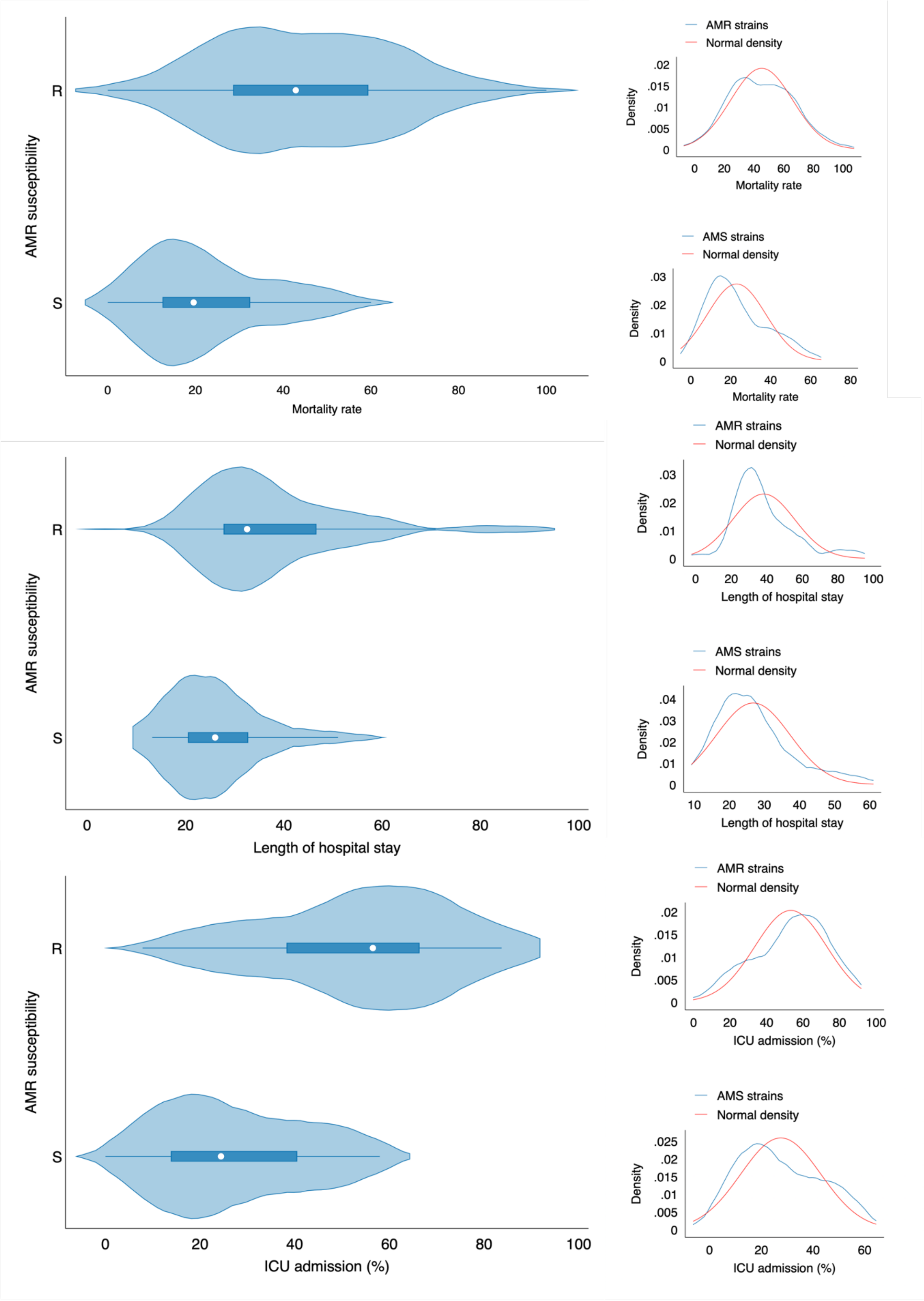

Fig C. Relationship between the primary outcomes: mortality rate, ICU admission, length of hospital stay. Weighted linear regressions were computed between the variables.

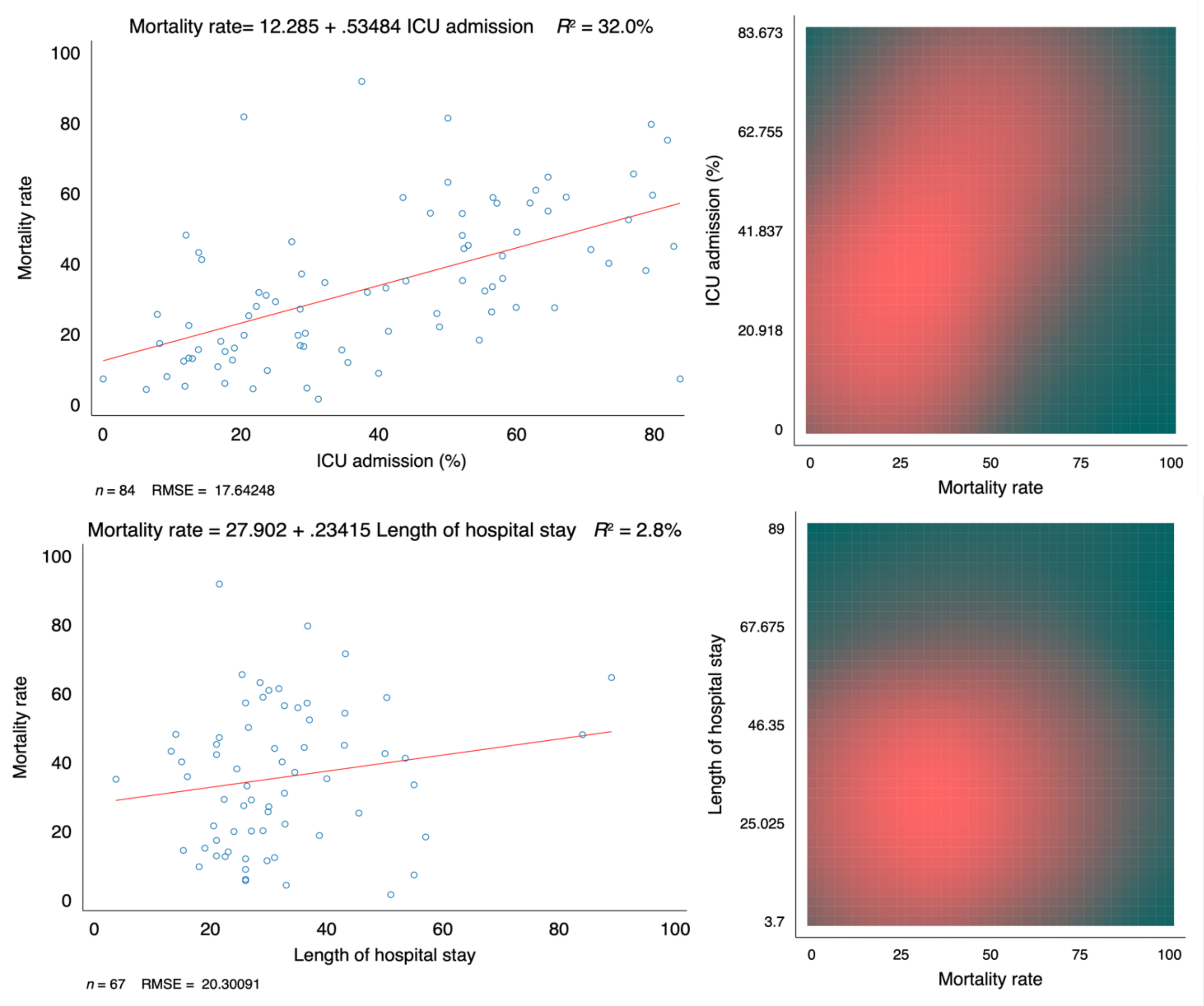

(3) Meta-analysis of full results

This section contains the results of the following analyses

a) Mortality results: general and subgroup meta-analyses

b) Length of hospital stay (LOS) results: general and subgroup meta-analyses

c) ICU admission results: general and subgroup meta-analyses

d) Mortality ORs unadjusted and adjusted results: general and subgroup meta-analyses

3.a) Mortality results: general and subgroup meta-analyses

Fig D. Meta-analysis using all the studies reporting mortality rates (N=93). Weights are from Doi's IVhet model. Dashed red line stands for the overall IVhet, whereas the black vertical line for OR=1. Hollow circles present the average within each study. Articles with duplicated samples were removed from the main analysis. IVhet estimate's p-value was p<0.001.

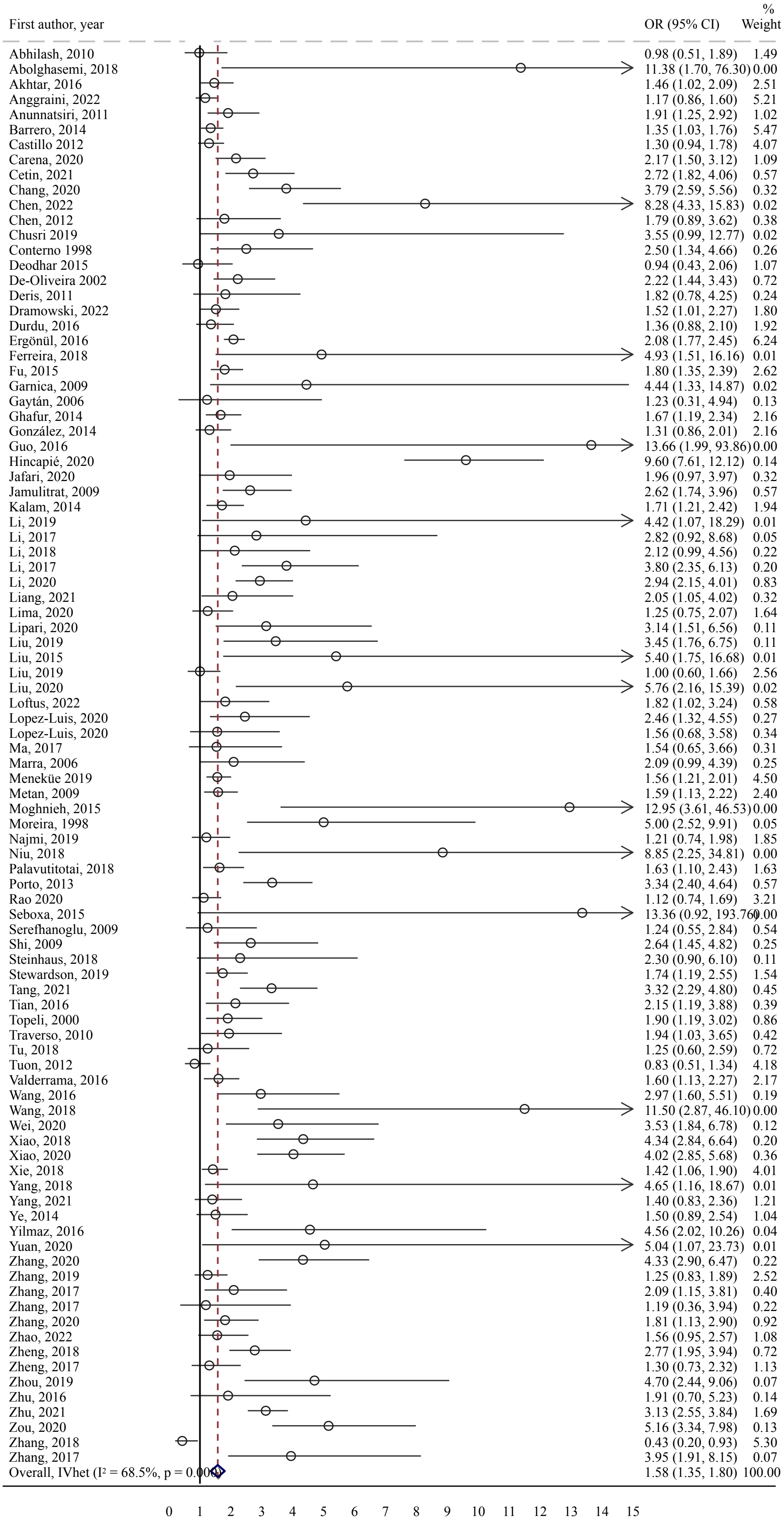

Fig E. Subgroup meta-analysis using all the studies reporting mortality rates/odds for critical (N=72) and high-priority (N=22) pathogens according to the WHO criteria: N total =94 studies. Weights are from Doi's IVhet model. Dashed red line stands for the overall IVhet, whereas the black vertical line for OR=1. Hollow circles present the average within each study. From the total studies (N=95), articles with duplicated samples were either removed from the analysis or considered only for gram-positives if only gram-positives were analysed at once and gram-negatives more than once for different bacteria within the articles' sample. Model for critical-priority pathogens p-value<0.001. Subgroup estimate's p-values were <0.001, and 0.045 for critical- and high-priority pathogens analyses, respectively. WHO= World Health Organization.

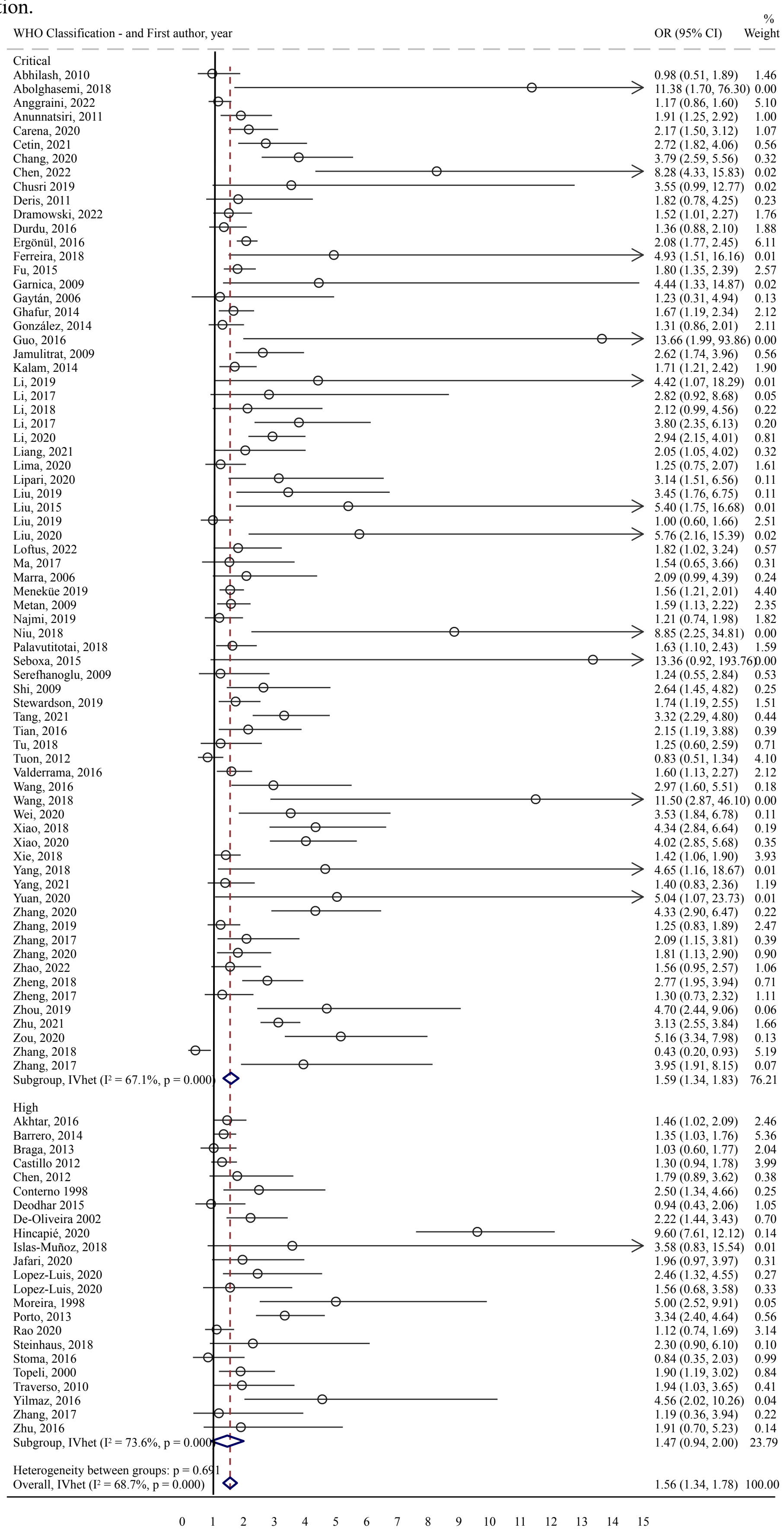

Fig F. Subgroup meta-analysis using all the studies reporting mortality rates by bacterium's family name (N= 97). Weights are from Doi's IVhet model. Dashed red line stands for the overall IVhet, whereas the black vertical line for OR=1. Hollow circles present the average within each study. *Enterococcus and Staphylococcaceae*: N=6, and N=17, respectively. Enterobacteriaceae, Pseudomonadaceae, Moraxellaceae: N=40, N=10, and N=16, respectively. Subgroup estimate's p-values were 0.005, 0.017, <0.001, 0.011 and 0.135 for Enterobactericieae, Enterococcus spp., Moraxellaceae, Pseudomonadaceae, and Staphylococcaceae models, respectively.

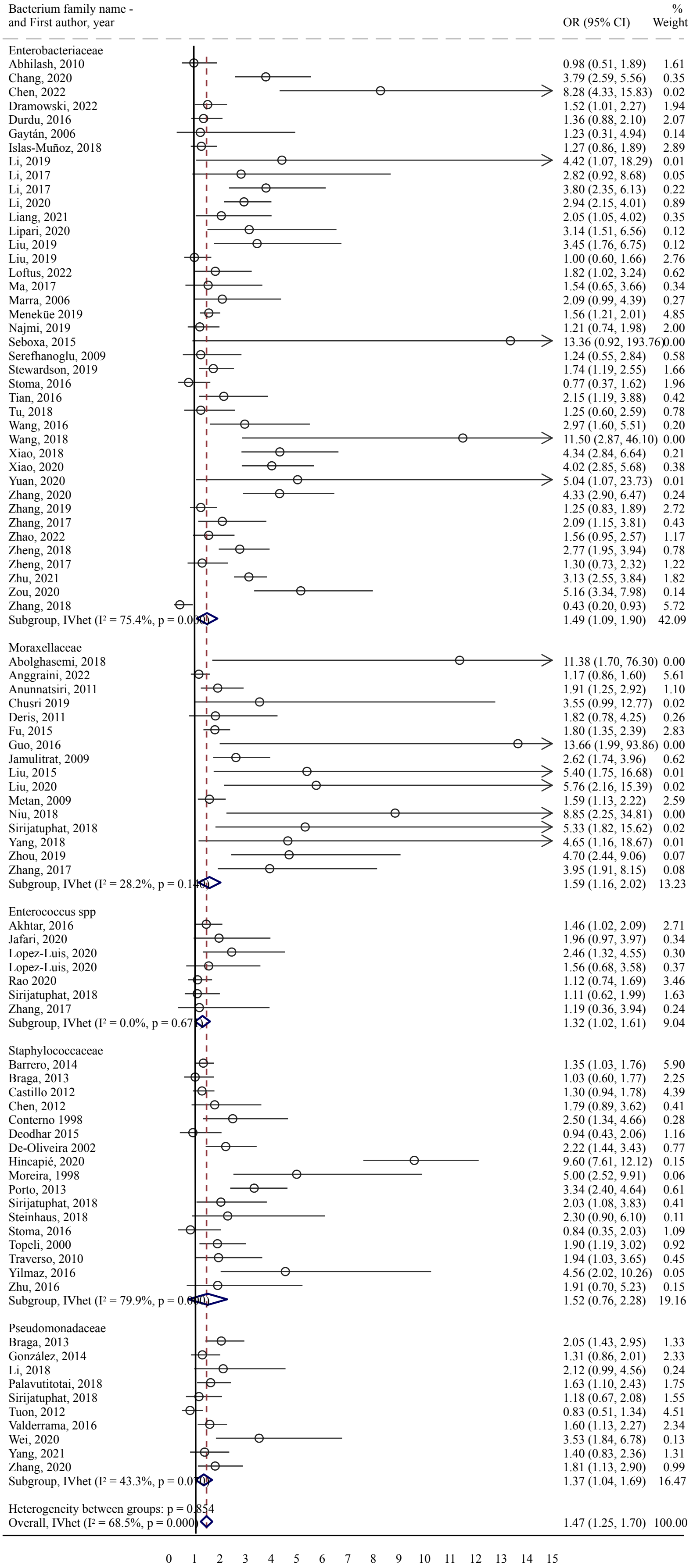

Fig G. Subgroup meta-analysis using all the studies reporting mortality rates by WHO Region (N=93). Weights are from Doi's IVhet model. Dashed red line stands for the overall IVhet, whereas the black vertical line for OR=1. Hollow circles present the average within each study. Studies with duplicated samples were excluded. Subgroup estimate's p-value were p=0.006, <0.001, 0.65, <0.001, 0.001, and 0.035 for Sout-East Asian Region, Eastern Mediterranean Region, the Americas, European Region, Western Pacific Region and the African Region models, respectively.

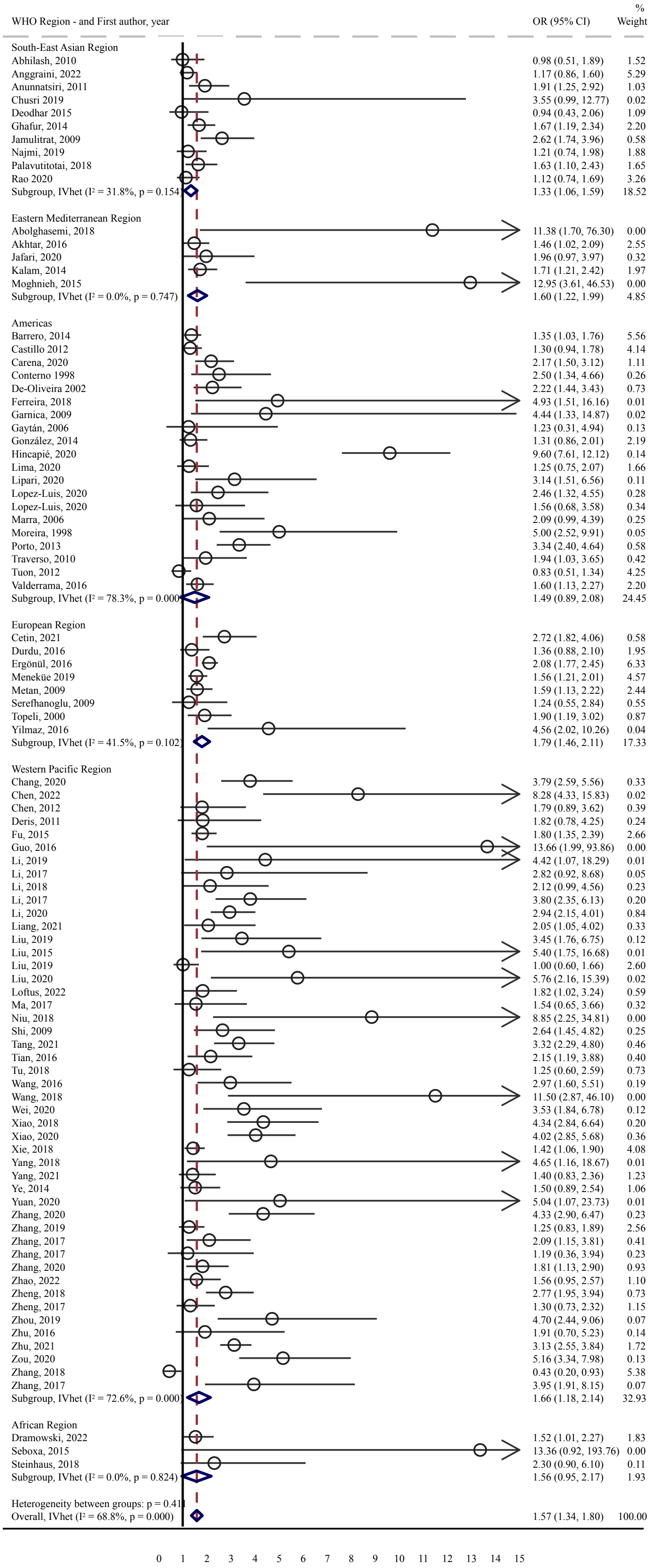

Fig H. Subgroup meta-analysis using all the studies reporting mortality rates by income level (N=92). Weights are from Doi's IVhet model. Dashed red line stands for the overall IVhet, whereas the black vertical line for OR=1. Hollow circles present the average within each study. Low income was compacted together with Lower-middle income countries. Low-income and Upper-Middle Income model subgroups presented an estimate's p-value<0.001.

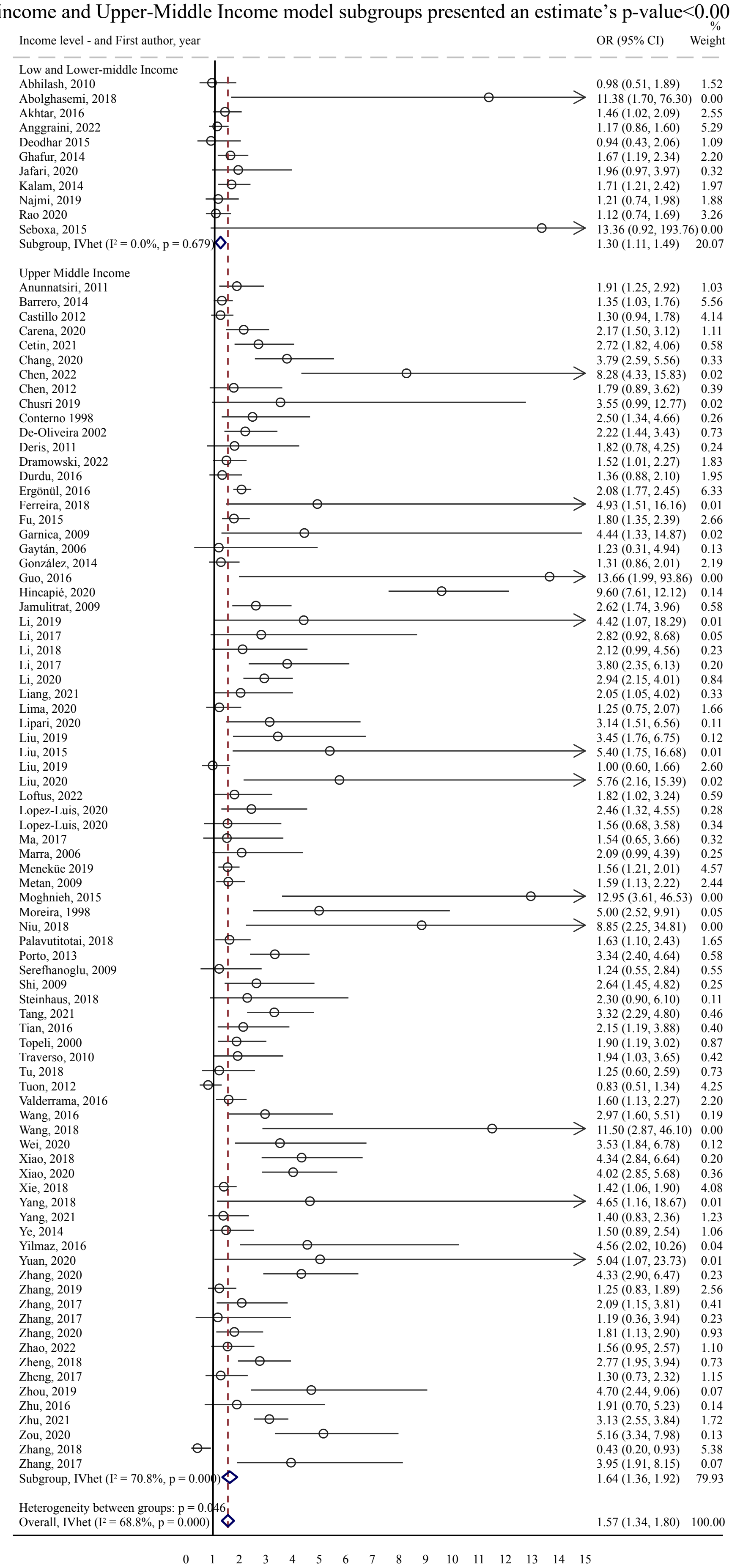

3.b) Length of hospital stay (LOS) results: general and subgroup meta-analyses

Fig I. Meta-analysis results using all the studies reporting the mean and standard deviation (SD) for the length of stay at the hospital (N=18). Weights are from Doi's IVhet model. SMD denotes standardised mean difference. Dashed red line stands for the overall IVhet, whereas the black vertical line for SMD=0. Overall estimate's p-value was p=0.001. SD= Standard deviation.

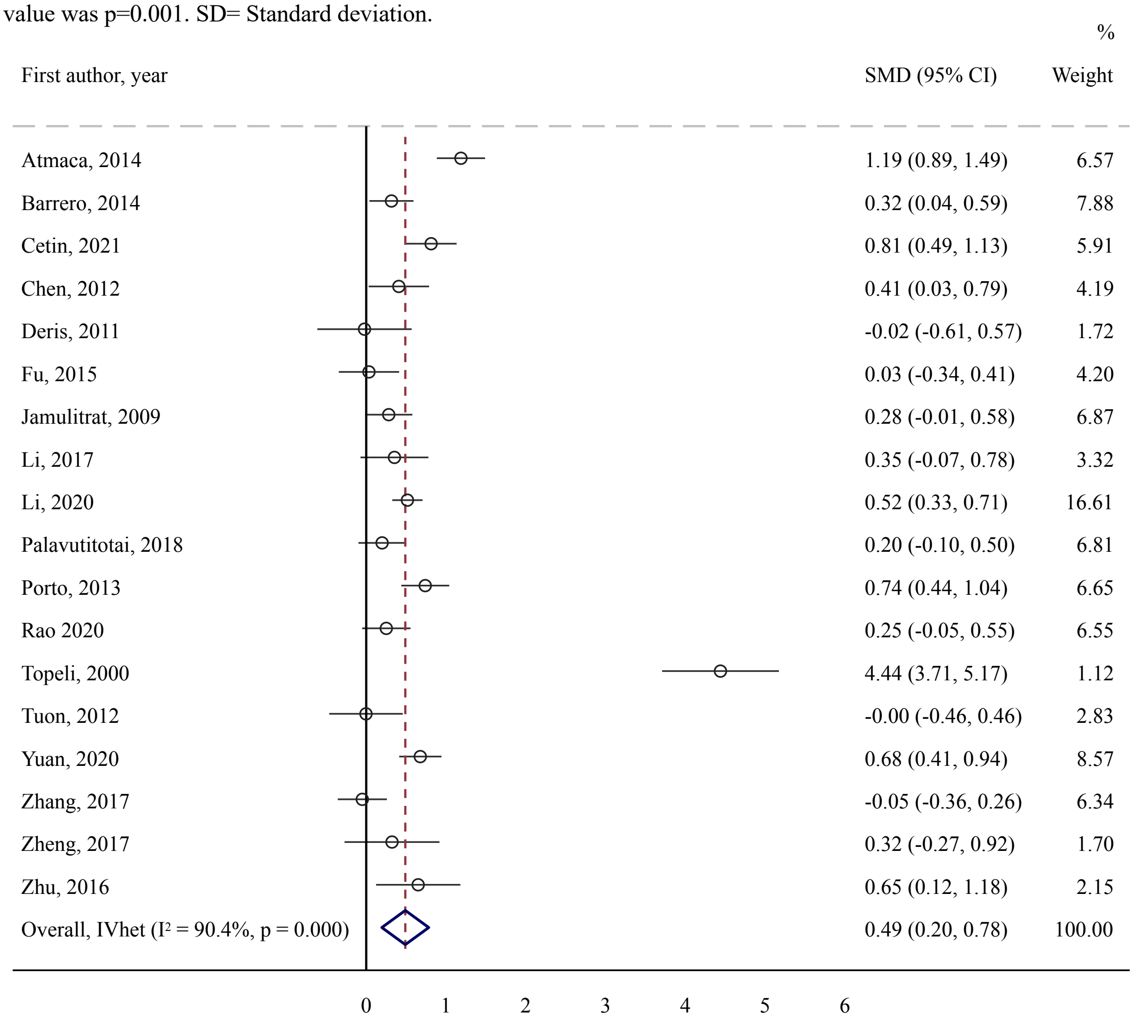

Fig J. Subgroup meta-analysis using all the studies reporting the mean and standard deviation (SD) for the length of stay at the hospital for critical and high-priority pathogens according to the WHO (N=11 and 7, respectively). Weights are from Doi's IVhet model. SMD denotes standardised mean difference. Dashed red line stands for the overall IVhet, whereas the black vertical line for SMD=0. Subgroup estimate's p-value were p<0.001 and p=0.040 for critical- and high-priority pathogen models, respectively. WHO= World Health Organization. SD= Standard deviation.

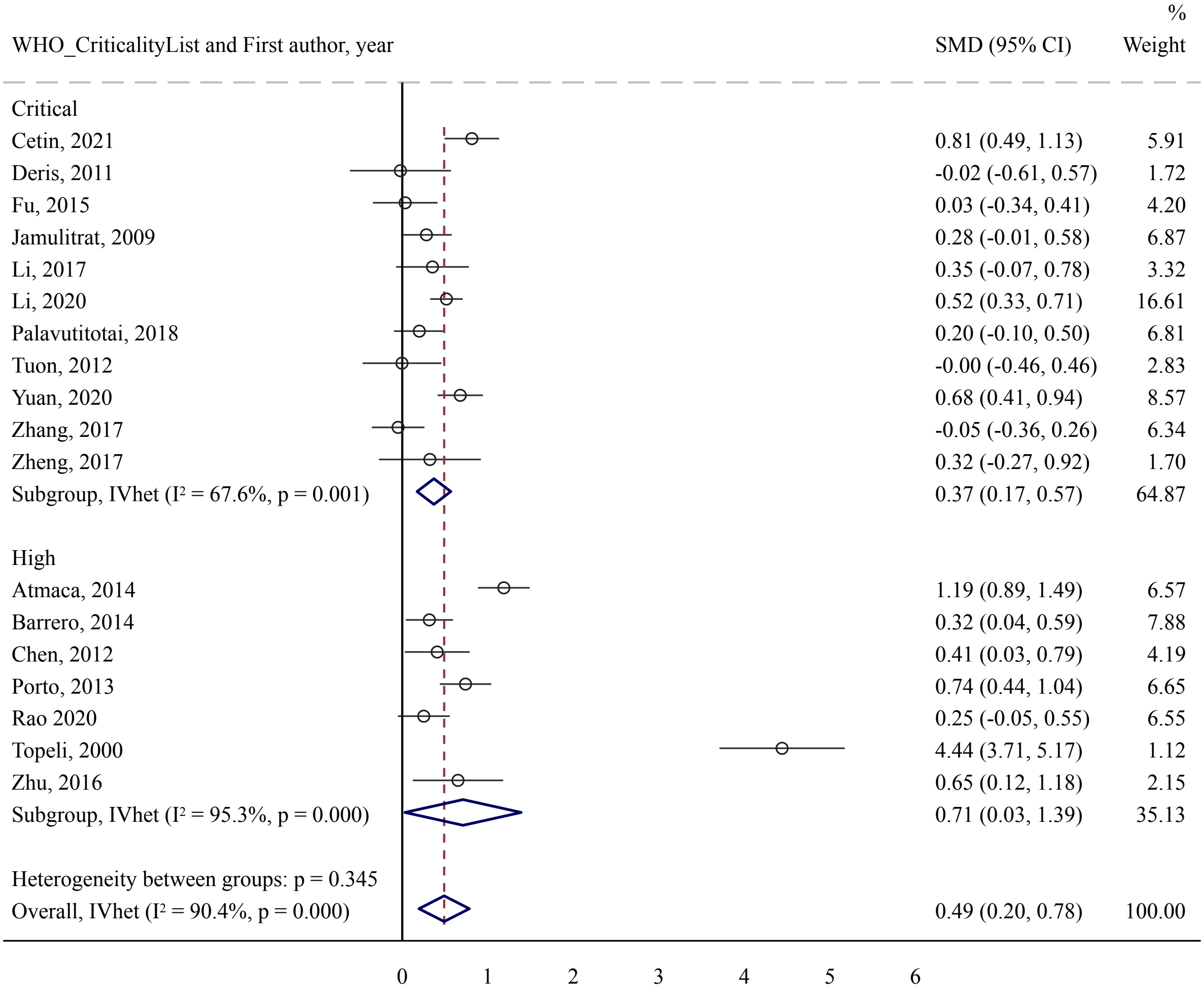

Fig K. Subgroup meta-analysis using all the studies reporting the mean and standard deviation (SD) for the length of stay at the hospital for Enterococcus spp. (N=1), Enterobacteriaceae (N=5), Moraxellaceae (N=3), Pseudomonadaceae (N=2), and Staphyloccocaceae (N=6). Weights are from Doi's IVhet model. SMD denotes standardised mean difference. Dashed red line stands for the overall IVhet, whereas the dotted black vertical line for SMD=0. Subgroup estimate's p-values were 0.004, 0.102, 0.155, 0.276, and 0.047 for Enterobactericieae, Enterococcus spp., Moraxellaceae, Pseudomonadaceae, and Staphylococcaceae models, respectively. SD= Standard deviation.

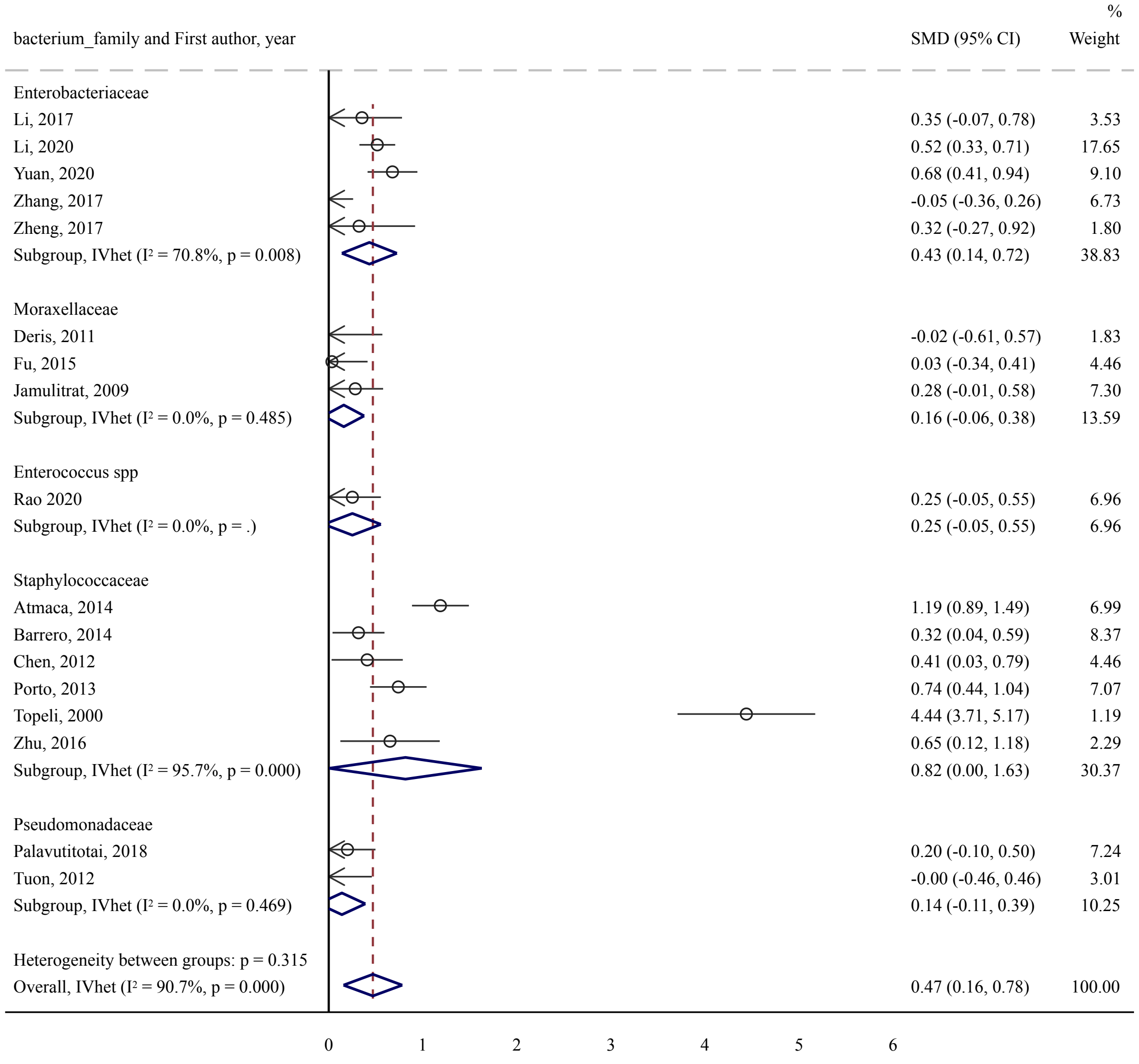

Fig L. Subgroup meta-analysis using all the studies reporting the mean and standard deviation (SD) for the length of stay at the hospital by income level (N=1 for Low and lower-middle income level, N=17 for Upper middle income). Weights are from Doi's IVhet model. SMD denotes standardised mean difference. Dashed red line stands for the overall IVhet, whereas the dotted black vertical line for SMD=0. Subgroup estimate's p-value were 0.102 and 0.002 for Lower Middle Income and Upper Middle Income models, respectively. SD= Standard deviation.

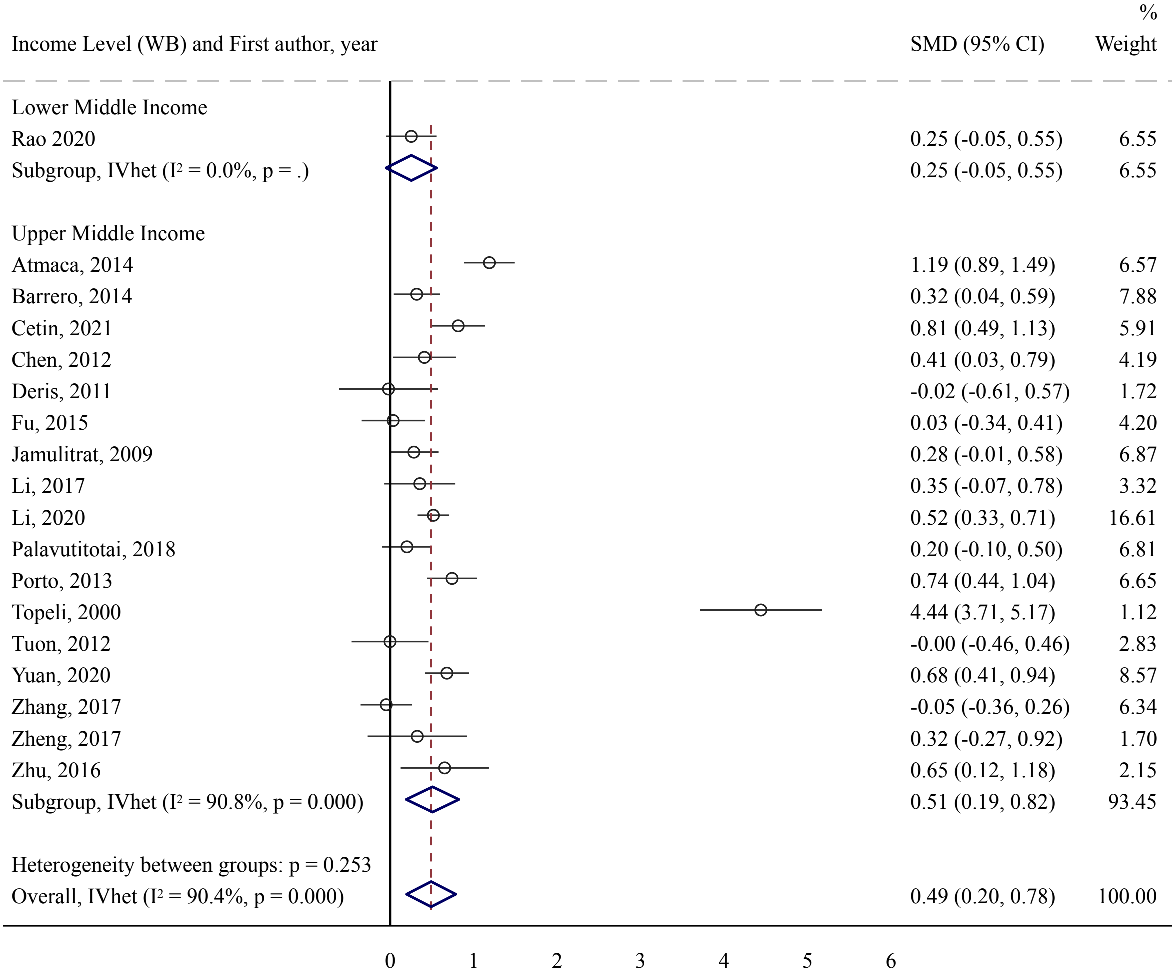

Fig M. Subgroup meta-analysis using all the studies reporting the mean and standard deviation (SD) for the length of stay at the hospital by WHO region (N=18). Weights are from Doi's IVhet model. SMD denotes standardised mean difference. Dashed red line stands for the overall IVhet, whereas the black vertical line for SMD=0. African and Eastern Mediterranean areas were omitted due to zero studies found providing LOS. Subgroup estimate's p-value were 0.007, 0.037, 0.120, and <0.001 for the South-East Asian Region, the Americas, European Region, and Western Pacific Region models, respectively. SD= Standard deviation. WHO= World Health Organization.

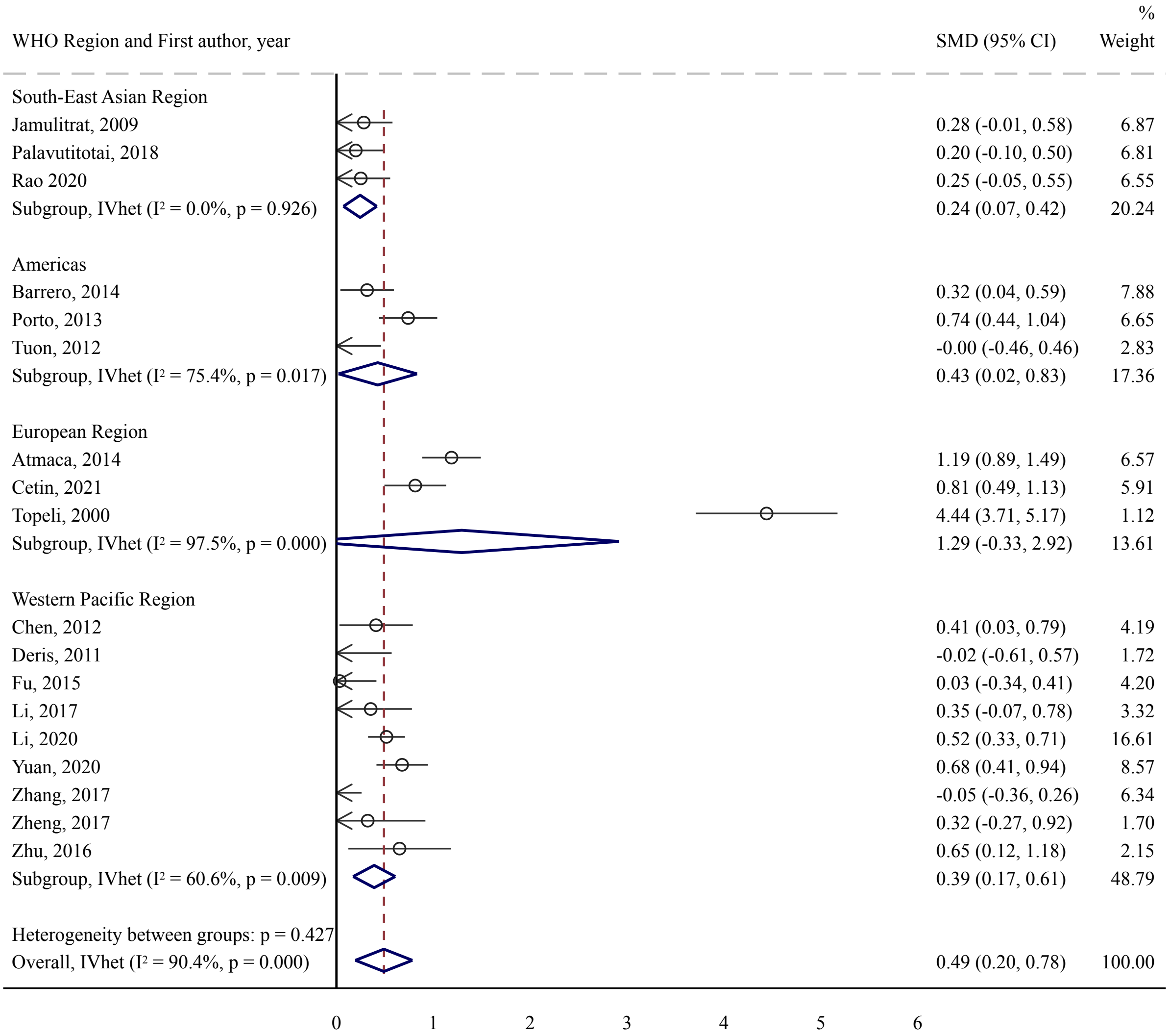

3.c) ICU admission results: general and subgroup meta-analyses

Fig N. Meta-analysis results using all the studies reporting ICU admission rates (N=52). Weights are from Doi's IVhet model. Dashed red line stands for the overall IVhet, whereas the dotted black vertical line for OR=1. Overall estimate's p-value was p<0.001.

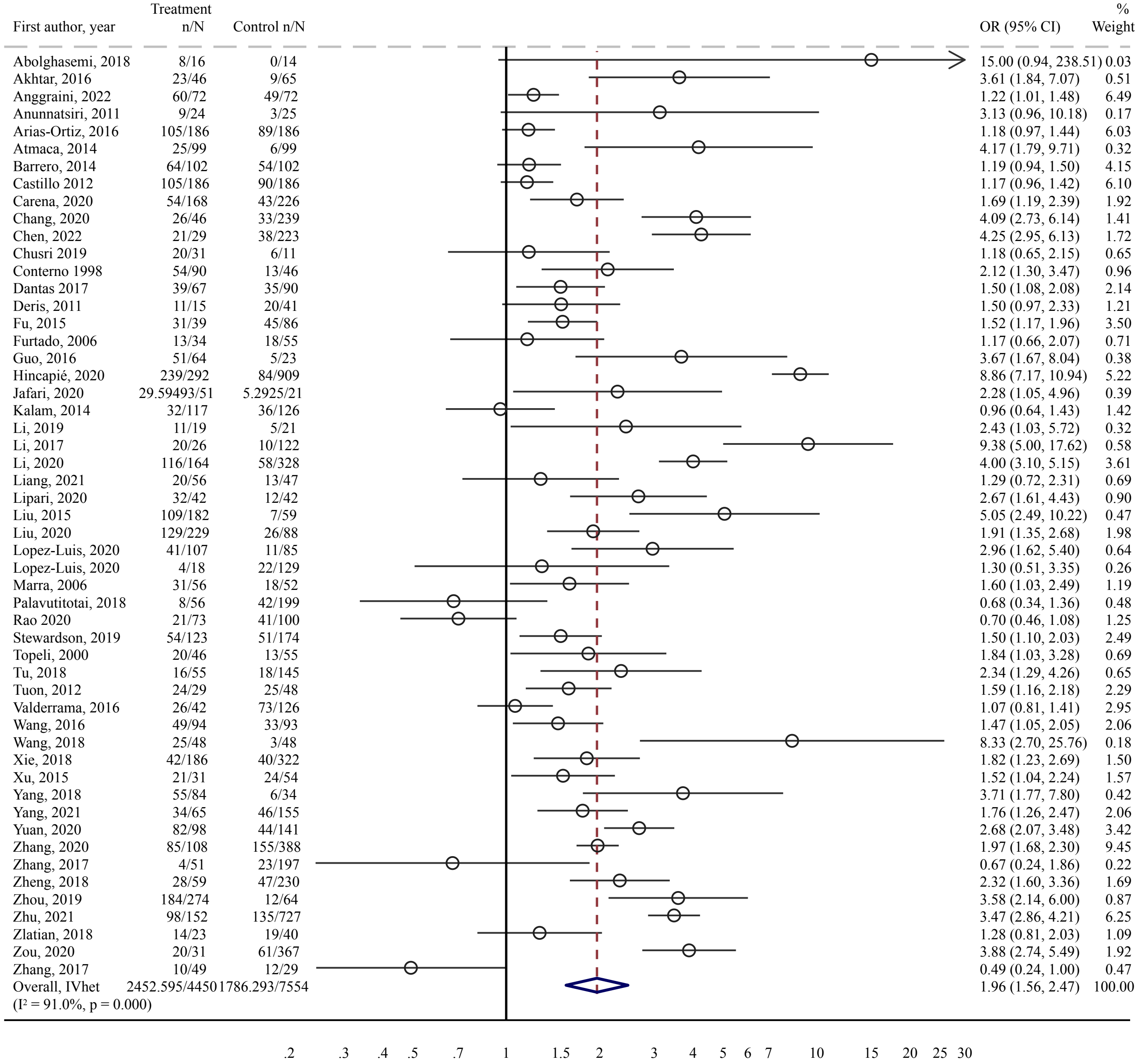

Fig O. Subgroup meta-analysis using all the studies reporting ICU admission rates for <u>critical</u> pathogens according to the WHO criteria (N=38). Weights are from Doi's IVhet model. Dashed red line stands for the overall IVhet, whereas the dotted black vertical line for OR=1.Estimate's p-value was <0.001. WHO= World Health Organization.

First author, year	Treatment n/N	Control n/N	OR (95% CI)	% Weight
Abolghasemi, 2018	8/16	0/14	15.00 (0.94, 238.51)	0.04
Anggraini, 2022	60/72	49/72	1.22 (1.01, 1.48)	9.25
Anunnatsiri, 2011	9/24	3/25	3.13 (0.96, 10.18)	0.24
Carena, 2020	54/168	43/226	1.69 (1.19, 2.39)	2.74
Chang, 2020	26/46	33/239	4.09 (2.73, 6.14)	2.01
Chen, 2022	21/29	38/223	4.25 (2.95, 6.13)	2.46
Chusri 2019	20/31	6/11	1.18 (0.65, 2.15)	0.92
Dantas 2017	39/67	35/90	1.50 (1.08, 2.08)	3.05
Deris, 2011	11/15	20/41	1.50 (0.97, 2.33)	1.73
Fu, 2015	31/39	45/86	1.52 (1.17, 1.96)	5.00
Guo, 2016	51/64	5/23	3.67 (1.67, 8.04)	0.54
Kalam, 2014	32/117	36/126	0.96 (0.64, 1.43)	2.02
Li, 2019	11/19	5/21	2.43 (1.03, 5.72)	0.45
Li, 2017	20/26	10/122	9.38 (5.00, 17.62)	0.83
Li, 2020	116/164	58/328	4.00 (3.10, 5.15)	5.15
Liang, 2021	20/56	13/47	1.29 (0.72, 2.31)	0.98
Lipari, 2020	32/42	12/42	2.67 (1.61, 4.43)	1.28
Liu, 2015	109/182	7/59	5.05 (2.49, 10.22)	0.66
Liu, 2020	129/229	26/88	1.91 (1.35, 2.68)	2.82
Marra, 2006	31/56	18/52	1.60 (1.03, 2.49)	1.70
Palavutitotai, 2018	8/56	42/199	0.68 (0.34, 1.36)	0.68
Stewardson, 2019	54/123	51/174	1.50 (1.10, 2.03)	3.55
Tu, 2018	16/55	18/145	2.34 (1.29, 4.26)	0.93
Tuon, 2012	24/29	25/48	1.59 (1.16, 2.18)	3.26
Valderrama, 2016	26/42	73/126	1.07 (0.81, 1.41)	4.21
Wang, 2016	49/94	33/93	1.47 (1.05, 2.05)	2.93
Wang, 2018	25/48	3/48	8.33 (2.70, 25.76)	0.26
Xie, 2018	42/186	40/322	1.82 (1.23, 2.69)	2.13
Yang, 2018	55/84	6/34	3.71 (1.77, 7.80)	0.60
Yang, 2021	34/65	46/155	1.76 (1.26, 2.47)	2.93
Yuan, 2020	82/98	44/141	2.68 (2.07, 3.48)	4.88
Zhang, 2020	85/108	155/388	1.97 (1.68, 2.30)	13.48
Zhang, 2017	4/51	23/197	0.67 (0.24, 1.86)	0.32
Zheng, 2018	28/59	47/230	2.32 (1.60, 3.36)	2.41
Zhou, 2019	184/274	12/64	3.58 (2.14, 6.00)	1.24
Zhu, 2021	98/152	135/727	3.47 (2.86, 4.21)	8.91
Zou, 2020	20/31	61/367	3.88 (2.74, 5.49)	2.74
Zhang, 2017	10/49	12/29	0.49 (0.24, 1.00)	0.67
Overall, IVhet (I^2 = 86.3%, p = 0.000)	1674/3066	1288/5422	2.02 (1.62, 2.52)	100.00

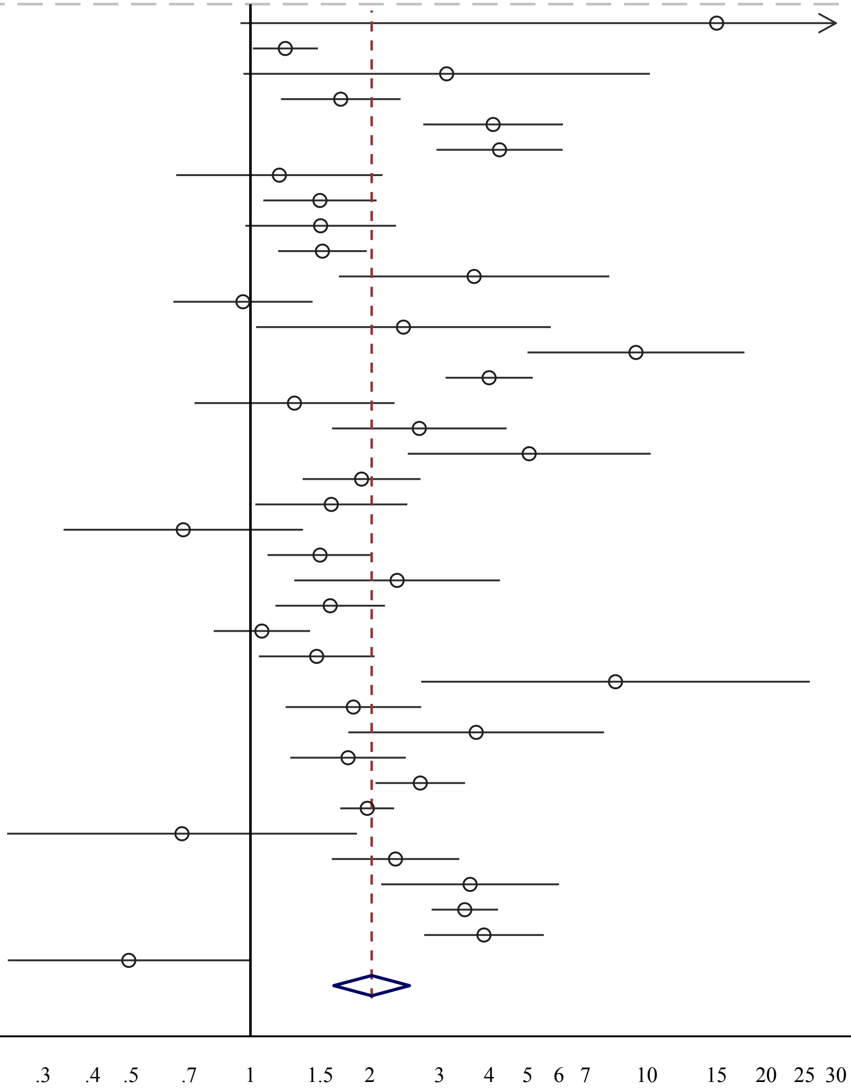

Fig P. Subgroup meta-analysis using all the studies reporting ICU admission rates for high-priority pathogens according to the WHO criteria (N=14). Weights are from Doi's IVhet model. Dashed red line stands for the overall IVhet, whereas the dotted black vertical line for OR=1. Estimate's p-value was 0.055. WHO= World Health Organization.

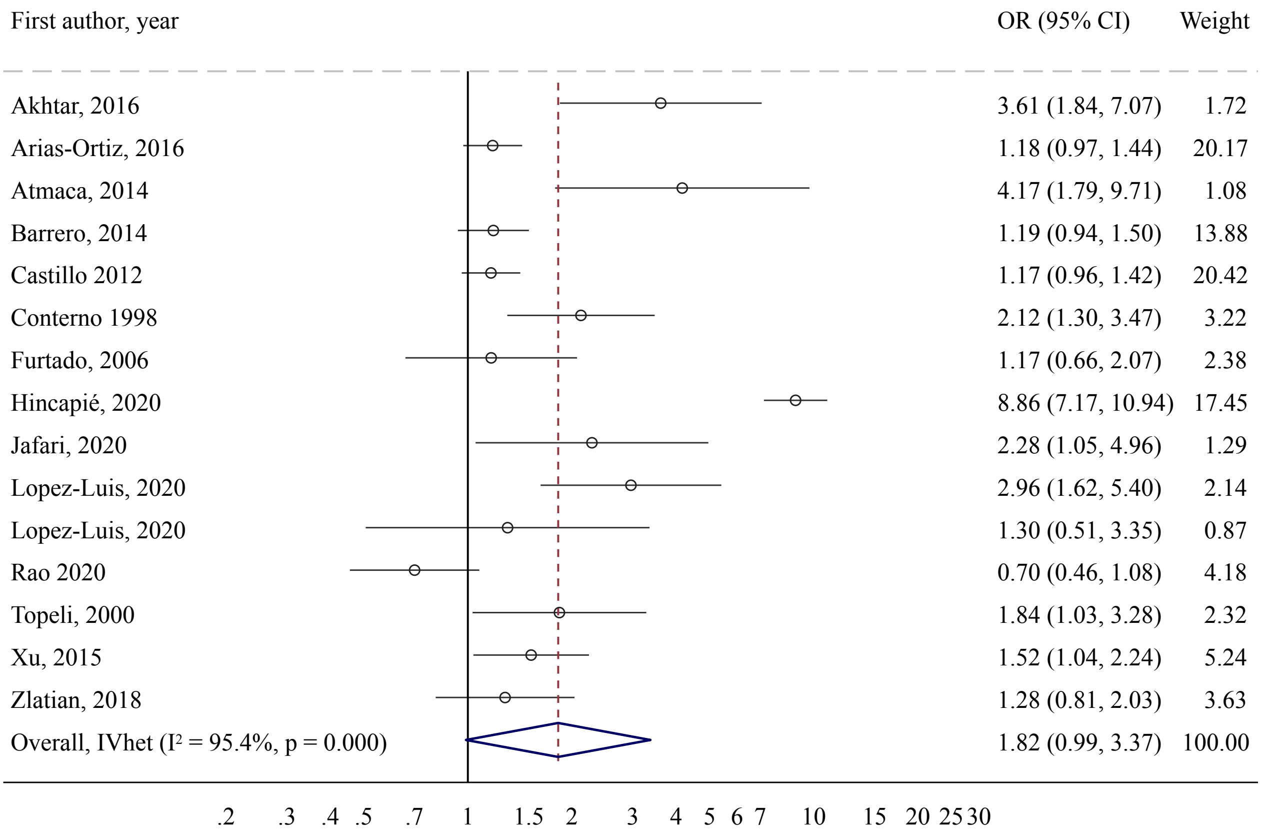

Fig Q. Subgroup meta-analysis using all the studies reporting ICU admission rates for Enterobacteriaceae (N=18). Weights are from Doi's IVhet model. Dashed red line stands for the overall IVhet, whereas the dotted black vertical line for OR=1. Estimate's p-value was <0.001.

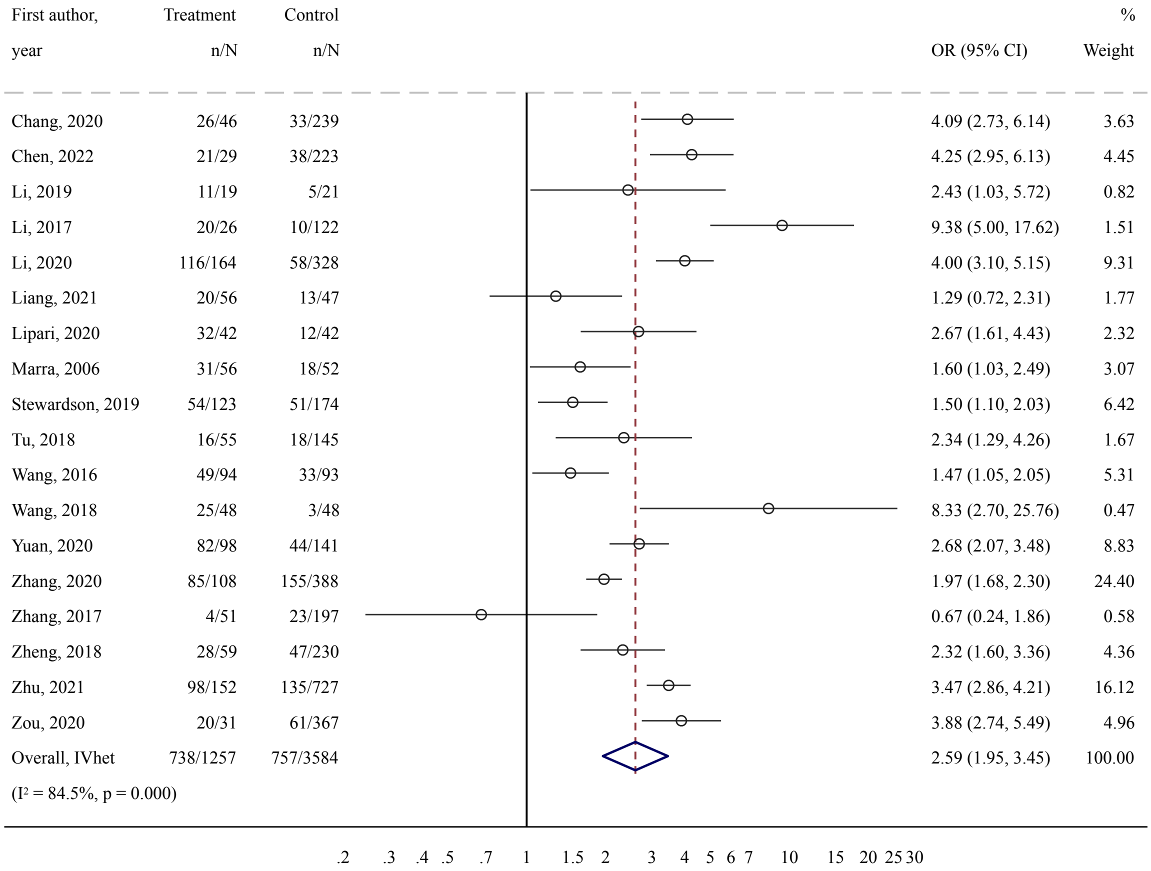

Fig R. Subgroup meta-analysis using all the studies reporting ICU admission rates for Enterococcus spp. (N=6). Weights are from Doi's IVhet model. Dashed red line stands for the overall IVhet, whereas the dotted black vertical line for OR=1. Estimate's p-value was 0.119.

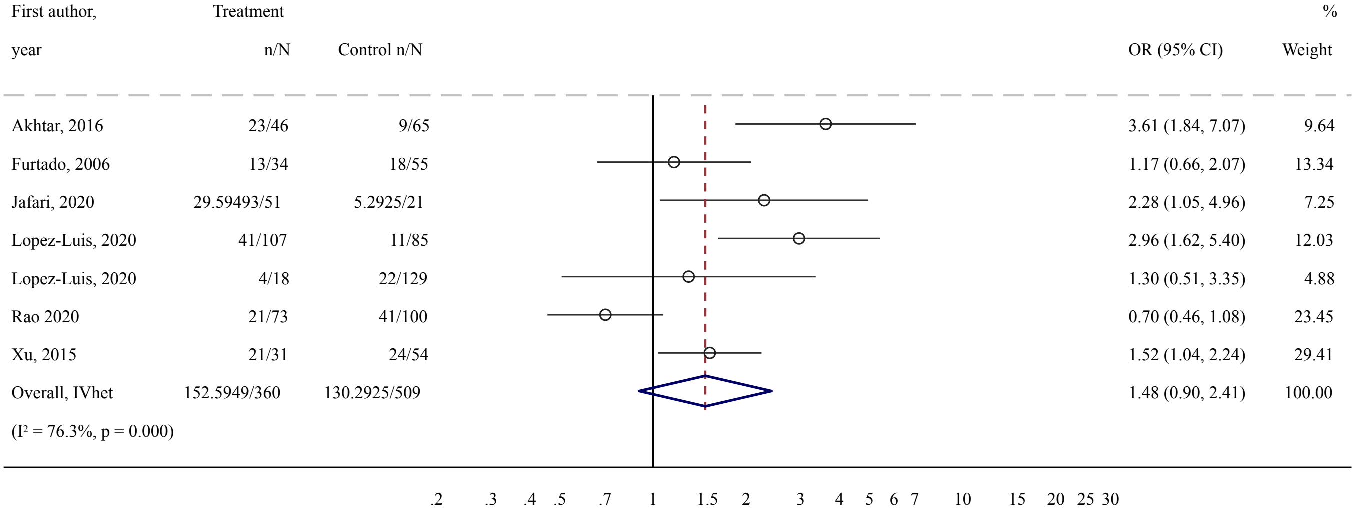

Fig S. Subgroup meta-analysis using all the studies reporting ICU admission rates for Moraxellaceae (N=12). Weights are from Doi's IVhet model. Dashed red line stands for the overall IVhet, whereas the dotted black vertical line for OR=1. Estimate's p-value was 0.039.

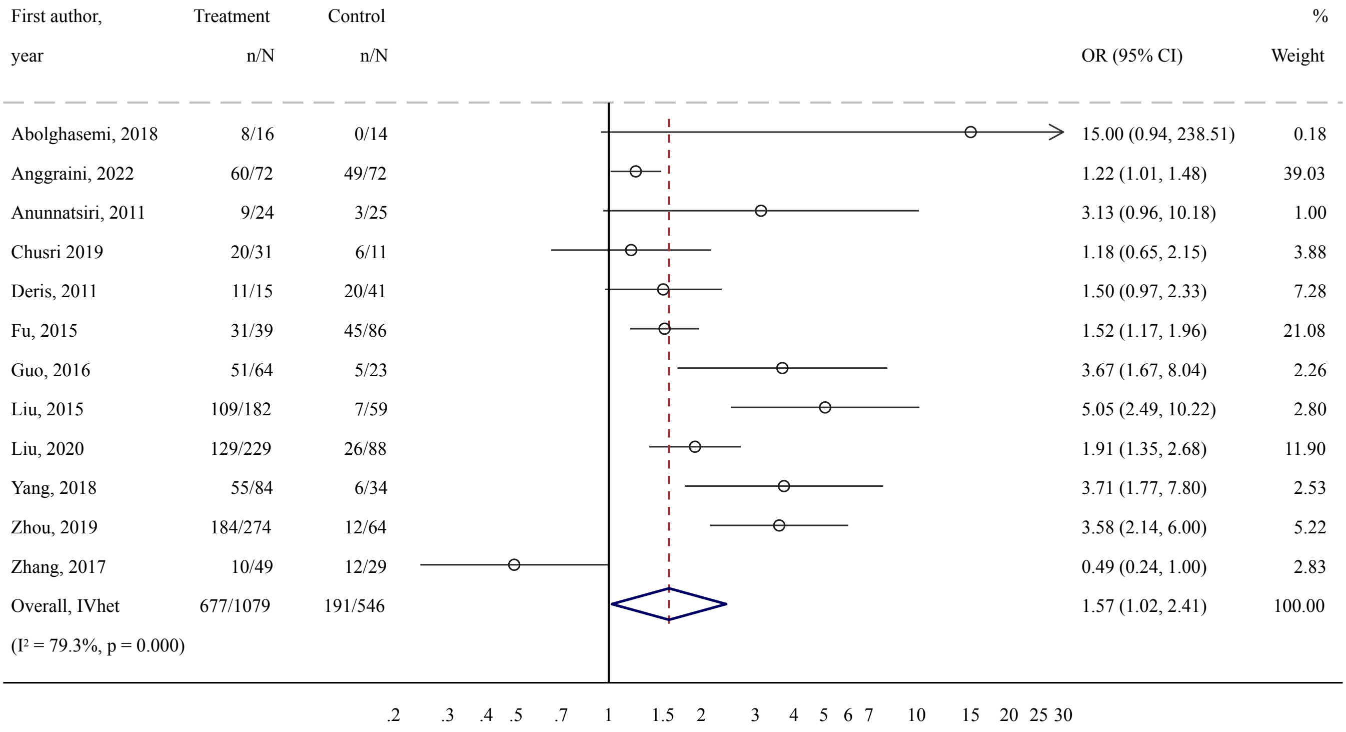

Fig T. Subgroup meta-analysis using all the studies reporting ICU admission rates for Pseudomonadaceae (N=5). Weights are from Doi's IVhet model. Dashed red line stands for the overall IVhet, whereas the dotted black vertical line for OR=1. Estimate's p-value was 0.018.

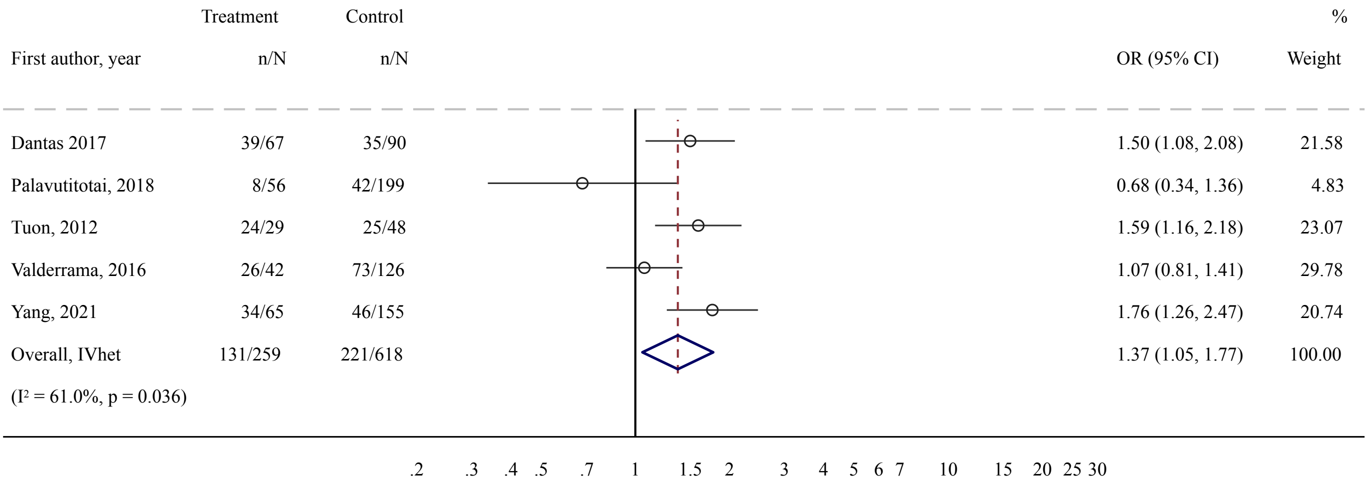

Fig U. Subgroup meta-analysis using all the studies reporting ICU admission rates for Staphylococcaceae (N=8). Weights are from Doi's IVhet model. Dashed red line stands for the overall IVhet, whereas the dotted black vertical line for OR=1. Estimate's p-value was 0.112.

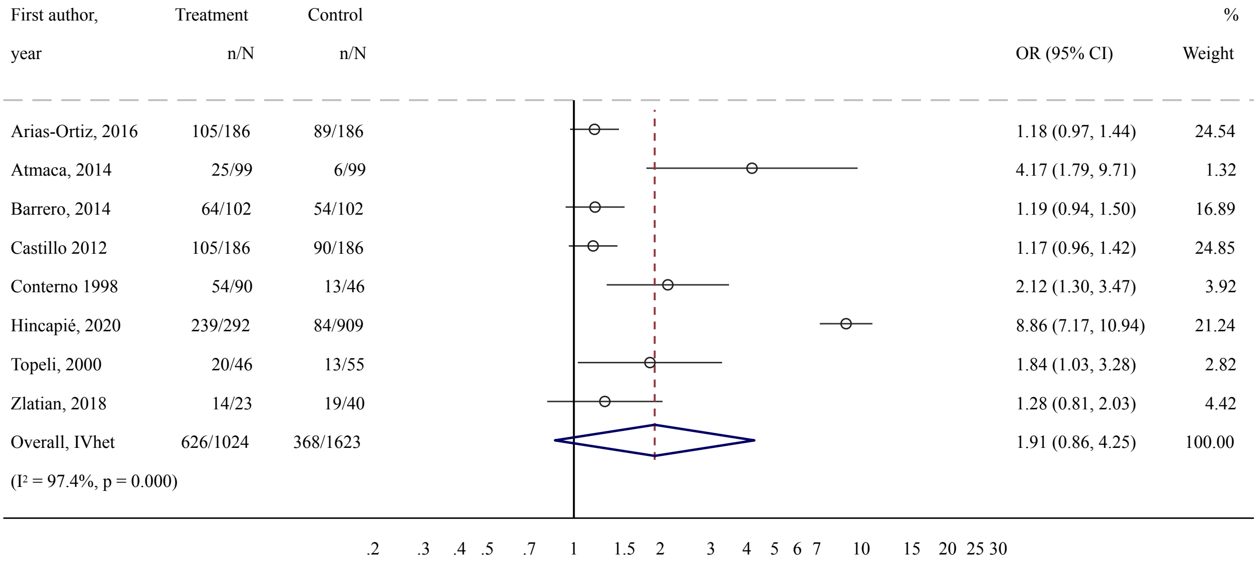

Fig V. Subgroup meta-analysis using all the studies reporting ICU admission rates (ESBL+) (N=2). Weights are from Doi's IVhet model. Dashed red line stands for the overall IVhet, whereas the dotted black vertical line for OR=1. Estimate's p-value was 0.472. ESBL= Extended Spectrum Beta-lactamase.

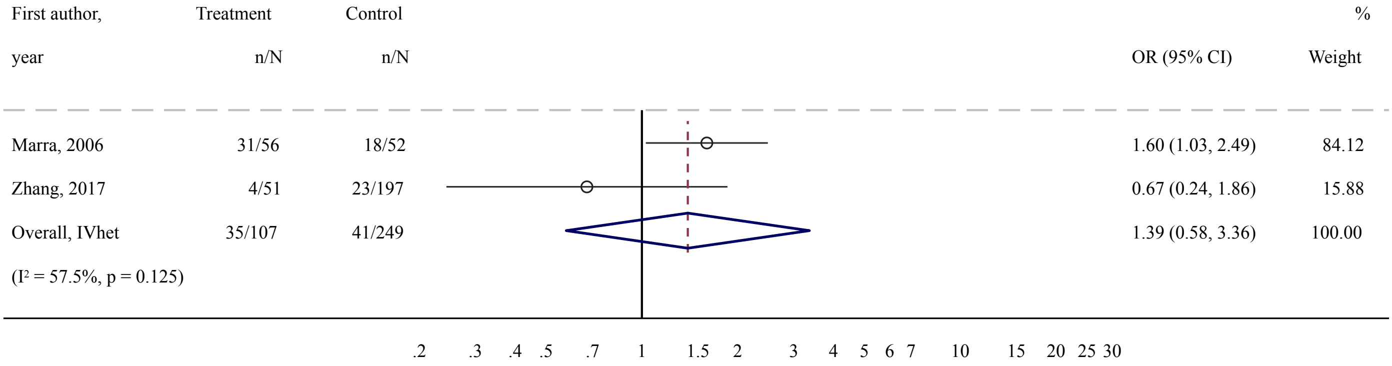

Fig W. Subgroup meta-analysis using all the studies reporting ICU admission rates by WHO region: Americas (N=13). Weights are from Doi's IVhet model. Dashed red line stands for the overall IVhet, whereas the dotted black vertical line for OR=1. Estimate's p-value was 0.023. WHO= World Health Organization.

First author, year	Treatment n/N	Control n/N	OR (95% CI)	% Weight
Arias-Ortiz, 2016	105/186	89/186	1.18 (0.97, 1.44)	16.99
Barrero, 2014	64/102	54/102	1.19 (0.94, 1.50)	11.70
Castillo 2012	105/186	90/186	1.17 (0.96, 1.42)	17.21
Carena, 2020	54/168	43/226	1.69 (1.19, 2.39)	5.42
Conterno 1998	54/90	13/46	2.12 (1.30, 3.47)	2.72
Dantas 2017	39/67	35/90	1.50 (1.08, 2.08)	6.04
Furtado, 2006	13/34	18/55	1.17 (0.66, 2.07)	2.00
Hincapié, 2020	239/292	84/909	8.86 (7.17, 10.94)	14.71
Lipari, 2020	32/42	12/42	2.67 (1.61, 4.43)	2.54
Lopez-Luis, 2020	41/107	11/85	2.96 (1.62, 5.40)	1.81
Lopez-Luis, 2020	4/18	22/129	1.30 (0.51, 3.35)	0.73
Marra, 2006	31/56	18/52	1.60 (1.03, 2.49)	3.35
Tuon, 2012	24/29	25/48	1.59 (1.16, 2.18)	6.45
Valderrama, 2016	26/42	73/126	1.07 (0.81, 1.41)	8.33
Overall, IVhet	831/1419	587/2282	1.77 (1.08, 2.89)	100.00
(I^2 = 95.5%, p = 0.000)				

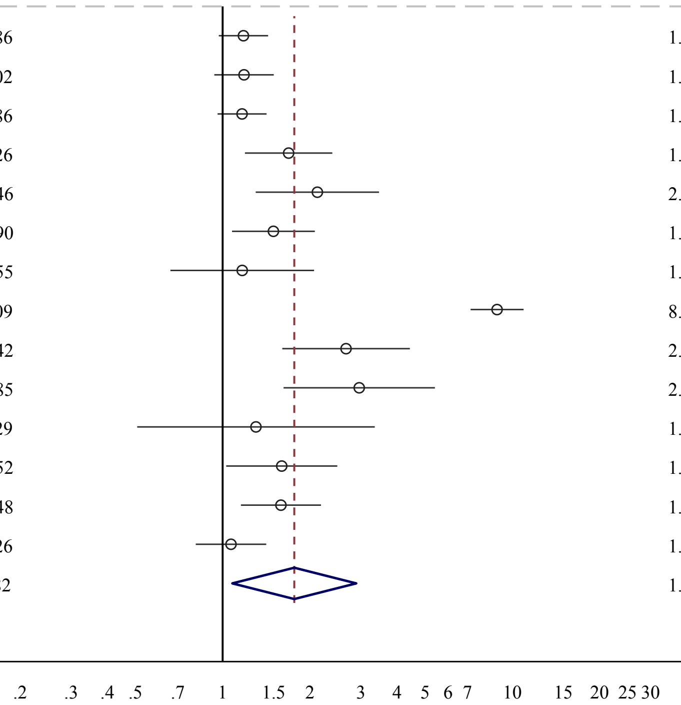

Fig X. Subgroup meta-analysis using all the studies reporting ICU admission rates by WHO region: Eastern Mediterranean (N=4). Weights are from Doi's IVhet model. Dashed red line stands for the overall IVhet, whereas the dotted black vertical line for OR=1. Estimate's p-value was 0.422. WHO= World Health Organization.

First author, year	Treatment n/N	Control n/N	OR (95% CI)	% Weight
Abolghasemi, 2018	8/16	0/14	15.00 (0.94, 238.51)	1.29
Akhtar, 2016	23/46	9/65	3.61 (1.84, 7.07)	21.87
Jafari, 2020	29.59493/51	5.2925/21	2.28 (1.05, 4.96)	16.45
Kalam, 2014	32/117	36/126	0.96 (0.64, 1.43)	60.39
Overall, IVhet	92.59493/230	50.2925/226	1.53 (0.55, 4.23)	100.00
(I^2 = 80.1%, p = 0.002)				

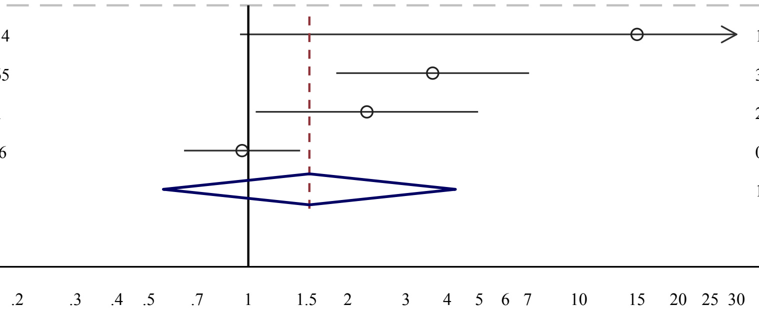

Fig Y. Subgroup meta-analysis using all the studies reporting ICU admission rates by WHO region: Europe (N=3). Weights are from Doi's IVhet model. Dashed red line stands for the overall IVhet, whereas the dotted black vertical line for OR=1. Estimate's p-value was 0.084. WHO= World Health Organization.

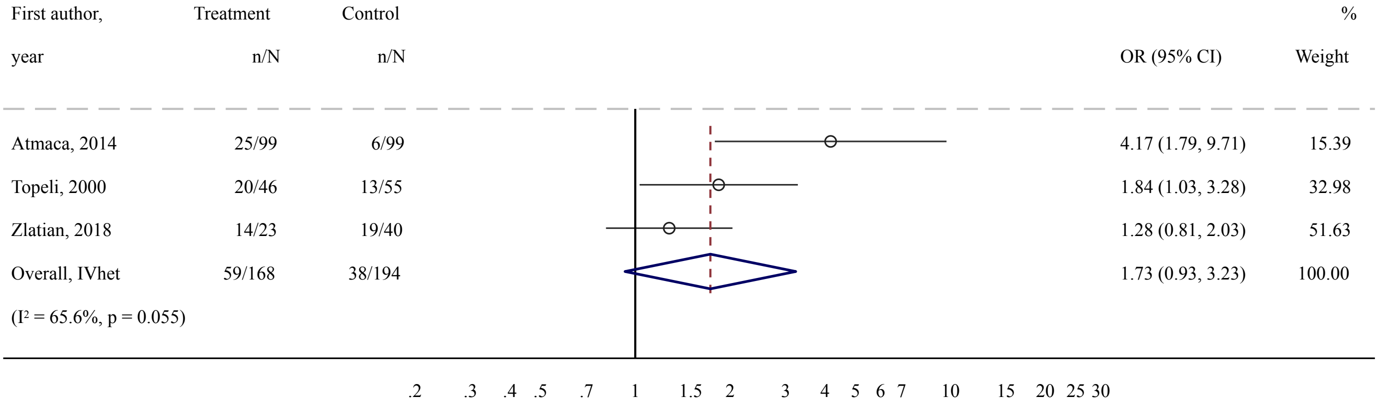

Fig Z. Subgroup meta-analysis using all the studies reporting ICU admission rates by WHO region: Southeast Asia (N=5). Weights are from Doi's IVhet model. Dashed red line stands for the overall IVhet, whereas the dotted black vertical line for OR=1. Estimate's p-value was 0.674. WHO= World Health Organization.

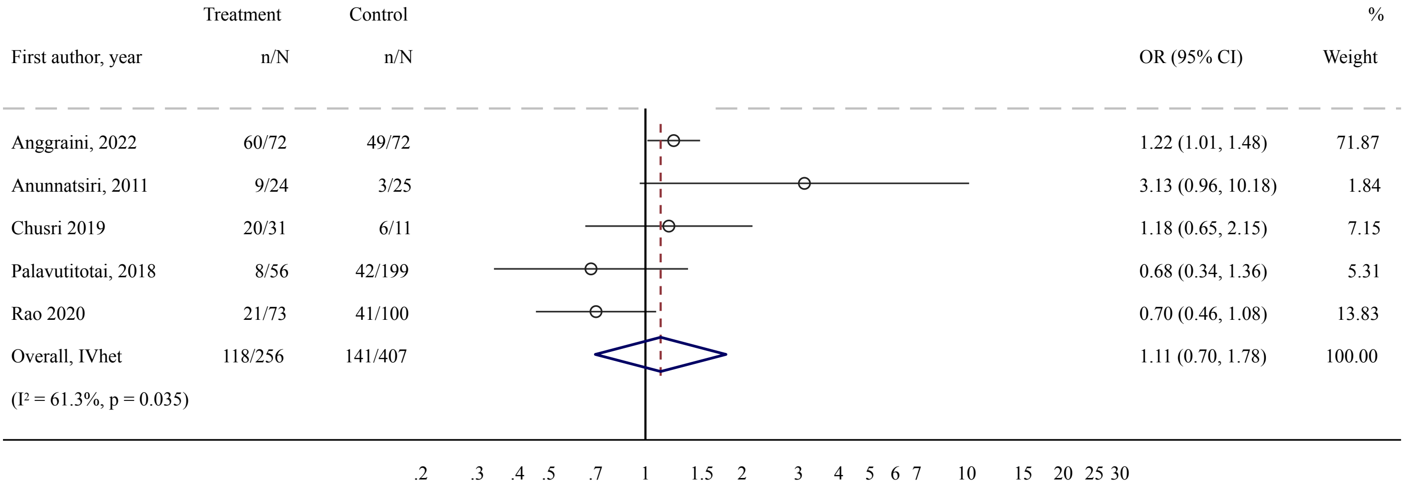

Fig AA. Subgroup meta-analysis using all the studies reporting ICU admission rates by WHO region: Western Pacific Region (N=26). Weights are from Doi's IVhet model. Dashed red line stands for the overall IVhet, whereas the dotted black vertical line for OR=1. The African region did not present studies enough to compute the analysis. Estimate's p-value was <0.001. WHO= World Health Organization.

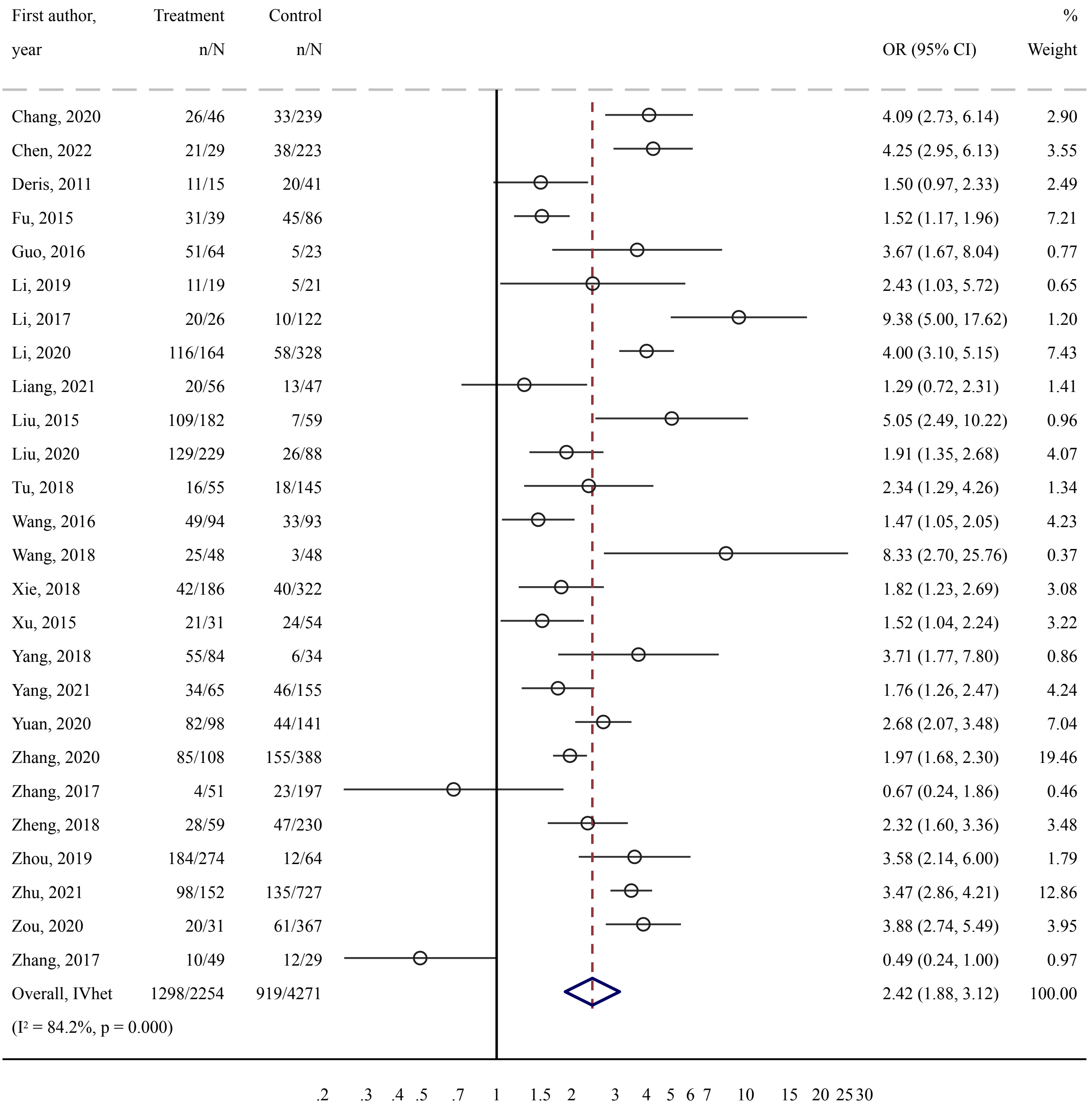

Fig AB. Subgroup meta-analysis using all the studies reporting ICU admission rates by income level: Low and Lower-Middle income countries (N=6). Weights are from Doi's IVhet model. Dashed red line stands for the overall IVhet, whereas the dotted black vertical line for OR=1. Estimate's p-value was 0.572.

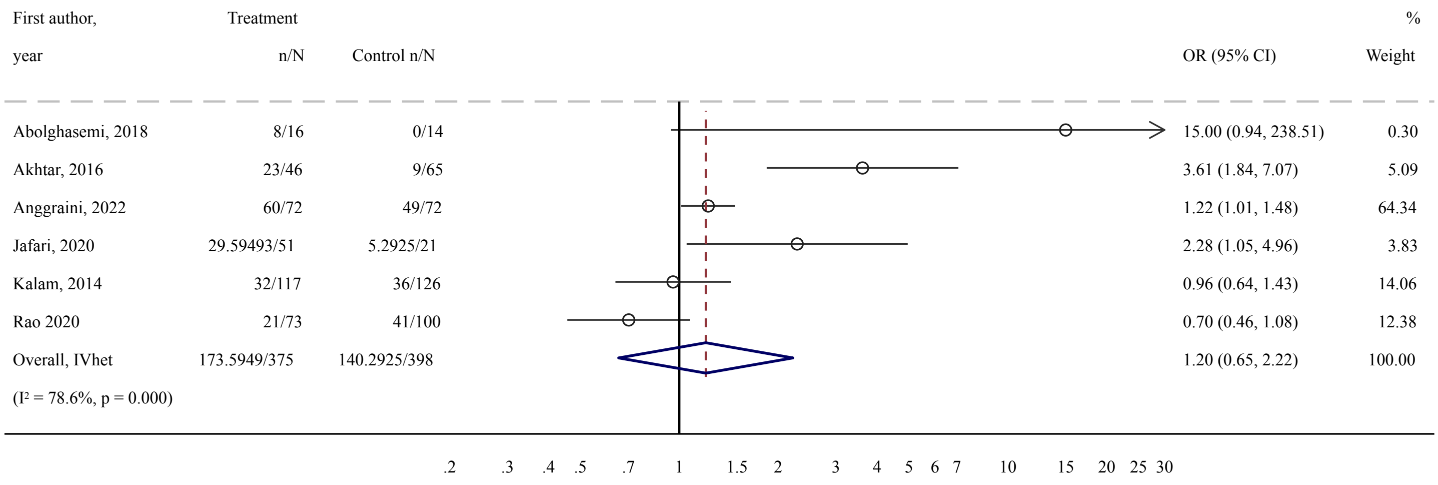

Fig AC. Subgroup meta-analysis using all the studies reporting ICU admission rates by income level: Upper-Middle income countries (N=45). Weights are from Doi's IVhet model. Dashed red line stands for the overall IVhet, whereas the dotted black vertical line for OR=1. Estimate's p-value was <0.001.

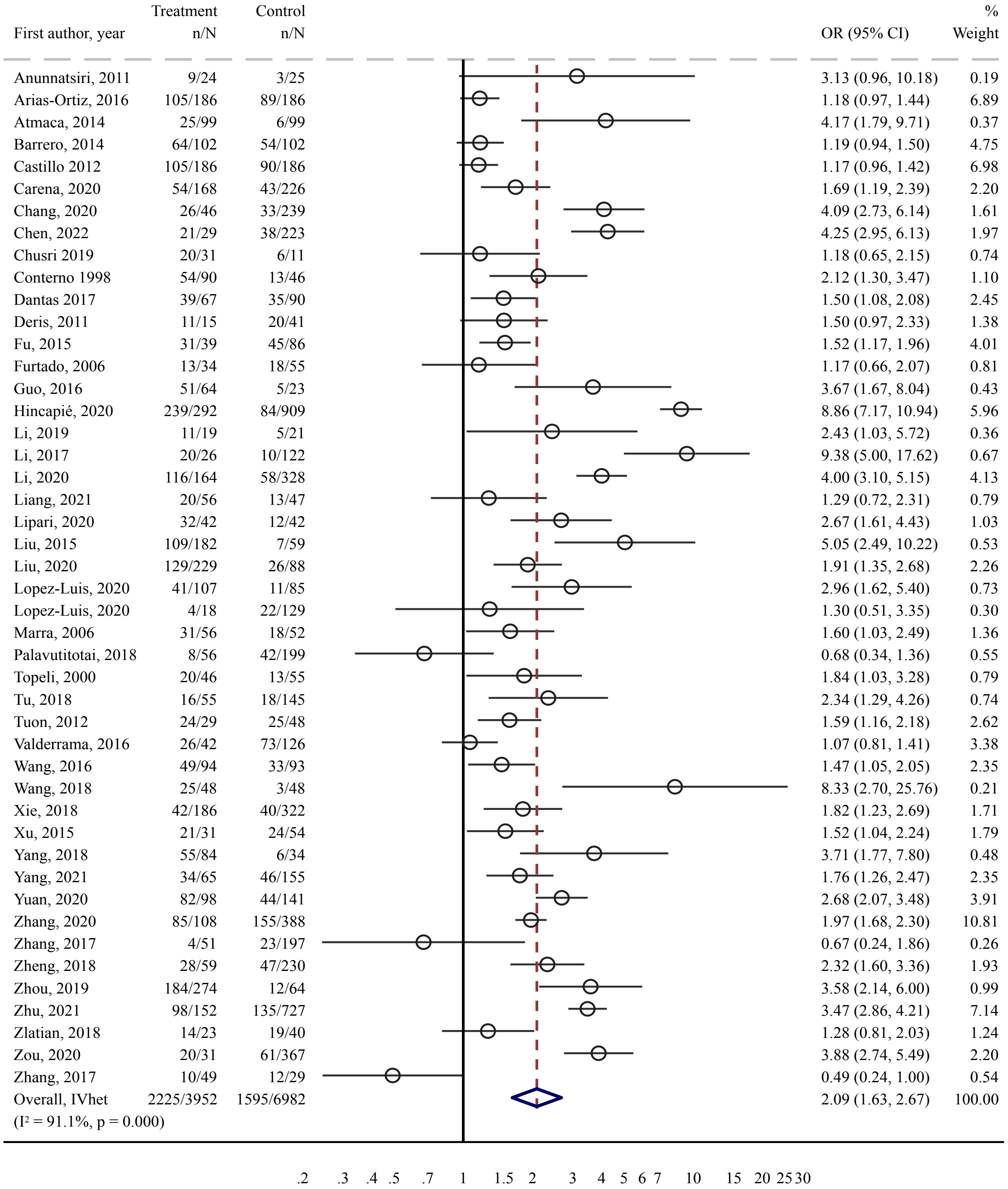

3.d) Odds ratios unadjusted and adjusted for mortality rates

Figs AD-AI report subgroup analysis for studies reporting either unadjusted or adjusted odds of mortality (the number of studies differs between these definitions). Figs AJ-AL report subgroup analysis for studies reporting both adjusted and unadjusted odds for mortality simulatenously (same number of studies per definition).

Fig AD. Subgroup analysis for studies reporting <u>unadjusted ORs</u> (N=25). Weights are from Doi's IVhet model. Dashed red line stands for the overall IVhet, whereas the black vertical line for OR=1. The number of studies differ from the number of studies providing unadjusted estimates, and viceversa. Estimate's p-value was 0.077.

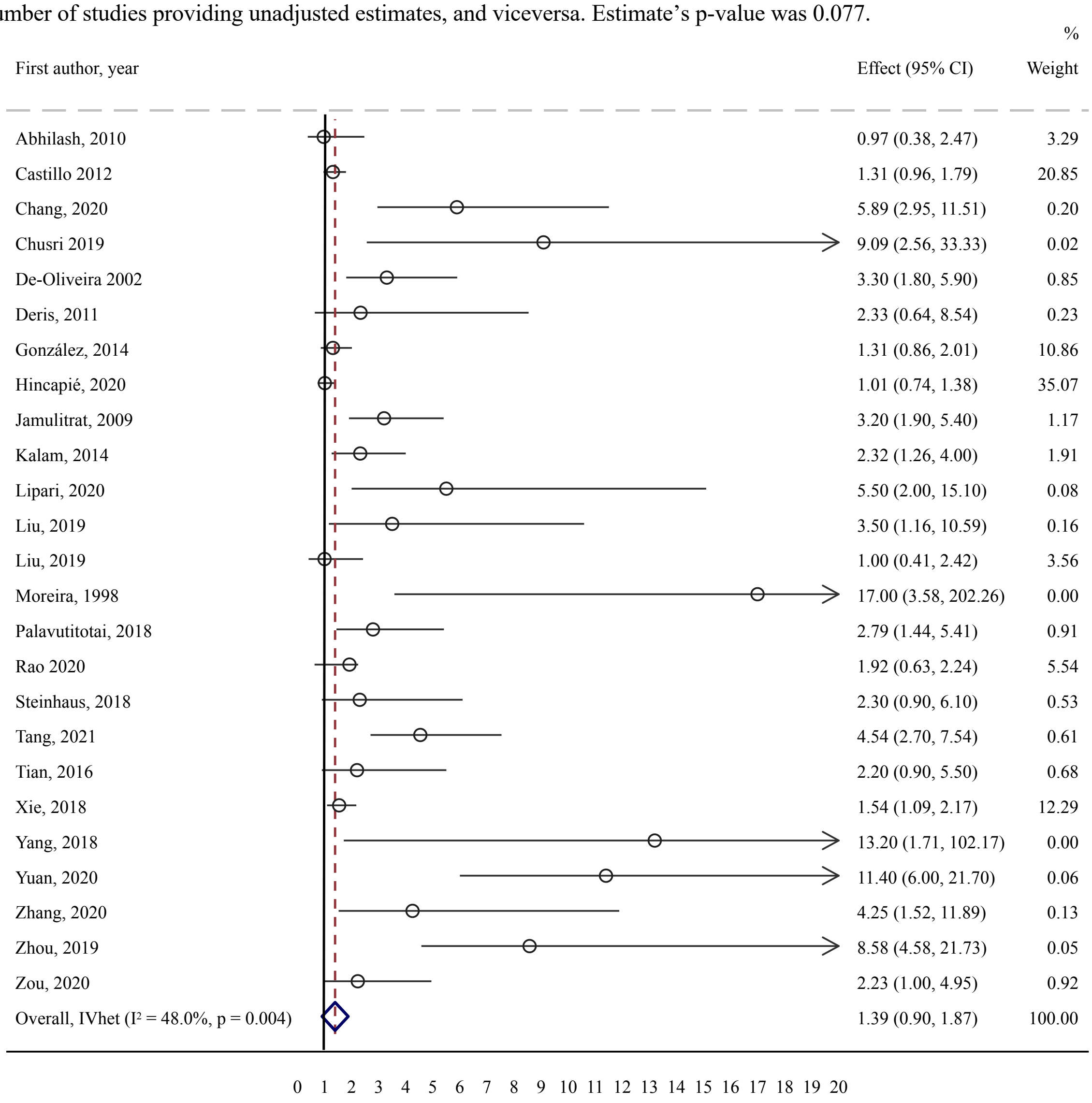

Fig AE. Subgroup analysis for studies reporting unadjusted ORs (N=26), by bacteria's gram type or WHO criticality category (critical=gram-negative, high-priority= gram-positive in this study). Weights are from Doi's IVhet model. Dashed red line stands for the overall IVhet, whereas the black vertical line for OR=1. High priority pathogens are also classified as gram-positive bacteria throughout the study. Critical priority pathogens are also classified as gram-negative bacteria in the present study. The number of studies differs from the number of studies providing unadjusted estimates and viceversa. Subgroup estimate's p-value were 0.355 and 0.503 for critical- and high-priority pathogens models, respectively. WHO= World Health Organization.

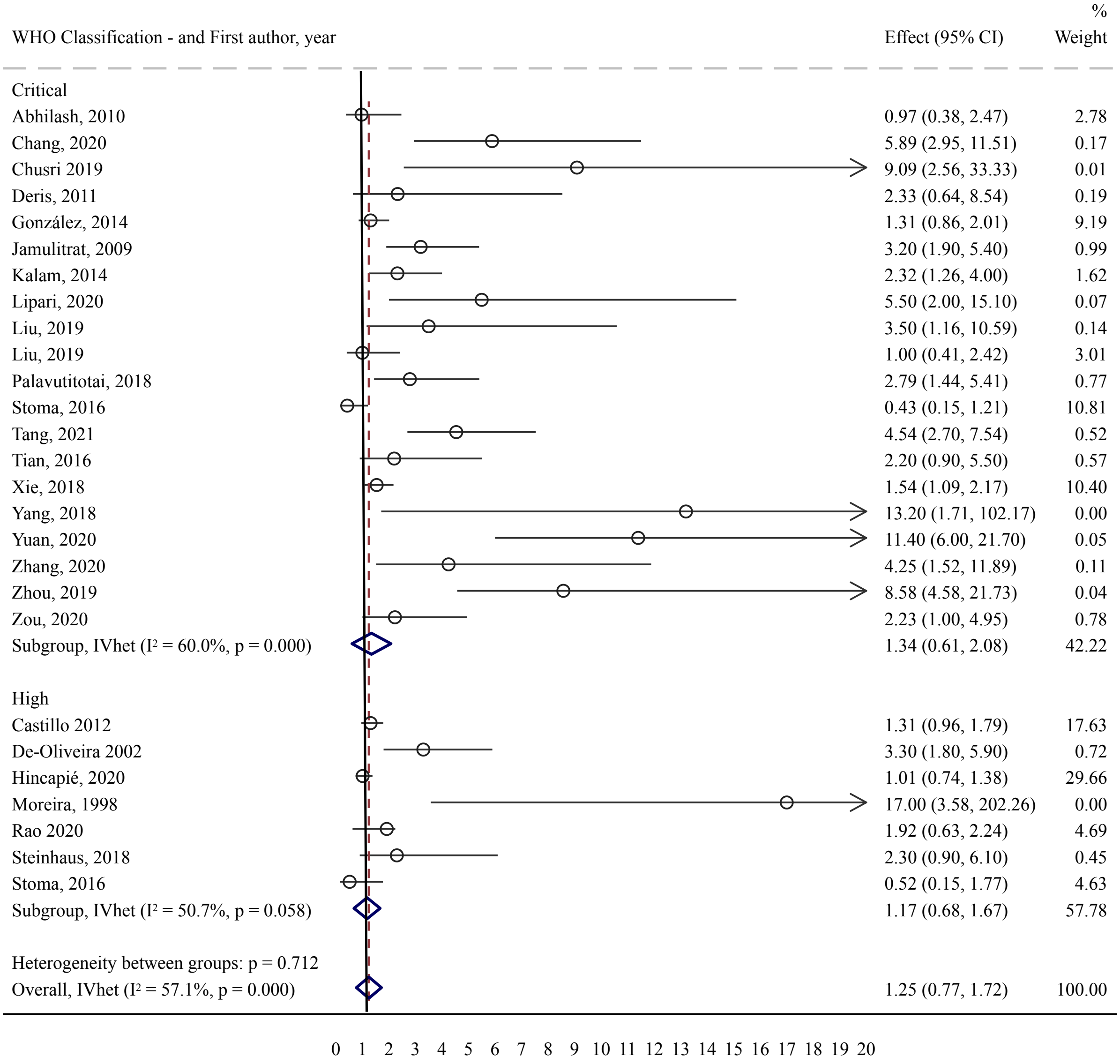

Fig AF. Subgroup analysis for studies reporting unadjusted ORs (N=23), by specific bacterium. Weights are from Doi's IVhet model. Dashed red line stands for the overall IVhet, whereas the black vertical line for OR=1. The number of studies differs from the number of studies providing unadjusted estimates and viceversa. Subgroup estimate's p-value were 0.733, <0.001, 0.005, 0.656, and 0.742 for Enterobacteriaceae, Moraxellaceae, Enterococcus spp., Staphylococcaceae and Pseudomonadaceae models, respectively.

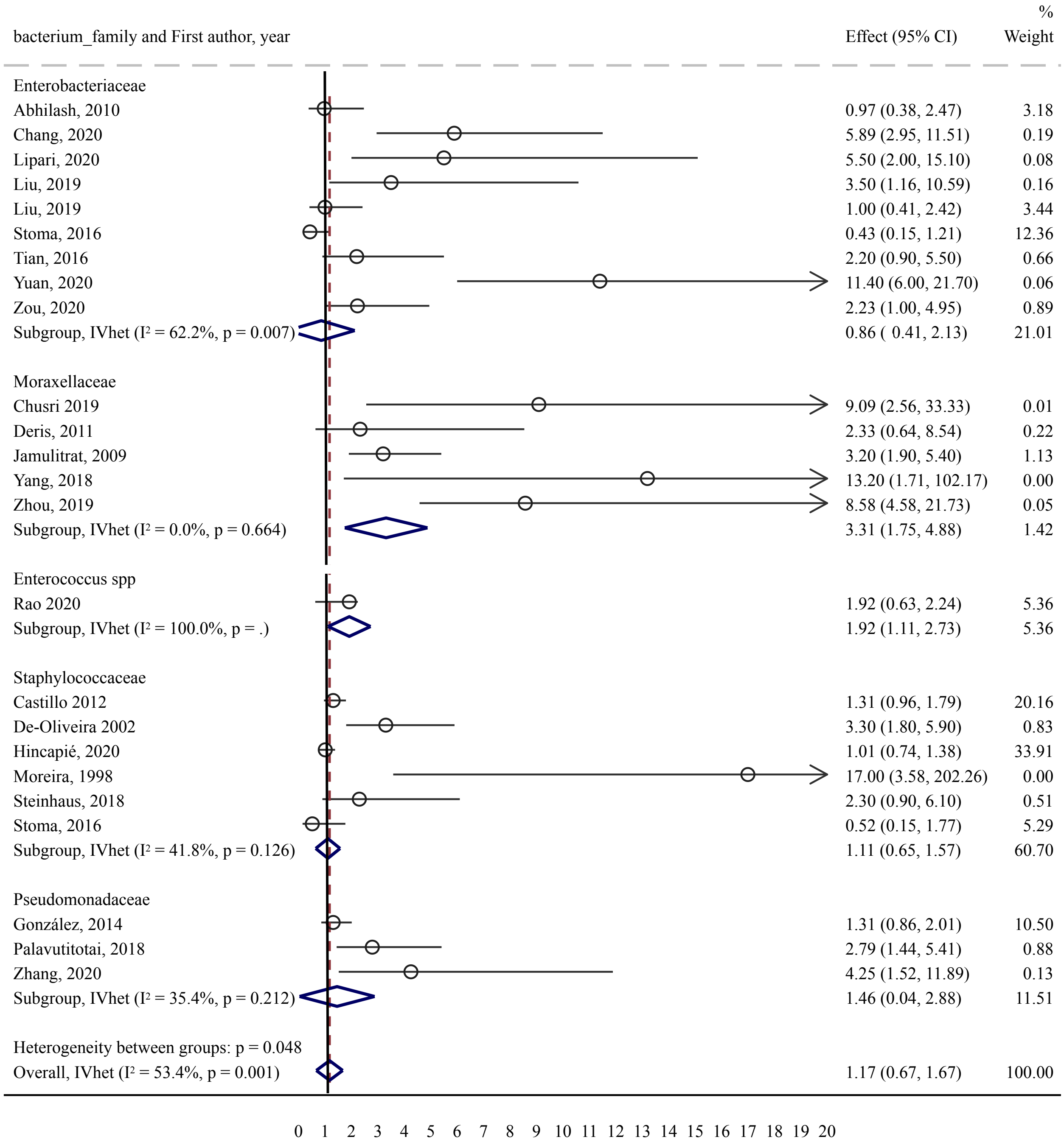

Fig AG. Subgroup analysis for studies reporting adjusted ORs (N=32). Weights are from Doi's IVhet model. Dashed red line stands for the overall IVhet, whereas the black vertical line for OR=1. The number of studies differs from the number of studies providing unadjusted estimates and viceversa. Estimate's p-value was <0.001.

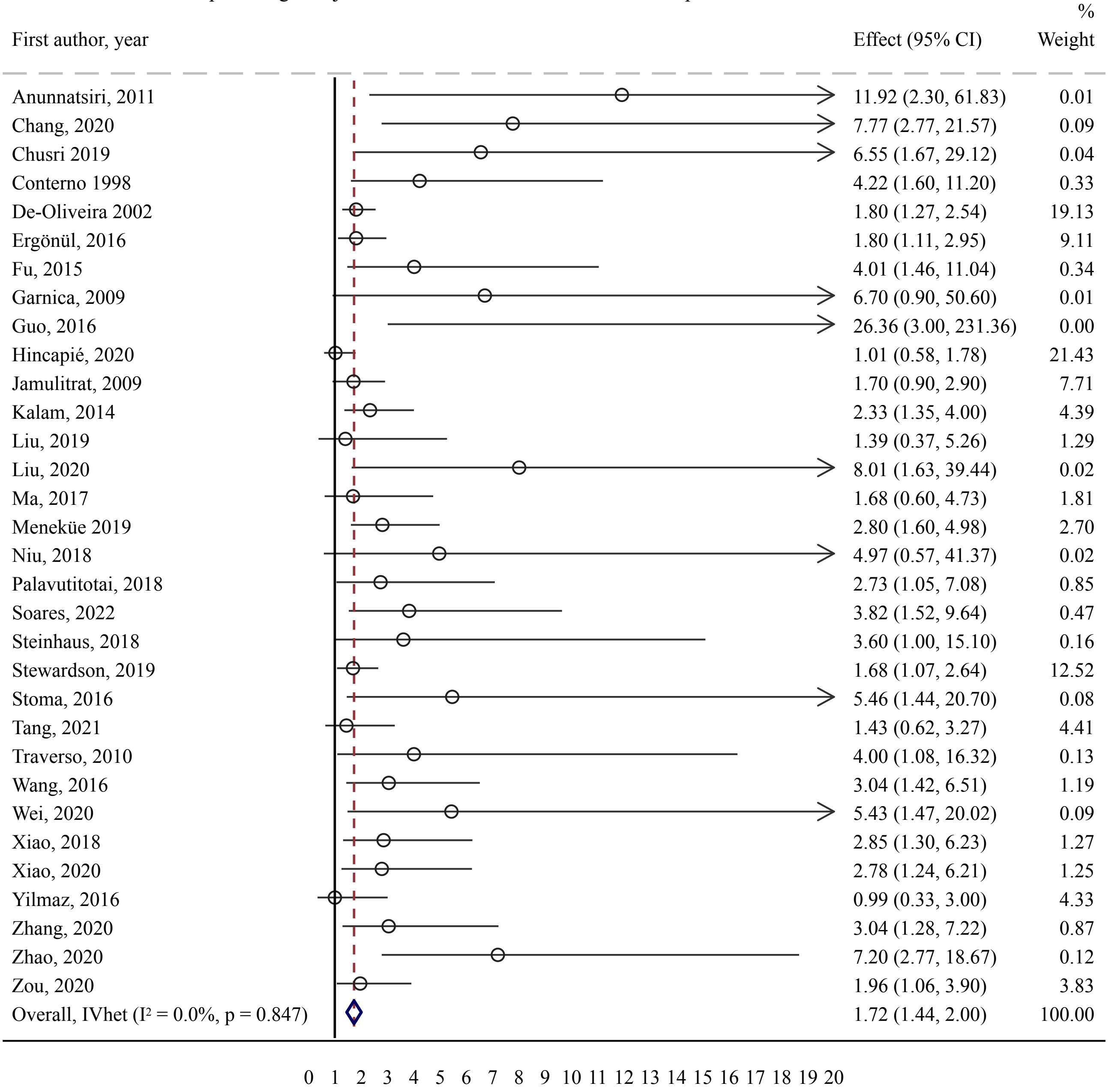

Fig AH. Subgroup analysis for studies reporting <u>adjusted ORs</u> (N=32), by bacteria's gram type (critical=gram-negative, high-priority= gram-positive in this study). Weights are from Doi's IVhet model. Dashed red line stands for the overall IVhet, whereas the black vertical line for OR=1. The number of studies differs from the number of studies providing unadjusted estimates and viceversa. Subgroup estimate's p-value were <0.001 and 0.091 for critical- and high-priority pathogen models, respectively. WHO= World Health Organization.

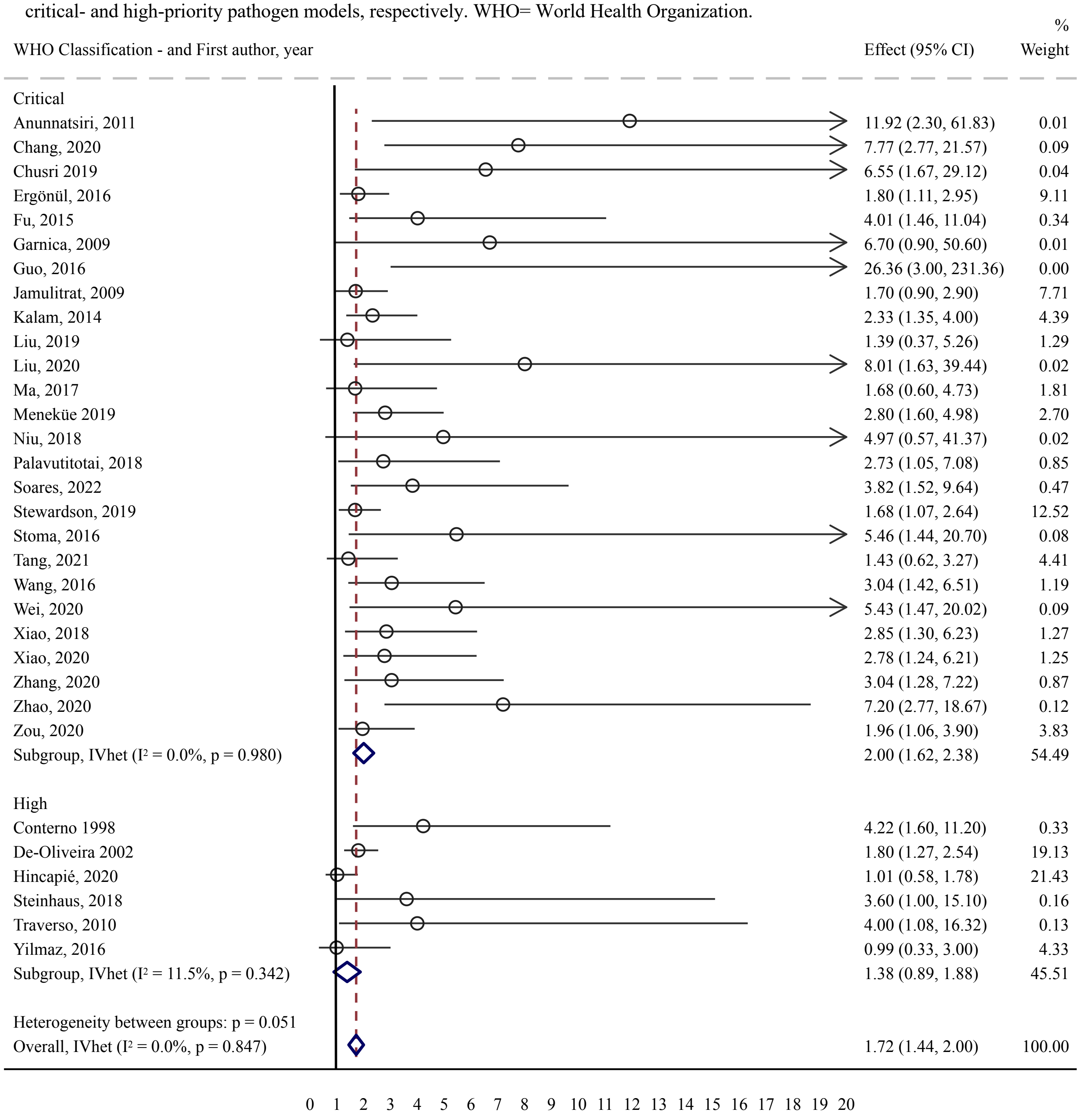

Fig AI. Subgroup analysis for studies reporting <u>adjusted ORs</u> (N=28), by specific bacterium. Weights are from Doi's IVhet model. Dashed red line stands for the overall IVhet, whereas the black vertical line for OR=1. One study combined Moraxellaceae and Pseusomnadaceae species altogether. The number of studies differs from the number of studies providing unadjusted estimates and viceversa. Subgroup estimate's p-value were <0.001, 0.037, 0.091, and 0.018 for Enterobacteriaceae, Moraxellaceae, Staphylococcaceae, and Pseudomonadaceae models, respectively.

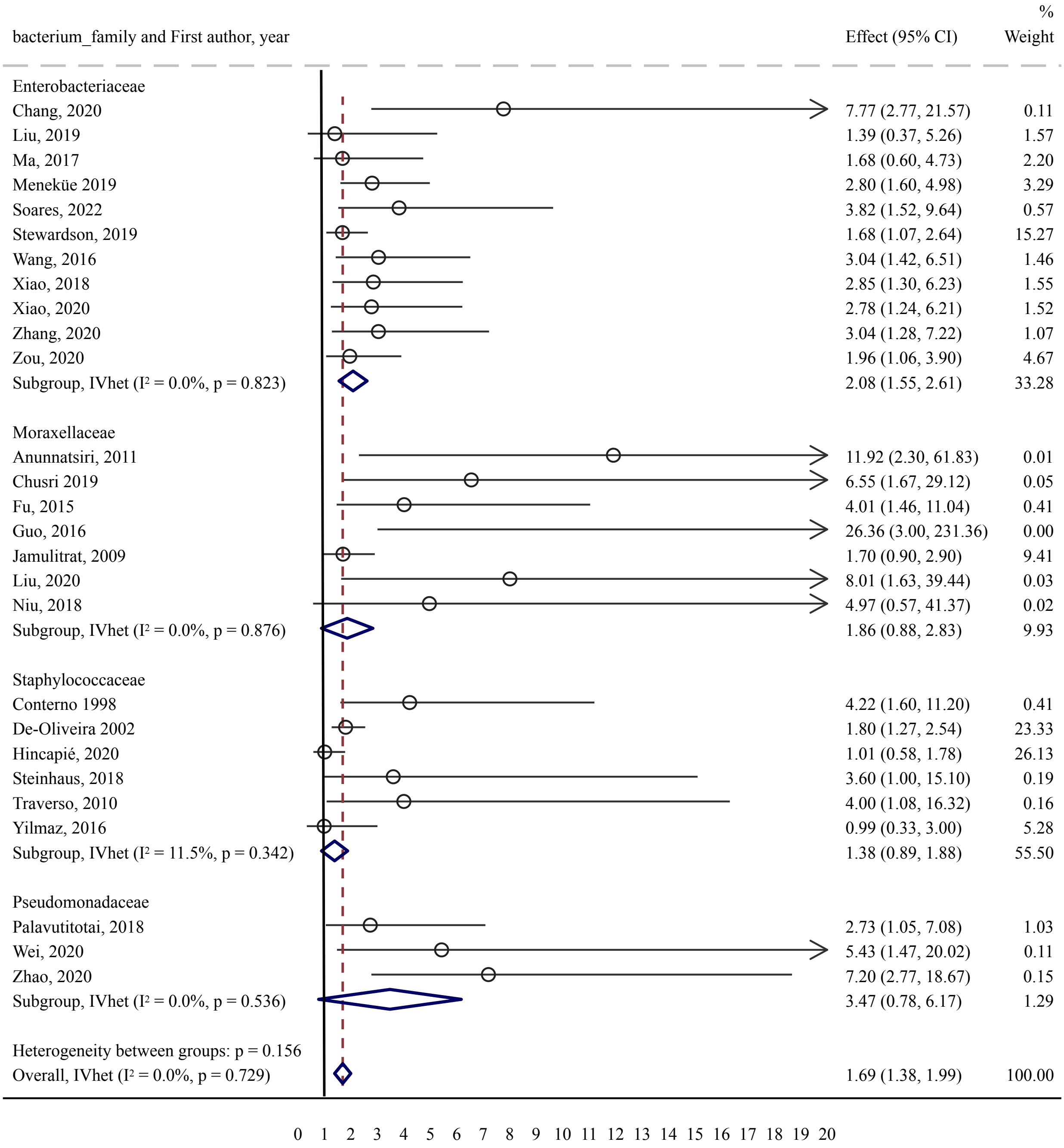

Fig AJ. Subgroup analysis for studies reporting <u>adjusted and unadjusted ORs simultaneously</u> (N=12), general mortality estimates. Weights are from Doi's IVhet model. Dashed red line stands for the overall IVhet, whereas the black vertical line for OR=1. One study combined Moraxellaceae and Pseusomnadaceae species altogether. Estimate's p-value were 0.486 and <0.001 for models A and B, respectively.

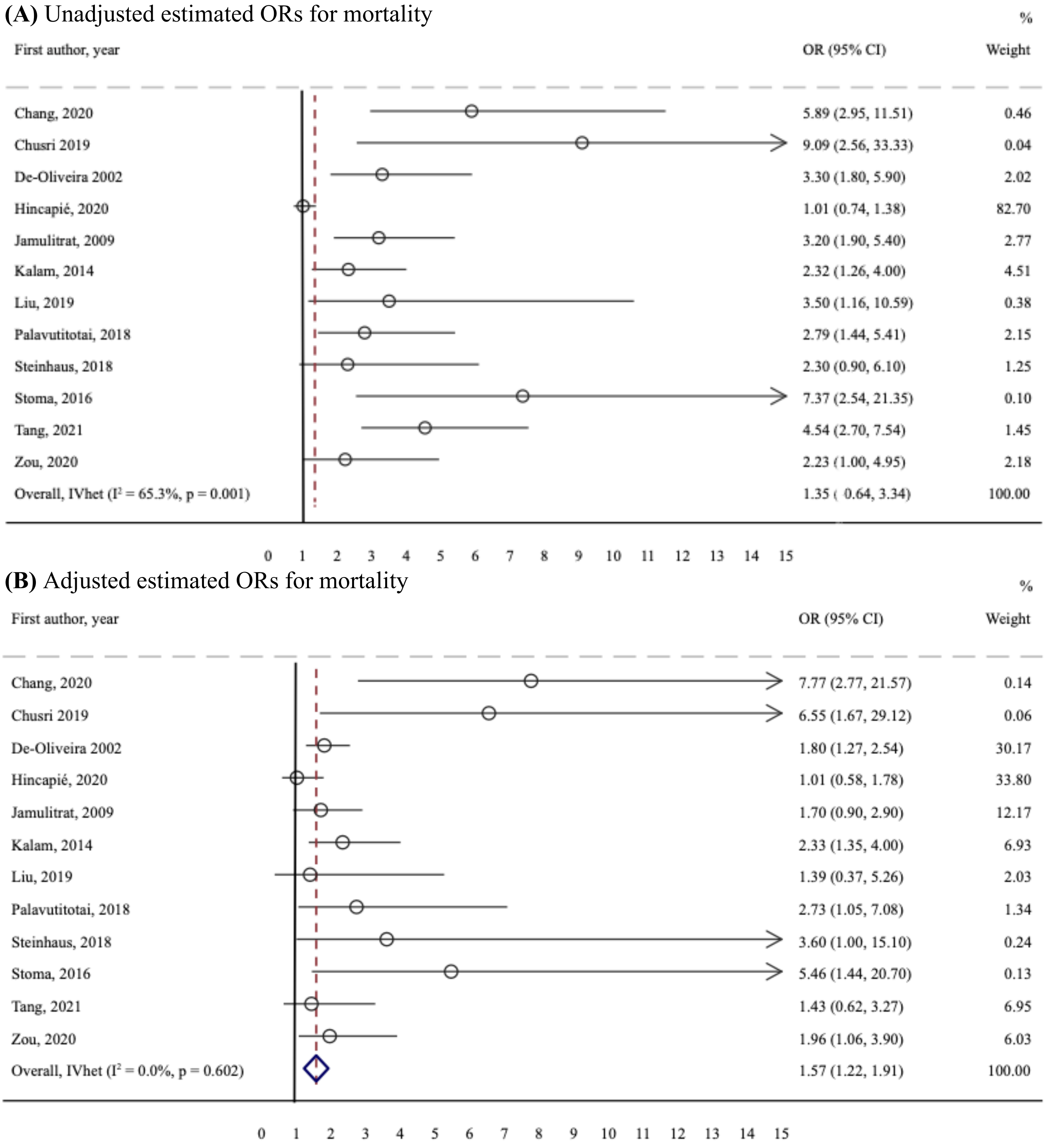

Fig AK. Subgroup analysis for studies reporting adjusted and unadjusted ORs simultaneously (N=12), mortality rates by gram-type or WHO criticality list classification (high= gram-positive, critical= gram-negative). Weights are from Doi's IVhet model. Dashed red line stands for the overall IVhet, whereas the black vertical line for OR=1. One study combined Moraxellaceae and Pseusomnadaceae species altogether. Estimate's p-value were <0.001 and 0.962 for models A's critical- and high-priority pathogen models, respectively. Estimate's p-value were <0.001 and 0.238 for models B's critical- and high-priority pathogen models, respectively. WHO= World Health Organization.

(A) Unadjusted estimated ORs for mortality

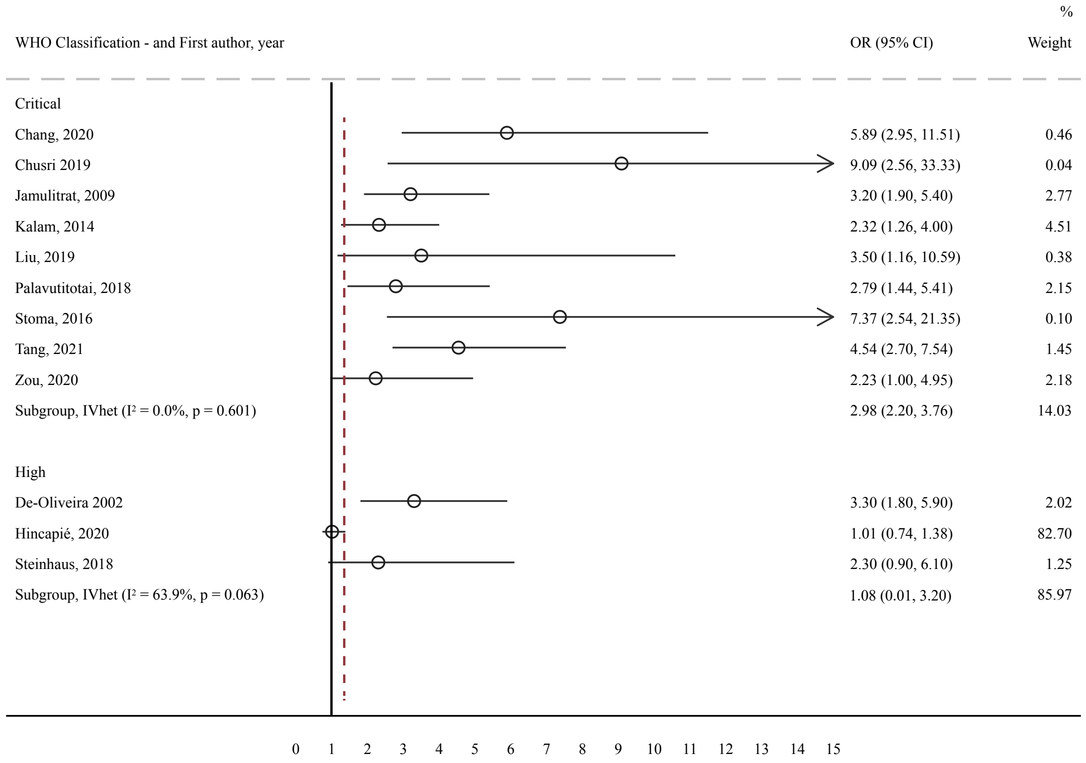

(B) Adjusted estimated ORs for mortality

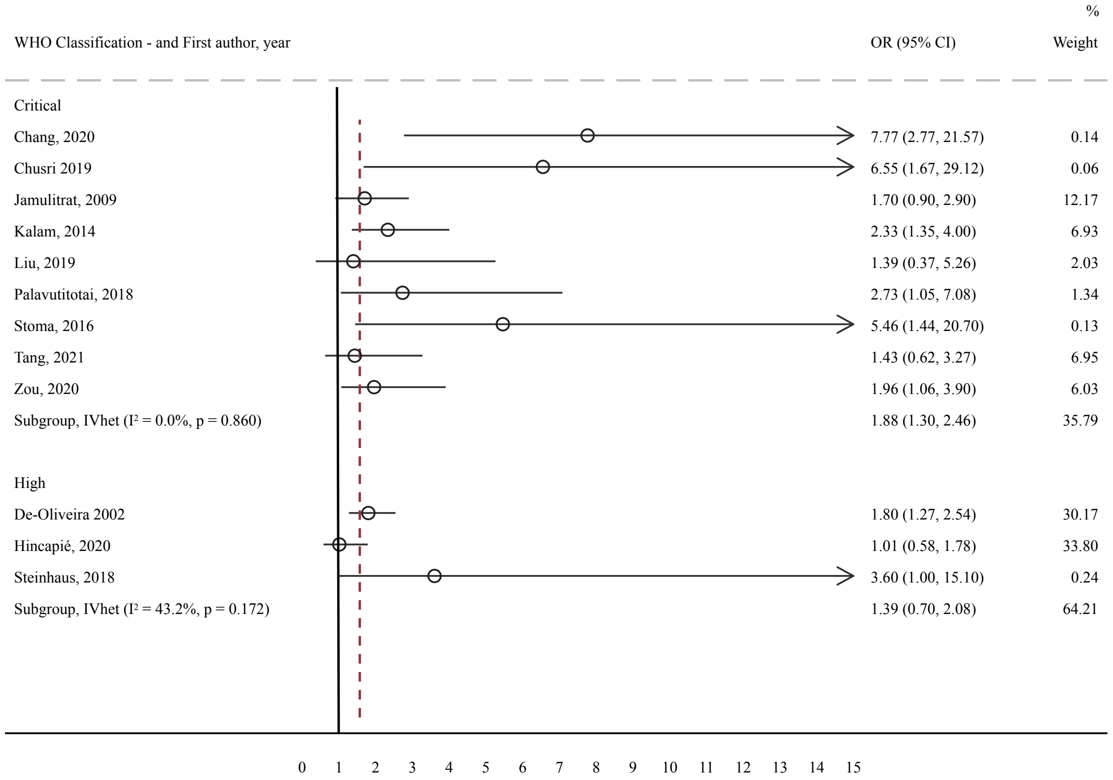

Fig AL. Subgroup analysis for studies reporting <u>adjusted and unadjusted ORs simultaneously</u> (N=10), mortality rates by bacterium family. Weights are from Doi's IVhet model. Dashed red line stands for the overall IVhet, whereas the black vertical line for OR=0. One study combined Moraxellaceae and Pseusomnadaceae species altogether. Estimate's p-value were 0.013, <0.001, 0.962, and 0.024 for models A's Enterobacteriaceae, Moaxellaceae, Staphylococcus, and Pseudomondaceae models, respectively. Estimate's p-value were 0.090, 0.102, 0.238, and 0.194 for models B's Enterobacteriaceae, Moaxellaceae, Staphylococcus, and Pseudomondaceae models, respectively.

(A) Unadjusted estimated ORs for mortality

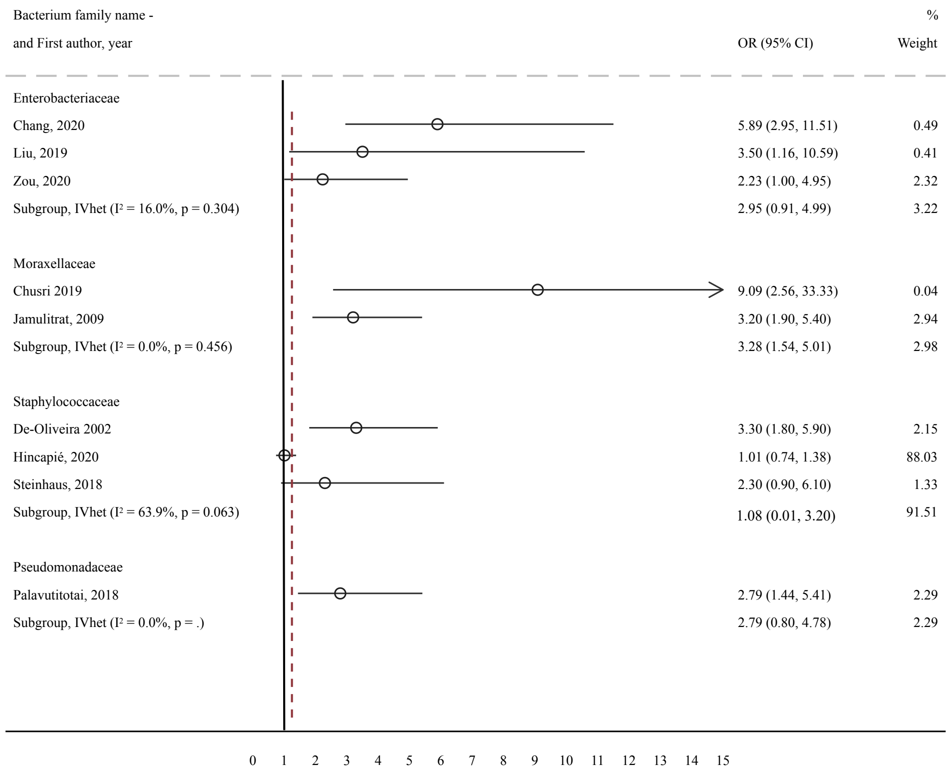

(B) Adjusted estimated ORs for mortality

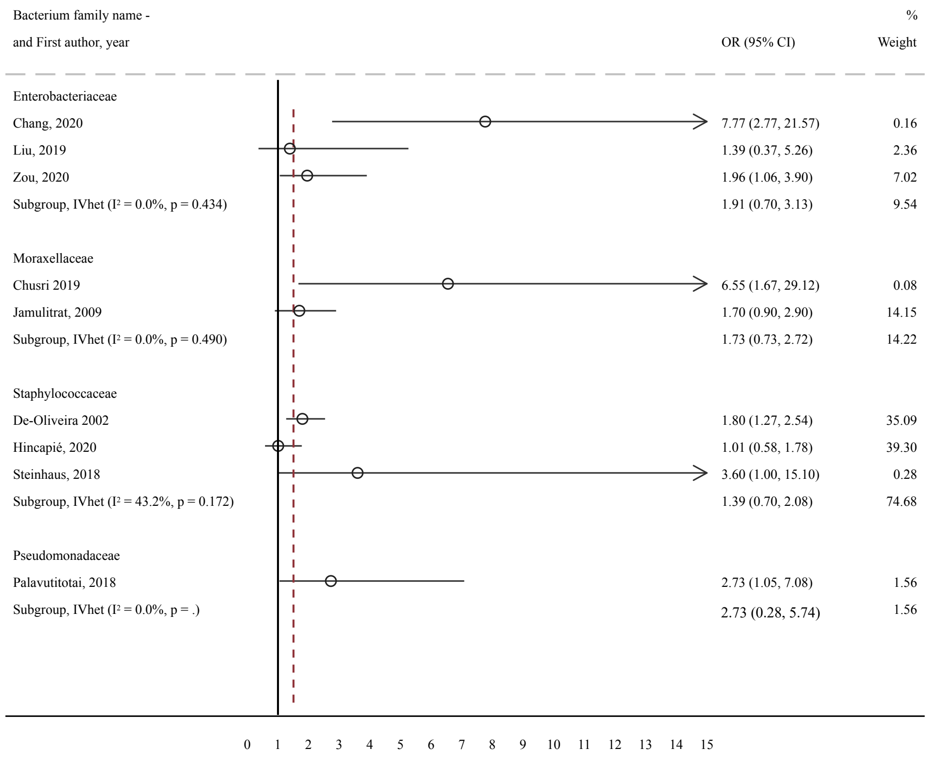

Table G. Summary of the subgroup meta-analysis results for income level and WHO region, by outcome variable

Outcome	Classification	Type	OR/SMD	Upper 95% CI	Lower 95% CI	Studies or sub-studies	p-value
Mortality	Income level	Low and Lower-middle income	1.30	1.11	1.49	11	<0.001
Mortality	Income level	Upper middle income	1.64	1.36	1.92	81	<0.001
Mortality	WHO region	Africa	1.56	0.95	2.17	3	0.035
Mortality	WHO region	Americas	1.49	0.89	2.08	19	0.065
Mortality	WHO region	Eastern Mediterranean	1.60	1.22	1.99	5	<0.001
Mortality	WHO region	Europe	1.79	1.49	2.11	8	<0.001
Mortality	WHO region	South-East Asia	1.33	1.06	1.59	10	0.006
Mortality	WHO region	Western Pacific	1.66	1.18	2.14	47	0.001
LOS	Income level	Low and Lower-middle income	0.25	-0.05	0.55	1	0.102
LOS	Income level	Upper middle income	0.51	0.19	0.82	17	0.002
LOS	WHO region	Africa	-	-	-	-	-
LOS	WHO region	Americas	0.43	0.02	0.83	3	0.037
LOS	WHO region	Eastern Mediterranean	-	-	-	-	-
LOS	WHO region	Europe	1.29	-0.33	2.92	3	0.120
LOS	WHO region	South-East Asia	0.24	0.07	0.42	3	0.007
LOS	WHO region	Western Pacific	0.39	0.17	0.61	9	<0.001
ICU	Income level	Low and Lower-middle income	1.20	0.65	2.22	6	0.572
ICU	Income level	Upper middle income	2.03	1.63	2.67	45	<0.001
ICU	WHO region	Africa	-	-	-	-	-
ICU	WHO region	Americas	1.77	1.08	2.89	13	0.023
ICU	WHO region	Eastern Mediterranean	1.53	0.55	4.23	4	0.422
ICU	WHO region	Europe	1.73	0.93	3.23	3	0.084
ICU	WHO region	South-East Asia	1.11	0.70	1.78	5	0.674
ICU	WHO region	Western Pacific	2.42	1.88	3.12	26	<0.001

ESBL: Extended-spectrum beta-lactamases. LOS: Length of hospital stay. ICU: Intensive Care Unit. Full disaggregated results, including their respective forest plots, are shown in S1 Text, section 3.

(4) Costs per hospital bed-day, mortality, ICU admission, and total costs per patient incurred

(4.1) Costs per hospital bed-day stay per patient according to the WHO-CHOICE

The WHO CHOICE model estimates the total costs (in 2008 USD adjusted to 2020 USD using US Gross Domestic Product GDP implicit price deflators) per hospital-bed stay and by country, specifically the 'hotel' component of hospital costs that includes personnel, capita, and food costs for inpatients. Costs related to drugs and treatments are excluded.

Briefly, we obtained the data for each LMICs included in the analysis per type of facility (primary, secondary, or teaching/tertiary hospital) as a first step (Table H). Subsequently, we multiplied those values (costs) by the respective average Standardised Mean Difference (SMD) value from the meta-analysis and its 95%CIs (the overall Standard Deviation was also incorporated into the multiplication) to compute the excess hospital stay produced by patients having bloodstream infections caused by antibiotic-resistant bacteria, compared to susceptible strains (Table I). Finally, we adjusted those estimates to 2020 USD using the US GDP implicit price deflators (see Table J). For analytical purposes, we used the costs estimated for teaching hospitals.

Table H. Costs of hospital bed-day per patient, and by country and hospital-level (in 2008 USD)

Country	Cost per bed day US$ by hospital level, in 2008 USD		
	Primary-level hospital	Secondary-level hospital	Teaching hospital
Argentina	$87.24	$91.02	$117.69
Belarus	$64.58	$67.37	$87.11
Brazil	$16.51	$17.23	$22.28
China	$30.42	$31.73	$41.03
Colombia	$52.10	$54.35	$70.28
Fiji	$36.08	$37.64	$48.67
India	$8.47	$8.83	$11.42
Indonesia	$18.62	$19.42	$25.11
Iran	$46.40	$48.41	$62.59
Lebanon	$72.78	$75.92	$98.17
Malaysia	$86.70	$90.45	$116.96
Mexico	$108.10	$112.78	$145.83
Romania	$102.08	$106.49	$137.70
Ethiopia	$1.98	$2.07	$2.68
South Africa	$55.26	$57.65	$74.55
Pakistan	$6.17	$6.43	$8.32
Thailand	$38.38	$40.04	$51.77
Turkey	$108.21	$112.88	$145.96

Values are expressed in 2008 United States Dollars (USD). Data extracted from the WHO-CHOICE website (http://www.who.int/choice/costs/en/).

Table I. Costs of total excess hospital bed-days per patient by country and hospital level using estimated SMD and their respective 95% CIs (in 2008 USD)

Country	Cost per bed-days US$ by hospital level, in 2008 USD					
	Primary-level hospital	95%CI	Secondary-level hospital	95%CI	Teaching/Tertiary hospital	95%CI
Argentina	837.0	341.6, 1332.4	873.3	356.4, 1390.1	1129.1	460.9, 1797.4
Belarus	619.6	252.9, 986.3	646.4	263.8, 1028.9	835.8	341.1, 1330.4
Brazil	158.4	64.7, 252.1	165.3	67.5, 263.1	213.8	87.2, 340.3
China	291.9	119.1, 464.6	304.4	124.3, 484.6	393.7	160.7, 626.6
Colombia	499.9	204, 795.7	521.4	212.8, 830.1	674.3	275.2, 1073.3
Fiji	346.2	141.3, 551	361.1	147.4, 574.9	466.9	190.6, 743.3
India	81.3	33.2, 129.4	84.7	34.6, 134.9	109.6	44.7, 174.4
Indonesia	178.6	72.9, 284.4	186.3	76, 296.6	240.9	98.3, 383.5
Iran	445.2	181.7, 708.6	464.5	189.6, 739.3	600.5	245.1, 955.9
Lebanon	698.3	285, 1111.5	728.4	297.3, 1159.5	941.9	384.4, 1499.3
Malaysia	831.8	339.5, 1324.1	867.8	354.2, 1381.4	1122.1	458, 1786.3
Mexico	1037.1	423.3, 1650.9	1082.0	441.6, 1722.4	1399.1	571.1, 2227.2
Romania	979.4	399.7, 1559	1021.7	417, 1626.4	1321.1	539.2, 2103
Ethiopia	19.0	7.8, 30.2	19.9	8.1, 31.6	25.7	10.5, 40.9
South Africa	530.2	216.4, 844	553.1	225.8, 880.5	715.2	291.9, 1138.6
Pakistan	59.2	24.2, 94.2	61.7	25.2, 98.2	79.8	32.6, 127.1
Thailand	368.2	150.3, 586.2	384.2	156.8, 611.5	496.7	202.7, 790.7
Turkey	1038.2	423.8, 1652.6	1083.0	442, 1723.9	1400.4	571.6, 2229.2

Values are expressed in 2008 United States Dollars (USD). Data were extracted from the WHO-CHOICE website (http://www.who.int/choice/costs/en/). Total costs were calculated using the average SMD from the meta-analysis and its 95%CIs which were multiplied by the overall Standard Deviation and the corresponding cost (by country and hospital level). Additionally, costs for primary- and secondary-level hospitals were also computed, but most studies reviewed were conducted in tertiary hospitals.

Table J. Costs of total excess hospital bed-days per patient and by country and hospital level using estimated SMD and their respective 95% CIs (adjusted to 2020 USD)

	Cost per bed-days US$ by hospital level, inflated in 2020 USD					
**Country**	Primary-level hospital	95%CI	Secondary-level hospital	95%CI	Teaching/Tertiary hospital	95%CI
Argentina	1006.1	410.6, 1601.5	1049.7	428.4, 1670.9	1357.2	554, 2160.5
Belarus	744.8	304, 1185.5	776.9	317.1, 1236.7	1004.6	410, 1599.1
Brazil	190.4	77.7, 303.1	198.7	81.1, 316.3	256.9	104.9, 409
China	350.8	143.2, 558.4	365.9	149.4, 582.5	473.2	193.1, 753.2
Colombia	600.8	245.2, 956.4	626.8	255.8, 997.7	810.5	330.8, 1290.2
Fiji	416.1	169.8, 662.3	434.1	177.2, 691	561.3	229.1, 893.5
India	97.7	39.9, 155.5	101.8	41.6, 162.1	131.7	53.8, 209.6
Indonesia	214.7	87.6, 341.8	224.0	91.4, 356.5	289.6	118.2, 461
Iran	535.1	218.4, 851.8	558.3	227.9, 888.7	721.8	294.6, 1149
Lebanon	839.3	342.6, 1336.1	875.5	357.4, 1393.7	1132.1	462.1, 1802.1
Malaysia	999.8	408.1, 1591.6	1043.1	425.8, 1660.4	1348.8	550.5, 2147.1
Mexico	1246.6	508.8, 1984.4	1300.6	530.9, 2070.4	1681.7	686.4, 2677.1
Romania	1177.2	480.5, 1873.9	1228.1	501.3, 1954.9	1588.0	648.2, 2527.8
Ethiopia	22.8	9.3, 36.3	23.9	9.7, 38	30.9	12.6, 49.2
South Africa	637.3	260.1, 1014.4	664.8	271.4, 1058.3	859.7	350.9, 1368.5
Pakistan	71.2	29, 113.3	74.2	30.3, 118	95.9	39.2, 152.7
Thailand	442.6	180.7, 704.6	461.8	188.5, 735	597.0	243.7, 950.4
Turkey	1247.9	509.3, 1986.5	1301.8	531.3, 2072.2	1683.2	687, 2679.4

Data calculated based on Table S4.2. Values were inflated to 2020 USD, producing a cumulative price increase of 20.21% over the period (cumulative rate of inflation between 2008-2020 using the US GDP implicit price deflators; https://fred.stlouisfed.org/series/GDPDEF).

(4.2) Mortality costs

We calculate the excess mortality costs based on the years of potential life lost (YPLL), years of potential productive life lost (YPPLL), and cost of productivity loss (CPL) definitions.

Therefore, the analyses adopted the life expectancy and human capital approaches. We calculate YPLL based on life expectancy and while the human capital approach is used to compute the CPL. The human capital approach places a monetary value on the loss due to poor health, disability, or mortality using the present value of the expected future earnings. The cost of productivity loss was calculated using the 2020 Gross Domestic Product (GDP) per capita per country. The standardised life expectancy at the age of death was extracted from the Global Burden of Disease study life tables [1]. We did not use country-specific life expectancy at birth, given that our study excluded children. Life expectancy might be underestimated for adults due to the large infant mortality rate in LMIC. In other words, studies' average age of death from ARB BSIs reported was used to calculate the standardised life expectancy at age of death following the Global Burden of Disease study life tables, which indicates that adult life expectancy are similar country to country and differences are not necessarily observed after accounting for their large infant mortality (i.e., we do not analyse children in the study; hence we do not expect life expectancy to vary too much by country).

Years of life lost from premature mortality (YPLL)
the calculation of time lost is based on the difference between the age at death and the standard life expectancy at that age. standard life expectancy represents the potential maximum life span of an individual at a given age who is not exposed to avoidable health risks or severe injuries and receives appropriate health services. Standard life expectancy is a time loss function on the age at death, quantifying the years lost due to early death.

The YPLL, and its CPL were estimated using the following formulas:

$$\mathrm{YPLL_c} = [(\text{1 additional death}) * (\text{Standardised life expectancy at the age of death})];$$

$$\mathrm{CPL_{YPLL}} = (\mathrm{YPLL_c}) * (\text{GDP per capita in 2020 } \mathrm{USD_c})$$

where c = country-specific information, the standardised life expectancy at the age of death was extracted from the literature (GBD study, life tables) [1, 2] based on the average age of patients from our included studies (extracted from the descriptive statistics of included studies; the average age of included studies' patients = 56.1). GDP per capita was extracted from the World Bank.

Costs of productivity loss (YPPLL)
The rationale for the human capital approach is that the withdrawal of an individual's labor due to premature death or permanent disability results in a loss to society of that individual's future production

The YPPLL, and its CPL were estimated using the following formulas:

$$\mathrm{YPPLL_c} = [(\text{1 additional death}) * (\text{retirement or maximum working age based on the OECD} - \text{average age of patients from included studies})];$$

$$\mathrm{CPL_{YPPLL}} = (\mathrm{YPPLL_c}) * (\text{GDP per capita in 2020 } \mathrm{USD_c}\ )$$

where c = country-specific information, the average age of patients was extracted from our included studies (extracted from the descriptive statistics of included studies; the average age of included studies' patients = 56.1). GDP per capita was extracted from the World Bank, and the retirement age was obtained from the OECD guidelines on working age (working age = 65 years)[3].

Interpretation and final adjustment of YPLL and YPPLL

We calculated excess costs related to years of life lost from premature mortality and productivity losses for a patient having ARB BSI.

Finally, mortality costs were discounted (discount rate=5%) using the Present Value formula PV= (FV*1/(1+ discount rate)^(n periods). Where FV stands for future values, which is equal to the total raw excess mortality

costs. 'n periods' are calculated by employing the difference between the age of retirement (65 years) and average age within our sampled studies (56.1 years), or by the life expectancy at age of death (=32.9). Then, the present value (PV) is calculated in Table L.

Assumptions

There is no statistically significant difference in age means between susceptible and resistant groups according to our descriptive statistics table. The average age was used as the observed age for calculating YPLL.

Table K. Calculation of YPLL, YPPLL and cost of productivity loss, by country

Country	**GDP per capita** (USD, 2020)	**YPLL** based on Life Expectancy at average age (LE) [1, 2]	**YPPLL** based on retirement age or working age population=65 years [3]
Argentina	8496	32.9	8.9
Belarus	6543	32.9	8.9
Brazil	6794	32.9	8.9
China	10409	32.9	8.9
Colombia	5307	32.9	8.9
Ethiopia	919	32.9	8.9
Fiji	4864	32.9	8.9
India	1910	32.9	8.9
Indonesia	3894	32.9	8.9
Iran	2746	32.9	8.9
Lebanon	5600	32.9	8.9
Malaysia	10161	32.9	8.9
Mexico	8655	32.9	8.9
Pakistan	1322	32.9	8.9
Romania	13047	32.9	8.9
South Africa	5742	32.9	8.9
Thailand	6991	32.9	8.9
Turkey	8561	32.9	8.9

YPLL= years of potential life lost. CPL= Cost of productivity loss. YPPLL= Years of potential productivity losses. Gross Domestic Product (GDP): Word Bank. GDP= Gross domestic product. LE= Life expectancy. USD= United States dollars.

Table L. Costs of year of potential life lost due to premature mortality by country using the LE at age of death and cost of productivity lossess (based on age of retirement), discounted

Country	CPL_{YPLL}	CPL_{YPPLL}
Argentina	56144	181070
Belarus	43235	139436
Brazil	44898	144800
China	68780	221823
Colombia	35070	113104
Ethiopia	6070	19578
Fiji	12624	40714
India	25733	82992
Indonesia	18148	58530
Iran	32142	103661
Lebanon	37004	119343
Malaysia	67142	216540
Mexico	57192	184450
Pakistan	8738	28180
Romania	86217	278059
South Africa	37941	122362
Thailand	46196	148986
Turkey	56571	182448

We used the Present value formula with a discount rate=5%. YPLL= years of potential life lost. CPL= Cost of producitivity loss. YPPLL= Years of potential productivity losses.

(4.3) Intensive care unit (ICU) costs per day and patient

Costs associated with intensive or critical care were collected from the literature as available. Costs were calculated per patient and daily. The costs of countries without any available information were estimated using the values from a tertiary hospital as reference (using the WHO-CHOICE approach) and consequently multiplied by a comparison ratio (ICU costs are estimated between three- to sevenfold those of general wards), in line with the literature [4-6]. We used the median 1:5 ratio between ICU and general ward costs. Whenever presented, national currencies were transformed to USD and therefore inflated to 2020 USD. The currency's value in time was adjusted by using the US GDP implicit price deflators. Finally, the excess cost of bloodstream infections caused by antimicrobial-resistant bacteria was calculated by multiplying the country-specific monetary costs by the excess of ICU admissions and the average length of stay at the ICU for the resistant strains (obtained from the estimates for ICU admission and the data collected for the meta-analysis on ICU length of stay). The average ICU hospital length of stay (LOS) was 11.66 days, as per the studies in our literature review suggest (see S1 Data).

Table M. Intensive care unit costs per patient (daily)

Country	Costs	Year	Study/Notes
Argentina	$250	2000	[7] Mean fixed total ICU costs / days at ICU
Belarus [*]	$2060	2020	-
Brazil	$934	2006	[8]
China	¥2860	1996	[9] Costs calculated using (total cost per patient per day)
Colombia	COP 576340	2004	[10]Average costs per group were calculated (weighted)
Ethiopia [*]	$8.04	2020	-
Fiji	FJD 577.8	2010	Inpatient costs at the ICU [11]
India	$255	2011	[12]
Indonesia	Rp. 501477	2011	[13]
Iran	$770	2017	[14] Costs were averaged accordingly
Lebanon [*]	$294.51	2020	-
Malaysia	$1324	2015	[15]
Mexico	MXN 63526	2010	[16]
Pakistan	PAK 57535	2013	[17]
Romania	€ 995.57	2017	[18]
South Africa	ZAR 22870	2016	[19]
Thailand	$589.7	2011	[20]
Turkey	TL 2992.43	2011	[21]

¥= Chinese yuan. COP= Colombian peso, USD or $: United States dollars, PAK: Pakistani rupee, €= Euros, MXN= Mexican peso, TL= Turkish lira, ZAR= South African Rand. [*] These countries values were imported using the ratio presented in the literature for ICU compared to general ward costs. Rp.= Indonesida Rupias. FJD= Fijian dollars. ICU= Intensive care unit.

Table N. Intensive care unit costs (per patient and daily) adjusted to 2020 USD

Country	Costs of ICU admission in 2020 USD, per patient and daily
Argentina	$375.74
Belarus	$523.55
Brazil	$1199.06
China	$1032.79
Colombia	$1662
Ethiopia	$16.1
Fiji	$358.20
India	$293.4
Indonesia	$57.70
Iran	$813.01
Lebanon	$590.05
Malaysia	$1445.75
Mexico	$4609.52
Pakistan	$502.85
Romania	$1199.45
South Africa	$1847.42
Thailand	$678.5
Turkey	$747.86

ICU= Intensive care unit. $ is for United States Dollars (USD) dollars. Values were inflated to 2020 USD, producing a cumulative price increase of 20.21% over the period (cumulative inflation rate between 2008-2020 using the US GDP implicit price deflators; https://fred.stlouisfed.org/series/GDPDEF).

Final Equation for ICU costs per patient

$$\text{ICU cost per patient} = (\text{ICU costs}_i \text{ per patient/day}^a) \times (\text{Average ICU LOS})$$

Where the average ICU LOS is 11.66, and 'i' is for each country. [a] These data were extracted from S1 Data. Moreover, ICU admission rate was 1.93 greater for resistant strains than susceptible, which indicates an excess of ICU admitted patients within the ratio 2:1. Therefore, our formula indicates exactly the excess costs per single patient. The final calculation is shown below.

Table O. Intensive care unit costs (per day/patient) adjusted to ICU LOS and reported in 2020 USD

Country	Final costs of ICU admission in 2020 USD per patient, adjusted to ICU LOS
Argentina	$4381.13
Belarus	$6104.59
Brazil	$13981.04
China	$12042.33
Colombia	$19378.92
Ethiopia	$187.73
Fiji	$4176.8
India	$3421.04
Indonesia	$672.78
Iran	$9479.70
Lebanon	$6879.98
Malaysia	$16857.45
Mexico	$53747.00
Pakistan	$5863.23
Romania	$13985.59
South Africa	$21540.92
Thailand	$7911.31
Turkey	$8720.05

ICU= Intensive care unit. LOS= Length of hospital stay. USD= United states dollars.

(4.4) Total excess costs incurred per patient

The excess costs incurred due to the patient's bloodstream infection caused by antibiotic-resistant bacteria are calculated as follows.

$$\text{Total excess costs per patient}_i = [\text{Excess cost of hospital stay adjusted to LOS, per patient}]_i + [\text{costs of potential life lost due to premature mortality, per patient}]_i + [\text{Excess cost of ICU admission adjusted to ICU LOS, per patient}]_i$$

Where 'i' stands for the ith country. Costs estimates were extracted as follows:

- $[\text{Excess cost of hospital stay adjusted to LOS, per patient}]_i$ **:** Data from Table J, teaching hospitals.
- $[\text{Costs of potential life lost due to premature mortality, per patient}]_i$ **:** Data from Table L.
- $[\text{Excess cost of ICU admission adjusted to ICU LOS, per patient}]_i$ **:** Data from Table O.

All costs are calculated based on the excess of the outcomes reported between a patient having bloodstream infections caused by antimicrobial resistant, compared to antimicrobial susceptible bacteria.

Table P. Total excess costs incurred for bloodstream infections caused by antibiotic-resistant bacteria in 2020 USD, per patient

Country	Total hospital-derived costs per patient (Hospital bed-day LOS + ICU admission LOS)	Total excess costs (including hospital-derived and YPLL[a] costs per person)	Total excess costs (including hospital-derived and YPPLL[a] costs per person)
Argentina	5,738	61,882	186,809
Belarus	7,109	50,344	146,545
Brazil	14,238	59,136	159,038
China	12,516	81,296	234,338
Colombia	20,189	55,259	133,293
Ethiopia	749	6,289	19,796
Fiji	4,309	17,362	45,452
India	3,711	29,286	86,545
Indonesia	1,395	19,111	59,492
Iran	10,612	42,343	113,862
Lebanon	8,229	45,016	127,355
Malaysia	18,539	85,348	234,746
Mexico	55,335	112,621	239,879
Pakistan	5,894	14,697	34,139
Romania	14,845	101,791	293,632
South Africa	21,637	60,341	144,763
Thailand	8,508	54,704	157,495
Turkey	10,403	66,975	192,851

$ is for 2020 United States Dollars (USD) dollars. [a] **Mortality costs calculated** using the years of potential life lost approach. ICU= Intensive care unit. LOS= Length of hospital stay.

(5) Meta-analysis statistics, by outcome and model type (subgroup)

Table Q. Statistics calculated for meta-analysis using mortality as an outcome, by model.

Model:	Stats	value	df, p-value; or 95%CI		Heterogeneitylevel
Model 1: General	Cochrane	295.44	93.00	p<0.001	
	H	1.78	1.44	2.13	
	I^2	68.50%	51.60%	77.90%	**M**
	tau^2	0.39			
	LFK index	5.06			
Model 2: Critical WHO	Cochrane	215.92	71.00	p<0.001	
	H	1.74	1.37	2.12	
	I^2	67.10%	46.40%	77.80%	**M**
	tau^2	0.36			
	LFK index	5.16			
Model 3: High WHO	Cochrane	85.77	25.00	p<0.001	
	H	1.85	1.16	2.55	
	I2	70.90%	25.70%	84.60%	**M**
	tau^2	0.48			
	LFK index	3.22			
Model 4: Enterobacteriaceae	Cochrane	158.65	38.00	p<0.001	
	H	2.07	1.42	2.62	
	I^2	74.90%	50.10%	85.40%	**M**
	tau^2	0.61			
	LFK index	4.73			
Model 5: Enterococcus spp	Cochrane	4.04	6.00	p=0.67	
	H	0.821	1.00	1.32	
	I^2	0.00%	0.00%	42.50%	**L**
	tau^2	0.00			
	LFK index	3.84			
Model 6: Moraxacellea	Cochrane	20.90	15	p=0.14	
	H	1.18	1.00	1.65	
	I^2	28.2%	0.00%	63.40%	**L**
	tau^2	0.12			
	LFK index	8.50			
Model 7: Pseudomonadaceae	Cochrane	15.87	9.00	p=0.07	
	H	1.33	1.00	1.96	
	I^2	43.30%	0.00%	73.80%	**M**
	tau^2	0.10			
	LFK index	2.98			
Model 8: Staphylococcaceae	Cochrane	79.48	16.00	p<0.001	
	H	2.23	1.13	3.33	
	I^2	79.90%	21.70%	91.00%	**H**
	tau^2	0.80			
	LFK index	3.76			

The heterogeneity was calculated using the I^2 statistics; I^2 values were classified as high (>75%), moderate (50-75%), and low (<50%) heterogeneity.

Table R. Statistics calculated for meta-analysis using ICU admission as an outcome, by model.

Model:	Stats	value	df, p-value; or; 95%CI		Heterogeneity level
Model 1: General	Cochrane	577.54	52.00	p<0.001	
	H	3.33	2.46	4.21	
	I^2	91.00%	83.40%	94.30%	H
	tau^2	0.33			
	LFK index	1.35			
Model 2: Critical WHO	Cochrane	270.37	37.00	p<0.001	
	H	2.70	1.89	3.51	
	I^2	86.30%	72.00%	91.90%	H
	tau^2	0.21			
	LFK index	1.03			
Model 3: High WHO	Cochrane	303.51	14.00	p<0.001	
	H	4.66	2.28	7.05	
	I2	95.40%	80.80%	98.00%	H
	tau^2	0.6803			
	LFK index	2.04			
Model 4: Enterobacteriaceae	Cochrane	109.95	17.00	p<0.001	
	H	2.54	1.45	3.64	
	I^2	84.50%	52.10%	92.50%	H
	tau^2	0.16			
	LFK index	0.02			
Model 5: Enterococcus spp	Cochrane	25.37	6.00	p<0.001	
	H	2.056	1.00	3.275	
	I^2	76.30%	0.00%	90.70%	H
	tau^2	0.271			
	LFK index	0.99			
Model 6: Moraxacellea	Cochrane	53.09	11.00	p<0.001	
	H	2.20	1.00	3.42	
	I^2	76.30%	0.00%	91.50%	H
	tau^2	0.20			
	LFK index	3.38			
Model 7: Pseudomonadaceae	Cochrane	10.25	4.00	p=0.04	
	H	1.60	1.00	2.71	
	I^2	61.00%	0.00%	87.50%	M
	tau^2	0.05			
	LFK index	2.15			
Model 8: Staphylococcaceae	Cochrane	273.30	7.00	p<0.001	
	H	6.248	2.31	10.275	
	I^2	97.40%	81.30%	99.10%	H
	tau^2	0.82			
	LFK index	2.38			

The heterogeneity was calculated using the I^2 statistics; I^2 values were classified as high (>75%), moderate (50-75%), and low (<50%) heterogeneity.

Table S. Statistics calculated for meta-analysis using length of stay at hospital as an outcome, by model.

Model:	Stats	value	df, p-value; or 95%CI		Heterogeneity Level
Model 1: General	Cochrane	176.20	17.00	p<0.001	
	H	3.22	2.01	4.43	
	I^2	90.40%	75.10%	94.90%	H
	tau^2	0.27			
	LFK index	0.45			
Model 2: Critical WHO	Cochrane	30.86	10.00	p=0.001	
	H	1.76	1.00	2.58	
	I^2	67.60%	0.00%	84.90%	M
	tau^2	0.06			
	LFK index	-1.27			
Model 3: High WHO	Cochrane	128.43	6.00	p<0.001	
	H	4.63	1.88	7.42	
	I2	95.30%	71.60%	98.20%	H
	tau^2	0.66			
	LFK index	2.23			
Model 4: Enterobacteriaceae	Cochrane	13.69	4.00	p=0.01	
	H	1.85	1.00	3.19	
	I^2	70.80%	0.00%	90.20%	M
	tau^2	0.60			
	LFK index	-2.11			
Model 5: Enterococcus spp	Cochrane	-	-	-	
	H	-	-	-	
	I^2	-	-	-	L
	tau^2	-			
	LFK index	0.99		-	
Model 6: Moraxacellea	Cochrane	1.45	2.00	p=0.49	
	H	0.85	1.00	1.64	
	I^2	0.00%	0.00%	62.90%	L
	tau^2	0.00			
	LFK index	-0.06			
Model 7: Pseudomonadaceae	Cochrane	0.52	1.00	p=0.469	
	H	0.72	1.00	1.62	
	I^2	0.00%	0.00%	62.10%	L
	tau^2	0			
	LFK index	-2.64			
Model 8: Staphylococcaceae	Cochrane	117.58	5.00	p<0.001	
	H	4.85	1.69	8.09	
	I^2	95.70%	64.90%	98.50%	H
	tau^2	0.78			
	LFK index	1.96			

The heterogeneity level was calculated using the I^2 statistics; I^2 values were classified as high (>75%), moderate (50-75%), and low (<50%) heterogeneity.

Fig AM. Doi plots for Model 1 (general) and by outcome based on Tables Q, R and S. Most traditional Funnel plots are presented in Fig AN. ES= Estimate.

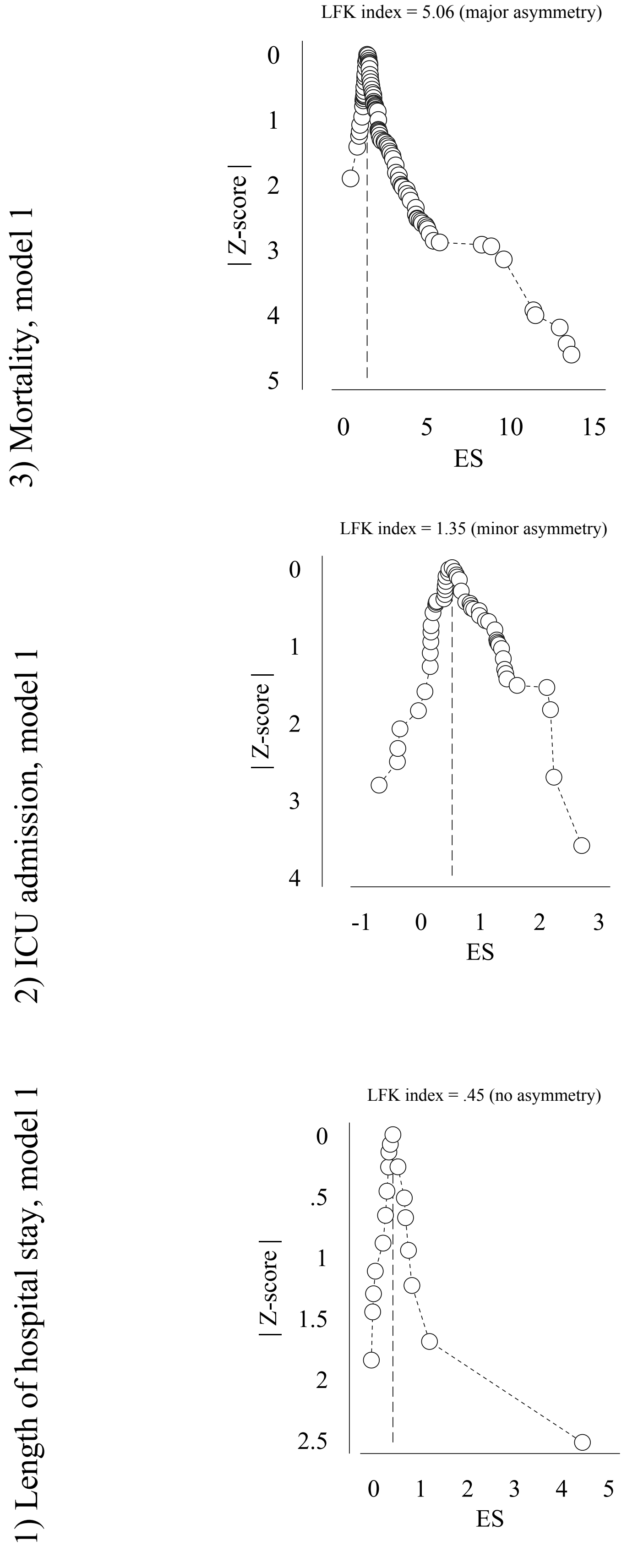

Fig AN. Funnel plots for Model 1 (general) and by outcome based on Tables Q, R and S. CI=Confidence Interval.

3) Mortality, model 1

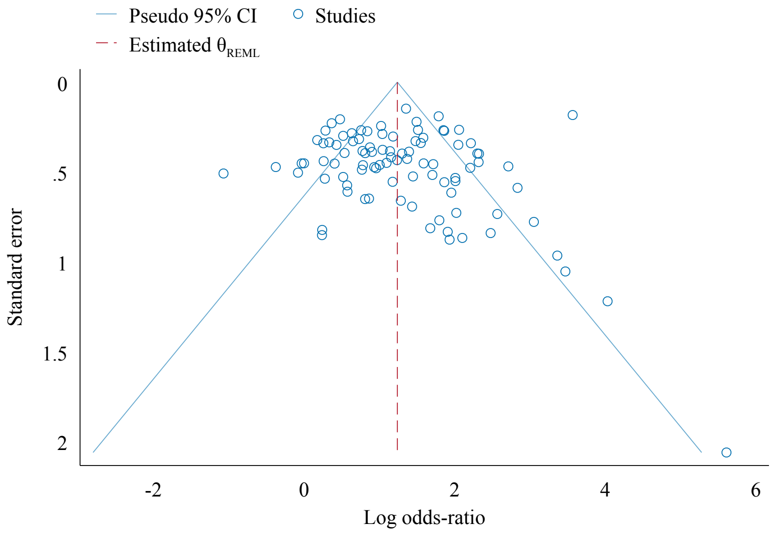

2) ICU admission, model 1

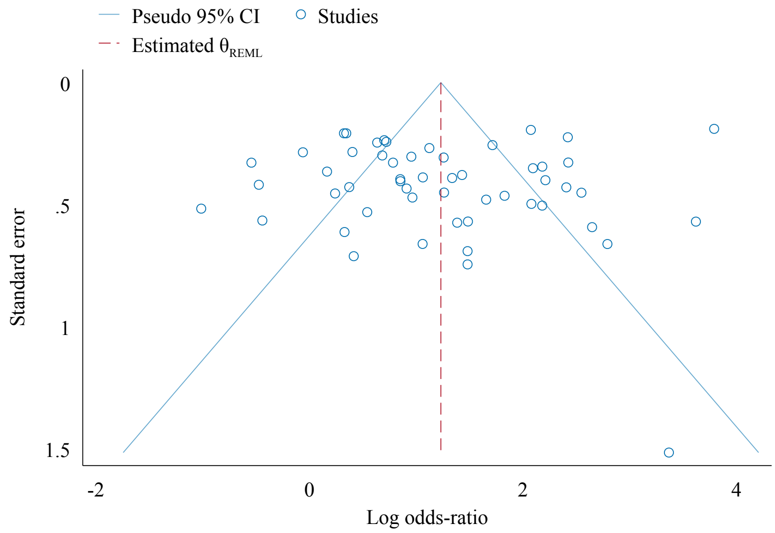

1) Length of hospital stay, model 1

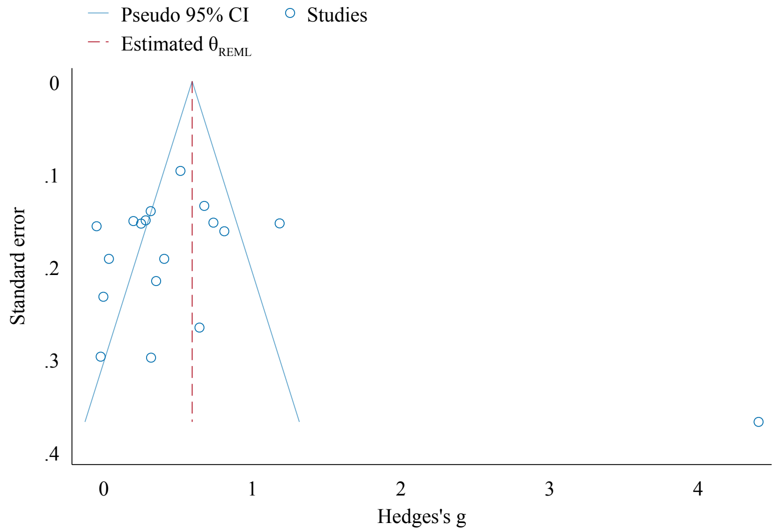

(6) Influence analyses: meta-analyses

Fig AO. Influence analysis for Model 1 using the mortality outcome (N=93) compared to the general estimates and without subgroup analyses. OR= Odds ratio, CI= Confidence Interval.

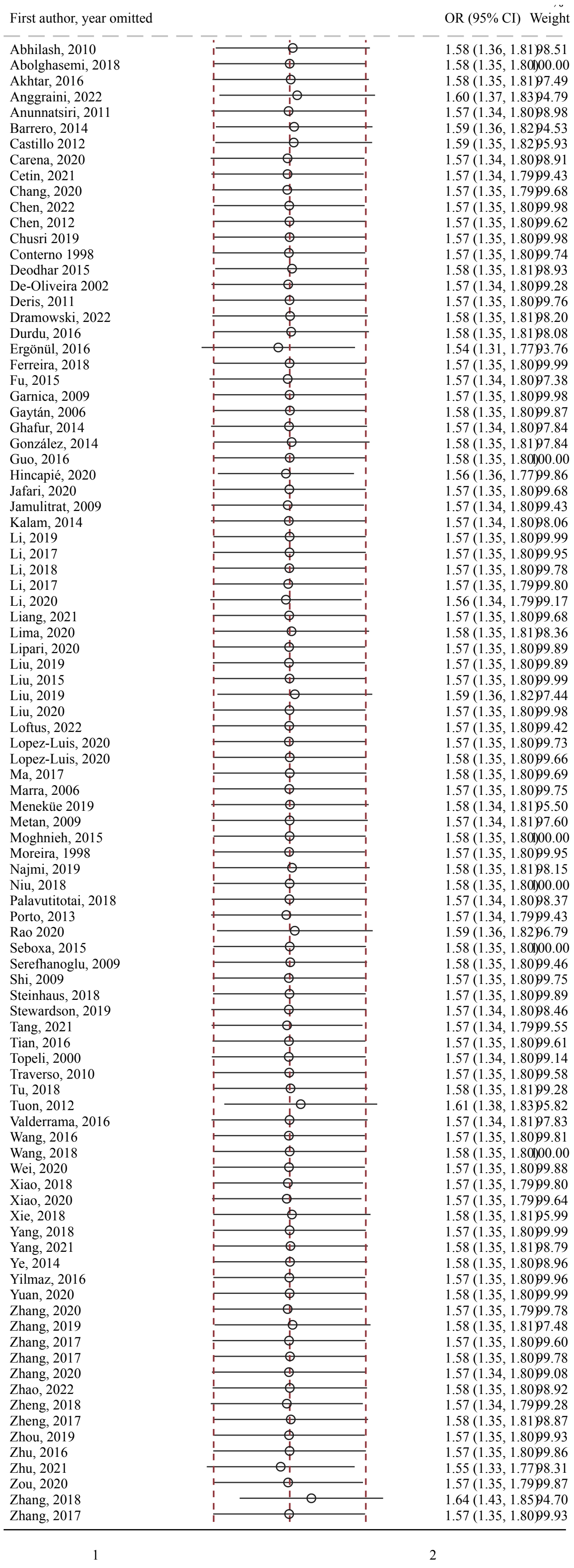

Fig AP. Influence analysis for Model 1 using the ICU admission outcome (N=52) compared to the general estimates and without subgroup analyses. OR= Odds ratio, CI= Confidence Interval.

First author, year omitted	Treatment n/N	Control n/N	OR (95% CI)	% Weight
Abolghasemi, 2018	2444.595/4434	1786.293/7540	1.96 (1.56, 2.46)	99.97
Akhtar, 2016	2429.595/4404	1777.293/7489	1.95 (1.55, 2.46)	99.49
Anggraini, 2022	2392.595/4378	1737.293/7482	2.03 (1.60, 2.56)	93.51
Anunnatsiri, 2011	2443.595/4426	1783.293/7529	1.96 (1.56, 2.47)	99.83
Arias-Ortiz, 2016	2347.595/4264	1697.293/7368	2.03 (1.60, 2.56)	93.97
Atmaca, 2014	2427.595/4351	1780.293/7455	1.96 (1.55, 2.46)	99.68
Barrero, 2014	2388.595/4348	1732.293/7452	2.00 (1.58, 2.54)	95.85
Castillo 2012	2347.595/4264	1696.293/7368	2.03 (1.60, 2.56)	93.90
Carena, 2020	2398.595/4282	1743.293/7328	1.97 (1.55, 2.49)	98.08
Chang, 2020	2426.595/4404	1753.293/7315	1.94 (1.54, 2.44)	98.59
Chen, 2022	2431.595/4421	1748.293/7331	1.93 (1.53, 2.44)	98.28
Chusri 2019	2432.595/4419	1780.293/7543	1.97 (1.56, 2.48)	99.35
Conterno 1998	2398.595/4360	1773.293/7508	1.96 (1.55, 2.47)	99.04
Dantas 2017	2413.595/4383	1751.293/7464	1.97 (1.56, 2.50)	97.86
Deris, 2011	2441.595/4435	1766.293/7513	1.97 (1.56, 2.48)	98.79
Fu, 2015	2421.595/4411	1741.293/7468	1.98 (1.56, 2.51)	96.50
Furtado, 2006	2439.595/4416	1768.293/7499	1.97 (1.56, 2.48)	99.29
Guo, 2016	2401.595/4386	1781.293/7531	1.96 (1.55, 2.46)	99.62
Hincapié, 2020	2213.595/4158	1702.293/6645	1.80 (1.49, 2.18)	94.78
Jafari, 2020	2423/4399	1781/7533	1.96 (1.56, 2.47)	99.61
Kalam, 2014	2420.595/4333	1750.293/7428	1.98 (1.57, 2.50)	98.58
Li, 2019	2441.595/4431	1781.293/7533	1.96 (1.56, 2.47)	99.68
Li, 2017	2432.595/4424	1776.293/7432	1.94 (1.55, 2.44)	99.42
Li, 2020	2336.595/4286	1728.293/7226	1.91 (1.51, 2.41)	96.39
Liang, 2021	2432.595/4394	1773.293/7507	1.97 (1.56, 2.48)	99.31
Lipari, 2020	2420.595/4408	1774.293/7512	1.95 (1.55, 2.47)	99.10
Liu, 2015	2343.595/4268	1779.293/7495	1.95 (1.55, 2.46)	99.53
Liu, 2020	2323.595/4221	1760.293/7466	1.96 (1.55, 2.48)	98.02
Lopez-Luis, 2020	2411.595/4343	1775.293/7469	1.96 (1.55, 2.46)	99.36
Lopez-Luis, 2020	2448.595/4432	1764.293/7425	1.96 (1.56, 2.47)	99.74
Marra, 2006	2421.595/4394	1768.293/7502	1.97 (1.56, 2.48)	98.81
Palavutitotai, 2018	2444.595/4394	1744.293/7355	1.97 (1.57, 2.48)	99.52
Rao 2020	2431.595/4377	1745.293/7454	1.99 (1.58, 2.50)	98.75
Stewardson, 2019	2398.595/4327	1735.293/7380	1.97 (1.56, 2.50)	97.51
Topeli, 2000	2432.595/4404	1773.293/7499	1.96 (1.56, 2.47)	99.31
Tu, 2018	2436.595/4395	1768.293/7409	1.96 (1.55, 2.47)	99.35
Tuon, 2012	2428.595/4421	1761.293/7506	1.97 (1.56, 2.49)	97.71
Valderrama, 2016	2426.595/4408	1713.293/7428	2.00 (1.58, 2.52)	97.05
Wang, 2016	2403.595/4356	1753.293/7461	1.97 (1.56, 2.50)	97.94
Wang, 2018	2427.595/4402	1783.293/7506	1.96 (1.55, 2.46)	99.82
Xie, 2018	2410.595/4264	1746.293/7232	1.96 (1.55, 2.48)	98.50
Xu, 2015	2431.595/4419	1762.293/7500	1.97 (1.56, 2.49)	98.43
Yang, 2018	2397.595/4366	1780.293/7520	1.96 (1.55, 2.46)	99.58
Yang, 2021	2418.595/4385	1740.293/7399	1.96 (1.55, 2.49)	97.94
Yuan, 2020	2370.595/4352	1742.293/7413	1.94 (1.53, 2.46)	96.58
Zhang, 2020	2367.595/4342	1631.293/7166	1.96 (1.55, 2.48)	90.55
Zhang, 2017	2448.595/4399	1763.293/7357	1.97 (1.56, 2.47)	99.78
Zheng, 2018	2424.595/4391	1739.293/7324	1.95 (1.55, 2.47)	98.31
Zhou, 2019	2268.595/4176	1774.293/7490	1.95 (1.55, 2.46)	99.13
Zhu, 2021	2354.595/4298	1651.293/6827	1.89 (1.50, 2.38)	93.75
Zlatian, 2018	2438.595/4427	1767.293/7514	1.97 (1.56, 2.49)	98.91
Zou, 2020	2432.595/4419	1725.293/7187	1.93 (1.53, 2.44)	98.08
Zhang, 2017	2442.595/4401	1774.293/7525	1.97 (1.57, 2.48)	99.53

(I² = 91.0%, p = 0.000)

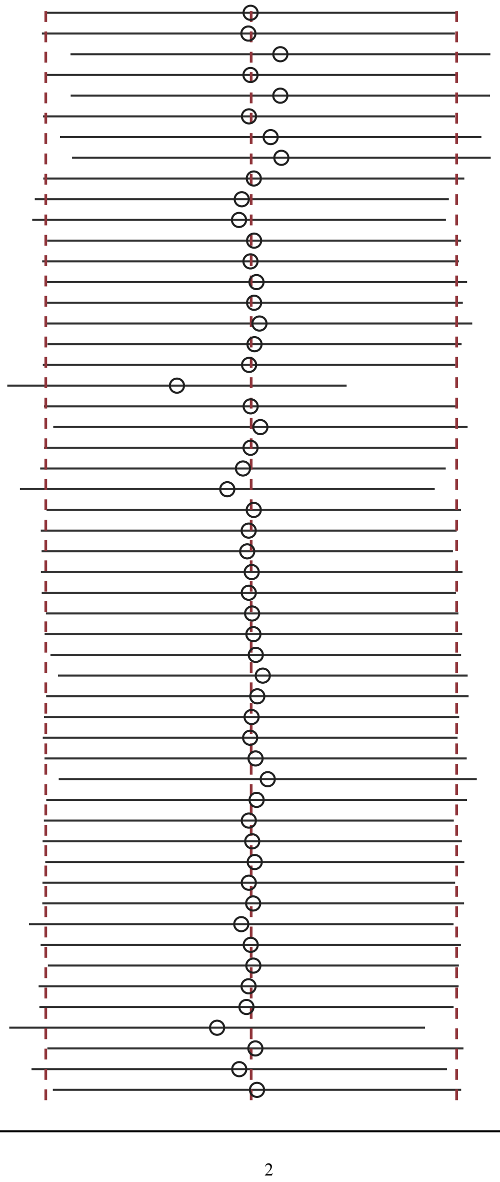

Fig AQ. Influence analysis for Model 1 using the length of hospital stay outcome (N=18) compared to the general estimates and without subgroup analyses. SMD= Standardised mean difference, CI= Confidence Intervals.

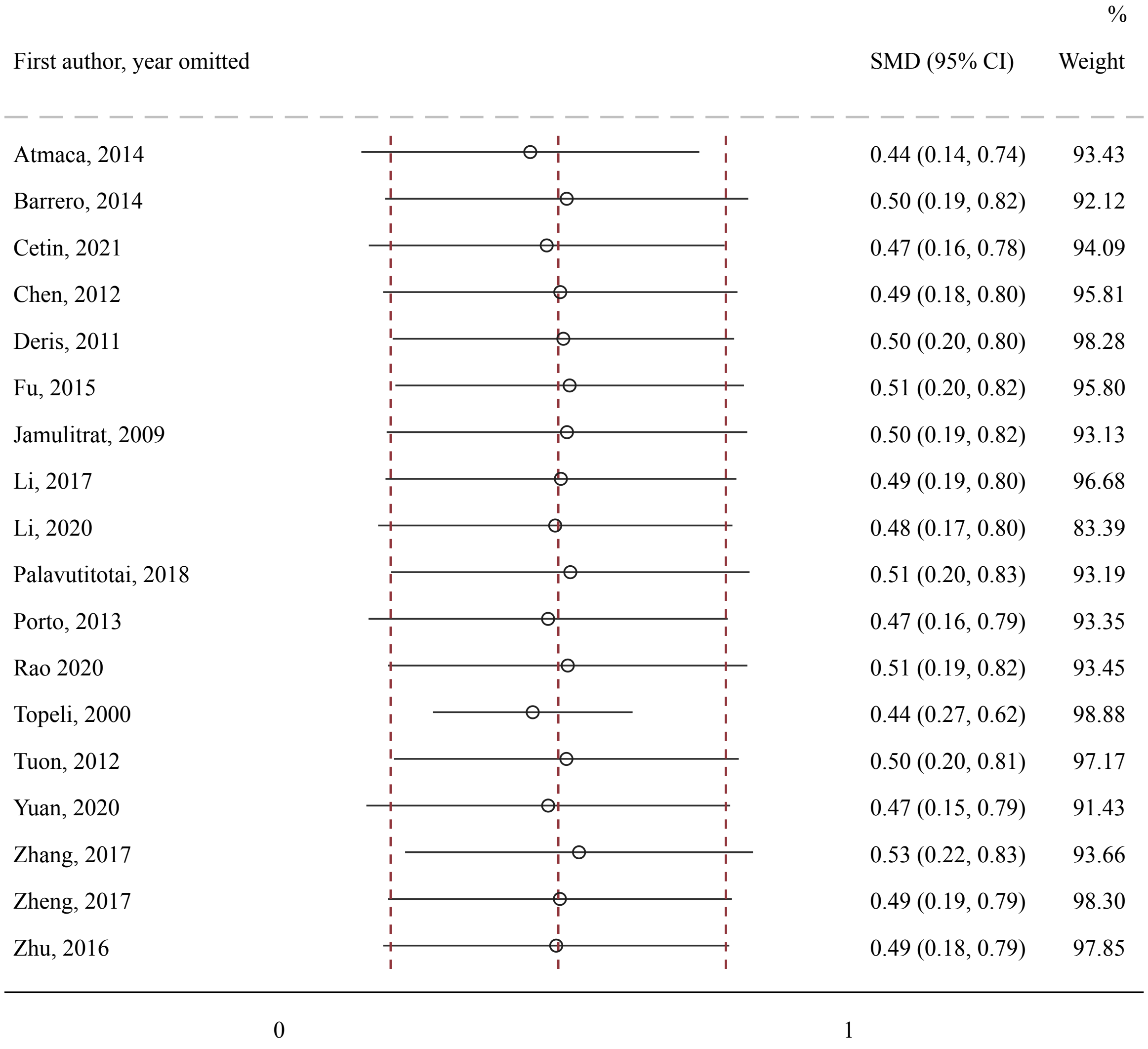

(7) Additional analyses

(7.a) Meta-analysis results dissagregated by prioritised antibiotic-bacterium specific pathogens according to the WHO

Fig AR. Meta-analysis results disaggregated by specific and prioritised antibiotic-bacterium pairs for mortality (N=56 studies). Weights are from Doi's IVhet model. Dashed red line stands for the overall IVhet, whereas the black vertical line for OR=1. CRAB= Carbapenem-resistant Acinetobacter baumanii, CREN= Carbapenem-resistant Enterobactericiae, CREC= Carbanpenem-resistant Escherichia coli, CRKP= Carbapenem-resistant Klebsiella pneumoniae, CRPA= Carbapenem-resistant Pseudomonas auregionsa, MRSA= Methicilin-resistant Staphylococcus aureus, VRE= Vancomycin-resistant Enterococcus faecium/faecalis. Estimates' p-value were 0.126, 0.592, 0.002, 0.092, 0.138, and 0.021 for CRAB, CREC, CRKP, CRPA, MRSA and VRE models, respectively.

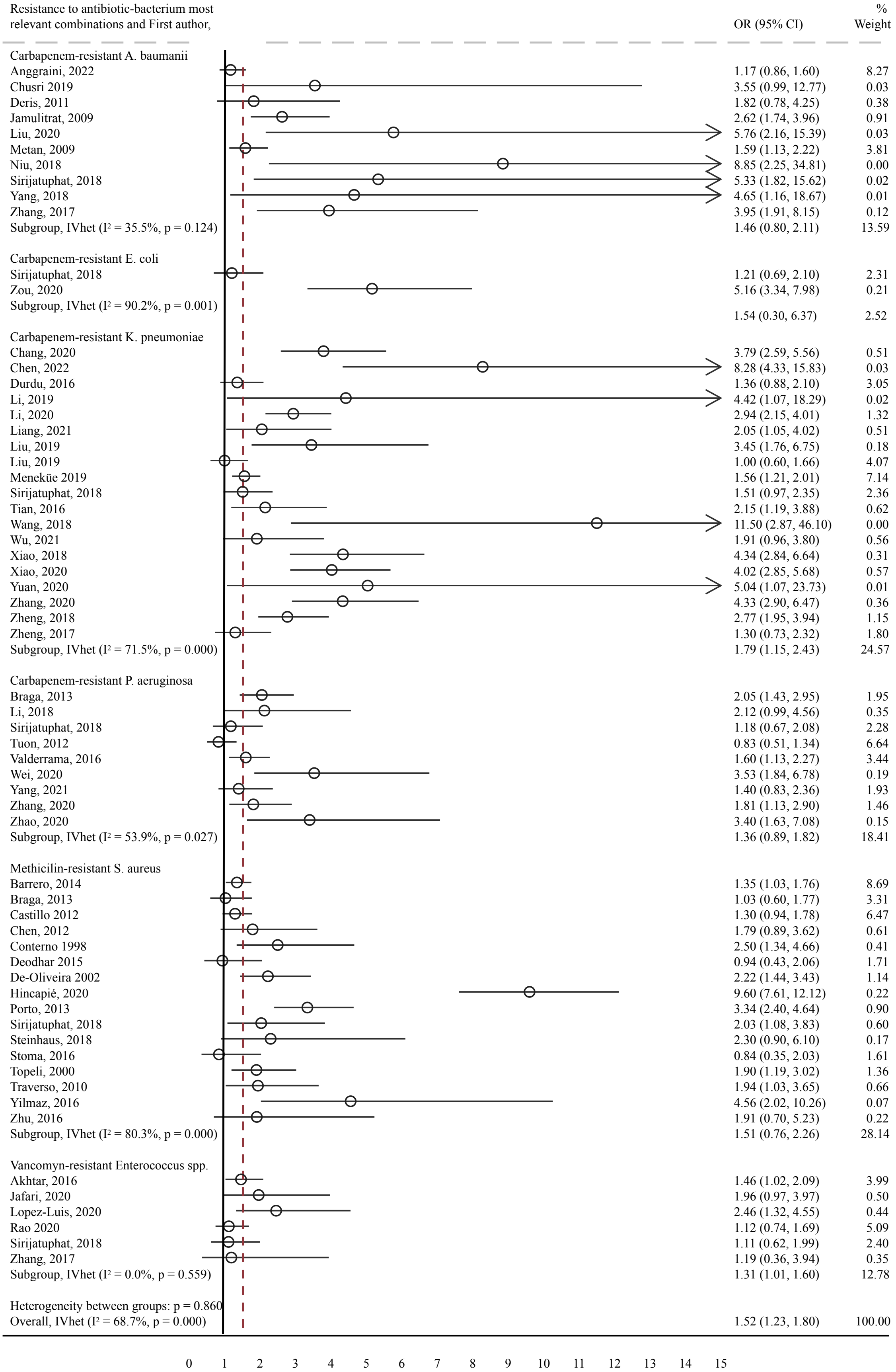

Fig AS. Meta-analysis results disaggregated by carbapenem-resistant Enterobacteriaceae for mortality (N=26 studies). Weights are from Doi's IVhet model. Dashed red line stands for the overall IVhet, whereas the black vertical line for OR=1. Estimate's p-value was <0.001 for CREN (carbapenem-resistant Enterobactericieae).

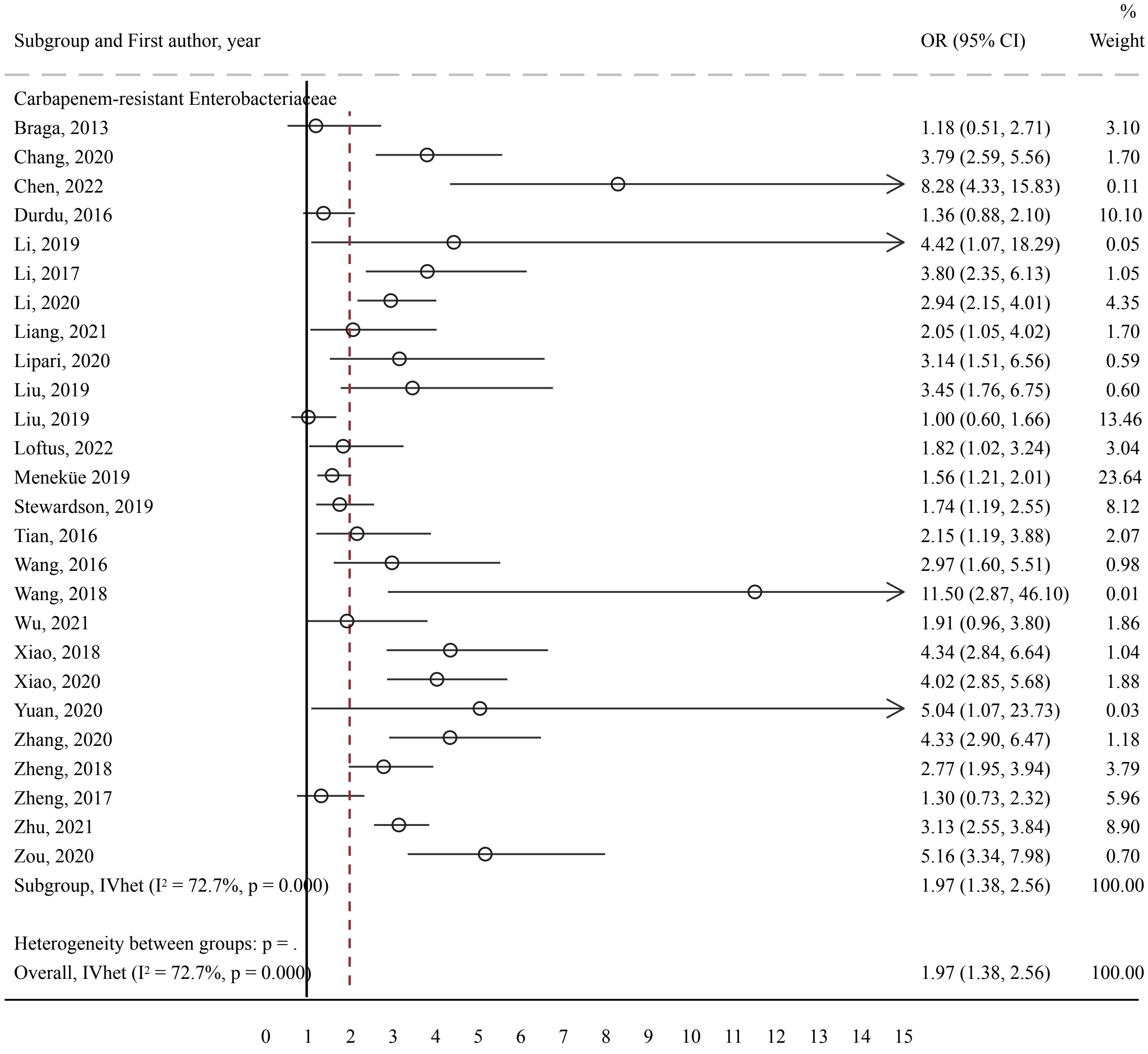

Fig AT. Meta-analysis results disaggregated by specific and prioritised antibiotic-bacterium pairs for LOS (N= 13 studies). Weights are from Doi's IVhet model. Dashed red line stands for the overall IVhet, whereas the black vertical line for 0. CRAB= Carbapenem-resistant Acinetobacter baumanii, CREN= Carbapenem-resistant Enterobactericiae, CREC= Carbanpenem-resistant Escherichia coli, CRKP= Carbapenem-resistant Klebsiella pneumoniae, CRPA= Carbapenem-resistant Pseudomonas auregionsa, MRSA= Methicilin-resistant Staphylococcus aureus, VRE= Vancomycin-resistant Enterococcus faecium/faecalis. Estimates' p-value were 0.104, <0.001, 1.000, 0.408, and 0.102 for CRAB, CRKP, CRPA, MRSA and VRE models, respectively.

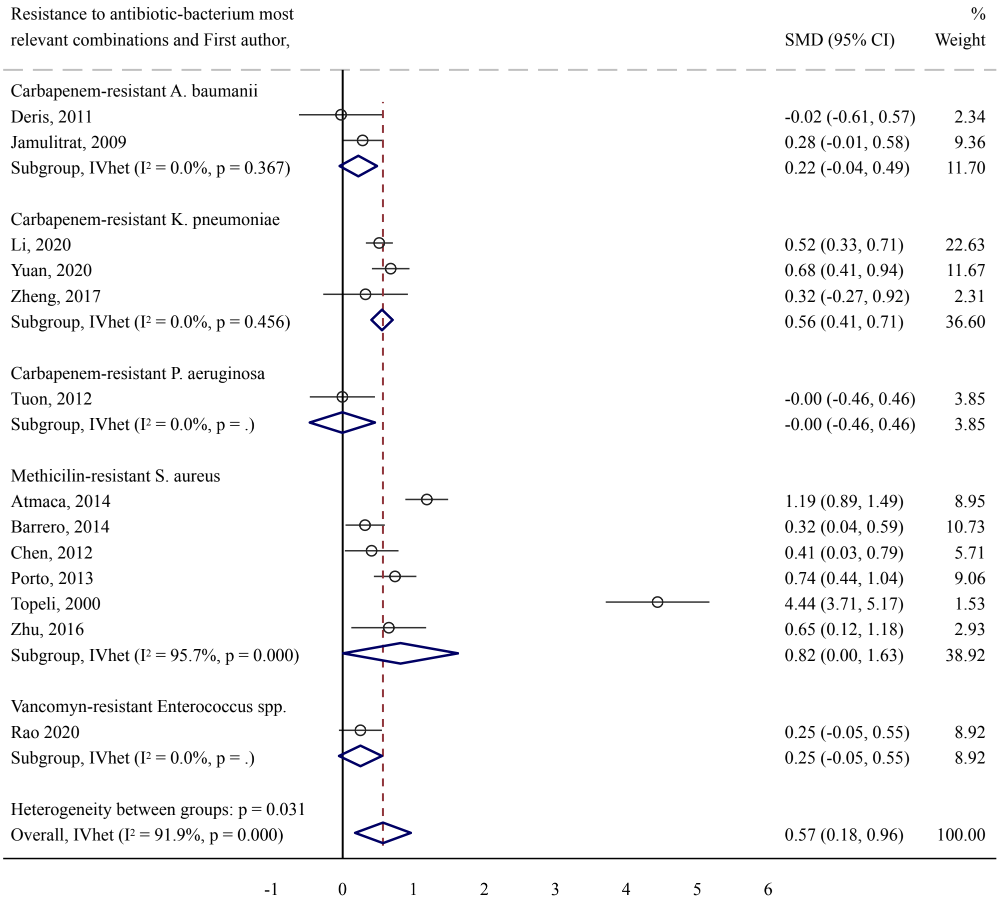

Fig AU. Meta-analysis results disaggregated by carbapenem-resistant Enterobacteriaceae for LOS (N=4 studies). Weights are from Doi's IVhet model. Dashed red line stands for the overall IVhet, whereas the black vertical line for 0. CRAB= Carbapenem-resistant Acinetobacter baumanii, CREN= Carbapenem-resistant Enterobactericiae, CREC= Carbanpenem-resistant Escherichia coli, CRKP= Carbapenem-resistant Klebsiella pneumoniae, CRPA= Carbapenem-resistant Pseudomonas auregionsa, MRSA= Methicilin-resistant Staphylococcus aureus, VRE= Vancomycin-resistant Enterococcus faecium/faecalis. Estimate's p-value was <0.001 for CREN.

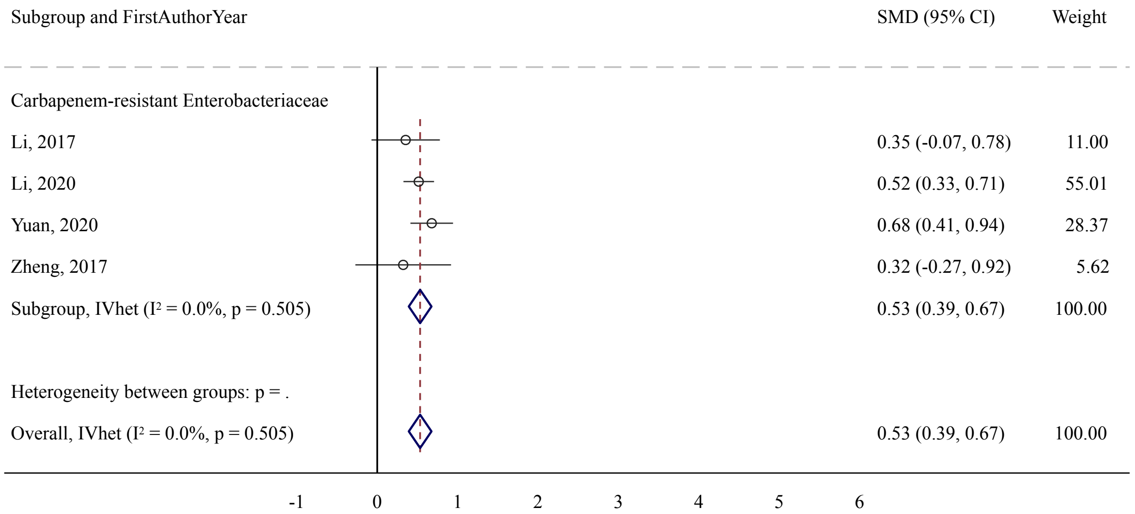

Fig AV. Meta-analysis results disaggregated by specific and prioritised antibiotic-bacterium pairs for ICU admission (N=33 studies). Weights are from Doi's IVhet model. Dashed red line stands for the overall IVhet, whereas the black vertical line for OR=1. CRAB= Carbapenem-resistant Acinetobacter baumanii, CREN= Carbapenem-resistant Enterobactericiae, CREC= Carbanpenem-resistant Escherichia coli, CRKP= Carbapenem-resistant Klebsiella pneumoniae, CRPA= Carbapenem-resistant Pseudomonas auregionsa, MRSA= Methicilin-resistant Staphylococcus aureus, VRE= Vancomycin-resistant Enterococcus faecium/faecalis. Estimates' p-value were 0.198, <0.001, <0.001, <0.001, 0.038, 0.112, and 0.152 for CRAB, CREC, CRKP, CRPA, MRSA and VRE models, respectively.

Resistance to antibiotic-bacterium most relevant combinations and First author,	Treatment n/N	Control n/N	OR (95% CI)	% Weight
Carbapenem-resistant A. baumanii				
Anggraini, 2022	60/72	49/72	1.22 (1.01, 1.48)	8.94
Chusri 2019	20/31	6/11	1.18 (0.65, 2.15)	0.89
Deris, 2011	11/15	20/41	1.50 (0.97, 2.33)	1.67
Liu, 2020	129/229	26/88	1.91 (1.35, 2.68)	2.73
Yang, 2018	55/84	6/34	3.71 (1.77, 7.80)	0.58
Zhang, 2017	10/49	12/29	0.49 (0.24, 1.00)	0.65
Subgroup, IVhet	285/480	119/275	1.36 (0.85, 2.16)	15.45
(I² = 75.4%, p = 0.001)				
Carbapenem-resistant E. coli				
Zou, 2020	20/31	61/367	3.88 (2.74, 5.49)	2.65
Subgroup, IVhet	20/31	61/367	3.88 (2.74, 5.49)	2.65
(I² = 0.0%, p = .)				
Carbapenem-resistant K. pneumoniae				
Chang, 2020	26/46	33/239	4.09 (2.73, 6.14)	1.94
Chen, 2022	21/29	38/223	4.25 (2.95, 6.13)	2.38
Li, 2019	11/19	5/21	2.43 (1.03, 5.72)	0.44
Li, 2020	116/164	58/328	4.00 (3.10, 5.15)	4.97
Liang, 2021	20/56	13/47	1.29 (0.72, 2.31)	0.95
Wang, 2018	25/48	3/48	8.33 (2.70, 25.76)	0.25
Yuan, 2020	82/98	44/141	2.68 (2.07, 3.48)	4.72
Zhang, 2020	85/108	155/388	1.97 (1.68, 2.30)	13.03
Zheng, 2018	28/59	47/230	2.32 (1.60, 3.36)	2.33
Subgroup, IVhet	414/627	396/1665	2.60 (1.81, 3.75)	31.00
(I² = 82.2%, p = 0.000)				
Carbapenem-resistant P. aeruginosa				
Tuon, 2012	24/29	25/48	1.59 (1.16, 2.18)	3.15
Valderrama, 2016	26/42	73/126	1.07 (0.81, 1.41)	4.07
Yang, 2021	34/65	46/155	1.76 (1.26, 2.47)	2.84
Subgroup, IVhet	84/136	144/329	1.39 (1.02, 1.90)	10.06
(I² = 66.6%, p = 0.050)				
Methicilin-resistant S. aureus				
Arias-Ortiz, 2016	105/186	89/186	1.18 (0.97, 1.44)	8.31
Atmaca, 2014	25/99	6/99	4.17 (1.79, 9.71)	0.45
Barrero, 2014	64/102	54/102	1.19 (0.94, 1.50)	5.72
Castillo 2012	105/186	90/186	1.17 (0.96, 1.42)	8.41
Conterno 1998	54/90	13/46	2.12 (1.30, 3.47)	1.33
Hincapié, 2020	239/292	84/909	8.86 (7.17, 10.94)	7.19
Topeli, 2000	20/46	13/55	1.84 (1.03, 3.28)	0.96
Zlatian, 2018	14/23	19/40	1.28 (0.81, 2.03)	1.50
Subgroup, IVhet	626/1024	368/1623	1.91 (0.86, 4.25)	33.85
(I² = 97.4%, p = 0.000)				
Vancomyn-resistant Enterococcus spp.				
Akhtar, 2016	23/46	9/65	3.61 (1.84, 7.07)	0.71
Furtado, 2006	13/34	18/55	1.17 (0.66, 2.07)	0.98
Jafari, 2020	29.59493/51	5.2925/21	2.28 (1.05, 4.96)	0.53
Lopez-Luis, 2020	41/107	11/85	2.96 (1.62, 5.40)	0.88
Rao 2020	21/73	41/100	0.70 (0.46, 1.08)	1.72
Xu, 2015	21/31	24/54	1.52 (1.04, 2.24)	2.16
Subgroup, IVhet	148.5949/342	108.2925/380	1.48 (0.87, 2.54)	6.98
(I² = 80.2%, p = 0.000)				
Heterogeneity between groups: p = 0.000				
Overall, IVhet	1577.595/2640	1196.293/4639	1.93 (1.43, 2.62)	100.00
(I² = 93.1%, p = 0.000)				

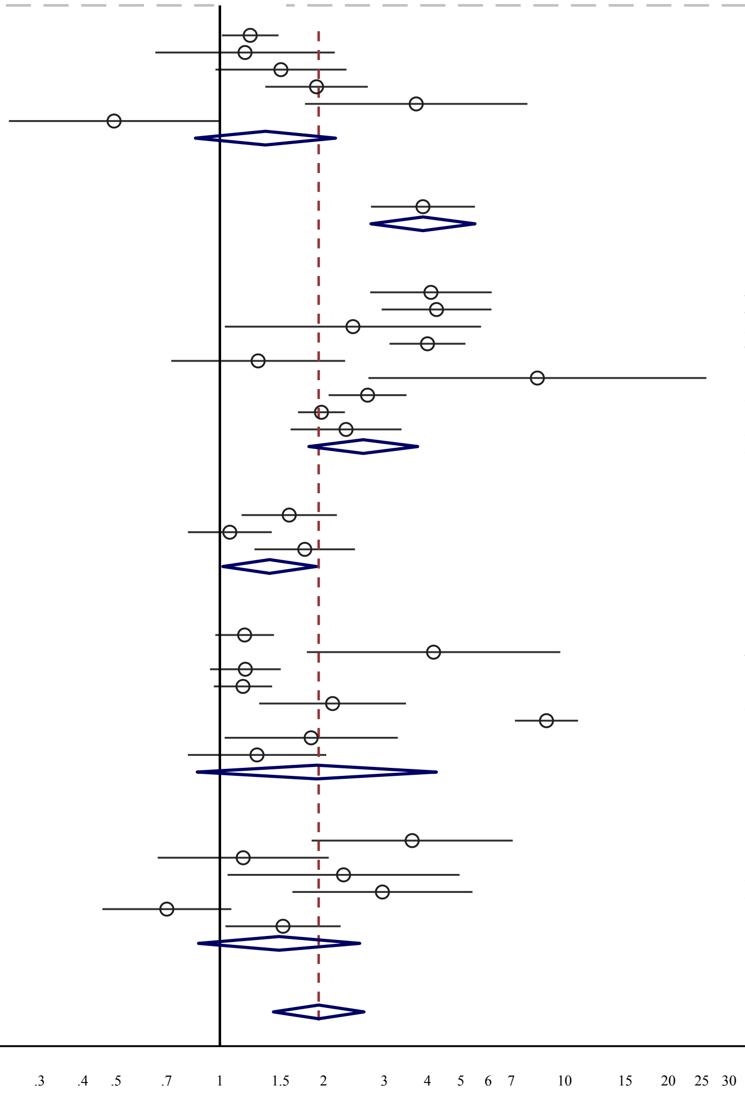

Fig AW. Meta-analysis results disaggregated by carbapenem-resistant Enterobacteriaceae for ICU admission (N= 15 studies). Weights are from Doi's IVhet model. Dashed red line stands for the overall IVhet, whereas the black vertical line for OR=1. CRAB= Carbapenem-resistant Acinetobacter baumanii, CREN= Carbapenem-resistant Enterobactericiae, CREC= Carbanpenem-resistant Escherichia coli, CRKP= Carbapenem-resistant Klebsiella pneumoniae, CRPA= Carbapenem-resistant Pseudomonas auregionsa, MRSA= Methicilin-resistant Staphylococcus aureus, VRE= Vancomycin-resistant Enterococcus faecium/faecalis. Estimates' p-value was <0.001.

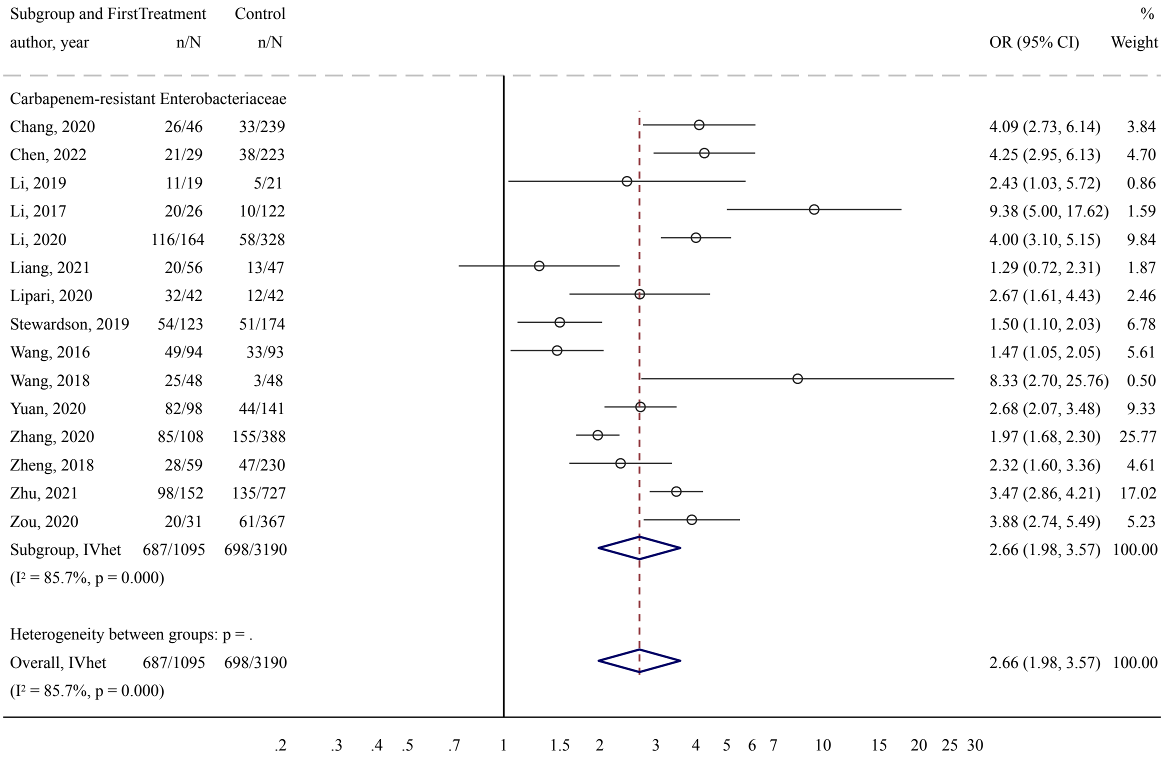

Table T. Summary of the subgroup meta-analysis results for specific antibiotic-bacterium combinations declared as important by the WHO, by outcome variable.

Outcome	Bacterium-antibiotic specific resistance combination*	OR/SMD	Lower 95% CI	Upper 95% CI	N of studies	p-value
Mortality	CRAB	1.46	0.80	2.11	10	0.126
Mortality	CREN	1.97	1.37	2.56	26	<0.001
Mortality	CREC	1.54	0.30	6.37	2	0.592
Mortality	CRKP	1.79	1.15	2.43	19	0.002
Mortality	CRPA	1.36	0.89	1.82	9	0.092
Mortality	MRSA	1.51	0.76	2.26	16	0.138
Mortality	VRE	1.31	1.01	1.60	6	0.021
LOS	CRAB	0.22	-0.04	0.49	2	0.104
LOS	CREN	0.53	0.39	0.67	4	<0.001
LOS	CREC ‡	-	-	-	-	-
LOS	CRKP	0.56	0.41	0.71	3	<0.001
LOS	CRPA ‡	0.00	-0.46	0.46	1	1.000
LOS	MRSA	0.82	0.00	1.63	6	0.048
LOS	VRE ‡	0.25	-0.05	0.55	1	0.102
ICU	CRAB	1.36	0.85	2.16	6	0.198
ICU	CREN	2.66	1.98	3.57	15	<0.001
ICU	CREC ‡	3.88	2.74	5.49	1	<0.001
ICU	CRKP	2.60	1.81	3.75	9	<0.001
ICU	CRPA	1.39	1.02	1.90	3	0.038
ICU	MRSA	1.91	0.86	4.25	8	0.112
ICU	VRE	1.48	0.87	2.54	6	0.152

LOS: Length of hospital stay. ICU: Intensive Care Unit. * All comparisons and ORs SMD computations were made in respect to their susceptible-specific counterpart. CRAB= Carbapenem-resistant *Acinetobacter baumanii*, CREN= Carbapenem-resistant *Enterobactericiae*, CREC= Carbanpenem-resistant *Escherichia coli,* CRKP= Carbapenem-resistant *Klebsiella pneumoniae*, CRPA= Carbapenem-resistant *Pseudomonas auregionsa*, MRSA= Methicilin-resistant *Staphylococcus aureus*, VRE= Vancomycin-resistant *Enterococcus faecium/faecalis.* ‡ Either non or only study reported estimates for the specific antibiotic-bacterium pair.

(7.b) Meta-analysis results dissagregated by studies' location including China and other than China

Table U. Meta-analysis subgroup results for bacterium family, and Gram-type for those studies carried out in China and other than China, by outcome.

Outcome	Bacteria family	Gram-type	Type of resistance	OR/SMD	Lower 95% CI	Upper 95% CI	N of studies	p-value
**I. China**								
Mortality	-	-	-	1.66	1.15	2.16	45	0.002
Mortality	Enterobactericieae	-	-	1.61	0.80	2.42	26	0.091
Mortality	Morelaxeae	-	-	1.98	0.57	3.39	8	0.133
Mortality	Pseudomonaceae	-	-	1.72	1.18	2.26	4	<0.001
Mortality	Enterococcus spp.	-	-	1.19	0.36	3.94	1	0.788
Mortality	Staphylococcus	-	-	1.82	0.65	2.99	2	0.124
Mortality	-	Gram-negative	-	1.66	1.11	2.21	41	0.004
Mortality	-	Gram-positive	-	1.63	0.66	2.61	3	0.164
LOS	-	-	-	0.40	0.18	0.63	8	0.001
LOS	Enterobactericieae	-	-	0.43	0.14	0.72	5	0.004
LOS	Morelaxeae	-	-	0.03	-0.34	0.41	1	0.885
LOS	Pseudomonaceae	-	-	NA	NA	NA	NA	NA
LOS	Enterococcus spp.	-	-	NA	NA	NA	NA	NA
LOS	Staphylococcus	-	-	0.49	0.18	0.80	2	0.002
LOS	-	Gram-negative	-	0.39	0.11	0.67	6	0.006
LOS	-	Gram-positive	-	0.49	0.18	0.80	2	0.002
ICU	-	-	-	2.45	1.90	3.17	25	<0.001
ICU	Enterobactericieae	-	-	2.74	2.01	3.74	15	<0.001
ICU	Morelaxeae	-	-	1.93	1.07	3.48	7	0.029
ICU	Pseudomonaceae	-	-	1.76	1.26	2.47	1	0.001
ICU	Enterococcus spp.	-	-	1.52	1.04	2.24	1	0.032
ICU	Staphylococcus	-	-	NA	NA	NA	NA	NA
ICU	-	Gram-negative	-	2.49	1.92	3.24	24	<0.001
ICU	-	Gram-positive	-	1.52	1.04	2.24	1	0.032
**II. Other than China**								
Mortality	-	-	-	1.54	1.31	1.76	48	<0.001
Mortality	Enterobactericieae	-	-	1.37	1.19	1.56	15	<0.001
Mortality	Morelaxeae	-	-	1.47	1.01	1.93	8	0.020
Mortality	Pseudomonaceae	-	-	1.30	0.93	1.67	6	0.079
Mortality	Enterococcus spp.	-	-	1.32	1.02	1.62	5	0.019
Mortality	Staphylococcus	-	-	1.51	0.70	2.32	15	0.179
Mortality	-	Gram-negative	-	1.54	1.35	1.72	30	<0.001
Mortality	-	Gram-positive	-	1.46	0.90	2.03	19	0.068
LOS	-	-	-	0.57	0.08	1.05	10	0.021
LOS	Enterobactericieae	-	-	NA	NA	NA	NA	NA
LOS	Morelaxeae	-	-	0.22	-0.04	0.49	2	0.104
LOS	Pseudomonaceae	-	-	0.14	-0.11	0.39	2	0.276
LOS	Enterococcus spp.	-	-	0.25	-0.05	0.55	1	0.102
LOS	Staphylococcus	-	-	0.91	-0.24	2.06	4	0.121
LOS	-	Gram-negative	-	0.33	0.02	0.64	5	0.037
LOS	-	Gram-positive	-	0.76	-0.13	1.65	5	0.094
ICU	-	-	-	1.60	1.14	2.25	27	0.007
ICU	Enterobactericieae	-	-	1.71	1.22	2.38	4	0.002
ICU	Morelaxeae	-	-	1.29	0.93	1.80	5	0.131
ICU	Pseudomonaceae	-	-	1.28	0.95	1.72	4	0.103
ICU	Enterococcus spp.	-	-	1.46	0.78	2.72	5	0.237
ICU	Staphylococcus	-	-	1.91	0.86	4.25	8	0.112
ICU	-	Gram-negative	-	1.36	1.15	1.61	14	<0.001
ICU	-	Gram-positive	-	1.84	0.95	3.58	13	0.071

ESBL: Extended-spectrum beta-lactamases. LOS: Length of hospital stay. ICU: Intensive Care Unit. * All comparisons and ORs SMD computations were made in respect to their susceptible-specific counterpart. NA= not applicable (missing information). Forest plots upon request.

Table V. Summary results of meta-analysis results for critical antibiotic-bacterium pathogens for those studies carried out in China and other than China, by outcome.

Country	Outcome	Bacterium-antibiotic specific resistance combination*	OR/SMD	Upper 95% CI	Lower 95% CI	N of studies included	p-value
I. China	Mortality	CRAB	4.43	1.78	7.08	4	<0.001
I. China	Mortality	CREN	2.36	1.54	3.18	21	<0.001
I. China	Mortality	CREC	5.16	2.84	7.48	1	<0.001
I. China	Mortality	CRKP	2.09	1.15	3.03	16	0.003
I. China	Mortality	CRPA	1.78	1.20	2.36	5	0.001
I. China	Mortality	MRSA	1.82	0.65	2.99	2	0.124
I. China	Mortality	VRE	1.19	0.36	3.94	1	0.788
I. China	LOS	CRAB	NA	NA	NA	NA	NA
I. China	LOS	CREN	0.53	0.39	0.67	4	<0.001
I. China	LOS	CREC ‡	NA	NA	NA	NA	NA
I. China	LOS	CRKP	0.56	0.41	0.71	3	<0.001
I. China	LOS	CRPA ‡	NA	NA	NA	NA	NA
I. China	LOS	MRSA	0.49	0.18	0.80	2	0.002
I. China	LOS	VRE ‡	NA	NA	NA	NA	NA
I. China	ICU	CRAB	1.68	0.52	5.45	3	0.394
I. China	ICU	CREN	2.78	2.03	3.79	13	<0.001
I. China	ICU	CREC ‡	3.88	2.74	5.49	1	<0.001
I. China	ICU	CRKP	2.60	1.81	3.75	9	<0.001
I. China	ICU	CRPA	1.76	1.26	2.47	1	0.001
I. China	ICU	MRSA	NA	NA	NA	NA	NA
I. China	ICU	VRE	1.52	1.04	2.24	1	0.032
II. Other than China	Mortality	CRAB	1.42	0.83	2.01	6	0.120
II. Other than China	Mortality	CREN	1.59	1.30	1.87	5	<0.001
II. Other than China	Mortality	CREC	1.21	0.50	1.91	1	0.589
II. Other than China	Mortality	CRKP	1.50	1.20	1.80	3	<0.001
II. Other than China	Mortality	CRPA	1.24	0.66	1.81	4	0.411
II. Other than China	Mortality	MRSA	1.50	0.70	2.30	14	0.183
II. Other than China	Mortality	VRE	1.31	1.01	1.61	5	0.023
II. Other than China	LOS	CRAB	0.22	-0.04	0.49	2	0.104
II. Other than China	LOS	CREN	NA	NA	NA	NA	NA
II. Other than China	LOS	CREC ‡	NA	NA	NA	NA	NA
II. Other than China	LOS	CRKP	NA	NA	NA	NA	NA
II. Other than China	LOS	CRPA ‡	0.00	-0.46	0.46	1	1.000
II. Other than China	LOS	MRSA	0.91	-0.24	2.06	4	0.121
II. Other than China	LOS	VRE ‡	0.25	-0.05	0.55	1	0.102
II. Other than China	ICU	CRAB	1.26	1.06	1.49	3	0.008
II. Other than China	ICU	CREN	1.75	0.97	3.16	2	0.063
II. Other than China	ICU	CREC ‡	NA	NA	NA	NA	NA
II. Other than China	ICU	CRKP	NA	NA	NA	NA	NA
II. Other than China	ICU	CRPA	1.27	0.86	1.88	2	0.233
II. Other than China	ICU	MRSA	1.91	0.86	4.25	8	0.112
II. Other than China	ICU	VRE	1.47	0.72	2.98	5	0.292

LOS: Length of hospital stay. ICU: Intensive Care Unit. * All comparisons and ORs SMD computations were made in respect to their susceptible-specific counterpart. CRAB= Carbapenem-resistant *Acinetobacter baumanii*, CREN= Carbapenem-resistant *Enterobactericiae*, CREC= Carbanpenem-resistant *Escherichia coli,* CRKP= Carbapenem-resistant *Klebsiella pneumoniae*, CRPA= Carbapenem-resistant *Pseudomonas auregionsa*, MRSA= Methicilin-resistant *Staphylococcus aureus*, VRE= Vancomycin-resistant *Enterococcus faecium/faecalis.* ‡ Either non or only study reported estimates for the specific antibiotic-bacterium pair. NA= not applicable (missing information)

Fig AX. Graphical results of Table V. CRAB= Carbapenem-resistant *Acinetobacter baumanii*, CREN= Carbapenem-resistant *Enterobactericiae*, CREC= Carbanpenem-resistant *Escherichia coli,* CRKP= Carbapenem-resistant *Klebsiella pneumoniae*, CRPA= Carbapenem-resistant *Pseudomonas auregionsa*, MRSA= Methicilin-resistant *Staphylococcus aureus*, VRE= Vancomycin-resistant *Enterococcus faecium/faecalis.* ‡ Either non or only study reported estimates for the specific antibiotic-bacterium pair. OR: Odds Ratio. SMD: Standardised mean difference. CI= Confidence Interval. C is for China and O for Other than China. LOS: length of hospital stay, ICU: Intensive care unit.

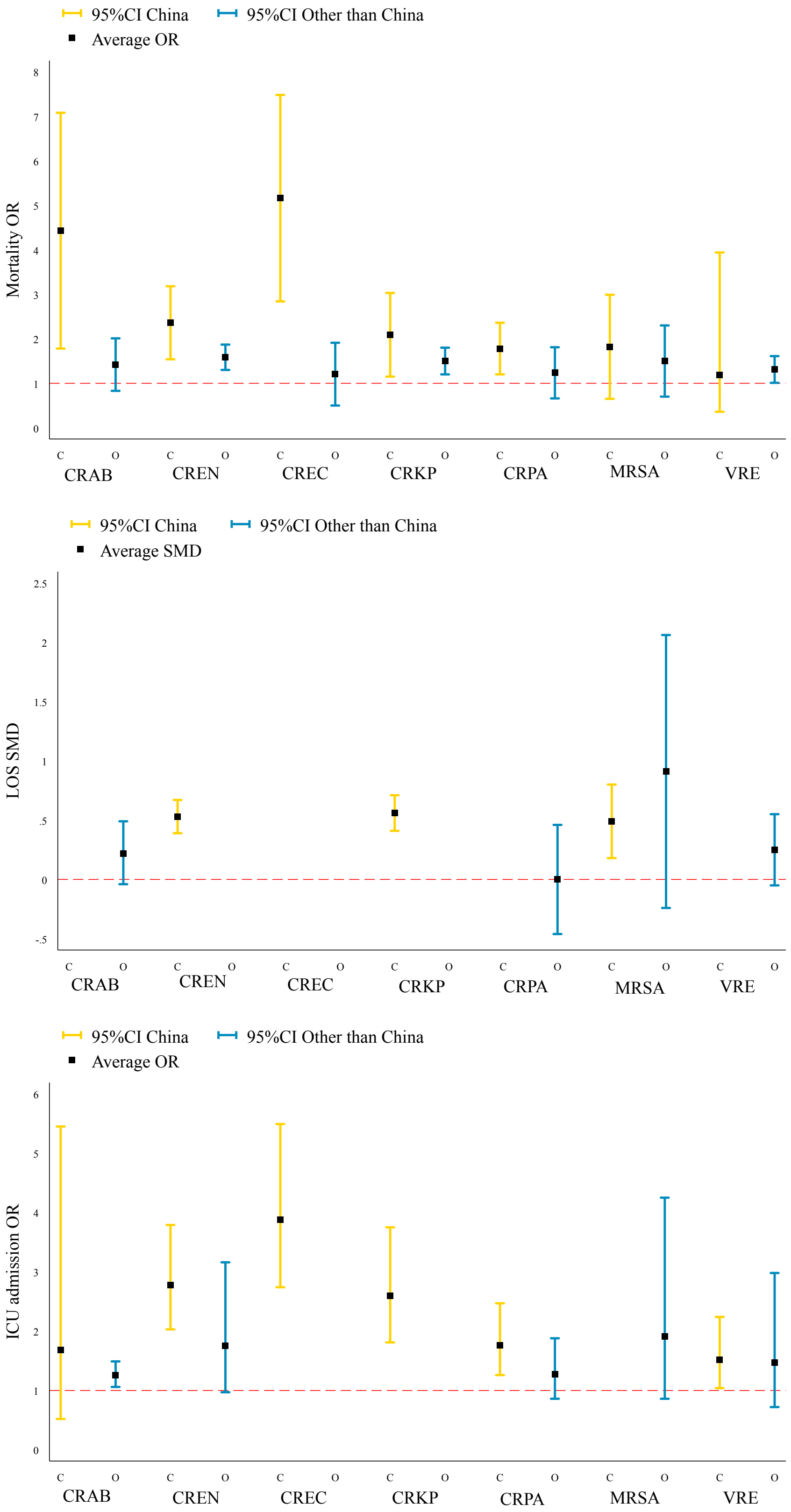

(7.c) Meta-regression using independent variables, by outcome

Table W. Meta-regression results for the mortality outcome (univariate and multivariable).

**Mortality**	Univariate meta-regression						Multivariate meta-regression		
	Model with non-imputed data			Model with Imputed data (N=100)			Imputed data (N=100 studies)		
	OR	95%CI	p-value	OR	95%CI	p-value	OR	95%CI	p-value
**Fully completed variables**									
Bacterium family									
Enterobacteriaceae	0.93	0.74,1.16	0.490	0.93	0.74,1.16	0.490			
Enterococcus	0.69	0.43,1.10	0.120	0.69	0.43,1.10	0.120			
Staphylococcus	0.86	0.60,1.22	0.380	0.86	0.60,1.22	0.380			
Pseudomonasceae	1.07	0.79,1.44	0.660	1.07	0.79,1.44	0.660			
Moraxellaceae	1.44**	1.02,2.04	0.040	1.44**	1.02,2.04	0.040	1.67***	1.18, 2.36	0.004
Bacterium type or ATB type									
Gram-negative bacteria	1.05	0.81,1.36	0.740	1.05	0.81,1.36	0.740			
B-lactam antibiotic	1.14	0.89,1.46	0.300	1.14	0.89,1.46	0.300			
WHO main region									
The Americas	0.96	0.74,1.25	0.770	0.96	0.74,1.25	0.770			
Europe	0.83	0.58,1.20	0.320	0.83	0.58,1.20	0.320			
Sout East Asia	0.66***	0.49,0.90	0.010	0.66***	0.49,0.90	0.010	0.62***	0.46, 0.85	0.004
Western Pacific region	1.34***	1.08,1.66	0.010	1.34***	1.08,1.66	0.010	1.18	0.93, 1.49	0.16
**Imputed variables, models type**		Non imputed data			Imputed data			Imputed data	
Age (ratio between ARB and ASB)	1.11	0.60,2.03	0.740	1.14	0.61,2.12	0.690			
Sex (female)	0.88	0.66,1.18	0.400	0.87	0.66,1.15	0.330			
Community-acquired infection	1.00	0.96,1.04	0.960	1.00	0.97,1.03	0.950			
Prior surgery	1.05	0.92,1.21	0.460	1.07	0.97,1.18	0.170			
Previous hospitalisation	1.02	0.97,1.07	0.460	1.02	0.99,1.06	0.250			
Commorbidities									
Any comorbidity	1.25	0.81,1.91	0.270	0.99	0.86,1.14	0.900			
Liver disease	1.14**	1.00,1.29	0.040	1.10*	1.00,1.22	0.060	1.04	0.93, 1.16	0.48
Kidney or renal disease	1.14	0.97,1.34	0.100	1.09	0.96,1.23	0.190			
Intrabdominal gastronteritis	1.06	0.88,1.26	0.520	1.06	0.87,1.28	0.570			
Hypertension	1.28**	1.04,1.59	0.020	1.11*	0.99,1.24	0.060	1.13**	1.00, 1.28	0.035
Diabetes	1.08	0.95,1.24	0.220	1.04	0.92,1.16	0.540			
Cardiovascular diseases	1.13	0.95,1.35	0.170	1.09	0.93,1.28	0.260			

Pulmonary diseases	1.08**	1.02,1.15	0.010	0.94	0.87,1.02	0.150	1.01	0.98, 1.04	0.52
Hermatologic diseases	0.92	0.82,1.03	0.130	0.95	0.74,1.24	0.720			
Solid tumour malignancy	0.92	0.63,1.34	0.660	1.02	0.99,1.06	0.150			
APACHE II score	1.07	0.94,1.23	0.290	1.01	0.98,1.05	0.470			
Source of bacteraemia									
Urinary tract source of bacteraemia	0.86	0.64,1.15	0.280	0.88	0.68,1.14	0.320			
Undetermined source of bacteraemia	1.09	0.88,1.36	0.410	1.18	1.00,1.39	0.040			
Pneumonia or respiratory	1.04**	1.01,1.07	0.020	1.02	0.99,1.06	0.130			
Intravenous cathether or vascular bactaraemia	0.95	0.84,1.07	0.390	0.99	0.88,1.11	0.890			
Constant				-			1.54***	1.23, 2.94	<0.001

OR= Odds Ratios. ARB= Antimicrobial resistant, ASB= antimicrobial susceptible. BSI= bloodstream infection. CI- Confidence interval. ATB= Antibiotic. WHO= World Health Organization. *** if p-value <0.01, ** p-vale<0.05, * p-value<0.10. N stands for the number of studies. The rest of the WHO regions were omitted because of few data were reported. Raw descriptive statistics are shown in Table F. We chose pulmonary diseases (comorbidity) over pneumonia/respiratory as source of bacteremia due to high collinearity, even though both were statistically significant in univariate analyses.

Table X. Meta-regression results for the ICU admission outcome (univariate and multivariate).

ICU admission	Univariate meta-regression						Multivariate meta-regression		
	Model with non-imputed data			Model with Imputed data (N=52)			Imputed data (N=52 studies)		
	OR	95% CI	p-value	OR	95% CI	p-value	OR	95% CI	p-value
**Fully completed variables (non-imputed)**									
Bacterium family									
Enterobacteriaceae	1.21	0.83,1.75	0.320	1.21	0.83,1.75	0.320			
Enterococcus	0.8	0.46,1.40	0.430	0.80	0.46,1.40	0.430			
Staphylococcus	1.04	0.67,1.63	0.850	1.04	0.67,1.63	0.850			
Pseudomonasceae	0.66	0.37,1.16	0.150	0.66	0.37,1.16	0.150			
Moraxellaceae	1.13	0.72,1.79	0.590	1.13	0.72,1.79	0.590			
Bacterium type or ATB type									
Gram-negative bacteria	1.08	0.73,1.58	0.710	1.08	0.73,1.58	0.710			
B-lactam antibiotic	1.24	0.86,1.81	0.250	1.24	0.86,1.81	0.250			
WHO main region									
The Americas	0.89	0.61,1.31	0.550	0.89	0.61,1.31	0.550			
Europe	0.81	0.42,1.54	0.510	0.81	0.42,1.54	0.510			
South East Asia	0.60*	0.33,1.10	0.100	0.60*	0.33,1.10	0.100	0.78	0.43,1.43	0.42
Western Pacific region	1.41*	1.00,1.99	0.050	1.41*	1.00,1.99	0.050	1.24	0.87,1.77	0.24
**Imputed variables**									
Age (ratio between ARB and ASB)	0.8	0.16,4.12	0.790	0.59	0.12,2.89	0.510			
Sex (female)	0.96	0.67,1.37	0.830	0.96	0.67,1.38	0.830			
Community-acquired infection	0.98	0.94,1.02	0.350	0.99	0.95,1.04	0.650			
Prior surgery	1.07	0.96,1.18	0.210	1.05	0.93,1.19	0.390			
Previous hospitalisation	1.02	0.98,1.07	0.310	1.02	0.96,1.08	0.470			
Commorbidities									
Any comorbidity	0.94	0.72,1.23	0.640	0.97	0.35,2.65	0.940			
Liver disease	1.11	0.94,1.32	0.220	1.36***	1.11,1.67	0.010	1.09	0.92,1.28	0.32
Kidney or renal disease	1.14	0.95,1.37	0.160	1.21	0.95,1.55	0.120			
Intrabdominal gastronteritis	1.00	0.75,1.33	0.980	0.99	0.73,1.33	0.920			
Hypertension	1.16	0.86,1.57	0.330	2.93*	0.88,9.80	0.070			
Diabetes	0.9	0.67,1.21	0.500	0.94	0.55,1.60	0.810			
Cardiovascular diseases	1.35**	1.07,1.70	0.010	1.36**	1.01,1.82	0.040	1.19	0.93,1.52	0.16
Pulmonary diseases	1.02	0.99,1.05	0.250	1.03	0.98,1.07	0.260			
Hermatologic diseases	0.98	0.89,1.09	0.760	0.95	0.83,1.08	0.410			
Solid tumour malignancy	0.76	0.53,1.08	0.130	0.42*	0.25,0.96	0.050			
APACHE II score	1.04	0.97,1.11	0.300	1.05	0.86,1.28	0.610			
Source of bacteraemia									
Urinary tract	0.63***	0.46,0.85	0.000	0.49***	0.36,0.69	0.000	0.72*	0.51,1.02	0.06

Undetermined	1.22*	0.97,1.53	0.090	0.57	0.25,1.30	0.170			
Pneumonia or respiratory	1.02	0.99,1.06	0.200	1.03	0.98,1.08	0.230			
Intravenous cathether or vascular	1.13	0.92,1.38	0.240	1.17	0.90,1.53	0.220			
Constant			-				1.61	0.73,3.52	0.23

OR= Odds Ratios. ARB= Antimicrobial resistant, ASB= antimicrobial susceptible. BSI= bloodstream infection. CI- Confidence interval. ATB= Antibiotic. WHO= World Health Organization. ICU= Intensive care unit. *** if p-value <0.01, ** p-vale<0.05, * p-value<0.10. N stands for the number of studies. The rest of the WHO regions were omitted because of few data were reported. Raw descriptive statistics are shown in Table F.

(8) Risk of bias assessment

Fig AY. Distribution of the Master scale scores by outcome. Distribution of the scores and 95% CI. Outliers are presented in circles. “x” stands for the median value. For more details, see S1 Data, MASTER scale spreadsheet.

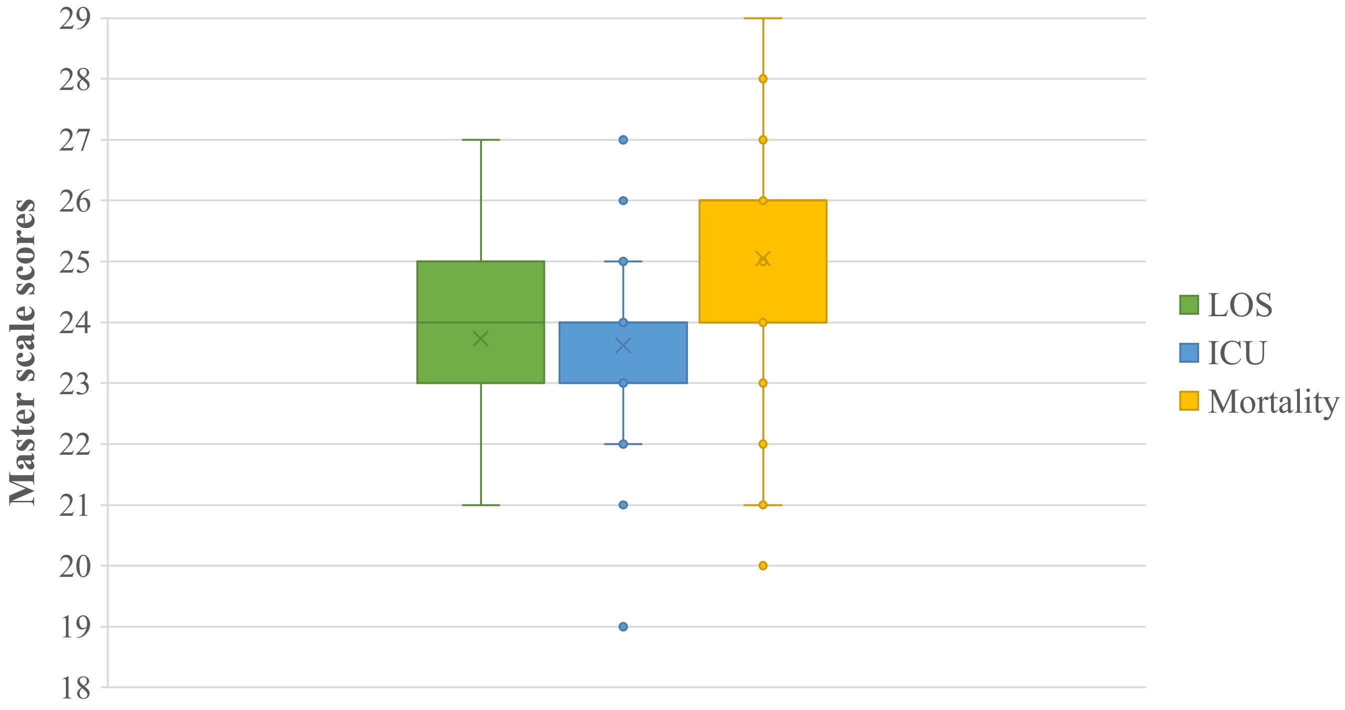

Fig AZ. Kernel density estimate of the Master scale scores by outcome. LOS= Length of hospital stay, ICU= Intensive care unit.

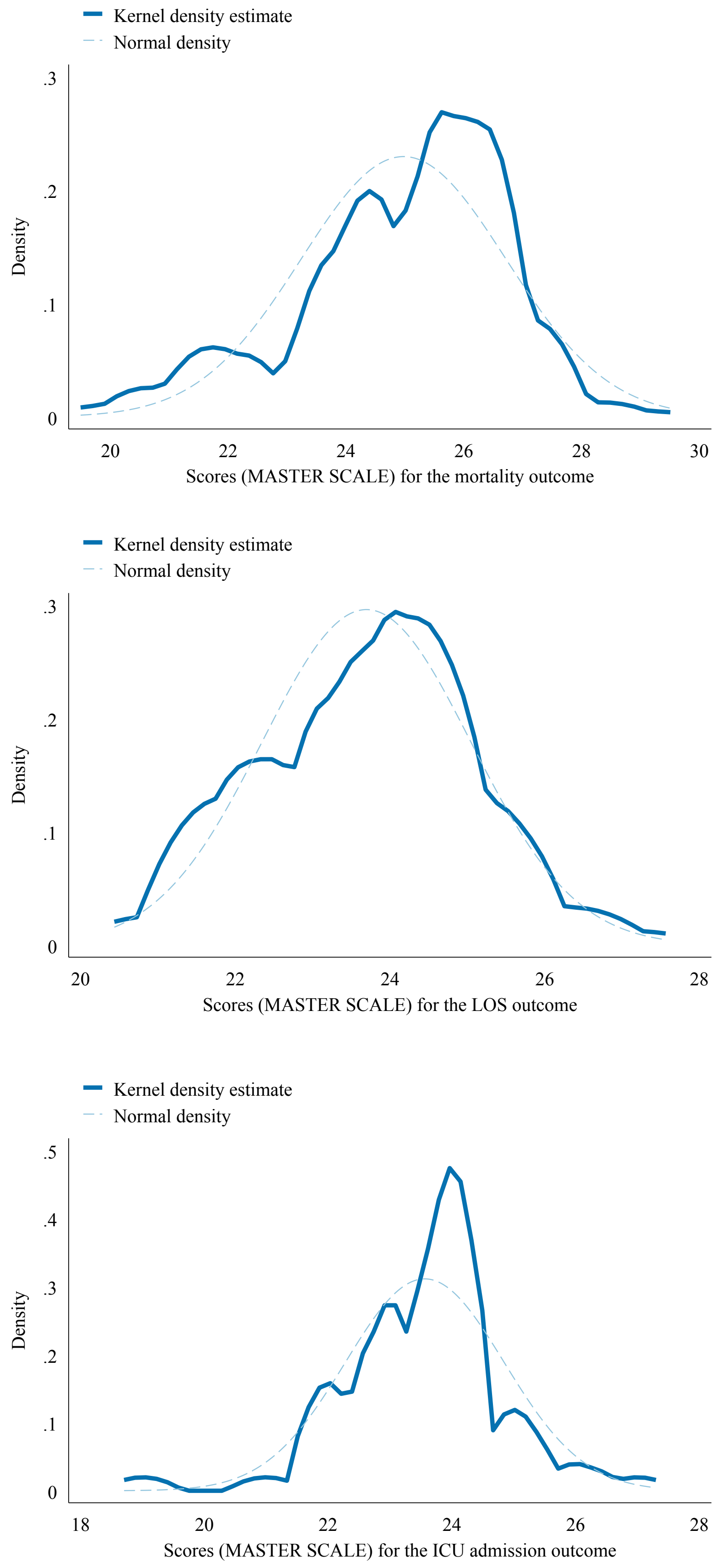

Fig BA. Percentage of fully completion by MASTER scale main safeguard and outcome. For more details, see S1 Data, MASTER scale spreadsheet. LOS= Length of hospital stay, ICU= Intensive care unit.

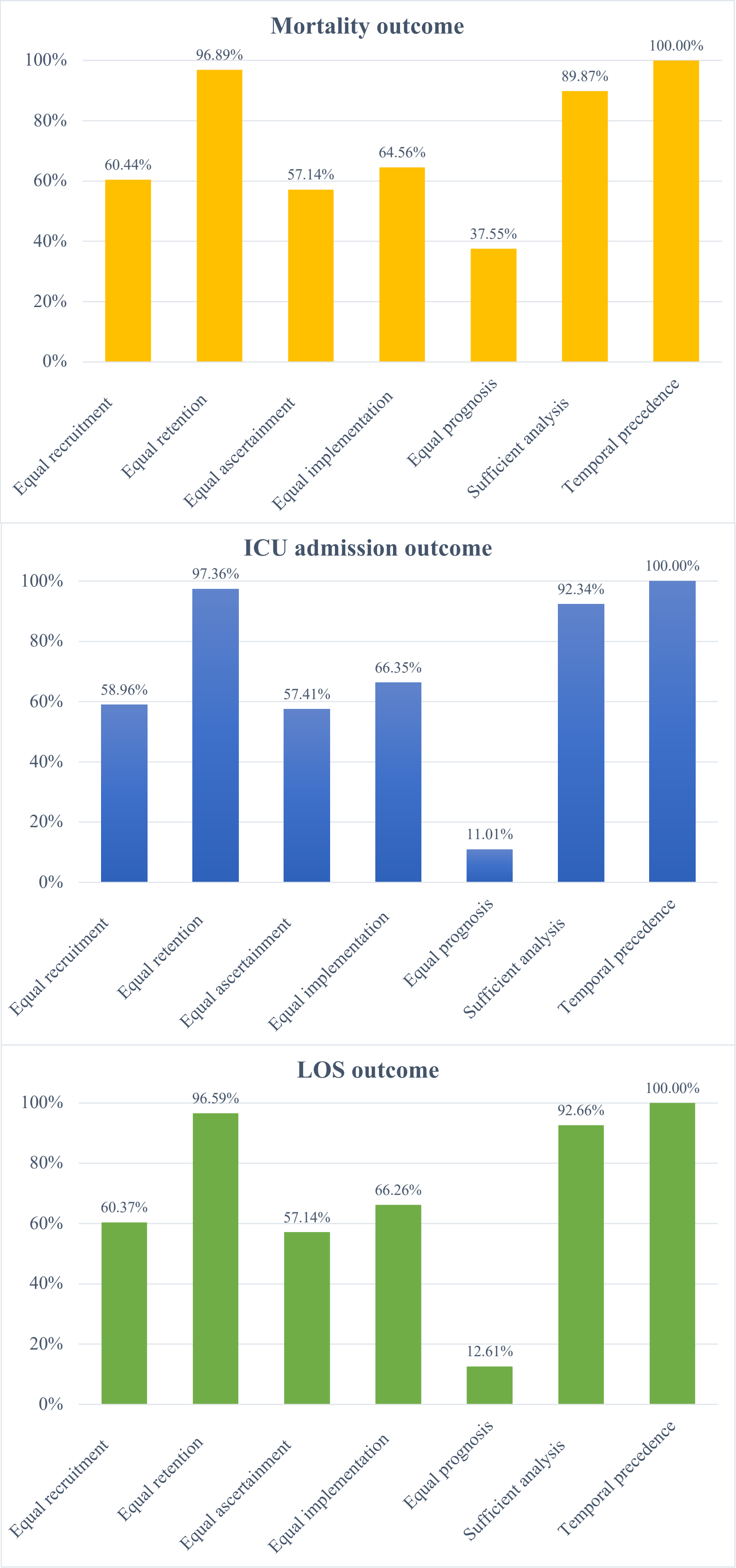

(9) Meta-analysis general results adjusted by articles's risk of bias assessment

We evaluated whether our main meta-analysis results varied by studies quality to acknowledge potential differences. We meta-analysed each outcome and subgroup by grouping the studies into high- and low-quality categories using the scores provided by the MASTER scale (i.e., studies with scores above the median were classified into high). We observed that general mortality and ICU admission estimates were more substantial (8.9% and 6.6% higher ORs compared to original estimates, respectively) among studies classified into the high-quality MASTER score. The differences are mainly guided by studies reporting Staphylococcaceae species. However, LOS was 12.24% lower compared to original estimates among high-quality MASTER score papers (14.29% higher LOS reported for studies with lower MASTER scores). Full results below in Table Y.

Nevertheless, stratification by quality might lead to a form of selection bias (collider-stratification bias) and should be taken with caution as they could induce a spurious association between effect size and precision within stratum [22].

Table Y. Summary results of the meta-analysis for the main outcome variables (N=94 studies) by separating the studies for low- [LS] and high-scores [HS] obtained from the MASTER scale.

Outcome variables	OR/SMD	95% CI		N of studies	Relative variation compared to overall estimate
		LCI	UPCI		
**I. Mortality rate[a]**	**OR**				
Overall [HS]	1.72	1.41	2.02	60	8.86%
Overall [LS]	1.36	1.06	1.66	33	-13.92%
WHO classification					
Critical pathogens (gram-negative) [HS]	1.7	1.42	1.98	50	6.92%
Critical pathogens (gram-negative) [LS]	1.34	0.87	1.80	22	-15.72%
High pathogens (gram-positive) [HS]	1.75	0.30	3.20	11	19.05%
High pathogens (gram-positive) [LS]	1.35	1.15	1.55	11	-8.16%
Bacterium family					
Enterobacteriaceae [HS]	1.81	1.36	2.27	27	21.48%
Enterobacteriaceae [LS]	1.11	0.53	1.69	13	-25.50%
Enterococcus spp [HS]	1.26	0.57	1.94	2	-4.55%
Enterococcus spp [LS]	1.37	0.97	1.76	4	3.79%
Moraxellaceae [HS]	1.48	1.07	1.88	11	-6.92%
Moraxellaceae [LS]	2.29	1.64	2.94	5	44.03%
Pseudomonadaceae [HS]	1.31	0.89	1.74	7	-4.38%
Pseudomonadaceae [LS]	1.52	1.01	2.04	3	10.95%
Staphylococcaceae [HS]	2.32	0.61	4.04	8	52.63%
Staphylococcaceae [LS]	1.34	1.08	1.59	9	-11.84%
**II. Length of stay (LOS)**	**SMD**				
Overall [HS]	0.43	0.28	0.58	9	-12.24%
Overall [LS]	0.56	-0.25	1.37	8	14.29%
WHO classification					
Critical pathogens (gram-negative) [HS]	0.43	0.20	0.66	5	16.22%
Critical pathogens (gram-negative) [LS]	0.11	-0.07	0.29	5	-70.27%
High pathogens (gram-positive) [HS]	0.43	0.20	0.65	4	-39.44%
High pathogens (gram-positive) [LS]	1.44	-0.72	3.60	3	102.82%
Bacterium family					
Enterobacteriaceae [HS]	0.55	0.40	0.69	3	28%
Enterobacteriaceae [LS]	0.03	-0.29	0.35	2	-93%
Enterococcus spp [HS]	0.25	-0.05	0.55	1	-
Enterococcus spp [LS]	-	-	-	-	-
Moraxellaceae [HS]	0.03	-0.34	0.41	1	-81%

Moraxellaceae [LS]	0.22	-0.04	0.49	2	38%
Pseudomonadaceae [HS]	0.2	-0.10	0.50	1	43%
Pseudomonadaceae [LS]	0	-0.46	0.46	1	-
Staphylococcaceae [HS]	0.49	0.22	0.76	3	-40%
Staphylococcaceae [LS]	1.44	-0.72	3.60	3	76%
**III. ICU admission**	**OR**				
Overall [HS]	2.09	1.53	2.84	30	6.63%
Overall [LS]	1.56	1.22	2.00	21	-20.41%
WHO classification					
Critical pathogens (gram-negative) [HS]	2.13	0.72	6.24	6	5.45%
Critical pathogens (gram-negative) [LS]	1.67	1.22	2.27	13	-17.33%
High pathogens (gram-positive) [HS]	2.07	1.59	2.71	24	13.74%
High pathogens (gram-positive) [LS]	1.44	0.97	2.14	8	-20.88%
Bacterium family					
Enterobacteriaceae [HS]	2.55	1.85	3.52	12	-1.54%
Enterobacteriaceae [LS]	2.38	1.33	4.27	5	-8.11%
Enterococcus spp [HS]	0.93	0.26	3.29	2	-37.16%
Enterococcus spp [LS]	1.81	1.15	2.85	4	22.30%
Moraxellaceae [HS]	1.58	1.00	2.50	8	0.64%
Moraxellaceae [LS]	1.49	0.57	3.90	4	-5.10%
Pseudomonadaceae [HS]	1.24	0.75	2.03	3	-9.49%
Pseudomonadaceae [LS]	1.54	1.23	1.94	2	12.41%
Staphylococcaceae [HS]	2.31	0.69	7.70	4	20.94%
Staphylococcaceae [LS]	1.54	1.23	1.94	2	-19.37%

WHO: World Health Organization. Differences and inconsistencies in the numbers are attributed to studies reporting multiple categories (WHO) or combined pathogens simultaneously. ICU stands for Intensive care unit. Full disaggregated results, including their respective forest plots, are shown in supplementary material, section 3. SMD stands for standardised mean difference. LCI: Lower confidence interval. UPCI: Upper confidence interval. N: Number. [a] From the total 95 studies included in the systematic review; five studies had missing data, one study only reported excess deaths for ARB BSIs at the country level, and therefore was excluded from the analysis [23], and other five studies evaluated mortality by comparison group but reported different bacteria for the sample of individuals and therefore excluded from the overall analysis but further included in subgroup analyses. [b] One study [24] reported demographic and ARB BSI data for two different pathogens and with non-duplicate episodes which were included as separate sub studies. [c] The number of studies/sub-studies differ from Table 2 because some studies did not report the standard deviation of LOS and therefore the SMD could not be computed.

(10) Guidelines for Accurate and Transparent Health Estimates Reporting (GATHER)

Table Z. Checklist of information that should be included in new reports of global health estimates [25].

Item #	Checklist item	Reported on page #
**Objectives and funding**		
**1**	Define the indicator(s), populations (including age, sex, and geographic entities), and time period(s) for which estimates were made.	Main text methods section
**2**	List the funding sources for the work.	Main text funding and acknowledgements sections
**Data Inputs**		
For all data inputs from multiple sources that are synthesized as part of the study:		
**3**	Describe how the data were identified and how the data were accessed.	Main text methods section, and supplementary material section 2-8
**4**	Specify the inclusion and exclusion criteria. Identify all ad-hoc exclusions.	Methods section and PRISMA chart (Fig 1)
**5**	Provide information on all included data sources and their main characteristics. For each data source used, report reference information or contact name/institution, population represented, data collection method, year(s) of data collection, sex and age range, diagnostic criteria or measurement method, and sample size, as relevant.	All articles (extracted) data is presented in S1 Data.
**6**	Identify and describe any categories of input data that have potentially important biases (e.g., based on characteristics listed in item 5).	Main text limitations and risk of bias assessment subsection under the results section.
For data inputs that contribute to the analysis but were not synthesized as part of the study:		
**7**	Describe and give sources for any other data inputs.	World Bank data on the list of LMICs countries[illegible] data on the list of pathogens by priority and liter[illegible] these sources are cited accordingly in methods s[illegible] and supplementary material S3-4.
For all data inputs:		
**8**	Provide all data inputs in a file format from which data can be efficiently extracted (e.g., a spreadsheet rather than a PDF), including all relevant meta-data listed in item 5. For any data inputs that cannot be shared because of ethical or legal reasons, such as third-party ownership, provide a contact name or the name of the institution that retains the right to the data.	S1 Data for tabulated data by study identified.
**Data analysis**		

**9**	Provide a conceptual overview of the data analysis method. A diagram may be helpful.	Main text methods section.
**10**	Provide a detailed description of all steps of the analysis, including mathematical formulae. This description should cover, as relevant, data cleaning, data pre-processing, data adjustments and weighting of data sources, and mathematical or statistical model(s).	Supplementary material section 1-8.
**11**	Describe how candidate models were evaluated and how the final model(s) were selected.	Methods section.
**12**	Provide the results of an evaluation of model performance, if done, as well as the results of any relevant sensitivity analysis.	We employed subgroup analyses and leave one out with multiple repetitions (see methods section)
**13**	Describe methods for calculating uncertainty of the estimates. State which sources of uncertainty were, and were not, accounted for in the uncertainty analysis.	Methods section.
**14**	State how analytic or statistical source code used to generate estimates can be accessed.	Methods section, all analyses were employed in Stata/QGIs.
**Results and Discussion**		
**15**	Provide published estimates in a file format from which data can be efficiently extracted.	Manuscript main Tables 1-3, and S1 Data.
**16**	Report a quantitative measure of the uncertainty of the estimates (e.g. uncertainty intervals).	95%CI provided for most estimates
**17**	Interpret results in light of existing evidence. If updating a previous set of estimates, describe the reasons for changes in estimates.	Introduction and discussion sections
**18**	Discuss limitations of the estimates. Include a discussion of any modelling assumptions or data limitations that affect interpretation of the estimates.	Discussion section, limitations subsection

This checklist should be used in conjunction with the GATHER statement and Explanation and Elaboration document, found on gather-statement.org

(11) PRISMA Checklist

Table AA. PRISMA Checklist.

Section and Topic	Item #	Checklist item	Location where item is reported
**TITLE**			
Title	1	Identify the report as a systematic review.	Title, page 1.
**ABSTRACT**			
Abstract	2	See the PRISMA 2020 for Abstracts checklist.	Abstract, page 2
**INTRODUCTION**			
Rationale	3	Describe the rationale for the review in the context of existing knowledge.	Introduction.
Objectives	4	Provide an explicit statement of the objective(s) or question(s) the review addresses.	Introduction.
**METHODS**			
Eligibility criteria	5	Specify the inclusion and exclusion criteria for the review and how studies were grouped for the syntheses.	Methods and supplementary material S1 Text, Table A.
Information sources	6	Specify all databases, registers, websites, organisations, reference lists and other sources searched or consulted to identify studies. Specify the date when each source was last searched or consulted.	Methods, page 4.
Search strategy	7	Present the full search strategies for all databases, registers and websites, including any filters and limits used.	Supplementary Material S1 Text, section I.
Selection process	8	Specify the methods used to decide whether a study met the inclusion criteria of the review, including how many reviewers screened each record and each report retrieved, whether they worked independently, and if applicable, details of automation tools used in the process.	Methods, page 4-5. Supplementary material, S1 Text, Table A.
Data collection process	9	Specify the methods used to collect data from reports, including how many reviewers collected data from each report, whether they worked independently, any processes for obtaining or confirming data from study investigators, and if applicable, details of automation tools used in the process.	Methods, page 5.
Data items	10a	List and define all outcomes for which data were sought. Specify whether all results that were compatible with each outcome domain in each study were sought (e.g. for all measures, time points, analyses), and if not, the methods used to decide which results to collect.	Methods, lines 75-89, page 5.
	10b	List and define all other variables for which data were sought (e.g. participant and intervention characteristics, funding sources). Describe any assumptions made about any missing or unclear information.	Methods, page 5.
Study risk of bias assessment	11	Specify the methods used to assess risk of bias in the included studies, including details of the tool(s) used, how many reviewers assessed each study and whether they worked independently, and if applicable, details of automation tools used in the process.	Methods, page 5.

Section and Topic	Item #	Checklist item	Location where item is reported
Effect measures	12	Specify for each outcome the effect measure(s) (e.g. risk ratio, mean difference) used in the synthesis or presentation of results.	Methods, page 5.
Synthesis methods	13a	Describe the processes used to decide which studies were eligible for each synthesis (e.g. tabulating the study intervention characteristics and comparing against the planned groups for each synthesis (item #5)).	Methods, page 5.
	13b	Describe any methods required to prepare the data for presentation or synthesis, such as handling of missing summary statistics, or data conversions.	Methods, pages 5-6.
	13c	Describe any methods used to tabulate or visually display results of individual studies and syntheses.	Methods, pages 5-6.
	13d	Describe any methods used to synthesize results and provide a rationale for the choice(s). If meta-analysis was performed, describe the model(s), method(s) to identify the presence and extent of statistical heterogeneity, and software package(s) used.	Methods, page 5-6.
	13e	Describe any methods used to explore possible causes of heterogeneity among study results (e.g. subgroup analysis, meta-regression).	Methods, statistical analyses, page 5.
	13f	Describe any sensitivity analyses conducted to assess robustness of the synthesized results.	Methods, page 6, sensitivity analyses.
Reporting bias assessment	14	Describe any methods used to assess risk of bias due to missing results in a synthesis (arising from reporting biases).	Methods, study quality and risk assessment subtitle, page 5.
Certainty assessment	15	Describe any methods used to assess certainty (or confidence) in the body of evidence for an outcome.	Methods, page 6.
**RESULTS**			
Study selection	16a	Describe the results of the search and selection process, from the number of records identified in the search to the number of studies included in the review, ideally using a flow diagram.	Results, page 6. Fig 1, main manuscript.
	16b	Cite studies that might appear to meet the inclusion criteria, but which were excluded, and explain why they were excluded.	Fig 1. Citation did not apply.
Study characteristics	17	Cite each included study and present its characteristics.	Table 1, main manuscript.
Risk of bias in studies	18	Present assessments of risk of bias for each included study.	Supplementary material S1 Data. Main manuscript reports a summary on page 18.
Results of individual studies	19	For all outcomes, present, for each study: (a) summary statistics for each group (where appropriate) and (b) an effect estimate and its precision (e.g. confidence/credible interval), ideally using structured tables or plots.	Table 1-3, main manuscript. Supplementary material S1 Text, section 3.

Section and Topic	Item #	Checklist item	Location where item is reported
Results of syntheses	20a	For each synthesis, briefly summarise the characteristics and risk of bias among contributing studies.	Results section, risk of bias assessment and quality of the studies subtitle. Supplementary material S1 Text, section 7-8.
	20b	Present results of all statistical syntheses conducted. If meta-analysis was done, present for each the summary estimate and its precision (e.g. confidence/credible interval) and measures of statistical heterogeneity. If comparing groups, describe the direction of the effect.	Results section, page 6-18.
	20c	Present results of all investigations of possible causes of heterogeneity among study results.	Supplementary material S1 Text. S1 Data.
	20d	Present results of all sensitivity analyses conducted to assess the robustness of the synthesized results.	Results, sensitivity analyses subtitle. Supplementary material S1 Text, section 6-7.
Reporting biases	21	Present assessments of risk of bias due to missing results (arising from reporting biases) for each synthesis assessed.	Results, risk of bias assessment subtitle.. Supplementary material (S1 Data), and Supplementary material S1 Text, section 8.
Certainty of evidence	22	Present assessments of certainty (or confidence) in the body of evidence for each outcome assessed.	Results.
**DISCUSSION**			
Discussion	23a	Provide a general interpretation of the results in the context of other evidence.	Discussion, page 24.
	23b	Discuss any limitations of the evidence included in the review.	Discussion, page 24-25.
	23c	Discuss any limitations of the review processes used.	Discussion, page 24-25.
	23d	Discuss implications of the results for practice, policy, and future research.	Discussion, , page 24-25.
**OTHER INFORMATION**			
Registration and protocol	24a	Provide registration information for the review, including register name and registration number, or state that the review was not registered.	Registered on PROSPERO: CRD42021264056. Stated in page 2.
	24b	Indicate where the review protocol can be accessed, or state that a protocol was not prepared.	Protocol can be accessed through PROSPERO (ID provided).
	24c	Describe and explain any amendments to information provided at registration or in the protocol.	No amendments were registered.

Section and Topic	Item #	Checklist item	Location where item is reported
Support	25	Describe sources of financial or non-financial support for the review, and the role of the funders or sponsors in the review.	Financial support was disclosed. There is no role of the funding source in the review.
Competing interests	26	Declare any competing interests of review authors.	Declared. No competing interests reported.
Availability of data, code and other materials	27	Report which of the following are publicly available and where they can be found: template data collection forms; data extracted from included studies; data used for all analyses; analytic code; any other materials used in the review.	All the information and data can be accessed through the main manuscript, and supplementary material S1 Text and S1 Data.

(12) Additional references

1. Murray Ch J. Comprehensive systematic analysis of global epidemiology: definitions, methods, simplification of DALYs, and comparative results from the Global Burden of Disease Study 2010. Supplement to: Murray CJL, Ezzati M, Flaxman AD, Lim S, Lozano R, Michaud C, et al. GBD 2010: design, definitions, and metrics. Lancet. 2012;380:2063-6.
2. Roth G. Global Burden of Disease Collaborative Network. Global Burden of Disease Study 2017 (GBD 2017) Results. Seattle, United States: Institute for Health Metrics and Evaluation (IHME), 2018. The Lancet. 2018;392:1736-88.
3. Organisation for Economic Cooperation and Development. Working age population 2022 [cited 2022 10th of October]. Available from: https://data.oecd.org/pop/working-age-population.htm#:~:text=The%20working%20age%20population%20is%20defined%20as%20those%20aged%2015%20to%2064.
4. Tan SS, Bakker J, Hoogendoorn ME, Kapila A, Martin J, Pezzi A, et al. Direct cost analysis of intensive care unit stay in four European countries: applying a standardized costing methodology. Value in Health. 2012;15(1):81-6.
5. Evans J, Kobewka D, Thavorn K, D'Egidio G, Rosenberg E, Kyeremanteng K. The impact of reducing intensive care unit length of stay on hospital costs: evidence from a tertiary care hospital in Canada. Canadian Journal of Anesthesia/Journal canadien d'anesthésie. 2018;65(6):627-35.
6. Oostenbrink JB, Buijs-Van der Woude T, van Agthoven M, Koopmanschap MA, Rutten FF. Unit costs of inpatient hospital days. Pharmacoeconomics. 2003;21(4):263-71.
7. Rosenthal VD, Guzman S, Migone O, Crnich CJ. The attributable cost, length of hospital stay, and mortality of central line-associated bloodstream infection in intensive care departments in Argentina: a prospective, matched analysis. Am J Infect Control. 2003;31(8):475-80.
8. Sogayar AM, Machado FR, Rea-Neto A, Dornas A, Grion CM, Lobo SM, et al. A multicentre, prospective study to evaluate costs of septic patients in Brazilian intensive care units. Pharmacoeconomics. 2008;26(5):425-34.
9. Cong Y. Ethical challenges in critical care medicine: a Chinese perspective. The Journal of medicine and philosophy. 1998;23(6):581-600.
10. Olivera COE, Urrego KAG, Duque MG, Góngora EM. Costos de atención en UCI de un Hospital universitario de Bogotá DC. Revista Repertorio de Medicina y Cirugía. 2006;15(3):133-42.
11. Irava W, Pellny M, Khan I. Costing study of selected health facilities in Fiji. Fiji: Ministry of Health. 2012.
12. Peter JV, Thomas K, Jeyaseelan L, Yadav B, Sudarsan TI, Christina J, et al. Cost of intensive care in India. International journal of technology assessment in health care. 2016;32(4):241-5.
13. Ensor T, Indradjaya S. The costs of delivering health services in Indonesia: report on a prospective survey 2010-2011. Jakarta: Kementrian Kesehatan Republik Indonesia. 2012.
14. Soleymani F. Costs of hospital-acquired infection for patients hospitalized in intensive care unit of an Iranian referral hospital. Medical journal of the Islamic Republic of Iran. 2018;32:67.
15. Aung YN, Nur AM, Ismail A, Aljunid SM. Determining the cost and length of stay at intensive care units and the factors influencing them in a teaching hospital in Malaysia. Value in health regional issues. 2020;21:149-56.
16. Velázquez LDS. Análisis de costos en las Unidades de Terapia Intensiva mexicanas. Estudio multicéntrico. Medicina Crítica. 2010;24(4):159-66.
17. Haque A, Naveed-ur-Rehman Siddiqui RK, Hoda M, Lakahni G, Hooda K. Cost of care in a paediatric intensive care unit of a tertiary-care university hospital of Pakistan. Trauma. 2015;21:14.1.
18. Lorenzovici L, Székely A, Csanádi M, Gaál P. Cost assessment of inpatient care episodes of stroke in Romania. Frontiers in Public Health. 2020;8.
19. Mahomed S, Mahomed O. Cost of intensive care services at a central hospital in South Africa. South African Medical Journal. 2019;109(1):35-9.
20. Khwannimit B, Bhurayanontachai R. The direct costs of intensive care management and risk factors for financial burden of patients with severe sepsis and septic shock. Journal of critical care. 2015;30(5):929-34.
21. Kockaya PD, Kavuncubasi S, Kockaya G. Cost of Intensive Care Stay in Turkey: In the View of Payer and Health Care Provider. Value in Health. 2013;16(7):A466.
22. Stone J, Gurunathan U, Glass K, Munn Z, Tugwell P, Doi SA. Stratification by quality induced selection bias in a meta-analysis of clinical trials. Journal of Clinical Epidemiology. 2019;107:51-9.
23. Lim C, Takahashi E, Hongsuwan M, Wuthiekanun V, Thamlikitkul V, Hinjoy S, et al. Epidemiology and burden of multidrug-resistant bacterial infection in a developing country. eLife. 2016;5(September). doi: 10.7554/eLife.18082. PubMed Central PMCID: PMC27599374.

24. López-Luis BA, Sifuentes-Osornio J, Lambraño-Castillo D, Ortiz-Brizuela E, Ramírez-Fontes A, Tovar-Calderón YE, et al. Risk factors and outcomes associated with vancomycin-resistant Enterococcus faecium and ampicillin-resistant Enterococcus faecalis bacteraemia: A 10-year study in a tertiary-care centre in Mexico City. J Glob Antimicrob Resist. 2020;24:198-204. Epub 2020/12/29. doi: 10.1016/j.jgar.2020.12.005. PubMed PMID: 33359937.

25. Stevens GA, Alkema L, Black RE, Boerma JT, Collins GS, Ezzati M, et al. Guidelines for accurate and transparent health estimates reporting: the GATHER statement. PLoS medicine. 2016;13(6):e1002056.
